# Assessing the safety and efficacy of TAVR compared to SAVR in low-to-intermediate surgical risk patients with aortic valve stenosis: An overview of reviews

**DOI:** 10.1016/j.ijcard.2020.04.022

**Published:** 2020-09-01

**Authors:** Roisin Mc Morrow, Christine Kriza, Patricia Urbán, Valeria Amenta, Juan Antonio Blasco Amaro, Dimitris Panidis, Hubert Chassaigne, Claudius Benedict Griesinger

**Affiliations:** European Commission, Joint Research Centre (JRC), Ispra, Italy

**Keywords:** Transcatheter aortic valve implantation, Transcatheter aortic valve replacement, TAVI, TAVR, Surgical aortic valve replacement, SAVR

## Abstract

**Background:**

Transcatheter aortic valve replacement (TAVR) was initially introduced to treat patients with aortic valve stenosis (AS) at high-risk for surgical aortic valve replacement (SAVR). Today, there is ample evidence supporting TAVR in high-risk groups. However, in recent years TAVR has been extended to low-to intermediate risk groups and relevant clinical evidence is still emerging, leaving some uncertainties.

**Methods:**

To obtain information on TAVR versus SAVR in low-to intermediate risk groups, we conducted an overview of systematic reviews following PRISMA guidelines and based on a systematic search of EMBASE, MEDLINE, Cochrane and CRD databases. We focused on systematic reviews assessing mortality and VARC 2 as clinical outcomes.

**Results:**

The majority of the 11 systematic reviews included in our study reported no differences in mortality between TAVR and SAVR at short and long-term follow-up times. Two reviews that included the most recent RCTs on low-risk patients reported a decreased mortality risk with TAVR at one-year follow-up. Regarding the secondary endpoints of stroke and MI, the majority of studies presented similar results for TAVR and SAVR. Acute Kidney Injury, Bleeding Complications, Atrial Fibrillation were less frequent with TAVR, with lower risk of Permanent Pacemaker Implantation and Aortic Regurgitation with SAVR.

**Conclusions:**

Our overview indicated that TAVR is a promising intervention for low-to-intermediate surgical risk patients; however additional evidence from longer term follow-up is needed to confirm these findings. This overview highlights inconsistencies about reporting and presentation of data, most notably limited clarity on effects of risk of bias on trial results.

## Highlights

• We reviewed the evidence for TAVR and SAVR in low-to intermediate risk patients.
• Most reviews found no difference in mortality between TAVR and SAVR.
• Acute Kidney Injury, Bleeding and Atrial Fibrillation were less frequent with TAVR.
• Pacemaker Implantation and Aortic Regurgitation were less frequent with SAVR.
• Most reviews found no difference for Stroke and Myocardial Infarction.

## 1 Introduction

Aortic stenosis (AS) is the most common form of valvular heart disease and it is characterised by a haemodynamically significant narrowing of the aortic valve [1,2].

In the past, surgical aortic valve replacement (SAVR) has been the standard treatment of symptomatic AS. In 2002 the less invasive transcatheter aortic valve implantation/replacement (TAVI/TAVR) was introduced for the first time and since then TAVR surpassed surgery for high-risk patients.

The first clinical trials comparing TAVR with SAVR were specifically focusing on patients deemed inoperable or at high risk for surgery [3] and TAVR devices were firstly approved and applied to this specific group of patients. However, most patients with severe AS are at intermediate or low surgical risk [4] (the EuroSCORE and the STS are the risk models used to distinguish among low, moderate and high risk groups of patients) [5].

Since TAVR represents a less invasive alternative to surgery, there is a worldwide trend toward the extension of TAVR for treatment of AS also in intermediate or low-risk patients. However, investigations on safety and efficacy of TAVR specifically focused on these patient groups are still emerging. Recently, two randomized clinical trials that compare the outcomes of TAVR versus SAVR in low-risk patients (PARTNER 3 and EVOLUT low-risk) have been published [6,7]. The outcomes of the trials indicated the non-inferiority of TAVR versus SAVR. Primary endpoints such as all-cause mortality were analysed at a follow-up time of one year in Partner 3 and two years in the EVOLUT low-risk trials.

In August 2019, based on the outcomes of the above mentioned trials, FDA has approved an expanded indication of some TAVR devices to patients with severe AS who are at low-risk for death or major complications during surgery [8]. However, more data would be needed on the long-term durability, safety and effectiveness of the TAVR valves, especially in light of their possible increased use in low-risk and younger patients.

In line with the above considerations, the FDA has specifically required manufacturers of TAVR devices, as part of approval process, to continue the annual follow-up of patients up to 10 years post procedure.

In order to obtain a view of the current status of cumulative evidence on clinical safety and effectiveness of TAVR versus SAVR interventions in low-to-intermediate surgical risk patients with AS, we evaluated information from systematic reviews.

In our overview of systematic reviews we summarise the results of published systematic reviews, with the aim of facilitating access to the full range of information available and to support healthcare decision making [9,10]. We do not attempt to quantitatively analyse the reported results in a meta-analysis. This is in line with guidance suggesting that data from individual studies should not be used more than once as this would give too much statistical power, risking a misleading estimate which may be overly precise [11].

To our knowledge, this is the first overview of reviews on this topic. Hence, the aim of the present study is: (a) to compare evidence from published systematic reviews of randomised clinical trials (RCTs) and observational studies regarding the efficacy and safety of TAVR versus SAVR in low-to-intermediate risk patients, (b) to look into consistency of main outcomes across different reviews and (c) assess methodological quality and the quality of evidence presented in systematic reviews.

## 2 Methodology

We performed an overview of systematic reviews assessing the safety and efficacy of TAVR compared to SAVR in low-to intermediate surgical risk patients with severe aortic valve stenosis.

### 2.1 Review protocol

We conducted this overview of systematic reviews with a previously registered and published protocol, PROSPERO 2020 CRD reference: CRD42020151651. Our systematic review was performed in accordance with the Preferred Reporting Items for Systematic Reviews and Meta-Analyses (PRISMA) [12], including the PRISMA Checklist. We used the systematic review methods outlined in the Cochrane Handbook, specifically focusing on guidance for overviews of reviews [13].

The PICO strategy was applied to formulate the main research question. “Outcome” was not included in the search strategy since this information is not always available in the title or abstract of papers. For the patient group we focused on individuals with severe AS that were categorised to be low-to-intermediate risk for surgery defined by EuroSCORE (<20%) and STS-PROM (<10%) [5]. We wanted to assess how TAVR compares to SAVR in terms of mortality as the primary endpoint and secondary endpoints (based on VARC 2 standardised endpoints).

### 2.2 Selection criteria

We included only peer-reviewed systematic reviews in our overview. Included studies had to report at least on the primary outcome of mortality for TAVR compared to SAVR in low-to intermediate patient risk groups [14]. We excluded articles that only assessed high-risk patient groups or did not include sufficient information regarding low-or intermediate patient risk groups. Further exclusion criteria included a different focus of the study, other than comparing TAVR versus SAVR or only assessing specific patient population groups. See Supplementary File, Table 1 for selection criteria.

### 2.3 Search strategy

We conducted a systematic search of the literature including EMBASE, MEDLINE (PubMed), Cochrane CENTRAL and CRD databases with no restrictions to publication year (see Supplementary File, Table 2 for details on the search strategy). The searches were performed on 11th of June 2019 and the language was limited to English, Italian, Spanish, French, German and Greek. Additional papers were identified through automated database notifications of new publications according to our search terms until November 2019. The reference lists from all included reviews were checked to identify any other potential reviews to include.

### 2.4 Study selection and data collection

Two authors independently performed a screening of citations by title and abstract, with discrepancies resolved by consensus. Disagreements between authors were solved by consensus. Citations identified in the systematic search were uploaded to Mendeley reference manager and duplicates were automatically deleted. The reasons for exclusion of papers are listed in Table 8, supplementary information.

### 2.5 Quality assessment

Two authors acquired the full-text of included papers and independently assessed their methodological quality using the AMSTAR2 tool [15]. Any discrepancies were resolved through discussion between the authors. AMSTAR2 is a critical appraisal tool for assessing the methodological quality of systematic reviews that include randomized controlled trials as well as non-randomized studies. The AMSTAR2 checklist covers 16 questions aimed at evaluating the soundness of the methodology and risk of bias. While AMSTAR2 is not intended to generate an overall score, the rating system indicates the overall confidence of the systematic review with respect to its methodology used.

The quality of evidence for each outcome per systematic review was evaluated using the following domains of the Grading of Recommendations Assessment, Development, and Evaluation (GRADE) tool: risk of bias, precision and consistency [16]. Two reviewers assessed the quality of evidence for each outcome, with discrepancies resolved through consensus. Two systematic reviews had existing GRADE assessments for each outcome, which were included in Supplementary Information, Table 6.

### 2.6 Data extraction and synthesis of results

Two authors independently extracted data on study characteristics and on patients enrolled in the trials included in each systematic review. The following outcome data was extracted: mortality as the primary endpoint and secondary endpoints including stroke, acute kidney injury, atrial fibrillation, bleeding, aortic regurgitation, permanent pacemaker and myocardial infarction.

## 3 Results

**Fig. 1:**
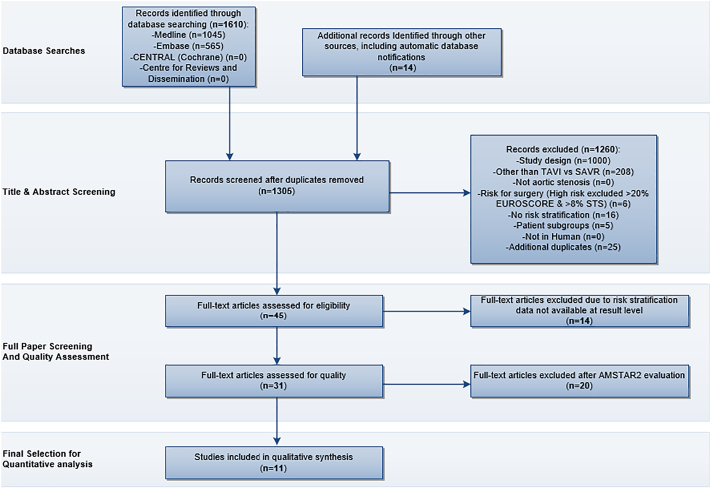
PRISMA flowchart This figure depicts the flow of information through the different phases of the systematic review. It maps out the number of records identified, included and excluded, and the reasons for exclusions.

The search strategy identified a total of 1624 articles. After removal of duplicates, 1305 articles were screened for meeting selection criteria. The remaining 31 articles were then evaluated for their methodological quality with the AMSTAR2 tool (Supplementary Information, Table 5). Systematic Reviews with a “Critically Low” score were excluded in our overview. We included all reviews with a score of “Low” quality or higher in order to provide a full picture of the available evidence, aiming to explore possible differences in findings according to AMSTAR2 scores. Eleven systematic reviews were included in the analysis [[17], [18], [19], [20], [21], [22], [23], [24], [25], [26], [27]], (see PRISMA flowchart, Fig. 1 and supplementary information, Table 8 for list of excluded records). According to the AMSTAR2 scoring, 5 systematic reviews received a “moderate quality” rating [[17], [18], [19], [20], [21]], while 6 systematic reviews were rated as “low quality” [[22], [23], [24], [25], [26], [27]].

### 3.1 Study characteristics

We identified that the systematic reviews used data from a total of 8 original RCTs and 63 observational studies in their analysis. There was a considerable overlap between the original studies included in the systematic reviews, especially for RCTs (Table 1). Patient enrolment included 8092 patients in the RCTs and 51,395 patients in the observational studies.

**Table 1:**
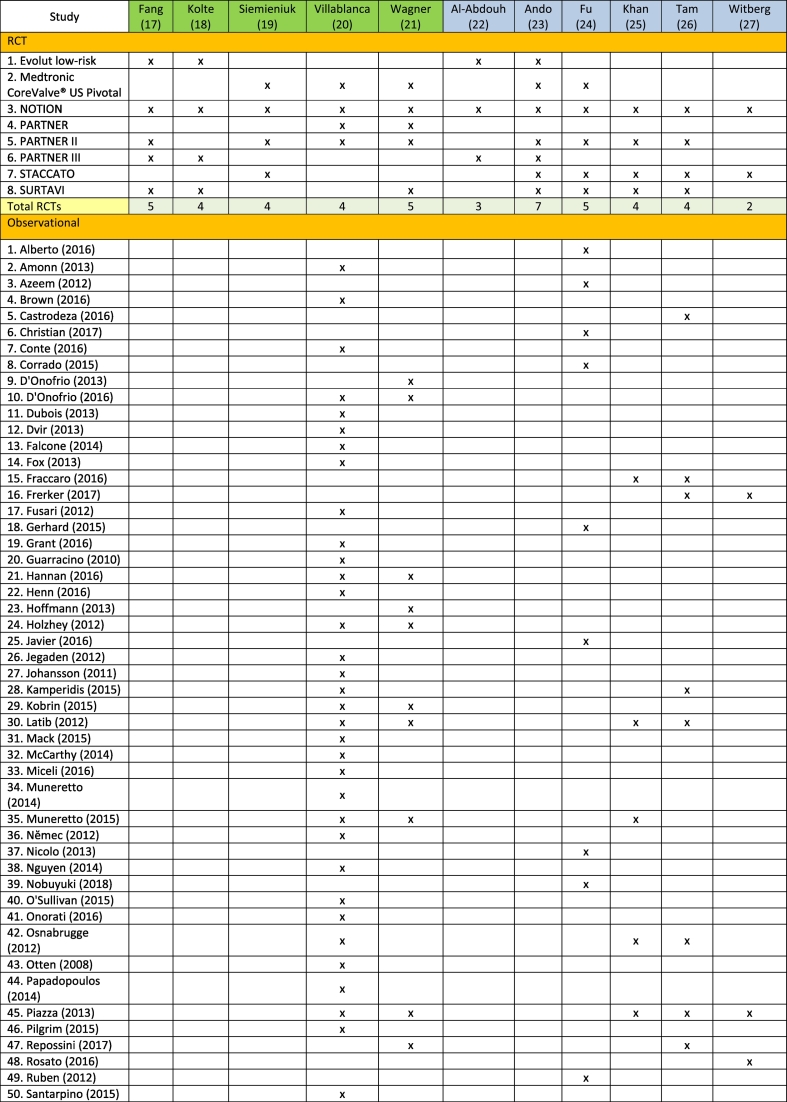

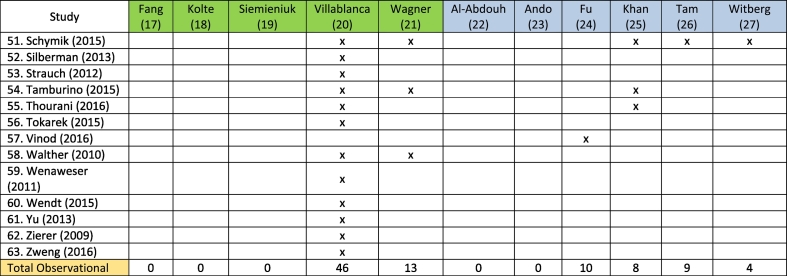
Overview of original studies included in systematic reviews, differentiated between RCTs and Observational studies.

Reviews highlighted in green have a moderate quality AMSTAR2 rating, while those marked blue have a low quality rating.

The follow-up times ranged from a mean indication of 7.5 to 36 months for RCTs and 15 to 30 months for observational studies. For details regarding study characteristics and patient baseline data, see Supplementary Information, Table 3, where we also report EuroSCORE and STS scores for each study. Notably, for those studies with higher risk scores, we only report outcomes for the low-to -intermediate risk groups.

### 3.2 Outcomes

Table 2 shows all outcome results as reported in the reviews, whereas Table 3 is a visual overview of results reported for each outcome.

**Table 2:**
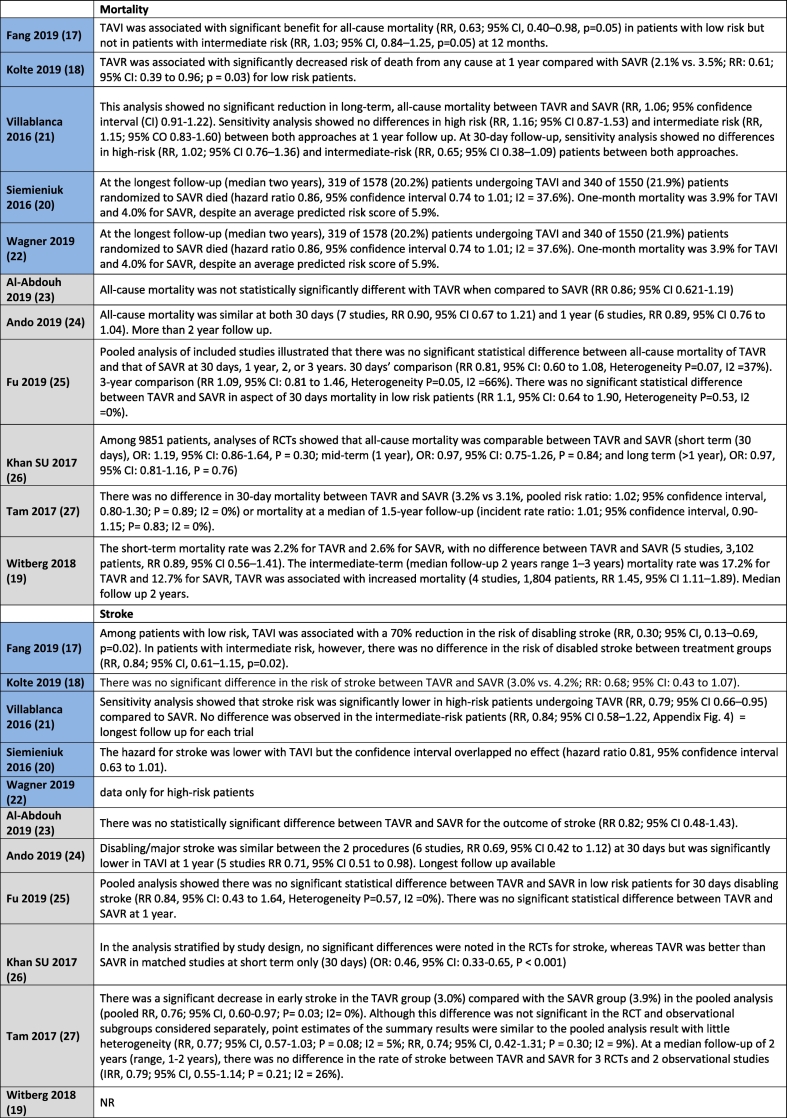

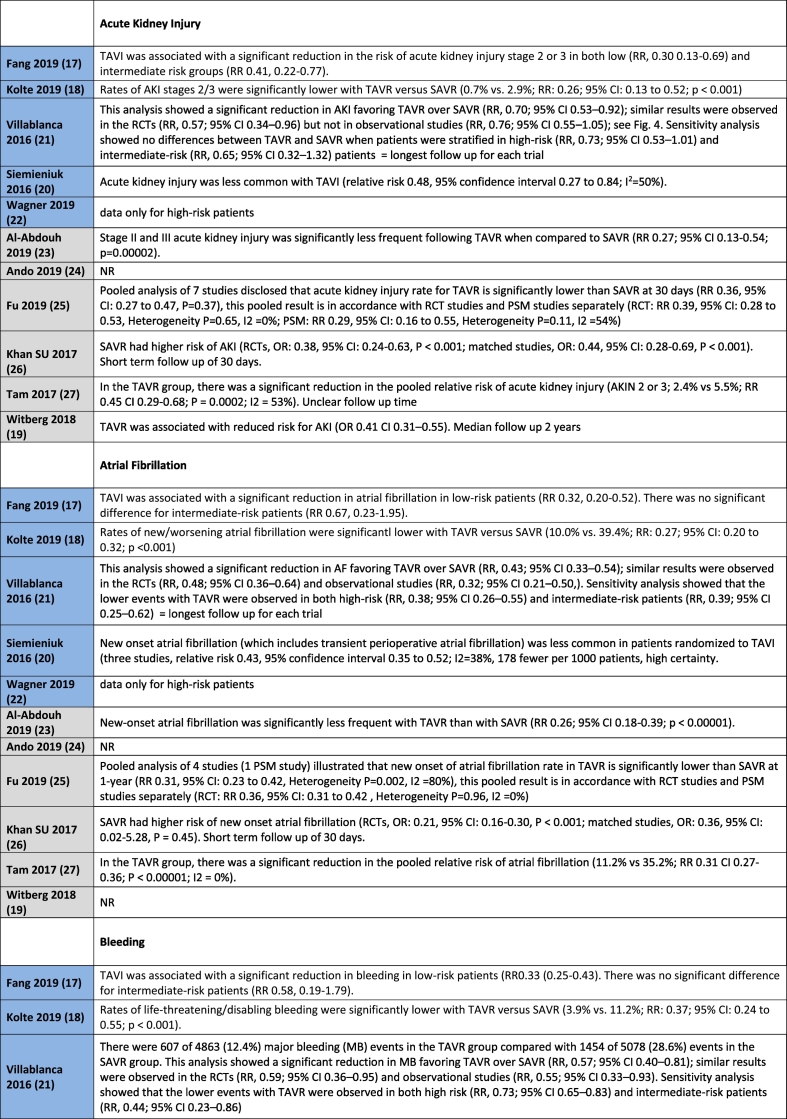

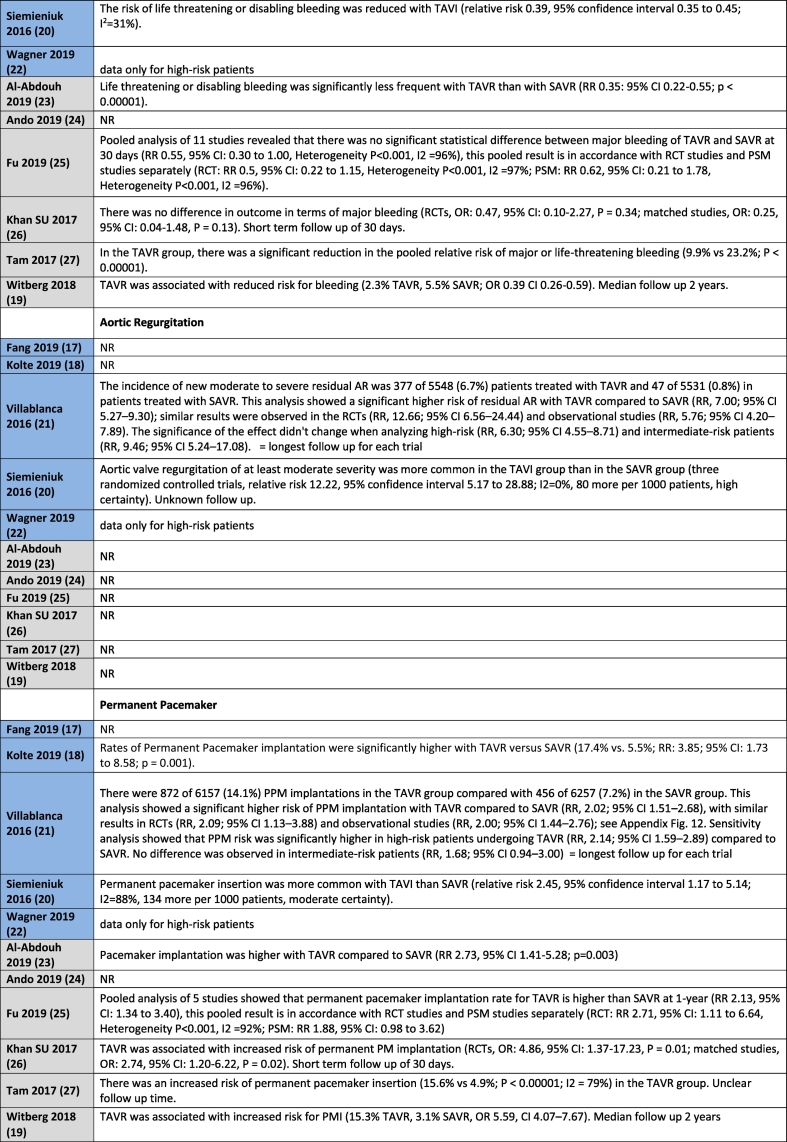

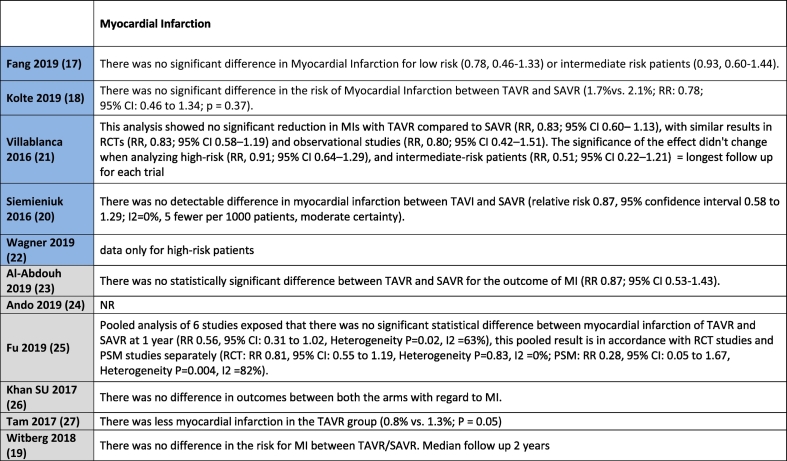
Results for the outcomes as reported in the reviews. Reviews highlighted in blue have a moderate quality AMSTAR2 rating, whereas those highlighted in grey have a low quality rating.

**Table 3:**
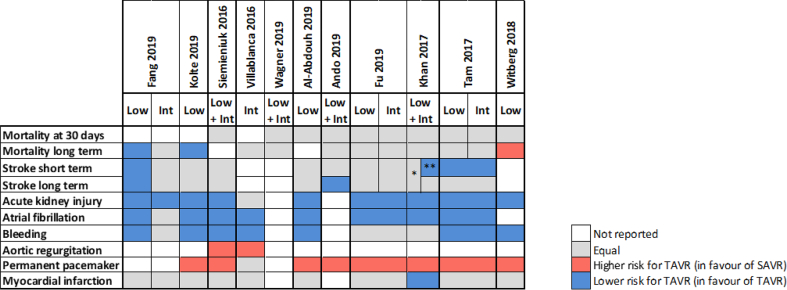
Graphical representation of the outcomes data reported in the different systematic reviews. Information for each outcome is depicted in rows, and for each publication in columns. When available in the publications data has been divided by population risk groups, otherwise is shown as pooled data (Low for low risk patients, Int for Intermediate risk patients and Low + Int for pooled data). * Only in RCTs, ** only in matched studies.

#### 3.2.1 Mortality

No difference in mortality at 30 days for intermediate or low-risk patient groups between TAVR and SAVR was reported in 8 out of 11 systematic reviews [[19], [20], [21],[23], [24], [25], [26], [27]], with the remaining three reviews not reporting on this outcome. Mortality in the longer term, ranging from 1 year to 2 years follow-up, was reported in all studies with no difference between TAVR and SAVR in the majority of eight studies [[19], [20], [21], [22], [23], [24], [25], [26]]. Witberg et al. [27] indicated that TAVR was associated with increased mortality for low-risk patients at a median follow-up of two years. Both Fang et al. [17] and Kolte et al. [18] reported that TAVR was associated with a significantly decreased risk of death from any cause at 1 year compared with SAVR for low-risk patients, with Fang noting that this finding did not apply to intermediate risk patients.

#### 3.2.2 Stroke

Nine studies reported on the outcome of stroke for intermediate or low-risk patients [[17], [18], [19], [20],[22], [23], [24], [25], [26]]. Fang et al. [17] reported a reduction in the risk of disabling stroke with TAVR in patients with low-risk, with no difference for intermediate risk patients. Villablanca et al. [20] reported no difference between TAVR and SAVR for intermediate risk patients. Siemieniuk et al. [19] indicated that the hazard for stroke was lower with TAVR but the confidence interval overlapped, indicating no effect for patients at low or intermediate risk. Ando et al. [23] reported similar results for stroke for TAVR and SAVR at 30 days, but noted that the risk of stroke was significantly lower with TAVR at 1 year follow-up for low-to intermediate patient risk groups. Fu et al. [24] stated that the stroke rate for TAVR was significantly lower than that of SAVR at 30 days in low and intermediate risk patients, with no difference reported between TAVR and SAVR at 1 year follow-up.

Khan et al. [25] reported a differentiation for stroke according to study type in their systematic review for low-to intermediate patient risk groups. No significant differences were noted in the RCTs, whereas TAVR was better than SAVR in observational studies at 30 days follow-up. Tam et al. [26] reported a significant decrease in stroke at 30 days in the TAVR group compared with the SAVR group in the pooled analysis, but noted that this difference was not significant when the RCT and observational subgroups were considered separately. Al-Abdouh et al. [22] and Kolte et al. [18] indicated that there was no statistically significant difference between TAVR and SAVR for the outcome of stroke in low-risk patients.

#### 3.2.3 Acute kidney injury (AKI)

Nine systematic reviews reported on the outcome of AKI for intermediate or low-risk patients. Villablanca et al. [20] reported no differences for TAVR and SAVR for AKI in their sensitivity analysis for intermediate-risk patients. The other eight systematic reviews reported that AKI was less common with TAVR than with SAVR for low and intermediate risk patients [[17], [18], [19],[23], [24], [25], [26], [27]].

#### 3.2.4 Bleeding complications

Nine studies reported on the outcome of Bleeding for intermediate or low-risk patients. Two systematic reviews indicated no differences between TAVR and SAVR [24,25], with seven systematic reviews highlighting a lower risk of bleeding with TAVR [[17], [18], [19], [20],^22^,^27^,^26^].

### 3.3 Conduction disturbance and arrhythmias

#### 3.3.1 Atrial fibrillation

Eight studies reported on the outcome of Atrial Fibrillation for intermediate or low-risk patients, all indicating a lower risk with TAVR [[17], [18], [19], [20],^22^,[25], [26], [27]].

#### 3.3.2 Permanent pacemaker

Eight studies reported on the outcome of Permanent Pacemaker Implantation for intermediate or low-risk patients. Seven studies indicated a higher risk of Permanent Pacemaker Implantation in the TAVR group [[18], [19], [20],^22^,[25], [26], [27]]. One study reported no difference in risk in their sensitivity analysis for intermediate risk patients [20].

#### 3.3.3 Myocardial infarction (MI)

Nine studies reported on the outcome of MI for intermediate or low-risk patients. Eight studies reported no difference in outcomes between TAVR and SAVR [[17], [18], [19], [20], [21],^22^,^25^,^26^]. One study indicated a lower risk for MI in low-to intermediate risk patients with TAVR [26].

### 3.4 Valvular function

#### 3.4.1 Aortic regurgitation

Two studies reported on the outcome of Aortic Regurgitation for intermediate or low-risk patients, with both indicating a lower risk in the SAVR group [20,21].

## 4 Discussion

To our knowledge, this is the first overview of reviews comparing TAVR and SAVR in low-to intermediate surgical risk patients with severe aortic valve stenosis, providing a comprehensive overview of systematic reviews both including RCTs and observational studies. Notably, 3 reviews include evidence from the most recent RCTs on low-risk patients, EVOLUT and PARTNER 3.

In the AMSTAR 2 assessment, we found that while most studies included appropriate tools for a risk-of-bias assessment, the reviews did not include an analysis of the potential effects of the risk of bias on the overall results. In addition, some systematic reviews did not discuss heterogeneity observed in the results in a satisfactory manner. For several studies, this led to an overall lower quality rating. This trend was also reported in other research areas using the AMSTAR 2 tool [28]. Overall, we could not identify any different trends in the results of studies with a “moderate” quality score versus those with a “low” quality rating.

Given the background of prevailing industry funding, especially in the context of RCTs studying the effects of TAVR versus SAVR efficacy for aortic stenosis patients, under-reporting the potential effects of bias in this area is problematic. Finally, the authors of two systematic reviews indicated that they had received industry funding without describing how they managed potential conflicts of interest [18,26]. After a thorough AMSTAR 2 check of methodological quality applied in the studies, we decided to include these reviews.

We found inconsistencies in the reporting of data across the systematic reviews, including variations in the numbers of patients included for the same studies, fragmented reporting on comorbidities and heterogeneous reporting on follow-up times.

The majority of studies reported no difference in all-cause mortality between TAVR and SAVR at 30 days and longer follow-up for intermediate and low-risk patients. Notably only Witberg [27] reported an increased mortality for low-risk patients with TAVR at 2 years. Witberg noted that this may be connected to a number of factors, such as the fact that the data in their analysis was based on studies performed at an earlier stage of the evolution of TAVR, and with the primary use of first generation devices. In addition, Witberg pointed to the modest sample size of their analysis on this point and to the fact that lower risk patients who are usually younger and with less comorbidities can recover better from complications that are more prevalent after SAVR. Chronic lung disease, advanced chronic kidney disease, frailty and some cardiovascular conditions have been linked in the literature to poor outcomes post-TAVR, independently of the procedural approach or the type of valve inserted [29]. It needs to be highlighted that the results for the long-term outcome of mortality were analysed at different follow up-times, ranging from 1 to 2 years. Both Fang [17] and Kolte [18] reported a significantly decreased risk of death from any cause at 1 year with TAVR for low-risk patients. Notably these reviews included the most recent RCTs on low-risk patients, EVOLUT and PARTNER 3. The outcome mortality at long-term follow-up, especially related to evidence for RCTs, was consistently rated as moderate or high for all studies with GRADE.

The importance of considering the latest evidence in valve durability over the long-term was highlighted in several systematic reviews, concluding that the findings are not generalizable to younger populations, due to a high mean age of patients included in the studies [18,22]. Valve durability remains a crucial factor especially for younger patients.

The outcome of stroke had the highest level of heterogeneity among the systematic reviews, with the majority of studies reporting no difference between TAVR and SAVR. These results were confirmed by a recent meta-analysis comparing the frequency of stroke after TAVR versus SAVR [30], where no difference in the incidence of stroke risk at 30 days and after one year was reported in a subgroup analysis of low-to-intermediate risk patients.

Reporting for the other outcomes stated in the results section was relatively consistent between the reviews. Notably, only two studies reported on the outcome of Aortic Regurgitation, both indicating a lower risk in the SAVR group.

Throughout the clinical trials, there were significant variations observed in the definition of surgical risk. For example, of the four trials included by Fang et al., covering low-risk patients, two defined low-risk of surgery to be <4% whereas the other two defined the value as <3% [17]. This point was also mentioned in Khan et al. [25].

### 4.1 Study limitations

One of the main strengths of this overview of systematic reviews of TAVR vs SAVR is the fact that it includes a comprehensive in-depth analysis of the available scientific literature. With respect to limitations, this overview does not evaluate results according to specific device types, different generations of the same devices or different types of valves; nor does it look at the different route of entry techniques used by these devices. These limitations can cause heterogeneity in our overall conclusions. With respect to the different generations of available TAVR devices, this can provoke a misleading perception as to how well the devices perform. For example, one of the RCT trials used the BE Edwards SAPIEN valve in their trial which is now no longer available [20]. As well as this, the RCTs in our overview use different generations of TAVR devices which could affect the overall results. However, Fang took this into consideration and performed a sensitivity analysis including only new generation valves, with no difference on the results of the main endpoints [17].

In addition, the rapid evolution of medical devices used for TAVR may lead to an underestimation of the treatment effect size of TAVR due to more emphasis put on earlier trial data. However, this is an inherent limitation of the methodology used in original articles. It is important to consider device improvements and evolution of sizing and practice in relation to TAVR since there could be a potential time-related dependency of the results. The relatively short duration of follow-up after intervention in some studies may leave uncertainties about critical outcomes.

A further limitation is the lack of clearly distinguished risk groups. As already mentioned in the discussion, this limitation is due to inconsistencies in the original reporting in the systematic reviews included. A total of three reviews focused on results for low-risk groups [18,22,27], while one reported sensitivity analysis results for the intermediate risk group [20] (Supplementary Information, Table 5). Seven reviews reported data for both low-to intermediate risk patients [17,19,21,[23], [24], [25], [26]], with three reviews distinguishing results for the two groups [17,24,26]. In clinical practice there is less focus on the use of EuroSCORE and STS-score to distinguish between risk groups. This is mainly attributed to the fact that both these risk scores omit important information that affects surgical risk. Additionally, the STS-score has been updated in 2018 and now provides much lower risk scores than previously. This limits the clinical applicability of using the risk scores.

## 5 Conclusions

The majority of studies included in our review were consistent in reporting no differences in mortality between TAVR and SAVR at short-and long term-follow up times. A decreased risk of mortality for low-risk patients with TAVR at 1 year follow-up was reported by two reviews that included the most recent RCTs on low-risk patients. Regarding the secondary endpoints of stroke and MI, the majority of studies presented similar results for TAVR and SAVR. Acute Kidney Injury, Bleeding Complications, Atrial Fibrillation were less frequent with TAVR, with lower risk of Permanent Pacemaker Implantation and Aortic Regurgitation with SAVR. Although data from longer follow-up times is needed to confirm these positive results, TAVR is emerging as a promising choice for low-to-intermediate risk patients.

In our overview we have summarized the available findings from numerous systematic reviews and highlighted inconsistencies in how the data are reported or presented, most notably the limited analysis on the effects of risk of bias and heterogeneity on trial results. AMSTAR2 guidance will need to be implemented more consistently to ensure high quality of future systematic reviews.

## Grant support

This work was supported by the 10.13039/501100000780European Commission 10.13039/501100000900Joint Research Centre (JRC) through the JRC Work Programme for 2019-2020, running under Horizon 2020, the current EU Framework Programme for research and innovation funding.

## Declaration of competing of interest

None.

## CRediT authorship contribution statement

**Roisin Mc Morrow:** Conceptualization, Methodology, Writing - original draft, Formal analysis. **Christine Kriza:** Conceptualization, Methodology, Writing - original draft, Writing - review & editing, Formal analysis. **Patricia Urbán:** Conceptualization, Methodology, Writing - original draft, Writing - review & editing, Visualization, Formal analysis. **Valeria Amenta:** Conceptualization, Methodology, Writing - original draft, Writing - review & editing, Formal analysis. **Juan Antonio Blasco Amaro:** Conceptualization, Writing - review & editing. **Dimitrios Panidis:** Conceptualization, Methodology, Writing - original draft, Writing - review & editing, Formal analysis. **Hubert Chassaigne:** Conceptualization, Methodology, Writing - original draft, Writing - review & editing, Supervision, Formal analysis. **Claudius Benedict Griesinger:** Conceptualization, Methodology, Writing - original draft, Supervision, Formal analysis.

## Appendix A Supplementary data

Supplementary material Image 1
