# Supplementary material for: Assessing the safety and efficacy of TAVR compared to SAVR in low-to-intermediate surgical risk patients with aortic valve stenosis: An overview of reviews
Source: Int J Cardiol. 2020 Sep 1;314:43–53. doi: 10.1016/j.ijcard.2020.04.022 (PMC7322533; doi:10.1016/j.ijcard.2020.04.022)
Supplement: Supplementary file 1 — Supplementary material [file mmc1.docx]

**Supplementary information**

**Table 1 Selection Criteria**

Inclusion criteria:

1. Study type: Systematic Reviews and Meta-analysis of Randomized Controlled Trials (RCTs) and/or Observational studies.
2. Intervention: Studies evaluating efficacy and/ or safety of TAVR. The routes of entry will not be analysed as well as the different types of TAVR devices i.e. Balloon expandable and self-expandable.
3. Comparator: SAVR (surgical aortic valve replacement)
4. Publication type: Peer-reviewed systematic reviews.
5. Patient characteristics:

- Low-to-Intermediate risk for surgery population
- with aortic valve stenosis (severe)
- with or without comorbidities
- Low-to-intermediate risk for death or complications associated with open-heart surgical aortic valve replacement (SAVR). Including the patient surgical risk assessment with STS / Euroscore parameters.

1. Clinical outcomes/endpoints:

Primary outcome: Mortality

Secondary outcomes: (based on VARC 2)

Included studies had to report at least on the primary outcome of mortality for TAVR compared to SAVR in low-to intermediate patient risk groups

1. Language limits: English, Italian, Spanish, French, German and Greek language.
2. No limitation has been applied to year of publication.

Exclusion criteria:

1. Study design: case studies or expert opinion, consensus groups and other non-comparative or non-randomized studies.
2. Studies including less frequently used delivery routes such as transcarotid, transcaval and antegrade aortic.
3. Guidelines, editorials, letter to the editor, Conference/congress communications and narrative reviews
4. Studies including only patients with contraindications for TAVR
5. Studies including only surrogate endpoints (e.g. imaging outcomes), non-disaggregated composite endpoints or haemodynamic parameters.
6. Technical or procedural descriptive studies
7. Not in human studies

**Table 2 Search Strategy and Search Strings**

When performing our literature search we used terms to restrict the title and abstract as well as Medical Subject Headings (MeSH) for MEDLINE and the "explosion term" (/exp) for EMBASE. Our main search keywords included "Aortic valve stenosis"; "Transcatheter aortic valve implantation"; "Transcatheter aortic valve replacement"; "Aortic valve prosthesis"; "Surgical aortic valve replacement"; "systematic review"; and "meta-analysis"

| PubMed |
| --- |
| ("Aortic valve stenosis"[MeSH Terms] OR Aortic Valve Stenosis[tiab] OR Aortic Valve Stenoses[tiab] OR Stenoses, Aortic Valve[tiab] OR Stenosis, Aortic Valve[tiab] OR Valve Stenoses, Aortic[tiab] OR Valve Stenosis, Aortic[tiab] OR Aortic Stenosis[tiab] OR Stenoses, Aortic[tiab] OR Stenosis, Aortic[tiab]) AND ("Transcatheter aortic valve replacement"[MeSH Terms] OR Transcatheter aortic valve replacement[tiab] OR Transcatheter aortic valve implantation[tiab] OR "Heart valve prosthesis"[MeSH Terms] OR Heart valve prosthesis[tiab] OR Heart Valve Prostheses[tiab] OR Prostheses, Heart Valve[tiab] OR Valve Prostheses, Heart[tiab] OR Valve Prosthesis, Heart[tiab] OR Prosthesis, Heart Valve[tiab] OR Cardiac Valve Prosthesis[tiab] OR Cardiac Valve Prostheses[tiab] OR Prostheses, Cardiac Valve[tiab] OR Prosthesis, Cardiac Valve[tiab] OR Valve Prostheses, Cardiac[tiab] OR Valve Prosthesis, Cardiac[tiab] OR "Heart valve prosthesis implantation"[MeSH Terms] OR heart valve prosthesis implantation[tiab] OR Implantation, Heart valve prosthesis[tiab] OR TAVI[tiab] OR Transcatheter aortic[tiab] OR Aortic valve prosthesis[tiab] OR valve prosthesis[tiab]) AND ("Aortic valve"[MeSH Terms] OR aortic valve[tiab] OR aortic valves[tiab] OR valve, aortic[tiab] OR valves, aortic[tiab] OR surgical aortic valve replacement[tiab] OR SAVR[tiab]) AND (analysis, meta[tiab] OR meta analysis[tiab] OR meta-analysis[tiab] OR metaanalysis[tiab] OR review, systematic[tiab] OR systematic review[tiab] OR review[tiab]) |
| EMBASE |
| ('aortic valve stenosis'/exp OR 'aorta valve stenosis':ti,ab OR 'aortic stenosis, valvular':ti,ab OR 'aortic valve stenosis':ti,ab OR 'aortic valvular stenosis':ti,ab OR 'stenosis, aorta valve':ti,ab OR 'valvular aortic stenosis':ti,ab) AND ('transcatheter aortic valve implantation'/exp OR 'tavi':ti,ab OR 'percutaneous aortic valve implantation':ti,ab OR 'percutaneous aortic valve replacement':ti,ab OR 'trans-apical aortic valve implantation':ti,ab OR 'trans-apical aortic valve replacement':ti,ab OR 'trans-arterial aortic valve implantation':ti,ab OR 'trans-arterial aortic valve replacement':ti,ab OR 'trans-catheter aortic valve implantation':ti,ab OR 'trans-catheter aortic valve replacement':ti,ab OR 'trans-cutaneous aortic valve implantation':ti,ab OR 'trans-cutaneous aortic valve replacement':ti,ab OR 'trans-femoral aortic valve implantation':ti,ab OR 'trans-femoral aortic valve replacement':ti,ab OR 'transapical aortic valve implantation':ti,ab OR 'transapical aortic valve replacement':ti,ab OR 'transarterial aortic valve implantation':ti,ab OR 'transarterial aortic valve replacement':ti,ab OR 'transcatheter aortic valve implantation':ti,ab OR 'transcatheter aortic valve replacement':ti,ab OR 'transcutaneous aortic valve implantation':ti,ab OR 'transcutaneous aortic valve replacement':ti,ab OR 'transfemoral aortic valve implantation':ti,ab OR 'transfemoral aortic valve replacement':ti,ab OR 'heart valve replacement'/exp OR 'cardiac valve replacement':ti,ab OR 'cardiac valvular replacement':ti,ab OR 'heart valve implantation':ti,ab OR 'heart valve prosthesis implantation':ti,ab OR 'heart valve replacement':ti,ab OR 'heart valvular replacement':ti,ab OR 'valve replacement':ti,ab OR 'valve replacement, heart':ti,ab) AND ('surgical aortic valve replacement'/exp OR 'surgical aortic valve replacement':ti,ab OR 'aortic valve replacement':ti,ab OR 'aortic valve'/exp OR 'aorta valve':ti,ab OR 'aortic cusp':ti,ab OR 'aortic heart valve':ti,ab OR 'aortic valve':ti,ab OR 'aortic valve annulies':ti,ab OR 'aortic valve anulus':ti,ab OR 'valva aortae':ti,ab OR 'valve, aorta':ti,ab) AND ('analysis, meta':ti,ab OR 'meta analysis':ti,ab OR 'meta-analysis':ti,ab OR 'metaanalysis':ti,ab OR 'review, systematic':ti,ab OR 'systematic review':ti,ab OR 'review':ti,ab) |

**Table 3: Study characteristics**

| Systematic Review | Study | Study Type | Total Population | TAVI Population | SAVR Population | EuroSCORE | | STS Score | | Mean Age | | n Female (%) | | Follow up (Months) | | |
| --- | --- | --- | --- | --- | --- | --- | --- | --- | --- | --- | --- | --- | --- | --- | --- | --- |
|  |  |  |  |  |  | TAVI | SAVR | TAVI | SAVR | TAVI | SAVR | TAVI | SAVR | Mean | Median | Max |
| Moderate quality review papers | | | | | | | | | | | | | | | | |
| Fang et al. 2019 | NOTION | RCT | 280 | 145 | 135 | / | | 3.0 | | 79.1 | | 53.2 | | 60 |  |  |
|  | Evolut R | RCT | 1468 | 734 | 734 | / | | 1.9 | | 73.9 | | 34.9 | | 24 |  |  |
|  | PARTNER 3 | RCT | 1000 | 496 | 454 | / | | 1.9 | | 73.3 | | 65.8 | | 12 |  |  |
|  | PARTNER 2A | RCT | 2032 | 1011 | 1021 | / | | 5.8 | | 81.6 | | 54.5 | | 24 |  |  |
|  | SURTAVI | RCT | 1746 | 864 | 796 | / | | 2.3 | | 79.6 | | 43.2 | | 24 |  |  |
|  | Mean | Total= 5 | Total= 6526 | Total= 3250 | Total= 3140 | / | | 3.0 | | 77.5 | | 48.52 | | 29 |  |  |
| Kolte et al. 2019 | NOTION | RCT | 280 | 145 | 135 | 1.9* | 2.0* | 2.9 | 3.2 | 79 | 79 | 67 (46.2) | 64 (47.4) | 12 |  |  |
|  | SURTAVI (STS < 3%) | RCT | 254 | 131 | 123 | / | / | 2.3 | 2.3 | 75 | 75 | 42 (32.1) | 39 (31.7) | 24 |  |  |
|  | PARTNER 3 | RCT | 950 | 496 | 454 | 1.5* | 1.5* | 1.9 | 1.9 | 73 | 74 | 161 (32.5) | 131 (28.9) | 12 |  |  |
|  | Evolut Low Risk | RCT | 1403 | 725 | 678 | / | / | 1.9 | 1.9 | 74 | 74 | 261 (36) | 229 (33.7) | 24 |  |  |
|  | Mean | Total =4 | Total = 2887 | Total= 1497 | Total= 1390 | 1.7 | 1.7 | 2.2 | 2.3 | 75 | 75 | 132 (36.7) | 116 (35.4) | 18 |  |  |
| Siemieniuk et al. 2016 | NOTION | RCT | 280 | 145 | 135 | / | | 3.0 | | 79.1 | | 131 (46.8) | | / | 24 | / |
|  | PARTNER 2A | RCT | 2032 | 1011 | 1021 | / | | 5.8 | | 81.6 | | 924 (45.5) | | / | 24 | / |
|  | STACCATO | RCT | 72 | 36 | 36 | / | | 3.3 | | 81 | | 49 (70.0) | | / | 3 | / |
|  | US Pivotal | RCT | 795 | 394 | 401 | / | | 7.4 | | 83.4 | | 372 (46.8) | | / | 36 | / |
|  | Mean | Total= 4 | Total= 3179 | Total= 1586 | Total= 1593 | / | | 4.9 | | 81 | | 369 (46) | | / | 2 | / |
| Villablanca et al. 2016 | CoreValve | RCT | 795 | 394 | 401 | 17.6 | 18.4 | 7.3 | 7.5 | 83 | 84 | / | | 36 | / | / |
|  | NOTION | RCT | 280 | 145 | 135 | 8.4 | 8.9 | 2.9 | 3.1 | 79 | 79 | / | | 24 | / | / |
|  | PARTNER | RCT | 699 | 348 | 351 | / | / | 11.8 | 11.7 | 84 | 85 | / | | 60 | / | / |
|  | PARTNER 2 | RCT | 2032 | 1011 | 1021 | / | / | 5.8 | 5.8 | 82 | 82 | / | | 24 | / | / |
|  | Mean of RCTs | Total= 4 | Total= 3806 | Total= 1898 | Total= 1908 | 13 | 13.7 | 7.0 | 7.0 | 82 | 83 | / | | 36 | / | / |
|  | Amonn | Obser. | 144 | 51 | 93 | 26.5 | 12.1 | 6.7 | 4.4 | 79 | 81 | / | | 15 | / | / |
|  | Brown | Obser. | 104 | 51 | 53 | / | / | 10.1 | 17.5 | 80 | 73 | / | | 24 | / | / |
|  | Conte | Obser. | 226 | 115 | 111 | 25.6 | 24.2 | 7.3 | 8 | 82 | 81 | / | | 12 | / | / |
|  | D'Onofrio | Obser. | 428 | 214 | 214 | 10.5 | 12.4 | / | / | 77 | 77 | / | | 12 | / | / |
|  | Dubois | Obser. | 108 | 73 | 35 | 25 | 18 | 7.3 | 6.6 | 82 | 83 | / | | 24 | / | / |
|  | Dvir | Obser. | 161 | 100 | 61 | 24.8 | 20.2 | 10.8 | 9.4 | 81 | 76 | / | | 12 | / | / |
|  | Falcone | Obser. | 153 | 51 | 102 | 31.6 | 18.8 | / | / | 79 | 71 | / | | 12 | / | / |
|  | Fox | Obser. | 26 | 8 | 18 | 9.5 | 8.8 | 9.2 | 18.8 | 70 | 67 | / | | 40 | / | / |
|  | Fusari | Obser. | 60 | 30 | 30 | 14.7 | 11.65 | 6.1 | 6.6 | 80 | 78 | / | | 21 | / | / |
|  | Grant | Obser. | 28092 | 3757 | 24335 | 21.9 | 7.9 | / | / | 81 | 68 | / | | 60 | / | / |
|  | Guarracino | Obser. | 60 | 30 | 30 | >20 (not used) | <20 (not used) | / | / | 82 | 82 | / | | 12 | / | / |
|  | Hannan | Obser. | 810 | 405 | 405 | / | / | / | / | / | / | / | | 12 | / | / |
|  | Henn | Obser. | 535 | 246 | 289 | / | / | 10.4 | 6.9 | 81 | 75 | / | | 12 | / | / |
|  | Holzhey | Obser. | 334 | 167 | 167 | 18.7 | 18.3 | / | / | 80 | 80 | / | | 22 | / | / |
|  | Jegaden | Obser. | 23 | 13 | 10 | 25 | 25 | / | / | 76 | 76 | / | | 13 | / | / |
|  | Johansson | Obser. | 80 | 40 | 40 | 24.5 | 22.7 | / | / | 82 | 81 | / | | 24 | / | / |
|  | Kamperidis | Obser. | 80 | 40 | 40 | 15.5 | 15.9 | / | / | 79 | 79 | / | | 18 | / | / |
|  | Kobrin | Obser. | 388 | 194 | 194 | / | / | / | / | 78 | 78 | / | | 12 | / | / |
|  | Latib | Obser. | 222 | 111 | 111 | 23.2 | 24.4 | / | / | 81 | 79 | / | | 12 | / | / |
|  | Mack | Obser. | 110 | 90 | 20 | / | / | 11.65 | 8.65 | 91 | 92 | / | | 12 | / | / |
|  | McCarthy | Obser. | 80 | 40 | 40 | / | / | 9.9 | 9.3 | 78 | 77 | / | | 24 | / | / |
|  | Miceli | Obser. | 74 | 37 | 37 | 22.4 | 10.2 | / | / | 80 | 76 | / | | 13 | / | / |
|  | Muneretto (2015) | Obser. | 612 | 204 | 408 | 19.5 | 19 | 8.2 | 8.1 | 80 | 80 | / | | 24 | / | / |
|  | Muneretto (2014) | Obser. | 163 | 55 | 108 | 20.4 | 18.6 | 6 | 5 | 82 | 80 | / | | 12 | / | / |
|  | Nĕmec | Obser. | 45 | 30 | 15 | 24.9 | 18.6 | / | / | 82 | 82 | / | | 12 | / | / |
|  | Nguyen | Obser. | 255 | 107 | 148 | / | / | 11.8 | 7.1 | 72 | 79 | / | | 24 | / | / |
|  | O'Sullivan | Obser. | 160 | 108 | 52 | 34.5 | 18.7 | 6.6 | 4.2 | 83 | 78 | / | | 12 | / | / |
|  | Onorati | Obser. | 56 | 28 | 28 | / | / | / | / | 78 | 77 | / | | 20 | / | / |
|  | Osnabrugge | Obser. | 84 | 42 | 42 | 12.9 | 12.5 | / | / | 79 | 79 | / | | 12 | / | / |
|  | Otten | Obser. | 53 | 39 | 14 | 15 | 9 | / | / | 81 | 81 | / | | 13 | / | / |
|  | Papadopoulos | Obser. | 80 | 40 | 40 | 24 | 19 | / | / | 81 | 80 | / | | 48 | / | / |
|  | Piazza | Obser. | 810 | 405 | 405 | 17.1 | 17.5 | / | / | 79 | 79 | / | | 12 | / | / |
|  | Pilgrim | Obser. | 364 | 257 | 107 | 22.4 | 9.8 | 5.1 | 3.4 | 83 | 80 | / | | 46 | / | / |
|  | Santarpino | Obser. | 204 | 102 | 102 | 18 | 17 | / | / | 79 | 80 | / | | 12 | / | / |
|  | Schymik | Obser. | 432 | 216 | 216 | 8.7 | 8.8 | / | / | 78 | 78 | / | | 12 | / | / |
|  | Silberman | Obser. | 252 | 64 | 188 | 18 | 14 | / | / | 84 | 80 | / | | 36 | / | / |
|  | Strauch | Obser. | 159 | 79 | 90 | 38 | 11 | 14 | 7 | 84 | 83 | / | | 12 | / | / |
|  | Tamburino | Obser. | 1300 | 650 | 650 | 20.4 | 18.6 | 6 | 5 | 81 | 80 | / | | 12 | / | / |
|  | Thourani | Obser. | 2051 | 1077 | 974 | / | / | 5.2 | 5.4 | 82 | 82 | / | | 12 | / | / |
|  | Tokarek | Obser. | 178 | 44 | 134 | 9.5 | 3.7 | / | / | 80 | 67 | / | | 24 | / | / |
|  | Walther | Obser. | 200 | 100 | 100 | 29 | 30 | 15.2 | / | 83 | 84 | / | | 12 | / | / |
|  | Wenaweser | Obser. | 364 | 257 | 107 | 24.7 | 12.5 | 6.4 | 4.8 | 82 | 80 | / | | 30 | / | / |
|  | Wendt | Obser. | 113 | 62 | 51 | 36.4 | 22.2 | 12.1 | 7.1 | 78 | 71 | / | | 60 | / | / |
|  | Yu | Obser. | 59 | 15 | 44 | 11 | 9.4 | / | / | 82 | 78 | / | | 14 | / | / |
|  | Zierer | Obser. | 51 | 21 | 30 | 38 | 35 | / | / | 85 | 82 | / | | 12 | / | / |
|  | Zweng | Obser. | 88 | 44 | 44 | / | / | / | / | 82 | 82 | / | | 24 | / | / |
|  | Mean of Observational | Total= 46 | Total= 40441 | Total= 9909 | Total= 30532 | 21.8 | 16.4 | 8.9 | 7.7 | 80 | 79 | / | | 20 | / | / |
| Wagner et al. 2019 | NOTION | RCT | 280 | 145 | 135 | 8.4 | 8.9 | 2.9 | 3.1 | 79.2 | 79 | 67 (46.2) | 64 (47.4) | / | / | 60 |
|  | PARTNER A | RCT | 699 | 348 | 351 | 29.3 | 29.2 | 11.8 | 11.7 | 83.6 | 84.5 | 147 (42.2) | 151  (43.3) | / | 16 | / |
|  | PARTNER 2A | RCT | 2032 | 1011 | 1021 | / | / | 5.8 | 5.8 | 81.5 | 81.7 | 463 (45.8) | 461 (45.2) | / | / | 24 |
|  | SURTAVI | RCT | 1746 | 879 | 867 | 11.9 | 11.6 | 4.4 | 4.5 | 79.9 | 79.8 | 371 (42.2) | 383 (44.2) | / | / | 24 |
|  | US CoreValve | RCT | 795 | 394 | 401 | 17.6 | 18.4 | 7.3 | 7.5 | 83.2 | 83.5 | 183 (46.4) | 189 (47.1) | 13.5 | / | / |
|  | Mean of RCT | Total= 5 | Total= 5552 | Total= 2777 | Total= 2775 | 16.8 | 17.0 | 6.4 | 6.5 | 82 | 82 | 246 (45) | 250 (45) | NA | NA | NA |
|  | D'Onofrio (2013) | Obser. | 286 | 143 | 143 | 20.2 | 18.3 | / | / | 77.6 | 73.5 | 90 (62.9) | 72 (50.3) | / | / | 12 |
|  | D'Onofrio (2016) | Obser. | 428 | 214 | 214 | 12.4 | 10.5 | / | / | 77.7 | 77.4 | 139 (65) | 138 (64.5) | / | / | 12 |
|  | Hannan | Obser. | 810 | 405 | 405 | / | / | / | / | / | / | 233 (57.5) | 219 (54.1) | / | 6.2 | / |
|  | Holzhey | Obser. | 334 | 167 | 167 | 18.7 | 18.3 | / | / | 79.8 | 80.5 | 108 (64.7) | 108 (64.7) | / | / | 12 |
|  | Kobrin | Obser. | 388 | 194 | 194 | / | / | / | / | 77.7 | 78 | 81 (41.8) | 85 (43.8) | / | / | 36 |
|  | Latib | Obser. | 222 | 111 | 111 | 23.2 | 24.4 | 4.57 | 4.6 | 80.5 | 79.4 | / | / | 37.2 | / | / |
|  | Muneretto (2015) * | Obser. | 612 | 204 | 408 | 19.5 | 19.05 | 8.2 | 8.1 | 80 | 79.5 | 91 (44.6) | 101 (49.7) | / | / | 24 |
|  | Piazza | Obser. | 810 | 405 | 405 | 17.1 | 17.5 | / | / | 79.9 | 79.4 | 230 (56.8) | 226  (55.8) | / | / | 1 |
|  | Repossini | Obser. | 284 | 142 | 142 | / | / | 7.2 | 6.7 | 76.2 | 76.4 | 57 (40.1) | 54 (38.1) | / | / | 12 |
|  | Schymik | Obser. | 432 | 216 | 216 | 8.7 | 8.8 | / | / | 78.3 | 78.2 | 116 (53.7) | 105 (48.6) | / | / | 12 |
|  | Tamburino | Obser. | 1300 | 650 | 650 | 9.5 | 10.2 | / | / | 80.5 | 80.3 | 383 (58.9) | 387 (59.5) | 20 | / | / |
|  | Walther | Obser. | 200 | 100 | 100 | 29 | 30 | 15.2 | / | 82.7 | 82.4 | 77 (77) | 70 (70) | / | / | 12 |
|  | Mean of Observational | Total= 12 | 6106 | 2951 | 3155 | 17.6 | 17.5 | 8.8 | 6.5 | 79 | 79 | 134 (54) | 130 (50) | NA | NA | NA |
| Low quality review papers | | | | | | | | | | | | | | | | |
| Al-Abdouh et al. 2019 | Evolut low-risk | RCT | 1468 | 734 | 734 | / | / | 1.9 | 1.9 | 74.0 | 73.8 | 266 (36.2) | 246 (33.5) | 12 | / | / |
|  | NOTION | RCT | 280 | 145 | 135 | / | / | 2.9 | 3.1 | 79.2 | 79 | 67 (46.2) | 64 (47.4) | 60 | / | / |
|  | PARTNER 3 | RCT | 950 | 496 | 454 | / | / | 1.9 | 1.9 | 73.3 | 73.6 | 161 (32.5) | 131 (28.85) | 12 | / | / |
|  | Mean of RCT | Total= 3 | Total=2698 | Total=1375 | Total=1323 | / | / | 2.2 | 2.3 | 76 | 76 | 165 (38) | 147 (37) | 28 | / | / |
| Ando et al. 2019 | CoreValve US study | RCT | 747 | 390 | 357 | / | / | 7.3 | 7.5 | 83.1 | 83.2 | (46.9) | (47.6) | / | / | / |
|  | Evolut low-risk | RCT | 1403 | 725 | 678 | / | / | 1.9 | 1.9 | 74.1 | 73.6 | (36.0) | (33.8) | / | / | / |
|  | NOTION | RCT | 280 | 145 | 135 | / | / | 2.9 | 3.1 | 79.2 | 79.0 | (46.2) | (47.4) | / | / | / |
|  | PARTNER 2 | RCT | 2033 | 1011 | 1022 | / | / | 5.8 | 5.8 | 81.5 | 81.7 | (45.8) | (45.2) | / | / | / |
|  | PARTNER 3 | RCT | 950 | 496 | 454 | / | / | 1.9 | 1.9 | 73.3 | 73.6 | (33.5) | (28.9) | / | / | / |
|  | STACCATO | RCT | 70 | 34 | 36 | / | / | 3.1 | 3.4 | 80 | 82 | (73.5) | (66.7) | / | / | / |
|  | SURTAVI | RCT | 1660 | 864 | 796 | / | / | 4.4 | 4.5 | 79.9 | 79.7 | (42.4) | (45) | / | / | / |
|  | Mean of RCT | Total= 7 | 7143 | 3665 | 3478 | / | / | 3.9 | 4.0 | 79 | 79 | (46) | (45) | / | / | / |
| Fu et al. 2019 | David (Medtronic US pivotal) | RCT | 795 | 394 | 401 | / | / | 7.3 | 7.5 | 83.2 | 83.5 | 183 (46) | 189 (47) | 36 | / | / |
|  | Hans (NOTION) | RCT | 280 | 145 | 135 | / | / | 2.9 | 3.1 | 79.2 | 79 | 67 (46) | 64 (47) | 24 | / | / |
|  | Martins (PARTNER II) | RCT | 2032 | 1011 | 1021 | / | / | 5.8 | 5.8 | 81.5 | 81.7 | 463 (46) | 461 (45) | 24 | / | / |
|  | Nielsen (STACCATO) | RCT | 70 | 34 | 36 | / | / | 3.1 | 3.4 | 80 | 82 | 25 (73.5) | 24 (67) | 3 | / | / |
|  | Reardon (SURTAVI) | RCT | 1746 | 879 | 867 | / | / | 4.4 | 4.5 | 79.9 | 79.8 | 371 (42) | 383 (44) | 24 | / | / |
|  | Mean of RCT | Total = 5 | Total=  4923 | Total= 2463 | Total= 2460 | / | / | 4.7 | 4.9 | 81 | 81 | 222(51) | 224 (50) | 22.2 | / | / |
|  | Alberto | Obser. | 284 | 142 | 142 | / | / | 6.7 | 7.2 | 76.4 | 76.2 | 54 (38) | 57 (40) | 1 | / | / |
|  | Azeem | Obser. | 222 | 111 | 111 | / | / | 4.6 | 4.6 | 80.5 | 79.4 | 49 (44) | 49 (44) | 12 | / | / |
|  | Christian | Obser. | 1610 | 805 | 805 | 6.8 | 4.2 | / | / | 77.5 | 77.5 | 486 (60) | 486 (60) | In-hospital | / | / |
|  | Corrado | Obser. | 1300 | 650 | 650 | 9.5 | 10.2 | / | / | 80.5 | 80.3 | 383 (59) | 387 (59) | 12 | / | / |
|  | Gerhard | Obser. | 432 | 216 | 216 | 8.7 | 8.8 | / | / | 78.3 | 78.2 | 116 (54) | 105 (49) | 36 | / | / |
|  | Javier | Obser. | 140 | 70 | 70 | / | / | 4.6 | 4.3 | 79 | 78 | 34 (49) | 36 (51) | 24 | / | / |
|  | Nicolo | Obser. | 510 | 255 | 255 | 17.3 | 17.6 | / | / | 80.6 | 79.7 | 156 (61) | 151 (59) | 12 | / | / |
|  | Nobuyuki | Obser. | 531 | 354 | 177 | / | / | 3.5 | 3.2 | 79.5 | 78 | 177 (50) | 94 (53) | 36 | / | / |
|  | Ruben | Obser. | 84 | 42 | 42 | 12.9 | 12.5 | / | / | 78.8 | 79.3 | 21 (50) | 20 (48) | 12 | / | / |
|  | Vinod | Obser. | 2021 | 1077 | 944 | / | / | 5.2 | 5.4 | 81.9 | 81.6 | 412 (38) | 425 (45) | 12 | / | / |
|  | Mean of Observational | Total = 10 | Total= 7134 | Total= 3722 | Total= 3412 | 11.0 | 10.7 | 4.9 | 4.9 | 79 | 79 | 189 (50) | 181 (51) | 17.4 | / | / |
| Khan et al. 2017 | PARTNER 2 | RCT | 2032 | 1011 | 1021 | / | / | 5.8 | 5.8 | 81.5 | 81.7 | (45.8) | (45.2) | / | / | / |
|  | NOTION | RCT | 280 | 145 | 135 | 8.4 | 8.9 | 2.9 | 3.1 | 79.2 | 79 | (46.2) | (47.4) | / | / | / |
|  | STACCATO | RCT | 70 | 34 | 36 | 9.4 | 10.3 | 3.1 | 3.4 | 80 | 82 | (73.5) | (66.7) | / | / | / |
|  | SURTAVI | RCT | 1660 | 864 | 796 | 11.9 | 11.6 | 4.4 | 4.5 | 79.9 | 79.7 | (42.4) | (45) | / | / | / |
|  | Mean of RCT | Total= 4 | Total= 4042 | Total= 2054 | Total= 1988 | 9.9 | 10.3 | 4.05 | 4.2 | 80 | 81 | (52) | (51) | / | / | / |
|  | Fraccaro | Obser. | 830 | 415 | 415 | 9.9 | 9.9 | NA | NA | 83.7 | 83.7 | (61.9) | (60) | / | / | / |
|  | Latib | Obser. | 222 | 111 | 111 | 23.2 | 24.4 | 4.5 | 4.6 | 80.5 | 79.4 | (55.9) | (55.9) | / | / | / |
|  | Muneretto | Obser. | 110 | 55 | 55 | 20.4 | 21.3 | 6 | 5.4 | 81 | 79 | (56.3) | (52.7) | / | / | / |
|  | Osnabrugge | Obser. | 84 | 42 | 42 | 12.9 | 12.5 | NA | NA | 78.8 | 79.3 | (50) | (47.6) | / | / | / |
|  | Piazza | Obser. | 510 | 255 | 255 | 17.5 | 17.1 | NA | NA | 79.9 | 79.4 | (56.8) | (55.8) | / | / | / |
|  | Schymik | Obser. | 432 | 216 | 216 | 8.7 | 8.8 | NA | NA | 78.3 | 78.2 | (53.7) | (48.6) | / | / | / |
|  | Tamburino | Obser. | 1300 | 650 | 650 | 9.5 | 10.2 | NA | NA | 80.5 | 80.3 | (58.9) | (59.5) | / | / | / |
|  | Thourani | Obser. | 2021 | 1077 | 944 | / | / | 5.2 | 5.4 | 81.9 | 81.6 | (38) | (45) | / | / | / |
|  | Mean of Observational | Total= 8 | Total= 5509 | Total= 2821 | Total= 2688 | 14.6 | 14.9 | 5.2 | 5.1 | 81 | 80 | (54) | (53) | / | / | / |
| Tam et al*.* 2017 | NOTION | RCT | 280 | 145 | 135 | 8.4 | 8.9 | 2.9 | 3.1 | 79.2 | 79 | (46.2) | (47.4) | 24 | / | / |
|  | PARTNER 2 | RCT | 2032 | 1011 | 1021 | / | / | 5.8 | 5.8 | 81.5 | 81.7 | (45.8) | (45.2) | 24 | / | / |
|  | STACCATO | RCT | 70 | 34 | 36 | 9.4 | 10.3 | 3.1 | 3.4 | 80 | 82 | (74.5) | (66.7) | 3 | / | / |
|  | SURTAVI | RCT | 1660 | 864 | 796 | 11.9 | 11.6 | 4.4 | 4.5 | 79.9 | 79.7 | (42.4) | (45) | 24 | / | / |
|  | Mean of RCT | Total=4 | Total=4042 | Total= 2054 | Total= 1988 | 9.9 | 10.3 | 4.05 | 4.2 | 80 | 81 | (52) | (51) | 18.75 | / | / |
|  | Castrodeza | Obser. | 140 | 70 | 70 | 9.4 | 9.3 | / | / | 79 | 78 | (48.6) | (51.4) | 12 | / | / |
|  | Fraccaro | Obser. | 830 | 415 | 415 | 9.9 | 9.9 | / | / | 83.7 | 83.7 | (61.9) | (60) | 36 | / | / |
|  | Frerker | Obser. | 1610 | 805 | 805 | 4.2 | 6.8 | / | / | 77.5 | 77.5 | (60.4) | (60.4) | Hospital | / | / |
|  | Kamperidis | Obser. | 80 | 40 | 40 | 15.5 | 15.9 | / | / | 79 | 79 | (0) | (0) | 18 | / | / |
|  | Latib | Obser. | 222 | 111 | 111 | 23.2 | 24.4 | 4.6 | 4.6 | 80.5 | 79.4 | (55.9) | (55.9) | 12 | / | / |
|  | Osnabrugge | Obser. | 84 | 42 | 42 | 12.9 | 12.5 | / | / | 78.8 | 79.3 | (50) | (47.6) | 12 | / | / |
|  | Piazza | Obser. | 510 | 255 | 255 | 17.3 | 17.6 | / | / | 80.6 | 79.7 | (61.2) | (59.2) | 12 | / | / |
|  | Repossini | Obser. | 284 | 142 | 142 | / | / | 7.2 | 6.7 | 76.2 | 76.4 | (40.1) | (38.1) | 3 | / | / |
|  | Schymik | Obser. | 432 | 216 | 216 | 8.7 | 8.8 | / | / | 78.3 | 78.2 | (53.7) | (48.6) | 36 | / | / |
|  | Mean of Observational | Total=9 | Total=4192 | Total= 2096 | Total= 2096 | 12.6 | 13.15 | NA | NA | 79 | 79 | (48) | (47) | 17.6 | / | / |
| Witberg et al. 2018 | Nielsen (STACCATO) | RCT | 70 | 34 | 36 | 9.4 | 10.3 | 3.1 | 3.4 | 80 | 82 | (73.5) | (66.7) | 3 | / | / |
|  | Thyregod (NOTION) | RCT | 280 | 145 | 135 | 8.4 | 8.9 | 2.9 | 3.1 | 79.2 | 79 | (46.2) | (47.4) | 12 | / | / |
|  | Mean of RCT | Total= 2 | Total= 350 | Total=179 | Total= 171 | 8.9 | 9.6 | 3 | 3.25 | 80 | 81 | (60) | (57) | 7.5 | NA | NA |
|  | Frerker | Obser. | 1610 | 805 | 805 | 6.8 | 4.2 | / | / | 77.5 | 77.5 | (60.4) | (60.4) | In hospital | / | / |
|  | Piazza | Obser. | 382 | 191 | 191 | / | / | <4 (not used) | <4 (not used) | / | / | / | / | 12 | / | / |
|  | Rosato | Obser. | 710 | 355 | 355 | 6.3 | 6.3 | / | / | 80.1 | 80.0 | (42.0) | (41.1) | 36 | / | / |
|  | Schymik | Obser. | 432 | 216 | 216 | 8.7 | 8.8 | / | / | 78.3 | 78.2 | (53.7) | (48.6) | 36 | / | / |
|  | Mean of Observational | Total= 4 | Total= 3134 | Total= 1567 | Total= 1567 | 7.3 | 6.4 | NA | NA | 79 | 79 | (52) | (50) | 28 | / | / |

* Kolte 2019 reported EUROSCORE II values

*Muneretto 2015 took average of two SAVR scores

**Table 4: Patient numbers in original studies**

Patient numbers from the original clinical papers were extracted in order to get a clear total number of the number of patients included in the 8 different RCT's and 64 different observational studies included in the 11 systematic reviews. This table has been made since there are sometimes variations in the systematic reviews on how the present the total population of included patients, sometimes they use the intention-to-treat number (usually) but other times they use the as-treated group.

| Clinical trials | As-treated | | Intention-to-treat | |
| --- | --- | --- | --- | --- |
|  | TAVI (n=) | SAVR (n=) | TAVI (n=) | SAVR (n=) |
| Randomised control trials | | | | |
| Evolut low risk | 725 | 678 | 734 | 734 |
| Medtronic CoreValve | 390 | 357 | 394 | 401 |
| NOTION | 141 | 134 | 145 | 135 |
| PARTNER | 348 | 351 | 348 | 351 |
| PARTNER II | 994 | 944 | 1011 | 1021 |
| PARTNER III | 496 | 454 | 503 | 497 |
| STACCATO | 34 | 36 | 36 | 36 |
| SURTAVI | 864 | 796 | 879 | 867 |
| Total | 3992 | 3750 | 4050 | 4042 |
| Observational studies | | | | |
| Alberto (2016) | 142 | 142 | NA | NA |
| Amonn (2013) | 51 | 53 | NA | NA |
| Azeem (2012) | 111 | 111 | NA | NA |
| Brown (2016) | 51 | 53 | NA | NA |
| Castrodeza (2016) | 70 | 70 | NA | NA |
| Christian (2017) | 805 | 805 | NA | NA |
| Conte (2016) | 115 | 111 | NA | NA |
| Corrado (2015) | 650 | 650 | NA | NA |
| D'Onofrio (2013) | 143 | 143 | NA | NA |
| D'Onofrio (2016) | 214 | 214 | NA | NA |
| Dubois (2013) | 73 | 35 | NA | NA |
| Dvir (2013) | 100 | 61 | NA | NA |
| Falcone (2014) | 51 | 102 | NA | NA |
| Fox (2013) | 8 | 18 | NA | NA |
| Fraccaro (2016) | 415 | 415 | NA | NA |
| Frerker (2017) | 805 | 805 | NA | NA |
| Fusari (2012) | 30 | 30 | NA | NA |
| Gerhard (2015) | 216 | 216 | NA | NA |
| Grant (2016) | 3757 | 24335 | NA | NA |
| Guarracino (2010) | 30 | 30 | NA | NA |
| Hannan (2016) | 405 | 405 | NA | NA |
| Henn (2016) | 246 | 289 | NA | NA |
| Holzhey (2012) | 167 | 167 | NA | NA |
| Javier (2016) | 70 | 70 | NA | NA |
| Jegaden (2012) | 13 | 10 | NA | NA |
| Johansson (2011) | 40 | 40 | NA | NA |
| Kamperidis (2015) | 40 | 40 | NA | NA |
| Kobrin (2015) | 194 | 194 | NA | NA |
| Latib (2012) | 111 | 111 | NA | NA |
| Mack (2015) | 90 | 20 | NA | NA |
| McCarthy (2014) | 40 | 40 | NA | NA |
| Miceli (2016) | 37 | 37 | NA | NA |
| Muneretto (2014) | 55 | 108 | NA | NA |
| Muneretto (2015) | 204 | 408 | NA | NA |
| Nĕmec (2012) | 30 | 15 | NA | NA |
| Nicolo (2013) | 255 | 255 | NA | NA |
| Nguyen (2014) | 107 | 148 | NA | NA |
| Nobuyuki (2018) | 354 | 177 | NA | NA |
| O'Sullivan (2015) | 108 | 52 | NA | NA |
| Onorati (2016) | 28 | 28 | NA | NA |
| Osnabrugge (2012) | 42 | 42 | NA | NA |
| Otten (2008) | 39 | 14 | NA | NA |
| Papadopoulos (2014) | 40 | 40 | NA | NA |
| Piazza (2013) | 405 | 405 | NA | NA |
| Pilgrim (2015) | 257 | 107 | NA | NA |
| Repossini (2017) | 142 | 142 | NA | NA |
| Rosato (2016) | 355 | 355 | NA | NA |
| Ruben (2012) | 42 | 42 | NA | NA |
| Santarpino (2015) | 102 | 102 | NA | NA |
| Schymik (2015) | 216 | 216 | NA | NA |
| Silberman (2013) | 64 | 188 | NA | NA |
| Strauch (2012) | 79 | 90 | NA | NA |
| Tamburino (2015) | 650 | 650 | NA | NA |
| Thourani (2016) | 1077 | 974 | NA | NA |
| Tokarek (2015) | 44 | 134 | NA | NA |
| Vinod (2016) | 1077 | 944 | NA | NA |
| Walther (2010) | 100 | 100 | NA | NA |
| Wenaweser (2011) | 257 | 107 | NA | NA |
| Wendt (2015) | 62 | 51 | NA | NA |
| Yu (2013) | 15 | 44 | NA | NA |
| Zierer (2009) | 21 | 30 | NA | NA |
| Zweng (2016) | 44 | 44 | NA | NA |
| Total | 15561 | 35834 | NA | NA |

**Table 5: AMSTAR2 assessment**

|  | **PICO** | **Protocol** | **Selection** | **Search** | **Screen2** | **Extract2** | **Exclude** | **Include** | **Bias** | **Funding** | **Meta** | **MetaB** | **RoB** | **Heter** | **Pub** | **CoI** |  |  |  |
| --- | --- | --- | --- | --- | --- | --- | --- | --- | --- | --- | --- | --- | --- | --- | --- | --- | --- | --- | --- |
|  | **Q1** | **Q2** | **Q3** | **Q4** | **Q5** | **Q6** | **Q7** | **Q8** | **Q9** | **Q10** | **Q11** | **Q12** | **Q13** | **Q14** | **Q15** | **Q16** | **Confidence** | **Included** | **Risk group** |
| Al-Abdouh | Y | N | Y | PY | Y | Y | N | Y | PY | N | Y | N | N | Y | N | Y | Low | Y | **Low risk** |
| Anantha-Narayanan | Y | N | Y | PY | Y | Y | N | PY | N | N | Y | N | N | Y | N | N | Critically Low | N | **Low risk** |
| Ando | Y | N | Y | PY | Y | N | N | PY | Y | N | Y | Y | Y | Y | Y | N | Low | Y | **Low/intermediate risk** |
| Arora | Y | N | N | PY | Y | N | N | PY | N | N | Y | N | N | N | Y | Y | Critically Low | N | **Intermediate risk** |
| Arora | Y | N | N | PY | Y | N | N | PY | N | N | N | N | N | Y | N | Y | Critically Low | N | **Low risk** |
| Burrage | Y | N | Y | PY | Y | Y | N | Y | N | N | Y | N | N | Y | Y | N | Critically Low | N | **Figure 6: mortality low-to intermediate risk** |
| Enezate | Y | N | N | N | Y | Y | N | PY | Y | N | PY | N | N | N | Y | Y | Critically Low | N | **Low/ intermediate risk** |
| Fang | Y | N | N | PY | Y | Y | Y | PY | Y | N | Y | Y | Y | Y | Y | Y | Moderate | Y | **Low/ intermediate risk** |
| Fu | Y | PY | Y | PY | Y | Y | N | Y | Y | N | Y | N | N | Y | N | Y | Low | Y | **Low/intermediate risk** |
| Garg | Y | N | N | PY | Y | Y | N | PY | N | N | Y | N | N | N | N | Y | Critically Low | N | **Low/intermediate risk** |
| Goel | Y | N | N | PY | Y | Y | N | PY | Y | N | PY | N | N | Y | N | Y | Critically Low | N | **Low risk** |
| Khan AR | Y | N | Y | PY | Y | Y | N | Y | PY | N | PY | N | N | Y | Y | Y | Critically Low | N | **Intermediate risk** |
| Khan SU | Y | N | Y | PY | Y | Y | N | PY | Y | N | Y | N | N | Y | Y | Y | Low | Y | **Low/intermediate risk** |
| Kheri | Y | N | N | N | N | N | N | PY | N | N | N | N | N | Y | N | N | Critically Low | N | **Low risk** |
| Kolte | Y | N | Y | PY | Y | Y | N | PY | Y | N | Y | Y | Y | N | Y | N | Moderate | Y | **Low risk** |
| Kondur | Y | N | N | PY | N | N | N | PY | N | N | PY | N | N | Y | Y | Y | Critically Low | N | **Low/intermediate risk** |
| Kundu | Y | N | Y | PY | N | Y | N | Y | N | N | Y | N | N | Y | Y | Y | Critically Low | N | **Low risk** |
| Lazkani | Y | N | N | PY | Y | Y | N | Y | PY | N | PY | N | N | Y | Y | Y | Critically Low | N | **Intermediate risk** |
| Rawasia | Y | N | N | PY | Y | Y | N | PY | Y/N | N | Y | Y | N | Y | Y | Y | Critically Low | N | **Low risk** |
| Sardar | Y | N | N | PY | Y | Y | N | Y | N | N | Y | N | N | Y | Y | Y | Critically Low | N | **Intermediate risk** |
| Siemieniuk | Y | PY | Y | PY | Y | Y | N | Y | Y | Y | Y | Y | Y | N | N | y | Moderate | Y | **Low/intermediate risk** |
| Singh | Y | N | Y | PY | Y | N | N | PY | Y | N | PY | N | N | N | Y | Y | Critically Low | N | **Intermediate risk** |
| Saleem | Y | N | Y | PY | Y | Y | N | PY | N | N | N | N | N | N | Y | Y | Critically Low | N | **Low risk** |
| Tam | Y | N | Y | PY | Y | N | N | Y | Y | N | Y | N | N | N | Y | N | Low | Y | **Low/intermediate risk** |
| Ueshima | Y | N | Y | PY | Y | Y | N | PY | Y | N | PY | Y | N | Y | Y | N | Critically Low | N | **Low/intermediate risk** |
| Villablanca | Y | PY | Y | PY | N | Y | N | PY | Y | N | Y | Y | Y | Y | Y | Y | Moderate | Y | **All risk, but with sensitivity analysis for intermediate risk** |
| Wagner | Y | N | Y | PY | Y | N | Y | Y | Y | Y | Y | Y | Y | Y | Y | Y | Moderate | Y | **All risk, but with subgroup analysis for low/ intermediate risk** |
| Wang | Y | N | Y | PY | Y | N | N | PY | Y | N | Y | N | N | N | Y | Y | Critically Low | N | **Low/intermediate risk** |
| Witberg | Y | PY | Y | PY | N | Y | N | PY | PY | N | Y | Y | N | N | Y | Y | Low | Y | **Low risk** |
| Witberg (2019) | Y | PY | Y | N | N | Y | N | PY | Y | N | Y | N | N | Y | N | Y | Critically Low | N | **Low risk** |
| Zhou | Y | N | N | PY | Y | N | N | PY | N | N | PY | N | N | N | Y | Y | Critically Low | N | **Low/intermediate risk** |

For full references of articles not included, refer to table 8

**Table 6: GRADE Assessment**

| **Ando et al. (only RCTs)** |  |  |
| --- | --- | --- |
| Mortality 30 days | ⨁⨁⨁⨁ HIGH |  |
| Mortality 1 year | ⨁⨁⨁⨁ HIGH |  |
| Stroke 30 days | ⨁⨁⨁◯ MODERATE | Imprecision |
| Stroke 1 year | ⨁⨁⨁⨁ HIGH |  |
|  |  |  |
| **Khan et al.** |  |  |
| Mortality Short Term RCT | ⨁⨁◯◯ LOW | Risk of Bias, Inconsistency |
| Mortality Short Term NRSI | ⨁◯◯◯ VERY LOW | Risk of Bias, Imprecision |
| Mortality Mid Term RCT | ⨁⨁⨁◯ MODERATE | Risk of Bias |
| Mortality Mid Term NRSI | ⨁◯◯◯ VERY LOW | Risk of Bias |
| Mortality Long Term RCT | ⨁⨁⨁◯ MODERATE | Risk of Bias |
| Stroke Short Term RCT | ⨁◯◯◯ VERY LOW | Risk of Bias, Imprecision, Inconsistency |
| Stroke Short Term NRSI | ⨁◯◯◯ VERY LOW | Risk of Bias |
| Stroke Mid Term RCT | ⨁⨁⨁◯ MODERATE | Risk of Bias |
| Stroke Mid Term NRSI | ⨁◯◯◯ VERY LOW | Risk of Bias, Imprecision |
| Stroke Long Term RCT | ⨁⨁⨁◯ MODERATE | Risk of Bias |
| Atrial Fibrillation RCTs | ⨁⨁⨁◯ MODERATE | Risk of Bias |
| Atrial Fibrillation NRSI | ⨁◯◯◯ VERY LOW | Risk of Bias, Imprecision, Inconsistency |
| AKI RCT | ⨁⨁⨁◯ MODERATE | Risk of Bias |
| AKI NRSI | ⨁◯◯◯ VERY LOW | Risk of Bias |
| Bleeding RCT | ⨁◯◯◯ VERY LOW | Risk of Bias, Imprecision, Inconsistency |
| Bleeding NRSI | ⨁◯◯◯ VERY LOW | Risk of Bias, Imprecision, Inconsistency |
| Permanent PM RCT | ⨁◯◯◯ VERY LOW | Risk of Bias, Imprecision, Inconsistency |
| Permanent PM NRSI | ⨁◯◯◯ VERY LOW | Risk of Bias, Inconsistency |
| MI Short Term RCT | ⨁⨁◯◯ LOW | Risk of Bias, Imprecision |
| MI Short Term NRSI | ⨁◯◯◯ VERY LOW | Risk of Bias, Imprecision |
| MI Mid Term RCT | ⨁⨁⨁◯ MODERATE | Risk of Bias |
| MI Mid Term NRSI | ⨁◯◯◯ VERY LOW | Risk of Bias, Imprecision, Inconsistency |
| MI Mid Term RCT | ⨁⨁⨁◯ MODERATE | Risk of Bias |
|  |  |  |
| **Tam et al.** |  |  |
| Mortality 30 days RCT | ⨁⨁⨁◯ MODERATE | Risk of Bias |
| Mortality 30 days NRSI | ⨁◯◯◯ VERY LOW | Risk of Bias |
| Late Mortality RCT | ⨁⨁⨁◯ MODERATE | Risk of Bias |
| Late Mortality NRSI | ⨁◯◯◯ VERY LOW | Risk of Bias |
| Stroke RCT | ⨁⨁⨁◯ MODERATE | Risk of Bias |
| Stroke NRSI | ⨁◯◯◯ VERY LOW | Risk of Bias, Imprecision |
| AKI RCT | ⨁⨁⨁◯ MODERATE | Risk of Bias |
| AKI NRSI | ⨁◯◯◯ VERY LOW | Risk of Bias, Imprecision |
| Atrial Fibrillation RCT | ⨁⨁⨁◯ MODERATE | Risk of Bias |
| Bleeding RCT | ⨁◯◯◯ VERY LOW | Risk of Bias, Imprecision, Inconsistency |
| Bleeding NRSI | ⨁◯◯◯ VERY LOW | Risk of Bias, Inconsistency |
| Permanent Pacemaker RCT | ⨁⨁◯◯ LOW | Risk of Bias, Inconsistency |
| Permanent Pacemaker NRSI | ⨁◯◯◯ VERY LOW | Risk of Bias |
| Myocardial Infarction RCT | ⨁⨁◯◯ LOW | Risk of Bias, Imprecision |
| Myocardial Infarction NRSI | ⨁◯◯◯ VERY LOW | Risk of Bias, Imprecision |
|  |  |  |
| **Wagner et al. (RCTs and NRSI)** |  |  |
| Mortality 30 days | ⨁⨁◯◯ LOW |  |
| Mortality 1 year | ⨁⨁◯◯ LOW |  |
|  |  |  |
| **Witberg et al.** |  |  |
| Mortality short term RCT | ⨁⨁⨁◯ MODERATE | Imprecision |
| Mortality short term NRSI | ⨁◯◯◯ VERY LOW | Imprecision |
| Mortality 2 years RCT | ⨁⨁◯◯ LOW | Imprecision, Inconsistency |
| Mortality 2 years NRSI | ⨁⨁◯◯ LOW |  |
| Acute kidney injury RCT | ⨁⨁⨁⨁ HIGH |  |
| Acute kidney injury NRSI | ⨁⨁◯◯ LOW |  |
| Bleeding RCT | ⨁⨁⨁⨁ HIGH |  |
| Bleeding NRSI | ⨁◯◯◯ VERY LOW | Inconsistency |
| Permanent pacemaker RCT | ⨁⨁⨁⨁ HIGH |  |
| Permanent pacemaker NRSI | ⨁⨁◯◯ LOW |  |
| Myocardial infarction RCT | ⨁⨁⨁◯ MODERATE | Imprecision |
| Myocardial infarction NRSI | ⨁◯◯◯ VERY LOW | Imprecision |
|  |  |  |
| **Fu et al.** |  |  |
| Mortality short term RCT | ⨁⨁⨁◯ MODERATE | Risk of Bias |
| Mortality short term NRSI | ⨁◯◯◯ VERY LOW | Risk of Bias, Imprecision |
| Mortality one year RCT | ⨁⨁⨁◯ MODERATE | Risk of Bias |
| Mortality one year NRSI | ⨁◯◯◯ VERY LOW | Risk of Bias |
| Mortality 2 years RCT | ⨁⨁⨁◯ MODERATE | Risk of Bias |
| Mortality 2 years NRSI | ⨁◯◯◯ VERY LOW | Risk of Bias |
| Mortality 3 years RCT | ⨁⨁◯◯ LOW | Risk of Bias, Inconsistency |
| Mortality 3 years NRSI | ⨁◯◯◯ VERY LOW | Risk of Bias |
| Acute kidney injury RCT | ⨁⨁⨁◯ MODERATE | Risk of Bias |
| Acute kidney injury NRSI | ⨁◯◯◯ VERY LOW | Risk of Bias |
| Bleeding RCT | ⨁◯◯◯ VERY LOW | Risk of Bias, Imprecision, Inconsistency |
| Bleeding NRSI | ⨁◯◯◯ VERY LOW | Risk of Bias, Imprecision |
| Permanent pacemaker RCT | ⨁⨁◯◯ LOW | Risk of Bias, Inconsistency |
| Permanent pacemaker NRSI | ⨁◯◯◯ VERY LOW | Risk of Bias, Inconsistency |
| Myocardial infarction RCT | ⨁⨁⨁◯ MODERATE | Risk of Bias |
| Myocardial infarction NRSI | ⨁◯◯◯ VERY LOW | Risk of Bias, Imprecision, Inconsistency |
| Stroke (30 days) RCT | ⨁⨁⨁◯ MODERATE | Risk of Bias |
| Stroke (30 days) NRSI | ⨁◯◯◯ VERY LOW | Risk of Bias, Imprecision |
| Stroke (1 year) RCT | ⨁⨁⨁◯ MODERATE | Risk of Bias |
| Stroke (1 year) NRSI | ⨁◯◯◯ VERY LOW | Risk of Bias, Imprecision, Inconsistency |
| Atrial Fibrillation RCT | ⨁⨁⨁◯ MODERATE | Risk of Bias |
| Atrial Fibrillation NRSI | ⨁◯◯◯ VERY LOW | Risk of Bias, Inconsistency |
|  |  |  |
| **Al-Abdouh et al. (only RCTs)** |  |  |
| Mortality | ⨁⨁⨁⨁ HIGH |  |
| Acute kidney injury | ⨁⨁⨁⨁ HIGH |  |
| Bleeding | ⨁⨁⨁⨁ HIGH |  |
| Permanent pacemaker | ⨁⨁⨁◯ MODERATE | Inconsistency |
| Myocardial infarction | ⨁⨁⨁◯ MODERATE | Imprecision |
| Stroke | ⨁⨁⨁◯ MODERATE | Imprecision |
| Atrial Fibrillation | ⨁⨁⨁◯ MODERATE | Inconsistency |
|  |  |  |
| **Villablanca et al. (RCTs and NRSI)** |  |  |
| Mortality (long term) | ⨁⨁◯◯ LOW |  |
| Mortality 30 days | ⨁◯◯◯ VERY LOW | Imprecision |
| Acute kidney injury | ⨁◯◯◯ VERY LOW | Impresion, Inconsistency |
| Bleeding | ⨁◯◯◯ VERY LOW | Inconsistency |
| Permanent pacemaker | ⨁◯◯◯ VERY LOW | Impresion, Inconsistency |
| Myocardial infarction | ⨁◯◯◯ VERY LOW | Imprecision |
| Stroke | ⨁⨁◯◯ LOW |  |
| Atrial Fibrillation | ⨁◯◯◯ VERY LOW | Inconsistency |
|  |  |  |
| **Siemieniuk et al. (RCTs), self-reported** |  |  |
| Mortality 2 years | ⨁⨁⨁◯ MODERATE |  |
| Stroke | ⨁⨁⨁◯ MODERATE |  |
| AKI | ⨁⨁◯◯ LOW |  |
| Atrial Fibrillation | ⨁⨁⨁⨁ HIGH |  |
| Bleeding | ⨁⨁⨁⨁ HIGH |  |
| Permanent Pacemaker | ⨁⨁⨁⨁ HIGH |  |
| Myocardial Infarction | ⨁⨁⨁◯ MODERATE |  |
|  |  |  |
| **Fang et al. (RCTs, self-reported)** |  |  |
| Mortality or stroke 12 months low risk | ⨁⨁⨁⨁ HIGH |  |
| Mortality or stroke 12 months intermediate risk | ⨁⨁⨁⨁ HIGH |  |
| All-cause mortality 12 months low risk | ⨁⨁⨁◯ MODERATE |  |
| All-cause mortality 12 months intermediate risk | ⨁⨁⨁⨁ HIGH |  |
| Disabling stroke 12 months low risk | ⨁⨁⨁◯ MODERATE |  |
| Disabling stroke 12 months intermediate risk | ⨁⨁⨁⨁ HIGH |  |
| Atrial fibrillation 12 months low risk | ⨁⨁◯◯ LOW |  |
| Atrial fibrillation 12 months intermediate risk | ⨁⨁◯◯ LOW |  |
| Acute kidney injury 12 months low risk | ⨁⨁⨁◯ MODERATE |  |
| Acute kidney injury 12 months intermediate risk | ⨁⨁⨁◯ MODERATE |  |
|  |  |  |
| **Kolte et al. (RCTs)** |  |  |
| Mortality all cause 1 year | ⨁⨁⨁⨁ HIGH |  |
| Cardiovascular death 1 year | ⨁⨁⨁⨁ HIGH |  |
| Acute kidney injury | ⨁⨁⨁⨁ HIGH |  |
| Bleeding | ⨁⨁⨁⨁ HIGH |  |
| Permanent pacemaker | ⨁⨁⨁◯ MODERATE | Inconsistency |
| Myocardial infarction 1 year | ⨁⨁⨁◯ MODERATE | Imprecision |
| Stroke (1 year) | ⨁⨁⨁◯ MODERATE | Imprecision |
| Atrial Fibrillation | ⨁⨁⨁⨁ HIGH |  |

**Table 7: PRISMA checklist**

| **Section/topic** | | **#** | | **Checklist item** | | **Reported on page #** |
| --- | --- | --- | --- | --- | --- | --- |
| **TITLE** | | | | | |  |
| Title | | 1 | | Identify the report as a systematic review, meta-analysis, or both. | | 2 |
| **ABSTRACT** | | | | | |  |
| Structured summary | | 2 | | Provide a structured summary including, as applicable: background; objectives; data sources; study eligibility criteria, participants, and interventions; study appraisal and synthesis methods; results; limitations; conclusions and implications of key findings; systematic review registration number. | | Abstract |
| **INTRODUCTION** | | | | | |  |
| Rationale | | 3 | | Describe the rationale for the review in the context of what is already known. | | 1-3 |
| Objectives | | 4 | | Provide an explicit statement of questions being addressed with reference to participants, interventions, comparisons, outcomes, and study design (PICOS). | | 4 |
| **METHODS** | | | | | |  |
| Protocol and registration | | 5 | | Indicate if a review protocol exists, if and where it can be accessed (e.g., Web address), and, if available, provide registration information including registration number. | | 4, tbc |
| Eligibility criteria | | 6 | | Specify study characteristics (e.g., PICOS, length of follow-up) and report characteristics (e.g., years considered, language, publication status) used as criteria for eligibility, giving rationale. | | 4-5 |
| Information sources | | 7 | | Describe all information sources (e.g., databases with dates of coverage, contact with study authors to identify additional studies) in the search and date last searched. | | 5 |
| Search | | 8 | | Present full electronic search strategy for at least one database, including any limits used, such that it could be repeated. | | Tab 2, supp |
| Study selection | | 9 | | State the process for selecting studies (i.e., screening, eligibility, included in systematic review, and, if applicable, included in the meta-analysis). | | 4-5 |
| Data collection process | | 10 | | Describe method of data extraction from reports (e.g., piloted forms, independently, in duplicate) and any processes for obtaining and confirming data from investigators. | | 4-5 |
| Data items | | 11 | | List and define all variables for which data were sought (e.g., PICOS, funding sources) and any assumptions and simplifications made. | | 4-5 |
| Risk of bias in individual studies | | 12 | | Describe methods used for assessing risk of bias of individual studies (including specification of whether this was done at the study or outcome level), and how this information is to be used in any data synthesis. | | 5-7 |
| Summary measures | | 13 | | State the principal summary measures (e.g., risk ratio, difference in means). | | No quantitative analysis |
| Synthesis of results | | 14 | | Describe the methods of handling data and combining results of studies, if done, including measures of consistency (e.g., I^2^) for each meta-analysis. | | No quantitative analysis |
| Section/topic | | # | | Checklist item | | Reported on page # |
| Risk of bias across studies | | 15 | | Specify any assessment of risk of bias that may affect the cumulative evidence (e.g., publication bias, selective reporting within studies). | | Tab 5, supp |
| Additional analyses | | 16 | | Describe methods of additional analyses (e.g., sensitivity or subgroup analyses, meta-regression), if done, indicating which were pre-specified. | | Not applicable |
| **RESULTS** | | | | |  | |
| Study selection | 17 | | Give numbers of studies screened, assessed for eligibility, and included in the review, with reasons for exclusions at each stage, ideally with a flow diagram. | | Fig 1 | |
| Study characteristics | 18 | | For each study, present characteristics for which data were extracted (e.g., study size, PICOS, follow-up period) and provide the citations. | | Tab 3, supp | |
| Risk of bias within studies | 19 | | Present data on risk of bias of each study and, if available, any outcome level assessment (see item 12). | | Tab 5, supp | |
| Results of individual studies | 20 | | For all outcomes considered (benefits or harms), present, for each study: (a) simple summary data for each intervention group (b) effect estimates and confidence intervals, ideally with a forest plot. | | Tab 2 | |
| Synthesis of results | 21 | | Present results of each meta-analysis done, including confidence intervals and measures of consistency. | | Not applicable | |
| Risk of bias across studies | 22 | | Present results of any assessment of risk of bias across studies (see Item 15). | | Tab 5, tab 6 supp | |
| Additional analysis | 23 | | Give results of additional analyses, if done (e.g., sensitivity or subgroup analyses, meta-regression [see Item 16]). | | Not applicable | |
| **DISCUSSION** | | | | |  | |
| Summary of evidence | 24 | | Summarize the main findings including the strength of evidence for each main outcome; consider their relevance to key groups (e.g., healthcare providers, users, and policy makers). | | 14-17 | |
| Limitations | 25 | | Discuss limitations at study and outcome level (e.g., risk of bias), and at review-level (e.g., incomplete retrieval of identified research, reporting bias). | | 17-18 | |
| Conclusions | 26 | | Provide a general interpretation of the results in the context of other evidence, and implications for future research. | | 18-19 | |
| **FUNDING** | | | | |  | |
| Funding | 27 | | Describe sources of funding for the systematic review and other support (e.g., supply of data); role of funders for the systematic review. | | Title page | |

*From:*  Moher D, Liberati A, Tetzlaff J, Altman DG, The PRISMA Group (2009). Preferred Reporting Items for Systematic Reviews and Meta-Analyses: The PRISMA Statement. PLoS Med 6(7): e1000097. doi:10.1371/journal.pmed1000097

For more information, visit: **www.prisma-statement.org**.

**Table 8: Details of records excluded**

We excluded in total 1260 papers for the following reasons:

**Study Design (n=1000):**

Aalaei-Andabili, S. H. and Bavry, A. A. (2019) ‘Left Ventricular Diastolic Dysfunction and Transcatheter Aortic Valve Replacement Outcomes: A Review’, *Cardiology and Therapy*, 8(1), pp. 21–28. doi: 10.1007/s40119-019-0134-5.

Abbasi, D. *et al.* (2018) ‘Diagnosis and Management of Rare Case of Mesenteric Hematoma Rupture after Transcatheter Aortic Valve Replacement (TAVR): A Case Report and Review of the Literature.’, *Case reports in vascular medicine*. United States, p. 6273538. doi: 10.1155/2018/6273538.

Abdelghani, M. *et al.* (2016) ‘Adjudicating paravalvular leaks of transcatheter aortic valves: a critical appraisal.’, *European heart journal*. England, 37(34), pp. 2627–2644. doi: 10.1093/eurheartj/ehw115.

Abdel-Wahab, M., El-Mawardy, M. and Richardt, G. (2015) ‘Update on transcatheter aortic valve replacement.’, *Trends in cardiovascular medicine*. United States, 25(2), pp. 154–161. doi: 10.1016/j.tcm.2014.10.001.

Abdul-Jawad Altisent, O. *et al.* (2016) ‘Embolic Protection Devices During TAVI: Current Evidence and Uncertainties’, *Revista espanola de cardiologia (English ed.)*. Spain, 69(10), pp. 962–972. doi: 10.1016/j.rec.2016.04.056.

Adams, H. S. L. *et al.* (2019) ‘Contemporary review of severe aortic stenosis’, *Internal Medicine Journal*, 49(3), pp. 297–305. doi: 10.1111/imj.14071.

Addis, A. and Davoli, M. (2016) ‘[Governance of innovative cardiovascular devices: when evidence is not enough].’, *Recenti progressi in medicina*. Italy, pp. 7–9. doi: 10.1701/2132.23096.

Afshar, Ata Hassani; Pourafkari, Leili; Nader, Nader D. (2016) ‘Periprocedural considerations of transcatheter aortic valve implantation for anesthesiologists’, *Journal of Cardiovascular and Thoracic Research*. N.D. Nader, Department of Anesthesiology, University at Buffalo, Buffalo, NY, United States, 8(2), pp. 49–55. doi: 10.15171/jcvtr.2016.10.

Agarwal, S. *et al.* (2015) ‘Transcatheter aortic valve replacement: Current perspectives and future implications’, *Heart*. S.R. Kapadia, Sones Cardiac Catheterization Laboratories, Department of Cardiovascular Medicine, J2-3, Heart and Vascular Institute, Cleveland Clinic, 9500 Euclid Avenue, Cleveland, OH, United States, England, 101(3), pp. 169–177. doi: 10.1136/heartjnl-2014-306254.

Agarwal, S. *et al.* (2013) ‘Comparison of multicenter registries and randomized control trials for transcatheter aortic valve replacement (TAVR).’, *Indian heart journal*. India, 65(4), pp. 400–411. doi: 10.1016/j.ihj.2013.06.007.

A., K., J.J., T. and V.T., N. (2018) ‘Management of Patients With Aortic Valve Stenosis’, *Mayo Clinic Proceedings*. V.T. Nkomo, Mayo Clinic College of Medicine and Science, 200 First St SW, Rochester, MN, United States, 93(4), pp. 488–508. doi: 10.1016/j.mayocp.2018.01.020.

G., A. *et al.* (2006) ‘Neonatal Surgical Aortic Commissurotomy: Predictors of Outcome and Long-Term Results’, *Annals of Thoracic Surgery*. G. Agnoletti, Pediatric Cardiology, Necker Enfants Malades, Paris, France, 82(5), pp. 1585–1592. doi: 10.1016/j.athoracsur.2006.05.049.

Ahmad, M. *et al.* (2019) ‘Conscious Sedation Versus General Anesthesia in Transcatheter Aortic Valve Replacement: A Cost and Outcome Analysis’, *Cureus*. doi: 10.7759/cureus.4812.

T., A. *et al.* (2015) ‘Challenges of valve surgeries in post-renal transplant patients’, *Annals of Cardiac Anaesthesia*. T. Ahmad, #156, 3rd Cross, Shirdi Sai Nagar, Banshankari, Bengaluru, Karnataka, India, 18(4), pp. 603–605. doi: 10.4103/0971-9784.166485.

Akins, C. W.; Travis, B. & Yoganathan, A. P. (2008) ‘Energy loss for evaluating heart valve performance’, *Journal of Thoracic and Cardiovascular Surgery*. C.W. Akins, Division of Cardiac Surgery, Massachusetts General Hospital, Boston, Mass, United States, 136(4), pp. 820–833. doi: 10.1016/j.jtcvs.2007.12.059.

Aksoy, O. *et al.* (2013) ‘Options for managing severe aortic stenosis: A case-based review’, *Cleveland Clinic Journal of Medicine*. V. Menon, Coronary Intensive Care Unit, Department of Cardiovascular Medicine, J1-5, Cleveland Clinic, 9500 Euclid Avenue, Cleveland, OH 44195, United States, United States, 80(4), pp. 243–252. doi: 10.3949/ccjm.80a.12069.

Aksoy, O. *et al.* (2016) ‘Aortic annular rupture during TAVR: Mini review.’, *Cardiovascular revascularization medicine : including molecular interventions*. United States, 17(3), pp. 199–201. doi: 10.1016/j.carrev.2016.03.005.

Aksu, T., Yuksel, U. C. and Tuzcu, M. (2010) ‘[Percutaneous treatment of aortic stenosis].’, *Turk Kardiyoloji Dernegi arsivi : Turk Kardiyoloji Derneginin yayin organidir*. Turkey, 38(4), pp. 290–301.

Al-Bawardy, B.; Gorospe, E. & Kraichely, R. (2013) ‘Heyde’s syndrome in the setting of bioprosthetic aortic valve mismatch: Case report and literature review’, *American Journal of Gastroenterology*. B. Al-Bawardy, Mayo Clinic, Rochester, MN, United States, 108, p. S296. doi: 10.1038/ajg.2013.267.

Alfadhli, J. *et al.* (2018) ‘Updates on transcatheter aortic valve replacement: Techniques, complications, outcome, and prognosis.’, *Journal of the Saudi Heart Association*. Netherlands, 30(4), pp. 340–348. doi: 10.1016/j.jsha.2018.07.002.

Ali, A. *et al.* (2011) ‘Enhanced left ventricular mass regression after aortic valve replacement in patients with aortic stenosis is associated with improved long-term survival.’, *The Journal of thoracic and cardiovascular surgery*. United States, 142(2), pp. 285–291. doi: 10.1016/j.jtcvs.2010.08.084.

Ali, N., Patel, P. A. and Lindsay, S. J. (2018) ‘Recent developments and controversies in transcatheter aortic valve implantation.’, *European journal of heart failure*. England, 20(4), pp. 642–650. doi: 10.1002/ejhf.1141.

Allende, R. *et al.* (2015) ‘The transradial approach during transcatheter structural heart disease interventions: a review.’, *European journal of clinical investigation*. England, 45(2), pp. 215–225. doi: 10.1111/eci.12398.

Alshuwaykh, O. and Krier, M. J. (2018) ‘A Case of Heyde Syndrome with Resolution of Gastrointestinal Bleeding Two Weeks After Aortic Valve Replacement.’, *The American journal of case reports*. United States, 19, pp. 924–926. doi: 10.12659/AJCR.911298.

Alsoufi, B. *et al.* (2007) ‘Management options in neonates and infants with critical left ventricular outflow tract obstruction’, *European Journal of Cardio-thoracic Surgery*. B. Alsoufi, The Cardiac Center, The Hospital for Sick Children, the University of Toronto, Toronto, Ont., Canada, Germany, 31(6), pp. 1013–1021. doi: 10.1016/j.ejcts.2007.03.015.

Alva, C., Gomez, F. D. and Yanez Gutierrez, L. (2006) ‘[Congenital aortic valve stenosis. Current treatment].’, *Archivos de cardiologia de Mexico*. Mexico, 76 Suppl 4, pp. S152-7.

Alvarez-Fernandez, B. *et al.* (2017) ‘[Non-cardiac aspects of aortic stenosis in the elderly: A review].’, *Revista espanola de geriatria y gerontologia*. Spain, 52(2), pp. 87–92. doi: 10.1016/j.regg.2016.06.001.

Amat-Santos, I. J., Cortes, C. and Varela-Falcon, L. H. (2017) ‘Delayed left anterior mitral leaflet perforation and infective endocarditis after transapical aortic valve implantation-Case report and systematic review.’, *Catheterization and cardiovascular interventions : official journal of the Society for Cardiac Angiography & Interventions*. United States, 89(5), pp. 951–954. doi: 10.1002/ccd.26410.

Amzaev, S. A. *et al.* (2018) ‘[Endovascular Treatment of Critical capital A, Cyrillicsmall o, Cyrillicrtic Stenosis: Current State of the Problem and Perspectives].’, *Kardiologiia*. Russia (Federation), 58(7), pp. 95–100.

Andersen, H. R. (2009) ‘History of percutaneous aortic valve prosthesis.’, *Herz*. Germany, 34(5), pp. 343–346. doi: 10.1007/s00059-009-3251-4.

Ankeney, J. L. & Tzeng, T. S. (1983) ‘Surgical therapy for congenital aortic valvular stenosis. A 23 year experience’, *Journal of Thoracic and Cardiovascular Surgery*. Div. Cardiothorac. Surg., Univ. Hosp., Cleveland, OH 44106, 85(1), pp. 41–48. Available at: <http://www.embase.com/search/results?subaction=viewrecord&from=export&id=L13094458>.

Annabi, M.-S. *et al.* (2018) ‘Workup and Management of Patients With Paradoxical Low-Flow, Low-Gradient Aortic Stenosis.’, *Current treatment options in cardiovascular medicine*. United States, 20(6), p. 49. doi: 10.1007/s11936-018-0642-y.

Antonini-Canterin, F. *et al.* (2006) ‘Is there a role of statins in the prevention of aortic biological prostheses degeneration.’, *Cardiovascular ultrasound*. England, 4, p. 26. doi: 10.1186/1476-7120-4-26.

Araque, J. C. *et al.* (2016) ‘The Role of Balloon Aortic Valvuloplasty in Patients With Aortic Valve Stenosis and Society of Thoracic Surgeons Risk of 15% or Higher’, *Annals of Thoracic Surgery*. K.L. Greason, Division of Cardiovascular Surgery, Joseph 5-200, Mayo Clinic, 200 First St SW, Rochester, MN, United States, Netherlands, 101(2), pp. 592–598. doi: 10.1016/j.athoracsur.2015.07.030.

Arias, E. A. *et al.* (2019) ‘TAVI for pure native aortic regurgitation: Are we there yet?’, *Interventional Cardiology Review*. E.A. Arias, National Institute of Cardiology Ignacio Chávez, Juan Badiano 1, Col Sección XVI, Tlalpan, Mexico City, Mexico, England, 14(1), pp. 26–30. doi: 10.15420/icr.2018.37.1.

Armijo, G., Nombela-Franco, L. and Tirado-Conte, G. (2018) ‘Cerebrovascular Events After Transcatheter Aortic Valve Implantation.’, *Frontiers in cardiovascular medicine*. Switzerland, 5, p. 104. doi: 10.3389/fcvm.2018.00104.

Arnold, S. V. (2019) ‘Calculating Risk for Poor Outcomes After Transcatheter Aortic Valve Replacement’, *Journal of Clinical Outcomes Management.*, 26(3), pp. 125–129.

Aronow, W. S. (2013) ‘A review of the pathophysiology, diagnosis, and treatment of aortic valve stenosis in elderly patients.’, *Hospital practice (1995)*. England, 41(4), pp. 66–77. doi: 10.3810/hp.2013.10.1082.

Aronow, W. S. (1995) ‘Usefulness of M-mode, 2-dimensional, and Doppler echocardiography in the diagnosis, prognosis, and management of valvular aortic stenosis, aortic regurgitation, and mitral annular calcium in older patients’, *Journal of the American Geriatrics Society*. W.S. Aronow, Hebrew Hospital Home, 801 Co-op City Blvd., Bronx, NY 10475, United States, 43(3), pp. 295–300. doi: 10.1111/j.1532-5415.1995.tb07342.x.

Arora, S. *et al.* (2016) ‘Staged high-risk percutaneous coronary intervention: With impella support after on-pump transcatheter aortic valve replacement’, *Texas Heart Institute Journal*. S. Arora, Division of Cardiology, University of North Carolina at Chapel Hil, 160 Dental Cir., Chapel Hill, NC, United States, United States, 43(5), pp. 423–427. doi: 10.14503/THIJ-16-5770.

Arora, S. *et al.* (2016) ‘Transcatheter versus surgical aortic valve replacement in intermediate risk patients: a meta-analysis.’, *Cardiovascular diagnosis and therapy*. China (Republic : 1949- ), 6(3), pp. 241–249. doi: 10.21037/cdt.2016.03.04.

Arora, S. *et al.* (2016) ‘TAVR in Intermediate-Risk Patients: A Review of the PARTNER 2 Trial and its Future Implications’, *The Journal of heart valve disease*, 25(6), pp. 653–656. Available at: http://www.embase.com/search/results?subaction=viewrecord&from=export&id=L621989852.

Arsalan, M. and Walther, T. (2016) ‘Durability of prostheses for transcatheter aortic valve implantation.’, *Nature reviews. Cardiology*. England, 13(6), pp. 360–367. doi: 10.1038/nrcardio.2016.43.

Asil, S. *et al.* (2016) ‘Transcatheter aortic valve implantation in patients with a mitral prosthesis; single center experience and review of literature.’, *International journal of cardiology*. Netherlands, 221, pp. 390–395. doi: 10.1016/j.ijcard.2016.07.051.

Auffret, V. *et al.* (2017) ‘Conduction Disturbances After Transcatheter Aortic Valve Replacement: Current Status and Future Perspectives.’, *Circulation*. United States, 136(11), pp. 1049–1069. doi: 10.1161/CIRCULATIONAHA.117.028352.

Awais, M. and Bach, D. S. (2009) ‘Exercise stress testing in asymptomatic severe aortic stenosis.’, *The Journal of heart valve disease*. England, 18(3), pp. 235–238.

Aydin, E. *et al.* (2014) ‘How to approach aortic valve disease in the elderly: a 25-year retrospective study.’, *Cardiovascular journal of Africa*. South Africa, 25(5), pp. 244–248. doi: 10.5830/CVJA-2014-051.

Azpitarte, J. *et al.* (2000) ‘[Guidelines of the Spanish Society of Cardiology on valve heart disease].’, *Revista espanola de cardiologia*. Spain, 53(9), pp. 1209–1278.

Bach, D. S. & Bach, David S. (2005) ‘Subvalvular left ventricular outflow obstruction for patients undergoing aortic valve replacement for aortic stenosis: Echocardiographic recognition and identification of patients at risk’, *Journal of the American Society of Echocardiography*. D.S. Bach, Department of Medicine, Division of Cardiology, University of Michigan, 1500 E Medical Center Dr, Ann Arbor, MI 48109, United States, United States, 18(11), pp. 1155–1162. doi: 10.1016/j.echo.2005.08.005.

Backer, C. L. (2005) ‘Repairing the aortic valve is best for children’, *Cardiology in the Young*. C.L. Backer, Division of Cardiovascular-Thoracic Surgery, Children’s Memorial Hospital, Northwestern University Feinberg School of Medicine, 2300 Children’s Plaza, Chicago, IL 60614, United States, 15(SUPPL. 1), pp. 122–124. doi: 10.1017/S1047951105001149.

Badano, L. P. *et al.* (2006) ‘Clinical and hemodynamic implications of supra-annular implant of biological aortic valves.’, *Journal of cardiovascular medicine (Hagerstown, Md.)*. United States, 7(7), pp. 524–532. doi: 10.2459/01.JCM.0000234771.96324.28.

Bagur, R. *et al.* (2018) ‘Association of comorbid burden with clinical outcomes after transcatheter aortic valve implantation’, *Heart*. T. Kinnaird, Heart Team, London Health Sciences Centre, London, ON, Canada, England, 104(24), pp. 2058–2066. doi: 10.1136/heartjnl-2018-313356.

Bahlmann, E., Kuck, K.-H. and Nienaber, C. A. (2011) ‘[Asymptomatic aortic stenosis].’, *Deutsche medizinische Wochenschrift (1946)*. Germany, 136(25–26), pp. 1371–1376. doi: 10.1055/s-0031-1280558.

Baker, Regina Y. *et al.* (2012) ‘Breast implants and minimally invasive cardiac surgery: a case series.’, *Annals of plastic surgery*. R.Y. Baker, Division of Plastic and Reconstructive Surgery, Department of Surgery, Keck School of Medicine, University of Southern California, Los Angeles, CA 90033, USA., United States, 69(1), pp. 10–13. doi: 10.1097/SAP.0b013e318221b54b.

Balanescu, S. M. *et al.* (2019) ‘The Onco-cardiologist Dilemma: to Implant, to Defer, or to Avoid Transcatheter Aortic Valve Replacement in Cancer Patients with Aortic Stenosis?’, *Current cardiology reports*. United States, 21(8), p. 83. doi: 10.1007/s11886-019-1166-0.

Balmforth, D. *et al.* (2017) ‘Is ministernotomy superior to right anterior minithoracotomy in minimally invasive aortic valve replacement?’, *Interactive cardiovascular and thoracic surgery*. England, 25(5), pp. 818–821. doi: 10.1093/icvts/ivx241.

Banach, M.*et al.* (2008) ‘Obesity and postoperative atrial fibrillation. Is there no connection?. Comment on: Wanahita et al. “Atrial fibrillation and obesity-results of a meta-analysis”’, *American Heart Journal*. M. Banach, Medical University of Lodz, Lodz, Poland, 156(1), p. e5. doi: 10.1016/j.ahj.2008.03.028.

Banovic, M. *et al.* (2017) ‘Percutaneous Treatment of Aortic Valve Disease: Contemporary Overview and Future Trends.’, *Current pharmaceutical design*. United Arab Emirates, 23(31), pp. 4687–4695. doi: 10.2174/1381612822666161018162022.

Baquero, G. A. *et al.* (2019) ‘Hybrid off-pump coronary artery bypass grafting surgery and transaortic transcatheter aortic valve replacement: Literature review of a feasible bailout for patients with complex coronary anatomy and poor femoral access.’, *Journal of cardiac surgery*. United States, 34(7), pp. 591–597. doi: 10.1111/jocs.14082.

Baradarian, S. *et al.* (2002) ‘Left ventricular outflow tract obstruction associated with chronic ventricular assist device support.’, *ASAIO journal (American Society for Artificial Internal Organs : 1992)*. United States, 48(6), pp. 665–667.

Barbanti, M. *et al.* (2013) ‘Aortic stenosis and mitral regurgitation: implications for transcatheter valve treatment.’, *EuroIntervention : journal of EuroPCR in collaboration with the Working Group on  Interventional Cardiology of the European Society of Cardiology*. France, 9 Suppl, pp. S69-71. doi: 10.4244/EIJV9SSA13.

Barbanti, M., Gargiulo, G. and Tamburino, C. (2016) ‘Renal dysfunction and transcatheter aortic valve implantation outcomes.’, *Expert review of cardiovascular therapy*. England, 14(12), pp. 1315–1323. doi: 10.1080/14779072.2016.1234377.

Barbanti, M. *et al.* (2018) ‘Optimization and simplification of transcatheter aortic valve implantation therapy.’, *Expert review of cardiovascular therapy*. England, 16(4), pp. 287–296. doi: 10.1080/14779072.2018.1449644.

Barbanti, M. *et al.* (2017) ‘Pathophysiology, incidence and predictors of conduction disturbances during Transcatheter Aortic Valve Implantation’, *Expert Review of Medical Devices*. M. Barbanti, Division of Cardiology, Ferrarotto Hospital, University of Catania, via Citelli 6, , Italy, 14(2), pp. 135–147. doi: 10.1080/17434440.2017.1282819.

Barbanti, M. and Tamburino, C. (2016) ‘Late degeneration of transcatheter aortic valves: pathogenesis and management.’, *EuroIntervention : journal of EuroPCR in collaboration with the Working Group on Interventional Cardiology of the European Society of Cardiology*. France, 12(Y), pp. Y33-6. doi: 10.4244/EIJV12SYA8.

Barbanti, M. *et al.* (2017) ‘Transcatheter aortic valve implantation in 2017: state of the art.’, *EuroIntervention : journal of EuroPCR in collaboration with the Working Group on Interventional Cardiology of the European Society of Cardiology*. France, 13(AA), pp. AA11–AA21. doi: 10.4244/EIJ-D-17-00567.

Barker, C. M. (2016) ‘Clinical trials in transcatheter aortic valve replacement.’, *Current opinion in cardiology*. United States, 31(4), pp. 343–348. doi: 10.1097/HCO.0000000000000301.

Barker, C. M. (2012) ‘Aortic Valve Surgery and Transcatheter Aortic Valve Replacement for the Very Old: Improved Interventional Therapeutic Options for Aortic Stenosis for Elderly’, *Current Cardiovascular Risk Reports*. C. M. Barker, Department of Cardiology, Methodist DeBakey Heart and Vascular Center, Smith Tower, 6550 Fannin Street, Suite 1901, Houston, TX, 77030, United States, 6(5), pp. 420–424. doi: 10.1007/s12170-012-0264-y.

Barnhorst, D. A. *et al.* (1975) ‘Long-term follow-up of isolated replacement of the aortic or mitral valve with the Starr-Edwards prosthesis.’, *The American journal of cardiology*. United States, 35(2), pp. 228–233. doi: 10.1016/0002-9149(75)90006-5.

Barnhorst, D. A. *et al.* (1975) ‘Isolated replacement of the aortic valve with the Starr-Edwards prosthesis. A 9 year review.’, *The Journal of thoracic and cardiovascular surgery*. D.A. Barnhorst, United States, 70(1), pp. 113–118. Available at: <http://www.embase.com/search/results?subaction=viewrecord&from=export&id=L5549165>.

C.R., B. *et al.* (2013) ‘Current management of left ventricular assist device erosion’, *Journal of Cardiac Surgery*. C.R. Bartoli, Division of Cardiovascular Surgery, University of Pennsylvania Medical Center, Philadelphia, PA 19104, United States, 28(6), pp. 776–782. doi: 10.1111/jocs.12207.

Bashir, M. *et al.* (2017) ‘Aortic Valve Replacement: Are We Spoiled for Choice?’, *Seminars in Thoracic and Cardiovascular Surgery*. M. Bashir, Barts Heart Centre, St Bartholomew’s Hospital, London, United Kingdom, United States, 29(3), pp. 265–272. doi: 10.1053/j.semtcvs.2017.08.003.

Basra, S. and Szerlip, M. (2017) ‘Transcatheter Aortic Valve Replacement and MitraClip to Reverse Heart Failure.’, *Interventional cardiology clinics*. Netherlands, 6(3), pp. 373–386. doi: 10.1016/j.iccl.2017.03.007.

Bavishi, C. *et al.* (2018) ‘Transcatheter aortic valve replacement in patients with severe aortic stenosis and heart failure.’, *Heart failure reviews*. United States, 23(6), pp. 821–829. doi: 10.1007/s10741-018-9726-8.

Bax, J. J. *et al.* (2019) ‘Transcatheter Aortic Valve Replacement: Role of Multimodality Imaging in Common and Complex Clinical Scenarios.’, *JACC. Cardiovascular imaging*. United States. doi: 10.1016/j.jcmg.2018.10.037.

Bechtel, J. F. and Sievers, H.-H. (2005) ‘[Aortic valve operation in young adults].’, *Deutsche medizinische Wochenschrift (1946)*. Germany, 130(12), pp. 669–674. doi: 10.1055/s-2005-865078.

Beckmann, E. *et al.* (2010) ‘Insights into the use of biomarkers in calcific aortic valve disease’, *Journal of Heart Valve Disease*. G. Ferrari, University of Pennsylvania School of Medicine, Harrison Department of Surgical Research, 500 S. Ridgeway Avenue, Glenolden, PA 19036, United States, England, 19(4), pp. 441–452. Available at: <http://www.embase.com/search/results?subaction=viewrecord&from=export&id=L359430889>.

Bedeir, K. *et al.* (2016) ‘Sutureless Aortic Valves: Combining the Best or the Worst?’, *Seminars in thoracic and cardiovascular surgery*. United States, 28(2), pp. 341–352. doi: 10.1053/j.semtcvs.2016.05.010.

Behan, M. W. *et al.* (2010) ‘Balloon aortic valvuloplasty: Review of the evidence and current indications’, *Interventional Cardiology*. M. W. Behan, Department of Cardiology, East Wing, St Thomas’ Hospital, Westminster Bridge Road, London SE1 7EH, United Kingdom, 2(5), pp. 673–679. doi: 10.2217/ica.10.64.

Ben-Ami, M. *et al.* (1990) ‘Aortic valve replacement during pregnancy. A case report and review of the literature.’, *Acta obstetricia et gynecologica Scandinavica*. United States, 69(7–8), pp. 651–653.

Ben-Dor, I. *et al.* (2010) ‘Utility of radiologic review for noncardiac findings on multislice computed tomography in patients with severe aortic stenosis evaluated for transcatheter aortic valve implantation.’, *The American journal of cardiology*. United States, 105(10), pp. 1461–1464. doi: 10.1016/j.amjcard.2009.12.071.

Ben-Dor, I. *et al.* (2010) ‘Patient selection--risk assessment and anatomical selection criteria for patients undergoing transfemoral aortic valve implantation.’, *Cardiovascular revascularization medicine : including molecular interventions*. United States, 11(2), pp. 124–136. doi: 10.1016/j.carrev.2009.03.005.

Benfari, G. *et al.* (2014) ‘Is TAVI superior to surgery in high-risk patients? Insight into the concept of individual risk assessment’, *Minerva Medica*. F. Onorati, Division of Cardiac Surgery, University of Verona, Medical School, Ospedale Civile Maggiore, piazzale Stefani 1, Verona, Italy, Italy, 105(6), pp. 487–495. Available at: http://www.embase.com/search/results?subaction=viewrecord&from=export&id=L603548612.

Ben-Shoshan, J., Amit, S. and Finkelstein, A. (2016) ‘Transcatheter Aortic Valve Implantation Infective Endocarditis: Current Data and  Implications on Prophylaxis and Management.’, *Current pharmaceutical design*. United Arab Emirates, 22(13), pp. 1959–1964.

Beran, E., Marzouk, J. F. K. and Dimitri, W. R. (2008) ‘Bilateral phrenic nerve palsy following aortic valve surgery.’, *Journal of cardiac surgery*. United States, 23(6), pp. 691–692. doi: 10.1111/j.1540-8191.2008.00753.x.

D., B. and Berdajs, D. (2013) ‘Aortic root rupture: Implications of catheter-guided aortic valve replacement’, *Current Opinion in Cardiology*. D. Berdajs, Department of Cardiovascular Surgery, Centre Hospitalier Universitaire Vaudois (CHUV), Rue du Bugnon 46, Lausanne 1011, Switzerland, United States, 28(6), pp. 632–638. doi: 10.1097/HCO.0b013e3283655bb5.

Berger, D. (2018) ‘Evolution of a TAVR program’, *Critical Care Nursing Quarterly*. D. Berger, Allegheny Health Network, Allegheny General Hospital, 320 East North Ave, Pittsburgh, PA, United States, 41(4), pp. 360–368. doi: 10.1097/CNQ.0000000000000221.

Bergler-Klein, J., Gyongyosi, M. and Maurer, G. (2014) ‘The role of biomarkers in valvular heart disease: focus on natriuretic peptides.’, *The Canadian journal of cardiology*. England, 30(9), pp. 1027–1034. doi: 10.1016/j.cjca.2014.07.014.

Bergmann, T., Sengupta, P. P. and Narula, J. (2017) ‘Is TAVR Ready for the Global Aging Population?’, *Global heart*. England, 12(4), pp. 291–299. doi: 10.1016/j.gheart.2017.02.002.

Berkowitz, J. M., Lansman, S. and Fyfe, B. (1998) ‘Coronary artery mycotic aneurysm following endocarditis of a composite aortic graft--a case report and literature review.’, *Angiology*. United States, 49(2), pp. 145–150. doi: 10.1177/000331979804900207.

Bertella, E. *et al.* (2013) ‘[Role of multimodality imaging in patients undergoing transcatheter aortic valve  implantation].’, *Recenti progressi in medicina*. Italy, 104(9), pp. 498–505. doi: 10.1701/1331.14739.

Bhatia, A. *et al.* (2015) ‘Adults With Left-Sided Pressure Loading Lesions’, *Current Treatment Options in Cardiovascular Medicine*. J.T. Kuvin, Division of Cardiology, CardioVascular Center, Tufts Medical Center, 800 Washington Street, Box 315, Boston, MA, United States, 17(12). doi: 10.1007/s11936-015-0416-8.

Bianco, V. *et al.* (2019) ‘Open Surgical Access for Transfemoral TAVR Should Not Be a Contraindication for Conscious Sedation.’, *Journal of cardiothoracic and vascular anesthesia*. United States, 33(1), pp. 39–44. doi: 10.1053/j.jvca.2018.05.036.

Bierhoff, M. *et al.* (2011) ‘Listeria peritonitis in patients on peritoneal dialysis: Two cases and a review of the literature’, *Netherlands Journal of Medicine*. M. Bierhoff, Department of Internal Medicine, Kennemer Gasthuis, Haarlem, Netherlands, 69(10), pp. 461–464. Available at: <http://www.embase.com/search/results?subaction=viewrecord&from=export&id=L362888096>.

Bigelow, J. C. *et al.* (1968) ‘Multiple valve replacement. Review of five years’ experience.’, *Circulation*. J.C. Bigelow, 38(4), pp. 656–663. doi: 10.1161/01.CIR.38.4.656.

Bilen, E. *et al.* (2014) ‘Optimal timing of valve replacement in asymptomatic severe aortic stenosis’, *The Journal of heart valve disease*. England, 23(5), pp. 524–533. Available at: <http://www.embase.com/search/results?subaction=viewrecord&from=export&id=L603857215>.

Bilkhu, R., Jahangiri, M. and Otto, C. M. (2019) ‘Patient-prosthesis mismatch following aortic valve replacement’, *Heart*, 105(Suppl 2), pp. s28–s33. doi: 10.1136/heartjnl-2018-313515.

Billings, F. T. *et al.* (2009) ‘Transcatheter aortic valve implantation: Anesthetic considerations’, *Anesthesia and Analgesia*. F. T. Billings, Division of Critical Care, Department of Anesthesiology, 1211 21st Avenue South, Nashville, TN 37212, United States, 108(5), pp. 1453–1462. doi: 10.1213/ane.0b013e31819b07ce.

Binter, C. *et al.* (2013) ‘Assessment of energy loss in aortic stenosis using Bayesian multipoint phase-contrast MRI’, *Journal of Cardiovascular Magnetic Resonance*. C. Binter, Institute for Biomedical Engineering, University and ETH Zurich, Zürich, Switzerland, 15, pp. 242–243. Available at: <http://www.embase.com/search/results?subaction=viewrecord&from=export&id=L70994164>.

Bitran, D. and Silberman, S. (2018) ‘The role of SU-AVR in tomorrow’s practice of AVR.’, *Minerva cardioangiologica*. Italy, 66(2), pp. 198–204. doi: 10.23736/S0026-4725.17.04552-2.

Blakeslee-Carter, J. *et al.* (2018) ‘A Novel Iliac Morphology Score Predicts Procedural Mortality and Major Vascular Complications in Transfemoral Aortic Valve Replacement.’, *Annals of vascular surgery*. Netherlands, 46, pp. 208–217. doi: 10.1016/j.avsg.2017.06.137.

Bleakley, C., Eskandari, M. and Monaghan, M. (2017) ‘3D transoesophageal echocardiography in the TAVI sizing arena: should we do it and how do we do it?’, *Echo research and practice*. England, 4(1), pp. R21–R32. doi: 10.1530/ERP-16-0041.

Bleakley, C. and Monaghan, M. J. (2018) ‘The Pivotal Role of Imaging in TAVR Procedures.’, *Current cardiology reports*. United States, 20(2), p. 9. doi: 10.1007/s11886-018-0949-z.

Blehm, A., Schurr, P. and Lichtenberg, A. (2013) ‘Role of primary bacterial contamination of a pulmonary homograft for Ross operation: report of a case and review of the literature.’, *The Thoracic and cardiovascular surgeon*. Germany, 61(6), pp. 541–542. doi: 10.1055/s-0031-1293601.

Bloch, A. *et al.* (1987) ‘[Ultrasonic diagnosis of aortic valve lesions in adults].’, *Ultraschall in der Medizin (Stuttgart, Germany : 1980)*. Germany, 8(5), pp. 206–211. doi: 10.1055/s-2007-1011696.

Bloomfield, G. S. *et al.* (2012) ‘A practical guide to multimodality imaging of transcatheter aortic valve replacement.’, *JACC. Cardiovascular imaging*. United States, 5(4), pp. 441–455. doi: 10.1016/j.jcmg.2011.12.013.

Bob-Manuel, T. *et al.* (2018) ‘A review of racial disparities in transcatheter aortic valve replacement (TAVR): accessibility, referrals and implantation.’, *Annals of translational medicine*. China, 6(1), p. 10. doi: 10.21037/atm.2017.10.17.

Bohula May, E. A. and Faxon, D. (2013) ‘Transcatheter aortic valve replacement: history and current status.’, *Trends in cardiovascular medicine*. United States, 23(5), pp. 172–178. doi: 10.1016/j.tcm.2012.11.001.

Boix-Garibo, R.; Uzzaman, M. M. & Bapat, V. (2015) ‘Review of Minimally Invasive Aortic Valve Surgery’, *Interventional Cardiology Review*. V. Bapat, Department of Cardiac Surgery, St Thomas’ Hospital, Westminster Bridge Road, London, United Kingdom, 10(3), pp. 144–148. doi: 10.15420/ICR.2015.10.03.144.

Bokhari, S. S. I., O’Neill, W. W. and Cohen, M. G. (2011) ‘A tale of two pressures: a case of pseudo-prosthetic mitral valve stenosis.’, *Catheterization and cardiovascular interventions : official journal of the Society for Cardiac Angiography & Interventions*. United States, 78(7), pp. 1022–1028. doi: 10.1002/ccd.22973.

van der Boon, R. M. A. *et al.* (2014) ‘Clinical implications of conduction abnormalities and arrhythmias after transcatheter aortic valve implantation.’, *Current cardiology reports*. United States, 16(1), p. 429. doi: 10.1007/s11886-013-0429-4.

van der Boon, R. M. *et al.* (2012) ‘New conduction abnormalities after TAVI--frequency and causes.’, *Nature reviews. Cardiology*. England, 9(8), pp. 454–463. doi: 10.1038/nrcardio.2012.58.

Borowski, A. *et al.* (2007) ‘Surgery for severe aortic stenosis with low transvalvular gradient and poor left ventricular function -- a single centre experience and review of the literature.’, *Journal of cardiothoracic surgery*. A. Borowski, Department of Thoracic and Cardiovascular Surgery, University of Düsseldorf, Germany., England, 2, p. 9. doi: 10.1186/1749-8090-2-9.

Bortolotti, U., Celiento, M. and Milano, A. D. (2014) ‘Enlargement of the aortic annulus during aortic valve replacement: a review.’, *The Journal of heart valve disease*. England, 23(1), pp. 31–39.

Boudewijns, M. *et al.* (2003) ‘Rothia dentocariosa, endocarditis and mycotic aneurysms: Case report and review of the literature’, *Clinical Microbiology and Infection*. M. Boudewijns, Clinical Laboratory, Virga Jesse Hospital, Stadsomvaart 11, B-3500 Hasselt, Belgium, 9(3), pp. 222–229. doi: 10.1046/j.1469-0691.2003.00503.x.

Bouhout, I. *et al.* (2019) ‘Aortic Valve Interventions in Pediatric Patients’, *Seminars in Thoracic and Cardiovascular Surgery*. N. Poirier, Division of Cardiac Surgery, University of Montreal, CHU-ME Ste-Justine, 3175 Côte Ste-Catherine, Montréal, Québec, Canada, 31(2), pp. 277–287. doi: 10.1053/j.semtcvs.2018.10.009.

Bourantas, C. V *et al.* (2012) ‘Transcatheter aortic valve implantation: new developments and upcoming clinical trials.’, *EuroIntervention : journal of EuroPCR in collaboration with the Working Group on  Interventional Cardiology of the European Society of Cardiology*. France, 8(5), pp. 617–627. doi: 10.4244/EIJV8I5A94.

Bourantas, C. V *et al.* (2013) ‘Future perspectives in transcatheter aortic valve implantation.’, *International journal of cardiology*. Netherlands, 168(1), pp. 11–18. doi: 10.1016/j.ijcard.2013.03.065.

Bozkurt Bilen, E. *et al.* (2014) ‘The importance of echocardiography in transcatheter aortic valve implantation’, *Echocardiography*. C. Sari, Cardiology Department, Atatürk Research and Training Hospital, Ankara, Turkey, United States, 31(1), pp. 101–110. doi: 10.1111/echo.12369.

Braithwaite, S. *et al.* (2010) ‘Anaesthesia in the cardiac catheterization laboratory’, *Current Opinion in Anaesthesiology*. W. F. Buhre, Division of Perioperative and Emergency Care, University Medical Center, P.O. Box 85500, 6708 GA Utrecht, Netherlands, 23(4), pp. 507–512. doi: 10.1097/ACO.0b013e32833bb5e4.

Bramstedt, K. A. (2003) ‘Aortic valve replacement in the elderly: frequently indicated yet frequently denied.’, *Gerontology*. Switzerland, 49(1), pp. 46–49. doi: 10.1159/000066502.

Brand, M. D. *et al.* (2001) ‘Radiation-associated valvular heart disease in Hodgkin’s disease is associated with characteristic thickening and fibrosis of the aortic-mitral curtain’, *Journal of Heart Valve Disease*. T.E. Meyer, Cardiovascular Division, Univ. Massachusetts-Mem. Med. Ctr., 55 Lake Avenue North, Worcester, MA 01655, United States, 10(5), pp. 681–685. Available at: <http://www.embase.com/search/results?subaction=viewrecord&from=export&id=L32962917>.

Braxton, J. H. *et al.* (2017) ‘Transcatheter Aortic Valve Replacement: A Review’, *Surgical Clinics of North America*. J.H. Braxton, Structural Heart Services, Marshfield Clinic, Saint Joseph Hospital, 1000 North Oak Avenue, Section 2C2, Marshfield, WI, United States, 97(4), pp. 899–921. doi: 10.1016/j.suc.2017.03.011.

Bridgewater, B. (2013) ‘Almanac 2012 adult cardiac surgery: The national society journals present selected research that has driven recent advances in clinical cardiology’, *Egyptian Heart Journal*. B. Bridgewater, UHSM, Southmoor Road, Manchester M23 9LT, United Kingdom, 65(1), pp. 43–50. doi: 10.1016/j.ehj.2012.10.003.

Bristow, J. D. and Kremkau, E. L. (1975) ‘Hemodynamic changes after valve replacement with Starr-Edwards prostheses.’, *The American journal of cardiology*. United States, 35(5), pp. 716–724. doi: 10.1016/0002-9149(75)90064-8.

Brown, J. W. *et al.* (1988) ‘Surgical spectrum of aortic stenosis in children: A thirty-year experience with 257 children’, *Annals of Thoracic Surgery*. Section of Cardiothoracic Surgery, Department of Surgery, Indiana University School of Medicine, Indianapolis, IN 46223, Netherlands, 45(4), pp. 393–403. doi: 10.1016/s0003-4975(98)90012-1.

Brown, M. L. *et al.* (2012) ‘Early thrombosis risk in patients with biologic valves in the aortic position.’, *The Journal of thoracic and cardiovascular surgery*. United States, 144(1), pp. 108–111. doi: 10.1016/j.jtcvs.2011.05.032.

Buchanan, K. D. *et al.* (2017) ‘Late-breaking Trials from the 2016 American Heart Association’s Scientific Sessions’, *Cardiovascular Revascularization Medicine*. K.D. Buchanan, Heart and Vascular Institute, MedStar Washington Hospital Center, 110 Irving St NW, Suite 4B-1, Washington, DC, United States, 18(1), pp. 70–74. doi: 10.1016/j.carrev.2016.12.002.

Bugan, B. and Tuzcu, E. M. (2014) ‘[Paravalvular aortic regurgitation after transcatheter aortic valve replacement].’, *Turk Kardiyoloji Dernegi arsivi : Turk Kardiyoloji Derneginin yayin organidir*. Turkey, 42(1), pp. 83–93. doi: 10.5543/tkda.2014.93636.

Bugan, B. *et al.* (2017) ‘Acute Aortic Regurgitation in the Current Era of Percutaneous Treatment: Pathophysiology and Hemodynamics’, *The Journal of heart valve disease*, 26(1), pp. 22–31. Available at: <http://www.embase.com/search/results?subaction=viewrecord&from=export&id=L627017925>.

Burgazli, K.M. *et al.* (2012) ‘Transcatheter aortic valve implantation: Our experience and review of the literature’, *Balkan Medical Journal*. K. M. Burgazi, Department of Internal Medicine, Division of Cardiology, Giessen University, Wichlinghauser Str 110-112, 42277 Wuppertal, Germany, Turkey, 29(2), pp. 118–123. doi: 10.5152/balkanmedj.2012.004.

Burwash, I. G. (2007) ‘Low-flow, low-gradient aortic stenosis: From evaluation to treatment’, *Current Opinion in Cardiology*. I.G. Burwash, University of Ottawa Heart Institute, 40 Ruskin Street, Ottawa, Ont. K1Y 4W7, Canada, 22(2), pp. 84–91. doi: 10.1097/HCO.0b013e32801466f5.

Butala, N.; Siegfried, E. & Weissler, A. (2013) ‘Molluscum BOTE sign: A predictor of imminent resolution’, *Pediatrics*. E. Siegfried, Saint Louis University, Cardinal Glennon Children’s Hospital, 1465 South Grand Ave, St Louis, MO 63104, United States, 131(5), pp. e1650–e1653. doi: 10.1542/peds.2012-2933.

Butany, J. *et al.* (1999) ‘The Toronto SPV bioprosthesis: review of morphological findings in eight valves.’, *Seminars in thoracic and cardiovascular surgery*. United States, 11(4 Suppl 1), pp. 157–162.

Butcher, J. T.*et al.* (2011) ‘Aortic valve disease and treatment: The need for naturally engineered solutions’, *Advanced Drug Delivery Reviews*. J.T. Butcher, 304 Weill Hall, Ithaca, NY 14853, United States, Netherlands, 63(4), pp. 242–268. doi: 10.1016/j.addr.2011.01.008.

C.F.J., A., M.J., M. and A.P., K. (2017) ‘Approaches to the Role of The Heart Team in Therapeutic Decision Making for Heart Valve Disease’, *Structural Heart*. C.F.J. Antonides, Department of Cardio-Thoracic Surgery, Erasmus University Medical Center Rotterdam, Thoraxcentrum, Rotterdam, Netherlands, 1(5–6), pp. 249–255. doi: 10.1080/24748706.2017.1380377.

C.-H., C. *et al.* (2014) ‘Blastocystis hominis infection in a post-cardiotomy patient on extracorporeal membrane oxygenation support: A case report and literature review’, *International Journal of Surgery Case Reports*. N.-K. Chou, Department of Surgery, National Taiwan University Hospital, National Taiwan University College of Medicine, No. 7 Chung-Shan South Road, Taipei 100, Taiwan, 5(9), pp. 637–639. doi: 10.1016/j.ijscr.2014.07.010.

Calabro, P. *et al.* (2019) ‘Are we ready for a gender-specific approach in interventional cardiology?’, *International journal of cardiology*. Netherlands, 286, pp. 226–233. doi: 10.1016/j.ijcard.2018.11.022.

Calero Nunez, S. *et al.* (2017) ‘[Wild-type transthyretin-related cardiac amyloidosis and degenerative aortic stenosis: Two inter-related pathologies in the elderly].’, *Revista espanola de geriatria y gerontologia*. Spain, 52(3), pp. 167–170. doi: 10.1016/j.regg.2016.05.002.

Cannata, F. *et al.* (2019) ‘Mitral valve stenosis after transcatheter aortic valve replacement: Case report and review of the literature.’, *Cardiovascular revascularization medicine : including molecular interventions*. United States. doi: 10.1016/j.carrev.2019.02.023.

Cao, D. *et al.* (2018) ‘Coronary Revascularisation in Transcatheter Aortic Valve Implantation Candidates: Why, Who, When?’, *Interventional cardiology (London, England)*. England, 13(2), pp. 69–76. doi: 10.15420/icr.2018:2:2.

Capodanno, D. and Leon, M. B. (2016) ‘Upcoming TAVI trials: rationale, design and impact on clinical practice.’, *EuroIntervention : journal of EuroPCR in collaboration with the Working Group on  Interventional Cardiology of the European Society of Cardiology*. France, 12(Y), pp. Y51-5. doi: 10.4244/EIJV12SYA12.

Capranzano, P., Van Mieghem, N. M. and Tamburino, C. (2018) ‘Appraisal of key trials in aortic and mitral fields.’, *EuroIntervention : journal of EuroPCR in collaboration with the Working Group on Interventional Cardiology of the European Society of Cardiology*. France, 14(AB), pp. AB19–AB32. doi: 10.4244/EIJ-D-18-00544.

Cardoso, R. N. *et al.* (2015) ‘Heart failure functional classification following transcatheter versus surgical aortic valve replacement: A meta-analysis of three randomized controlled trials’, *Journal of the American College of Cardiology*. R.N. Cardoso, University of Miami, Jackson Memorial Hospital, Miami, FL, United States, 66(15), p. B299. Available at: <http://www.embase.com/search/results?subaction=viewrecord&from=export&id=L72065452>.

Carlier, J. (1989) ‘[Presentation of 3 cases of aortic valve stenosis and review of the literature].’, *Revue medicale de Liege*. Belgium, 44(22), pp. 665–693.

Carrascal Hinojal, Y. *et al.* (1996) ‘Extracorporeal circulation in pregnancy: report of a case. Update and review of the literature’, *Revista española de cardiología*. Y. Carrascal Hinojal, Servicio de Cirugía Cardíaca, Hospital 12 de Octubre, Madrid., 49(10), pp. 776–779. Available at: <http://www.embase.com/search/results?subaction=viewrecord&from=export&id=L127192591>.

Carrel, T.; Englberger, L. & Stalder, M. (2013) ‘Recent developments for surgical aortic valve replacement: The concept of sutureless valve technology’, *Open Journal of Cardiology*. T. Carrel, Clinic for Cardiovascular Surgery, University Hospital Berne, CH-3006 Berne, Switzerland, 4(1). Available at: <http://www.embase.com/search/results?subaction=viewrecord&from=export&id=L369492428>.

Cartier, R. and Vistarini, N. (2014) ‘Influence of atrial fibrillation in trans-catheter aortic valve replacement.’, *Minerva medica*. Italy, 105(6), pp. 467–473.

Castrovinci, S. *et al.* (2016) ‘Minimally invasive aortic valve surgery’, *Journal of Geriatric Cardiology*. K. Fattouch, Department of Cardiovascular Surgery, GVM Care and Research, Maria Eleonora Hospital, Viale Regione Siciliana 1571, Palermo, Italy, 13(6), pp. 499–503. doi: 10.11909/j.issn.1671-5411.2016.06.005.

Cauldwell, M. *et al.* (2019) ‘Cardiac interventions and cardiac surgery and pregnancy’, *International Journal of Cardiology*. M. Cauldwell, Academic Department of Obstetrics and Gynaecology, Chelsea and Westminster Hospital, 369 Fulham Road, London, United Kingdom, 276, pp. 43–47. doi: 10.1016/j.ijcard.2018.09.100.

Chakravarty, T. *et al.* (2018) ‘Complications after Transfemoral Transcatheter Aortic Valve Replacement with a Balloon-Expandable Prosthesis: The Importance of Preventative Measures and Contingency Planning.’, *Catheterization and cardiovascular interventions : official journal of the Society for Cardiac Angiography & Interventions*. United States, 91(5), pp. E29–E42. doi: 10.1002/ccd.24888.

Chambers, J. B. (2009) ‘Aortic stenosis.’, *European journal of echocardiography : the journal of the Working Group on Echocardiography of the European Society of Cardiology*. England, 10(1), pp. i11-9. doi: 10.1093/ejechocard/jen240.

Chand, E. M.; Freant, L. J. & Rubin, J. W. (1999) ‘Aortic valve rheumatoid nodules producing clinical aortic regurgitation and a review of the literature’, *Cardiovascular Pathology*. E.M. Chand, Medical College of Georgia, Department of Pathology, 1120 15th Street, Augusta, GA 30912-3605, United States, 8(6), pp. 333–338. doi: 10.1016/S1054-8807(99)00024-1.

Chandrasekhar, J. *et al.* (2017) ‘Valvular Heart Disease in Women, Differential Remodeling, and Response to New Therapies’, *Current Treatment Options in Cardiovascular Medicine*. R. Mehran, The Zena and Michael A. Wiener Cardiovascular Institute, The Icahn School of Medicine at Mount Sinai, One Gustave L. Levy Place, Box 1030, New York, NY, United States, United States, 19(9), p. 74. doi: 10.1007/s11936-017-0573-z.

Chang, Y. C. *et al.* (2012) ‘Papillary muscle stump mimicking a left ventricular mass after mitral valve replacement with partial chordal preservation’, *Asian Biomedicine*. K.C. Hung, Second Section of Cardiology, Department of Internal Medicine, Chang Gung University College of Medicine, Taipei 10591, Taiwan, 6(4), pp. 625–628. doi: 10.5372/1905-7415.0604.101.

Chapman, J. V, Bierig, S. M. and Trask, R. V (1998) ‘Evaluation of a bileaflet aortic valve prosthesis before and after thrombolytic tissue plasminogen activator therapy by continuous wave Doppler ultrasound.’, *Journal of the American Society of Echocardiography : official publication of the American Society of Echocardiography*. United States, 11(5), pp. 478–479.

Chatterjee, S. *et al.* (2014) ‘Health-related quality of life after transcatheter or surgical aortic valve replacement in high-risk patients with severe aortic stenosis: an updated review of literature.’, *Current cardiology reports*. United States, 16(4), p. 473. doi: 10.1007/s11886-014-0473-8.

Chawla, S. K. *et al.* (1977) ‘Coronary obstruction secondary to direct cannulation.’, *The Annals of thoracic surgery*. Netherlands, 23(2), pp. 135–138. doi: 10.1016/s0003-4975(10)64086-6.

Chenot, F. *et al.* (2010) ‘Evaluation of anatomic valve opening and leaflet morphology in aortic valve bioprosthesis by using multidetector CT: comparison with transthoracic echocardiography.’, *Radiology*. United States, 255(2), pp. 377–385. doi: 10.1148/radiol.0000082294.

Cheruvu, C., Blanke, P. and Leipsic, J. (2015) ‘Imaging the Aortic Annulus with Multi-Detector Computed Tomography and 3-Dimensional Transesophageal Echocardiography.’, *Interventional cardiology clinics*. Netherlands, 4(1), pp. 23–37. doi: 10.1016/j.iccl.2014.09.002.

Chester, A. H. (2011) ‘Molecular and cellular mechanisms of valve calcification’, *Aswan Heart Centre Science and Practice Series*. A.H. Chester, Imperial College London Heart Science Centre, Harefield Hospital, Harefield, Middlesex. UB9 6JH, United Kingdom, 2011(1). doi: 10.5339/ahcsps.2011.4.

Cheung, A. and Soon, J.-L. (2011) ‘Transcatheter aortic valve replacement: where will we be in 5 years?’, *Current opinion in cardiology*. United States, 26(2), pp. 106–112. doi: 10.1097/HCO.0b013e32834398ba.

Cheungpasitporn, W., Thongprayoon, C. and Kashani, K. (2016) ‘Transcatheter Aortic Valve Replacement: a Kidney’s Perspective.’, *Journal of renal injury prevention*. Iran, 5(1), pp. 1–7. doi: 10.15171/jrip.2016.01.

Chhatriwalla, A. K. *et al.* (2018) ‘Is Bioprosthetic Valve Fracture Beneficial for Patients with Larger Surgical Valves Undergoing Valve in Valve TAVR?’, *Current cardiology reports*. United States, 20(10), p. 95. doi: 10.1007/s11886-018-1035-2.

Choi, J. W. *et al.* (2018) ‘Indexed left atrial volume and 1-year mortality in patients undergoing transcatheter aortic valve replacement’, *JACC: Cardiovascular Interventions*. J.W. Choi, University of California Davis, Sacramento, CA, United States, 11(4), pp. S55–S56. doi: 10.1016/j.jcin.2018.01.182.

Christ, T. *et al.* (2017) ‘The Ross Procedure in Adults: Long-Term Results of Homografts and Stentless Xenografts for Pulmonary Valve Replacement’, *Thoracic and Cardiovascular Surgeon*. T. Christ, Department of Cardiovascular Surgery, Charité-Universitätsmedizin Berlin, Charitéplatz 1, Berlin, Germany, 65(8), pp. 656–661. doi: 10.1055/s-0036-1586157.

Christides, C. *et al.* (1989) ‘[Aortic valve replacement with extracorporeal circulation in a pregnant woman. Apropos of a case].’, *Annales de chirurgie*. France, 43(2), pp. 90–93.

Chrysohoou, C. *et al.* (2015) ‘Echocardiographic and clinical factors related to paravalvular leak incidence in  low-gradient severe aortic stenosis patients post-transcatheter aortic valve implantation.’, *European heart journal cardiovascular Imaging*. England, 16(5), pp. 558–563. doi: 10.1093/ehjci/jeu288.

Chrysohoou, C.; Tsiachris, D. & Stefanadis, C. (2011) ‘Aortic stenosis in the elderly: Challenges in diagnosis and therapy’, *Maturitas*. C. Chrysohoou, 1st Cardiology Clinic, University of Athens, 46, Polemiston Str, 16674 Glyfada, Greece, 70(4), pp. 349–353. doi: 10.1016/j.maturitas.2011.09.009.

Clavel, M.-A., Cote, N. and Pibarot, P. (2017) ‘Dilemma in the therapeutic management of low-gradient aortic stenosis.’, *Current opinion in cardiology*. United States, 32(2), pp. 147–151. doi: 10.1097/HCO.0000000000000374.

Clayton, B., Morgan-Hughes, G. and Roobottom, C. (2014) ‘Transcatheter aortic valve insertion (TAVI): a review.’, *The British journal of radiology*. England, 87(1033), p. 20130595. doi: 10.1259/bjr.20130595.

Cocchia, R. *et al.* (2017) ‘Patient selection for transcatheter aortic valve replacement: A combined clinical and multimodality imaging approach.’, *World journal of cardiology*. United States, 9(3), pp. 212–229. doi: 10.4330/wjc.v9.i3.212.

Cockburn, J. *et al.* (2012) ‘Vascular closure after transcatheter aortic valve interventions--the current state of play.’, *Journal of interventional cardiology*. United States, 25(5), pp. 526–532. doi: 10.1111/j.1540-8183.2012.00751.x.

Coeytaux, R. R. *et al.* (2010) ‘Percutaneous heart valve replacement for aortic stenosis: state of the evidence.’, *Annals of internal medicine*. United States, 153(5), pp. 314–324. doi: 10.7326/0003-4819-153-5-201009070-00267.

Cohn, L. H. (1984) ‘The long-term results of aortic valve replacement.’, *Chest*. United States, 85(3), pp. 387–396. doi: 10.1378/chest.85.3.387.

Cohn, L. H. and Narayanasamy, N. (2007) ‘Aortic valve replacement in elderly patients: what are the limits?’, *Current opinion in cardiology*. United States, 22(2), pp. 92–95. doi: 10.1097/HCO.0b013e32802086bc.

Collet, J.-P. and Montalescot, G. (2013) ‘Antithrombotic and antiplatelet therapy in TAVI patients: a fallow field?’, *EuroIntervention : journal of EuroPCR in collaboration with the Working Group on  Interventional Cardiology of the European Society of Cardiology*. France, 9 Suppl, pp. S43-7. doi: 10.4244/EIJV9SSA9.

Collins, M. J. *et al.* (2008) ‘Implications of a congenitally abnormal valve: a study of 1025 consecutively excised aortic valves.’, *Journal of clinical pathology*. England, 61(4), pp. 530–536. doi: 10.1136/jcp.2007.051904.

Comas, G. M.; McIver, B. & Thourani, V. H. (2012) ‘Innovations in aortic valve therapy for elderly patients’, *Aging Health*. V.H. Thourani, Emory University Hospital Midtown, Medical Office Tower, Cardiac Surgery, 550 Peachtree Street, Atlanta, GA 30308, United States, 8(2), pp. 179–189. doi: 10.2217/ahe.12.13.

Concha, M. *et al.* (2004) ‘The ross procedure’, *Journal of Cardiac Surgery*. P.J. Aranda, C/Las lomas 2-4-3, 14005-Córdoba, Spain, 19(5), pp. 401–409. doi: 10.1111/j.0886-0440.2004.04080.x.

Condado, J. F. *et al.* (2017) ‘End-stage renal disease and severe aortic stenosis: Does valve replacement improve one-year outcomes?’, *Catheterization and cardiovascular interventions : official journal of the Society for Cardiac Angiography & Interventions*. United States, 89(6), pp. 1109–1115. doi: 10.1002/ccd.26875.

Cone, L. A., Battista, B. A. and Shaeffer, C. W. J. (2003) ‘Endocarditis due to Peptostreptococcus anaerobius: case report and literature review of peptostreptococcal endocarditis.’, *The Journal of heart valve disease*. England, 12(3), pp. 411–413.

Cordoba-Soriano, J. G. *et al.* (2015) ‘Valve thrombosis following transcatheter aortic valve implantation: A systematic review’, *Revista Espanola de Cardiologia*. J. Rodés-Cabau, Quebec Heart and Lung Institute, Laval University, 2725 Chemin Ste-Foy, Quebec City, QC, Canada, Spain, 68(3), pp. 198–204. doi: 10.1016/j.recesp.2014.10.004.

Cormican, D. *et al.* (2019) ‘TAVR Procedural Volumes and Patient Outcomes: Analysis of Recent Data’, *Journal of Cardiothoracic and Vascular Anesthesia*. H. Ramakrishna, Division of Cardiovascular and Thoracic Anesthesiology, Department of Anesthesiology and Perioperative Medicine, Mayo Clinic, 5777 East Mayo Boulevard, Phoenix, AZ, United States. doi: 10.1053/j.jvca.2019.04.016.

Cornell, J. E. *et al.* (2017) ‘Annals understanding clinical research: Evaluating the meaning of a summary estimate in a meta-analysis’, *Annals of Internal Medicine*. J.E. Cornell, University of Texas Health Science Center at San Antonio, 7703 Merton Minter Boulevard, San Antonio, TX, United States, 167(4), pp. 275–277. doi: 10.7326/M17-1454.

Corrigan, Frank E. *et al.* (2019) ‘Imaging for Predicting, Detecting, and Managing Complications After Transcatheter Aortic Valve Replacement’, *JACC: Cardiovascular Imaging*. S. Lerakis, Icahn School of Medicine at Mount Sinai, One Gustave L. Levy Place, Box 1030, New York, NY, United States, 12(5), pp. 904–920. doi: 10.1016/j.jcmg.2018.07.036.

Costa, F. D. A. da *et al.* (2017) ‘20 years experience with the Ross operation in middle-aged patients: the autologous principle is still alive.’, *Interactive cardiovascular and thoracic surgery*. England, 24(3), pp. 348–354. doi: 10.1093/icvts/ivw365.

Coughlan, J. J. *et al.* (2018) ‘Annular Rupture During Transcatheter Aortic Valve Implantation: Predictors, Management and Outcomes.’, *Interventional cardiology (London, England)*. England, 13(3), pp. 140–144. doi: 10.15420/icr.2018.20.2.

Covello, R. D., Landoni, G. and Zangrillo, A. (2011) ‘Anesthetic management of transcatheter aortic valve implantation.’, *Current opinion in anaesthesiology*. United States, 24(4), pp. 417–425. doi: 10.1097/ACO.0b013e328347f99f.

Cowell, S. J. *et al.* (2004) ‘Calcific aortic stenosis: Same old story?’, *Age and Ageing*. S.J. Cowell, Department of Cardiology, Royal Infirmary, 51 Little France Crescent, Edinburgh EH16 4SA, United Kingdom, England, 33(6), pp. 538–544. doi: 10.1093/ageing/afh175.

Cowger, J. *et al.* (2015) ‘Comprehensive review and suggested strategies for the detection and management of aortic insufficiency in patients with a continuous-flow left ventricular assist device’, *Journal of Heart and Lung Transplantation*. J. Cowger, Department of Cardiology, St. Vincent Heart Center of Indiana, 8333 Naab Road, #400, Indianapolis, IN, United States, 34(2), pp. 149–157. doi: 10.1016/j.healun.2014.09.045.

Craver, J. M. *et al.* (1988) ‘Predictors of mortality, complications, and length of stay in aortic valve replacment for aortic stenosis’, *Circulation*. Department of Surgery, Emory University School of Medicine, Atlanta, GA, United States, 78(3 II SUPPL.), pp. I-85-I–90. Available at: <http://www.embase.com/search/results?subaction=viewrecord&from=export&id=L18215368>.

Crawford, T. C. *et al.* (2016) ‘Phase of Care Mortality Analysis: A Unique Method for Comparing Mortality Differences Among Transcatheter Aortic Valve Replacement and Surgical Aortic Valve Replacement Patients.’, *Seminars in thoracic and cardiovascular surgery*. United States, 28(2), pp. 245–252. doi: 10.1053/j.semtcvs.2016.06.008.

Culler, Steven D. *et al.* (2018) ‘Trends in Aortic Valve Replacement Procedures Between 2009 and 2015: Has Transcatheter Aortic Valve Replacement Made a Difference?’, *Annals of Thoracic Surgery*. M.R. Katz, Division of Cardiothoracic Surgery, Medical University of South Carolina, 114 Doughty St, Ste BM 282, Charleston, SC, United States, Netherlands, 105(4), pp. 1137–1143. doi: 10.1016/j.athoracsur.2017.10.057.

Cummings, C. *et al.* (2004) ‘Impact of left ventricular dysfunction on outcome in aortic stenosis patients after aortic valve replacement.’, *The journal of extra-corporeal technology*. United States, 36(4), pp. 348–350.

Curtain, J. P. and O’Brien, J. W. (2016) ‘A man with dark urine and shortness of breath: a case-based review of paravalvular leaks.’, *BMJ case reports*. England, 2016. doi: 10.1136/bcr-2015-213399.

Dahou, A. *et al.* (2015) ‘Impact and Management of Paravalvular Regurgitation After Transcatheter Aortic Valve Replacement.’, *Interventional cardiology clinics*. Netherlands, 4(1), pp. 67–82. doi: 10.1016/j.iccl.2014.09.008.

Dal-Bianco, J. P. *et al.* (2008) ‘Management of asymptomatic severe aortic stenosis.’, *Journal of the American College of Cardiology*. United States, 52(16), pp. 1279–1292. doi: 10.1016/j.jacc.2008.07.020.

Daneault, B. *et al.* (2011) ‘Stroke associated with surgical and transcatheter treatment of aortic stenosis: a comprehensive review.’, *Journal of the American College of Cardiology*. United States, 58(21), pp. 2143–2150. doi: 10.1016/j.jacc.2011.08.024.

Danson, E. *et al.* (2016) ‘Assessment, treatment, and prognostic implications of CAD in patients undergoing  TAVI.’, *Nature reviews. Cardiology*. England, 13(5), pp. 276–285. doi: 10.1038/nrcardio.2016.9.

Davidson, M. J., White, J. K. and Baim, D. S. (2006) ‘Percutaneous therapies for valvular heart disease.’, *Cardiovascular pathology : the official journal of the Society for Cardiovascular Pathology*. United States, 15(3), pp. 123–129. doi: 10.1016/j.carpath.2006.02.004.

Davies, M. J *et al.* (1996) ‘Demographic characteristics of patients undergoing aortic valve replacement for stenosis: Relation to valve morphology’, *Heart*. M.J. Davies, BHF Cardiovascular Pathology Unit, St George’s Hospital Medical School, Cranmer Terrace, London SW17 0RE, United Kingdom, England, 75(2), pp. 174–178. doi: 10.1136/hrt.75.2.174.

Davies, W. R. and Thomas, M. R. (2014) ‘European experience and perspectives on transcatheter aortic valve replacement.’, *Progress in cardiovascular diseases*. United States, 56(6), pp. 625–634. doi: 10.1016/j.pcad.2014.02.002.

Davis, E. M., Friedman, S. K. and Baker, T. M. (2013) ‘A review of antithrombotic therapy for transcatheter aortic valve replacement.’, *Postgraduate medicine*. England, 125(1), pp. 59–72. doi: 10.3810/pgm.2013.01.2625.

Davlouros, P. A. *et al.* (2018) ‘Transcatheter aortic valve replacement and stroke: a comprehensive review.’, *Journal of geriatric cardiology : JGC*. China, 15(1), pp. 95–104. doi: 10.11909/j.issn.1671-5411.2018.01.008.

Dayan, V. *et al.* (2016) ‘Predictors and Outcomes of Prosthesis-Patient Mismatch After Aortic Valve Replacement.’, *JACC. Cardiovascular imaging*. United States, 9(8), pp. 924–933. doi: 10.1016/j.jcmg.2015.10.026.

De Biase, C. *et al.* (2018) ‘What are the remaining limitations of TaVi?’, *Journal of Cardiovascular Surgery*. N. Dumonteil, Clinique Pasteur GCVI, 45 Avenue de Lombez, BP27617, Toulouse Cedex 3, France, Italy, 59(3), pp. 373–380. doi: 10.23736/s0021-9509.18.10489-7.

De Heer, L. M. *et al.* (2012) ‘Multidetector row computed tomography assessment of the native aortic and mitral valve: A call for routine assessment of left-sided heart valves during coronary computed tomography’, *Cardiology in Review*. L.M. De Heer, Department of Cardiothoracic Surgery, University Medical Center, P.O. box 85500, 3508 GA Utrecht, Netherlands, 20(5), pp. 222–229. doi: 10.1097/CRD.0b013e318250eaaa.

De Larochelliere, H. *et al.* (2019) ‘Blood Disorders in Patients Undergoing Transcatheter Aortic Valve Replacement: A Review.’, *JACC. Cardiovascular interventions*. United States, 12(1), pp. 1–11. doi: 10.1016/j.jcin.2018.09.041.

Debiais, F. *et al.* (1997) ‘Time-frequency analysis of heart murmurs. Part I: Parametric modelling and numerical simulations.’, *Medical & biological engineering & computing*. United States, 35(5), pp. 474–479.

Decena, B. F. 3rd and Tischler, M. D. (1999) ‘Stress echocardiography in valvular heart disease.’, *Cardiology clinics*. Netherlands, 17(3), pp. 555–72, ix.

Dehedin, B. *et al.* (2011) ‘Anesthesia and perioperative management of patients who undergo transfemoral transcatheter aortic valve implantation: an observational study of general versus local/regional anesthesia in 125 consecutive patients.’, *Journal of cardiothoracic and vascular anesthesia*. United States, 25(6), pp. 1036–1043. doi: 10.1053/j.jvca.2011.05.008.

Delgado, V. *et al.* (2010) ‘Multimodality imaging in transcatheter aortic valve implantation: key steps to assess procedural feasibility.’, *EuroIntervention : journal of EuroPCR in collaboration with the Working Group on  Interventional Cardiology of the European Society of Cardiology*. France, 6(5), pp. 643–652. doi: 10.4244/EIJV6I5A107.

Delgado, V. *et al.* (2011) ‘Measuring aortic valve annulus size for transcatheter aortic valve implantation - 2D or 3D imaging techniques?’, *Interventional Cardiology Review*. V. Delgado, Cardiology, Leiden University Medical Center, Albinusdreef 2, Leiden, Netherlands, 6(2), pp. 161–164. Available at: <http://www.embase.com/search/results?subaction=viewrecord&from=export&id=L615332232>.

Demerouti, E. *et al.* (2013) ‘Left ventricular geometry and systolic function improvement after percutaneous closure of aortic prosthetic paravalvular leak.’, *The Journal of heart valve disease*. E. Demerouti, Noninvasive Diagnostics Department, Cardiology Section, Onassis Cardiac Surgery Center, Athens, Greece., England, 22(6), pp. 862–866. Available at: <http://www.embase.com/search/results?subaction=viewrecord&from=export&id=L372683269>.

Demir, O. M. *et al.* (2018) ‘The Role of Cerebral Embolic Protection Devices During Transcatheter Aortic Valve Replacement.’, *Frontiers in cardiovascular medicine*. Switzerland, 5, p. 150. doi: 10.3389/fcvm.2018.00150.

Demir, O. M. *et al.* (2017) ‘Management of failing bioprosthesis in elderly patients who have undergone transcatheter aortic valve replacement.’, *Expert review of medical devices*. England, 14(10), pp. 763–771. doi: 10.1080/17434440.2017.1376651.

Desai, C. S. *et al.* (2013) ‘Transcatheter aortic valve replacement: current status and future directions.’, *Seminars in thoracic and cardiovascular surgery*. United States, 25(3), pp. 193–196. doi: 10.1053/j.semtcvs.2013.09.002.

Desser, A. S. *et al.* (2017) 'Sutureless aortic valve replacement for treatment of severe aortic stenosis: A single technology assessment of Perceval sutureless aortic valve', Oslo, Norway.

Dewhurst, A. & Rawlings, T. (2009) ‘Percutaneous valve replacement and repair in the adult: Techniques and anaesthetic considerations’, *Current Anaesthesia and Critical Care*. A. Dewhurst, Department of Anaesthesia, St George’s Hospital, London, SW17 0QT, United Kingdom, 20(4), pp. 155–159. doi: 10.1016/j.cacc.2009.02.002.

Deyell, M. W. *et al.* (2006) ‘Q fever endocarditis: a case report and review of the literature.’, *The Canadian journal of cardiology*. England, 22(9), pp. 781–785. doi: 10.1016/s0828-282x(06)70295-1.

Dharmarajan, K. *et al.* (2017) ‘The medically managed patient with severe symptomatic aortic stenosis in the TAVR era: Patient characteristics, reasons for medical management, and quality of shared decision making at heart valve treatment centers.’, *PloS one*. United States, 12(4), p. e0175926. doi: 10.1371/journal.pone.0175926.

Di Piazza, M. *et al.* (2014) ‘[TAVI in degenerative aortic stenosis treatment: state of the art and future perspectives].’, *Recenti progressi in medicina*. Italy, 105(2), pp. 63–67. doi: 10.1701/1417.15698.

[Díez-Villanueva P](https://www.ncbi.nlm.nih.gov/pubmed/?term=D%C3%ADez-Villanueva%20P%5BAuthor%5D&cauthor=true&cauthor_uid=24529484) *et al.* (2014) ‘Direct injury to right coronary artery in patients undergoing tricuspid annuloplasty’, *Annals of Thoracic Surgery*. P. Díez-Villanueva, Department of Cardiology, Hospital General Universitario Gregorio Marañón, Universidad Complutense de Madrid, C/ Doctor Esquerdo 46, 28007, Madrid, Spain, 97(4), pp. 1300–1305. doi: 10.1016/j.athoracsur.2013.12.021.

Dill, K. E. *et al.* (2013) ‘ACR appropriateness criteria imaging for transcatheter aortic valve replacement’, *Journal of the American College of Radiology*. F.J. Rybicki, American College of Radiology, 1891 Preston White Drive, Reston, VA 20191, United States, United States, 10(12), pp. 957–965. doi: 10.1016/j.jacr.2013.09.002.

Dimitrow, P. P. (2014) ‘Aortic stenosis: new pathophysiological mechanisms and their therapeutic implications.’, *Polskie Archiwum Medycyny Wewnetrznej*. Poland, 124(12), pp. 723–730.

Doenst, T. *et al.* (2018) ‘Cardiac surgery 2017 reviewed.’, *Clinical research in cardiology : official journal of the German Cardiac Society*. Germany, 107(12), pp. 1087–1102. doi: 10.1007/s00392-018-1280-9.

Doherty, J. U. *et al.* (2018) ‘ACC/AATS/AHA/ASE/ASNC/HRS/SCAI/SCCT/SCMR/STS 2017 Appropriate Use Criteria for Multimodality Imaging in Valvular Heart Disease: A Report of the American College of Cardiology Appropriate Use Criteria Task Force, American Association for Thoracic Surgery, ’, *Journal of the American Society of Echocardiography*, 31(4), pp. 381–404. doi: 10.1016/j.echo.2017.08.012.

Dohi, M., Doi, K. and Yaku, H. (2015) ‘Early stenosis of an aortic porcine bioprosthesis due to thrombosis: Case report  and literature review.’, *The Journal of thoracic and cardiovascular surgery*. United States, 149(6), pp. e83-6. doi: 10.1016/j.jtcvs.2015.02.039.

Donald, J. S. and Konstantinov, I. E. (2016) ‘Surgical Aortic Valvuloplasty Versus Balloon Aortic Valve Dilatation in Children.’, *World journal for pediatric & congenital heart surgery*. United States, 7(5), pp. 583–591. doi: 10.1177/2150135116651091.

Doss, M. and Walther, T. (2016) ‘Transcatheter/Hybrid Aortic Valves in the Young.’, *Seminars in thoracic and cardiovascular surgery. Pediatric cardiac surgery annual*. United States, 19(1), pp. 68–74. doi: 10.1053/j.pcsu.2015.12.002.

Dulgheru, R. *et al.* (2016) ‘Multimodality imaging strategies for the assessment of aortic stenosis: Viewpoint of the heart valve clinic international database (HAVEC) group’, *Circulation: Cardiovascular Imaging*. P. Lancellotti, Departments of Cardiology, GIGA Cardiovascular Sciences, Heart Valve Clinic, CHU Sart Tilman, University of Liège Hospital, Avenue de l’hôpital, n1, Liège, Belgium, United States, 9(2), p. e004352. doi: 10.1161/CIRCIMAGING.115.004352.

Dumesnil, J. G.; Pibarot, P. & Carabello, B. (2010) ‘Paradoxical low flow and/or low gradient severe aortic stenosis despite preserved left ventricular ejection fraction: Implications for diagnosis and treatment’, *European Heart Journal*. J. G. Dumesnil, Department of Medicine, Québec Heart and Lung Institute, Laval University, 2725 Chemin Sainte-Foy, Québec, QC G1V 4G5, Canada, 31(3), pp. 281–289. doi: 10.1093/eurheartj/ehp361.

Durko, A. P., Osnabrugge, R. L. and Kappetein, A. P. (2018) ‘Long-term outlook for transcatheter aortic valve replacement.’, *Trends in cardiovascular medicine*. United States, 28(3), pp. 174–183. doi: 10.1016/j.tcm.2017.08.004.

Dvir, D. *et al.* (2012) ‘[Transcatheter aortic-valve implantation].’, *Harefuah*. D. Dvir, Cardiology Department, Rabin Medical Center, Petach Tikva, Sackler Faculty of Medicine, Tel Aviv University, Tel Aviv, Israel., Israel, 151(4), pp. 237-241,252. Available at: <http://www.embase.com/search/results?subaction=viewrecord&from=export&id=L365000341>.

Dvir, D. *et al.* (2009) ‘Percutaneous aortic valve implantation in patients with coronary artery disease: review of therapeutic strategies.’, *The Journal of invasive cardiology*. D. Dvir, Department of Cardiology, Rabin Medical Center, Petach Tikva, Israel., United States, 21(12), pp. E237-241. Available at: <http://www.embase.com/search/results?subaction=viewrecord&from=export&id=L355886297>.

Dvir, D. *et al.* (2009) ‘Percutaneous aortic valve implantation: Early clinical experience and future perspectives’, *Israel Medical Association Journal*. R. Kornowski, Department of Cardiology, Rabin Medical Center, Petah Tikva 49100, Israel, Israel, 11(4), pp. 244–249. Available at: http://www.embase.com/search/results?subaction=viewrecord&from=export&id=L354765014.

Dvir, D. *et al.* (2014) ‘Transcatheter Aortic Valve-in-Valve Implantation for Patients With Degenerative Surgical Bioprosthetic Valves’, *Current Problems in Cardiology*. D. Dvir, United States, 39(1), pp. 7–27. doi: 10.1016/j.cpcardiol.2013.10.001.

Dvir, D. *et al.* (2012) ‘The development of transcatheter aortic valve replacement in the USA.’, *Archives of cardiovascular diseases*. Netherlands, 105(3), pp. 160–164. doi: 10.1016/j.acvd.2012.02.003.

Dvir, D. *et al.* (2013) ‘Paravalvular regurgitation after transcatheter aortic valve replacement: Diagnosis, clinical outcome, preventive and therapeutic strategies’, *Cardiovascular Revascularization Medicine*. R. Waksman, Interventional Cardiology, MedStar Washington Hospital Center, 110 Irving Street, Suite 4B-1, NW, Washington, DC 20010, United States, United States, 14(3), pp. 174–181. doi: 10.1016/j.carrev.2013.02.003.

Dvir, D. and Webb, J. G. (2015) ‘Transcatheter Aortic Valve-in-Valve Implantation for Patients With Degenerative Surgical Bioprosthetic Valves’, *Circulation Journal*, 79(4), pp. 695–703. doi: 10.1253/circj.CJ-14-1418.

Dworakowski, R. and Maccarthy, P. (2012) ‘Where should transcatheter aortic valve implantation go beyond 2012?’, *Journal of cardiovascular medicine (Hagerstown, Md.)*. United States, 13(8), pp. 516–523. doi: 10.2459/JCM.0b013e328354cdac.

Dworakowski, R. and Wendler, O. (2013) ‘2012 update on the Edwards SAPIENTM transcatheter heart valve.’, *Minerva cardioangiologica*. Italy, 61(4), pp. 471–482.

Dworakowski, R. and Wendler, O. (2012) ‘My rules of a perfect SAPIEN transcatheter aortic valve implantation.’, *Minerva cardioangiologica*. Italy, 60(1), pp. 71–83.

E.M.A., W. *et al.* (2016) ‘An up-to-date overview of the most recent transcatheter implantable aortic valve prostheses’, *Expert Review of Medical Devices*. J. Baan, Heart Center, Academic Medical Center, University of Amsterdam, Amsterdam, Netherlands, England, 13(1), pp. 31–45. doi: 10.1586/17434440.2016.1120665.

Eckstein, F. S. *et al.* (2000) ‘Surgery of the left ventricular outflow tract and ascending aorta in newborns, children andinfants: Own experience and review of the literature’, *Zeitschrift fur Herz-, Thorax- und Gefasschirurgie*. T.P. Carrel, Univ. Herz-und Gefaßchirurgie, Freiburgstrasse, CH-3010 Bern, Switzerland, 14(6), pp. 231–238. doi: 10.1007/s003980070001.

Edwin, F. *et al.* (2010) ‘Experience from a single centre concerning the surgical spectrum and outcome of adolescents and adults with congenitally malformed hearts in West Africa’, *Cardiology in the Young*. F. Edwin, P.O. Box KB 591, Korle Bu, Accra-Ghana, Ghana, 20(2), pp. 159–164. doi: 10.1017/S1047951109990679.

Efthymiou, C. A., Mills, R. J. and O’Regan, D. J. (2013) ‘Early postoperative thrombosis of an aortic bioprosthetic valve: should anticoagulation be patient specific?’, *Journal of cardiac surgery*. United States, 28(6), pp. 723–729. doi: 10.1111/jocs.12181.

Eggebrecht, H. *et al.* (2015) ‘The current situation and the future of emergent cardiac surgery in TAVI’, *Interventional Cardiology Review*. H. Eggebrecht, Cardioangiological Center Bethanien (CCB), Im Prüfling 23, Frankfurt, Germany, England, 10(1), pp. 55–57. doi: 10.15420/icr.2015.10.1.55.

Eisen, A. *et al.* (2012) ‘Infective endocarditis in the transcatheter aortic valve replacement era: comprehensive review of a rare complication.’, *Clinical cardiology*. United States, 35(11), pp. E1-5. doi: 10.1002/clc.22052.

El Diasty, M. *et al.* (2009) ‘Results of combined aortic valve replacement and CABG surgery in octogenarian patients during five years’, *Interactive Cardiovascular and Thoracic Surgery*. M. El Diasty, Hospital Juan Canalejo, La Coruna, Spain, 8, p. S39. doi: 10.1510/icvts.2009.0000S1.

Eleid, M. F., Thomas, J. D. and Nishimura, R. A. (2014) ‘Increased prosthetic valve gradients: abnormal prosthetic function or pressure recovery?’, *Catheterization and cardiovascular interventions : official journal of the Society for Cardiac Angiography & Interventions*. United States, 84(6), pp. 908–911. doi: 10.1002/ccd.25432.

Elkins, R. C. *et al.* (1997) ‘Congenital aortic valve disease: Improved survival and quality of life’, *Annals of Surgery*. R.C. Elkins, Thoracic Surgery, Univ. of Oklahoma Hlth. Sci. Center, P.O. Box 26901, Oklahoma City, OK 73190, United States, 225(5), pp. 503–511. doi: 10.1097/00000658-199705000-00007.

Elmariah, S. (2015) ‘Patterns of Left Ventricular Remodeling in Aortic Stenosis: Therapeutic Implications’, *Current Treatment Options in Cardiovascular Medicine*. S. Elmariah, Division of Cardiology, Massachusetts General Hospital, Harvard Medical School, Harvard Clinical Research Institute, 55 Fruit Street, GRB 800, Boston, MA, United States, 17(7), pp. 1–15. doi: 10.1007/s11936-015-0391-0.

El-Mawardy, M., Abdel-Wahab, M. and Richardt, G. (2014) ‘Transcatheter aortic valve implantation: technique, complications and perspectives.’, *Expert review of cardiovascular therapy*. England, 12(8), pp. 1005–1024. doi: 10.1586/14779072.2014.929942.

Eltchaninoff, H. *et al.* (2018) ‘TAVI and valve performance: update on definitions, durability, transcatheter heart valve failure modes and management.’, *EuroIntervention : journal of EuroPCR in collaboration with the Working Group on Interventional Cardiology of the European Society of Cardiology*. France, 14(AB), pp. AB64–AB73. doi: 10.4244/EIJ-D-18-00653.

Ennezat, P. V. *et al.* (2010) ‘Key role of Doppler echocardiography in the emergency management of elderly patients’, *Archives of Cardiovascular Diseases*. P. V. Ennezat, Cardiology Hospital, Intensive Care Unit, boulevard Pr-J.-Leclercq, 59037 Lille cedex, France, 103(2), pp. 115–128. doi: 10.1016/j.acvd.2009.11.002.

Esaki, J. *et al.* (2017) ‘Risk Factors for Late Aortic Valve Dysfunction After the David V Valve- Sparing Root Replacement.’, *The Annals of thoracic surgery*. Netherlands, 104(5), pp. 1479–1487. doi: 10.1016/j.athoracsur.2017.04.005.

Escobedo-Uribe, C. and Schoenhagen, P. (2015) ‘[Usefulness of multidetector computed tomography in transcatheter aortic valve implantation. Advantage of a tridimentional imaging modality].’, *Archivos de cardiologia de Mexico*. Mexico, 85(1), pp. 23–31. doi: 10.1016/j.acmx.2014.07.002.

Escudero, X. *et al.* (2017) ‘Coronary angiography in a patient with a previously implanted trans-catheter aortic CoreValve ®: Technical aspects, challenges and review of the literature’, *Archivos de Cardiologia de Mexico*. X. Escudero, Puente de Piedra No. 150-721, Colonia Toriello Guerra, Delegación Tlalpan, Ciudad de México, Mexico, 87(1), pp. 86–88. doi: 10.1016/j.acmx.2016.12.001.

Everett, R. J. *et al.* (2018) ‘Timing of intervention in aortic stenosis: a review of current and future strategies.’, *Heart (British Cardiac Society)*. England, 104(24), pp. 2067–2076. doi: 10.1136/heartjnl-2017-312304.

Ewe, S. H., Delgado, V. and Bax, J. J. (2012) ‘Imaging and quantification of aortic regurgitation after TAVI.’, *EuroIntervention : journal of EuroPCR in collaboration with the Working Group on  Interventional Cardiology of the European Society of Cardiology*. France, 8 Suppl Q, pp. Q21-30. doi: 10.4244/EIJV8SQA6.

F.C.R., F., G.S., S. and F., T. (2011) ‘Heyde’s syndrome:Case report and literature review’, *Arquivos Brasileiros de Cardiologia*. F. C. R. Figuinha, Rua Teodoro Sampaio, 408 / 24, Pinheiros - 05406-000 - São Paulo, SP, Brazil, 96(3), pp. e42–e45. doi: 10.1590/S0066-782X2011000300017.

Faerber, G. *et al.* (2014) ‘Valve-in-valve transcatheter aortic valve implantation: The new playground for prosthesis-patient mismatch’, *Journal of Interventional Cardiology*. T. Doenst, Department of Cardiothoracic Surgery, Friedrich Schiller University of Jena, Erlanger Allee 101, 07747 Jena, Germany, United States, 27(3), pp. 287–292. doi: 10.1111/joic.12108.

[Falcão-Pires I](https://www.ncbi.nlm.nih.gov/pubmed/?term=Falc%C3%A3o-Pires%20I%5BAuthor%5D&cauthor=true&cauthor_uid=22280423), [Gavina C](https://www.ncbi.nlm.nih.gov/pubmed/?term=Gavina%20C%5BAuthor%5D&cauthor=true&cauthor_uid=22280423), [Leite-Moreira AF](https://www.ncbi.nlm.nih.gov/pubmed/?term=Leite-Moreira%20AF%5BAuthor%5D&cauthor=true&cauthor_uid=22280423). (2012) ‘Understanding the molecular and cellular changes behind aortic valve stenosis’, *Current Pharmaceutical Biotechnology*. A. F. Leite-Moreira, Department of Physiology, Faculty of Medicine, Alameda Professor Hernâni Monteiro, 4200-319 Porto, Portugal, 13(13), pp. 2485–2496. doi: 10.2174/138920112804583050.

Farid, S. *et al.* (2015) ‘Coronary ostial compromise in aortic valve replacement: an avoidable complication.’, *Asian cardiovascular & thoracic annals*. England, 23(5), pp. 535–542. doi: 10.1177/0218492315573105.

Farkhooy, A. and Flachskampf, F. A. (2013) ‘The most important publications of the past year in echocardiography.’, *Herz*. Germany, 38(1), pp. 10–17. doi: 10.1007/s00059-012-3742-6.

Fassl, J. *et al.* (2009) ‘Anesthesia management for transapical transcatheter aortic valve implantation: a  case series.’, *Journal of cardiothoracic and vascular anesthesia*. United States, 23(3), pp. 286–291. doi: 10.1053/j.jvca.2008.12.026.

Fathala, A. *et al.* (2017) ‘Non-Cardiovascular Computed Tomography Incidental Findings in Patients Who Underwent Transaortic Valve Implantation Procedure.’, *Cardiology research*. Canada, 8(1), pp. 13–19. doi: 10.14740/cr445w.

Feldman, T. and Ali, O. (2012) ‘Transcatheter mitral valve interventions: current status and future perspective.’, *EuroIntervention : journal of EuroPCR in collaboration with the Working Group on  Interventional Cardiology of the European Society of Cardiology*. France, 8 Suppl Q, pp. Q53-9. doi: 10.4244/EIJV8SQA10.

Feldman, T., Pearson, P. and Smart, S. S. (2016) ‘Percutaneous closure of post TAVR LV apical pseudoaneurysm.’, *Catheterization and cardiovascular interventions : official journal of the Society for Cardiac Angiography & Interventions*. United States, 88(3), pp. 479–485. doi: 10.1002/ccd.26157.

[Fernández Esmerats J](https://www.ncbi.nlm.nih.gov/pubmed/?term=Fern%C3%A1ndez%20Esmerats%20J%5BAuthor%5D&cauthor=true&cauthor_uid=26651130), [Heath J](https://www.ncbi.nlm.nih.gov/pubmed/?term=Heath%20J%5BAuthor%5D&cauthor=true&cauthor_uid=26651130), [Jo H](https://www.ncbi.nlm.nih.gov/pubmed/?term=Jo%20H%5BAuthor%5D&cauthor=true&cauthor_uid=26651130). (2016) ‘Shear-Sensitive Genes in Aortic Valve Endothelium’, *Antioxidants and Redox Signaling*. H. Jo, Department of Biomedical Engineering, Emory University, Georgia Institute of Technology, 1760 Haygood Drive, Atlanta, GA, United States, 25(7), pp. 401–404. doi: 10.1089/ars.2015.6554.

Fernandez Suarez, F. E. *et al.* (2013) ‘[Anesthetic management and experience in the transcatheter implantation of the CoreValve((R)) self-expanding aortic valve].’, *Revista espanola de anestesiologia y reanimacion*. Spain, 60(8), pp. 440–447. doi: 10.1016/j.redar.2013.05.009.

Fernandez, D. *et al.* (2013) ‘Percutaneous transcatheter aortic valve implantation: present and future perspective.’, *Expert review of medical devices*. England, 10(2), pp. 185–199. doi: 10.1586/erd.12.78.

Ferrari, E. and E., F. (2012) ‘Transapical aortic “valve-in-valve” procedure for degenerated stented bioprosthesis’, *European Journal of Cardio-thoracic Surgery*. E. Ferrari, Cardiovascular Surgery Department, Centre Hôpitalier Universitaire Vaudois (CHUV), University Hospital of Lausanne, CH-1011 Lausanne, Switzerland, Germany, 41(3), pp. 485–490. doi: 10.1093/ejcts/ezr027.

Ferrari, S. *et al.* (2016) ‘[Quality of life after transcatheter aortic valve implantation: a Comprehensive literature review and critical appraisal].’, *Giornale italiano di cardiologia (2006)*, 17(12 Suppl 1), pp. 5S – 14. doi: 10.1714/2613.26897.

Feuchtner, G. (2013) ‘Imaging of cardiac valves by computed tomography.’, *Scientifica*. Egypt, 2013, p. 270579. doi: 10.1155/2013/270579.

Fields, A. V.; Mizus, M. C. & Forfia, P. (2012) ‘Premature onset aortic valve stenosis may be another end-organ complication of limited scleroderma’, *American Journal of Respiratory and Critical Care Medicine*. A.V. Fields, University of Pennsylvania, School of Medicine, Philadelphia, PA, United States, 185. Available at: <http://www.embase.com/search/results?subaction=viewrecord&from=export&id=L71986420>.

Finn, M. and Green, P. (2015) ‘The Application of Frailty to the Modern Cardiac Risk Assessment: a Case- Based Review.’, *Current cardiovascular risk reports*. United States, 9(12). doi: 10.1007/s12170-015-0476-z.

Finn, M. and Green, P. (2014) ‘Transcatheter aortic valve implantation in the elderly: who to refer?’, *Progress in cardiovascular diseases*. United States, 57(2), pp. 215–225. doi: 10.1016/j.pcad.2014.08.003.

Finn, M. T. *et al.* (2017) ‘Coronary Revascularization in Patients Undergoing Transcatheter Aortic Valve Replacement.’, *The Canadian journal of cardiology*. England, 33(9), pp. 1099–1109. doi: 10.1016/j.cjca.2017.03.016.

Fishbein, G. A. and Fishbein, M. C. (2019) ‘Pathology of the Aortic Valve: Aortic Valve Stenosis/Aortic Regurgitation.’, *Current cardiology reports*. United States, 21(8), p. 81. doi: 10.1007/s11886-019-1162-4.

Fisher, A. A. and Davis, M. W. (2004) ‘Alkaptonuric ochronosis with aortic valve and joint replacements and femoral fracture: a case report and literature review.’, *Clinical medicine & research*. United States, 2(4), pp. 209–215.

Flaherty, M. P. and Grubb, K. J. (2015) ‘Transcatheter aortic valve replacement: focus on sex-related differences in outcomes.’, *American journal of cardiovascular drugs : drugs, devices, and other interventions*. New Zealand, 15(2), pp. 95–101. doi: 10.1007/s40256-015-0110-y.

Fleming, J. *et al.* (1969) ‘Long-term results of aortic valve replacement with the Starr-Edwards valve.’, *British medical journal*. England, 1(5637), pp. 139–141. doi: 10.1136/bmj.1.5637.139.

Forcillo, J. *et al.* (2017) ‘Readmission rates after transcatheter aortic valve replacement in high- and extreme-risk patients with severe aortic stenosis.’, *The Journal of thoracic and cardiovascular surgery*. United States, 154(2), pp. 445–452. doi: 10.1016/j.jtcvs.2017.03.144.

Forcillo, J. *et al.* (2017) ‘Assessment of Commonly Used Frailty Markers for High- and Extreme-Risk Patients Undergoing Transcatheter Aortic Valve Replacement.’, *The Annals of thoracic surgery*. Netherlands, 104(6), pp. 1939–1946. doi: 10.1016/j.athoracsur.2017.05.067.

Forrest, J. K. (2012) ‘Transcatheter aortic valve replacement: design, clinical application, and future  challenges.’, *The Yale journal of biology and medicine*. United States, 85(2), pp. 239–247.]

Forrest, J. K., Mangi, A. and Vaitkeviciute, I. (2015) ‘Transcatheter aortic valve replacement: US experience.’, *Current opinion in anaesthesiology*. United States, 28(1), pp. 107–112. doi: 10.1097/ACO.0000000000000142.

Francke, M. *et al.* (2018) ‘Clinical outcomes in transcatheter aortic valve replacement recipients without electrocardiographic evidence of left ventricular hypertrophy’, *Journal of the American College of Cardiology*. M. Francke, Albany Medical Center, Albany, NY, United States, 71(11). doi: 10.1016/S0735-1097(18)31970-3.

Franco, A. *et al.* (2012) ‘Anaesthetic management of transcatheter aortic valve implantation.’, *Annals of cardiac anaesthesia*. India, 15(1), pp. 54–63. doi: 10.4103/0971-9784.91484.

Franzone, A. *et al.* (2017) ‘Evolving Indications for Transcatheter Aortic Valve Interventions.’, *Current cardiology reports*. United States, 19(11), p. 107. doi: 10.1007/s11886-017-0921-3.

Freeman, R. V.; Crittenden, G. & Otto, C. (2004) ‘Acquired aortic stenosis’, *Expert Review of Cardiovascular Therapy*. R.V. Freeman, Division of Cardiology, University of Washington, 1959 NE Pacific St., Seattle, WA 98109, United States, 2(1), pp. 107–116. doi: 10.1586/14779072.2.1.107.

Freitas-Ferraz, A. B. *et al.* (2019) ‘Aortic Stenosis and Small Aortic Annulus’, *Circulation*. United States, 139(23), pp. 2685–2702. doi: 10.1161/CIRCULATIONAHA.118.038408.

C., F. *et al.* (2012) ‘The significance of postprocedural aortic regurgitation after transcatheter aortic valve implantation on postprocedural prognosis’, *Future Cardiology*. C. Frerker, Division of Cardiology, AK St Georg Hospital, Lohmühlenstrasse 5, 20099 Hamburg, Germany, England, 8(4), pp. 637–645. doi: 10.2217/fca.12.37.

Friedman, T. *et al.* (2008) ‘Bicuspid aortic valve: Clinical approach and scientific review of a common clinical entity’, *Expert Review of Cardiovascular Therapy*. J.A. Elefteriades, Sections of Cardiac Surgery, Yale University School of Medicine, 333 Cedar St., New Haven, CT 06510, United States, England, 6(2), pp. 235–248. doi: 10.1586/14779072.6.2.235.

Fryearson, J. *et al.* (2016) ‘The role of TTE in assessment of the patient before and following TAVI for AS.’, *Echo research and practice*. England, 3(2), pp. R19-34. doi: 10.1530/ERP-16-0004.

Fu, S. & Wang, H. (2018) ‘Aortic and mitral valve replacement following previous left pneumonectomy: A case report’, *Biomedical Research (India)*. H. Wang, Department of Thoracic Surgery, The Zhejiang Provincial People’s Hospital, China, 29(10), pp. 2057–2060. Available at: <http://www.embase.com/search/results?subaction=viewrecord&from=export&id=L622756023>.

Fukui, M. *et al.* (2019) ‘Association of Structural and Functional Cardiac Changes With Transcatheter Aortic Valve Replacement Outcomes in Patients With Aortic Stenosis.’, *JAMA cardiology*. United States. doi: 10.1001/jamacardio.2018.4830.

Funder, J. A. (2012) ‘Current status on stentless aortic bioprosthesis: a clinical and experimental perspective.’, *European journal of cardio-thoracic surgery : official journal of the European Association for Cardio-thoracic Surgery*. Germany, 41(4), pp. 790–799. doi: 10.1093/ejcts/ezr141.

Furukawa, H. and Tanemoto, K. (2014) ‘Current status and future perspectives of prosthetic valve selection for aortic valve replacement.’, *General thoracic and cardiovascular surgery*. Japan, 62(1), pp. 19–23. doi: 10.1007/s11748-013-0262-0.

Furukawa, H. *et al.* (2015) ‘Current topics on bicuspid aortic valve: clinical aspects and surgical management’, *Annals of thoracic and cardiovascular surgery : official journal of the Association of Thoracic and Cardiovascular Surgeons of Asia*. Japan, 21(4), pp. 314–321. doi: 10.5761/atcs.ra.15-00130.

Fuster, V. (2016) ‘Editor-in-Chief’s Top Picks From 2015: Part Two’, *Journal of the American College of Cardiology*, 67(7), pp. 817–842. doi: 10.1016/j.jacc.2015.12.003.

Gada, H. *et al.* (2011) ‘Quality-of-life implications of immediate surgery and watchful waiting in asymptomatic aortic stenosis: a decision-analytic model.’, *Circulation. Cardiovascular quality and outcomes*. United States, 4(5), pp. 541–548. doi: 10.1161/CIRCOUTCOMES.111.961839.

Gafoor, S. *et al.* (2014) ‘Paravalvular leak closure after transcatheter aortic valve replacement with a self-expanding prosthesis.’, *Catheterization and cardiovascular interventions : official journal of the Society for Cardiac Angiography & Interventions*. United States, 84(1), pp. 147–154. doi: 10.1002/ccd.25176.

Gafoor, S. *et al.* (2015) ‘Safety of transcatheter aortic valve implantation in a hospital with visiting on-site cardiac surgery.’, *Journal of interventional cardiology*. United States, 28(1), pp. 76–81. doi: 10.1111/joic.12176.

Gallegos, R. P. (2006) ‘Selection of prosthetic heart valves’, *Current Treatment Options in Cardiovascular Medicine*. R.P. Gallegos, Division of Cardiovascular and Thoracic Surgery, Brigham and Women’s Hospital, 75 Francis Street, Boston, MA 02115, United States, 8(6), pp. 443–452. doi: 10.1007/s11936-006-0032-8.

Gallo, G. *et al.* (2019) ‘Molecular and clinical implications of natriuretic peptides in aortic valve stenosis’, *Journal of Molecular and Cellular Cardiology*. S. Rubattu, Department of Clinical and Molecular Medicine, School of Medicine and Psychology, Sapienza University of Rome, Italy, England, 129, pp. 266–271. doi: 10.1016/j.yjmcc.2019.03.011

da Gama Ribeiro, V. *et al.* (2011) ‘Vascular access in transcatheter aortic valve implantation’, *International Journal of Cardiovascular Imaging*. M.A. Costa, Harrington-McLaughlin Heart and Vascular Institute, University Hospitals, Case Western Reserve University, 11100 Euclid Ave. LKS 3001, Cleveland, OH 44106-5038, United States, United States, 27(8), pp. 1235–1243. doi: 10.1007/s10554-011-9900-8.

Gangl, C. *et al.* (2015) ‘TAVI-data in recent publications’, *Journal fur Kardiologie*. C. Gangl, Abteilung für Kardiologie, Klinik für Innere Medizin II, Medizinische Universität, Währinger Gürtel 18-20, Wien, Austria, 22(7–8), pp. 168–172. Available at: <http://www.embase.com/search/results?subaction=viewrecord&from=export&id=L605365040>.

Gardner, T. J. *et al.* (1982) ‘Valve replacement in children. A fifteen-year perspective.’, *The Journal of thoracic and cardiovascular surgery*. United States, 83(2), pp. 178–185.

Garg, V. *et al.* (2017) ‘Changes in myocardial deformation after transcatheter and surgical aortic valve replacement’, *Echocardiography*. V. Garg, Department of Medicine, Division of Cardiology, David Geffen School of Medicine at UCLA, Ronald Reagan UCLA Medical Center, Los Angeles, CA, United States, United States, 34(4), pp. 603–613. doi: 10.1111/echo.13485.

Gasior, T. *et al.* (2018) ‘Cerebral embolic protection systems for transcatheter aortic valve replacement.’, *Journal of interventional cardiology*. United States, 31(6), pp. 891–898. doi: 10.1111/joic.12573.

Gedela, M. *et al.* (2018) ‘Prosthetic Aortic Valve Endocarditis Following Transcatheter Aortic Valve Implantation’, *South Dakota medicine : the journal of the South Dakota State Medical Association*, 71(12), pp. 546–549. Available at: http://www.embase.com/search/results?subaction=viewrecord&from=export&id=L626688564.

Genereux, P. *et al.* (2013) ‘Paravalvular leak after transcatheter aortic valve replacement’, *Minerva Cardioangiologica*. M.B. Leon, Columbia University Medical Center, New York-Presbyterian Hospital, 177 Fort Washington Avenue, New York, NY 10032, United States, Italy, 61(5), pp. 529–537. Available at: <http://www.embase.com/search/results?subaction=viewrecord&from=export&id=L370496349>.

Genereux, P. *et al.* (2012) ‘Transcatheter aortic valve implantation 10-year anniversary: review of current evidence and clinical implications.’, *European heart journal*. England, 33(19), pp. 2388–2398. doi: 10.1093/eurheartj/ehs220.

Genereux, P. *et al.* (2016) ‘Natural History, Diagnostic Approaches, and Therapeutic Strategies for Patients with Asymptomatic Severe Aortic Stenosis’, *Journal of the American College of Cardiology*. P. Généreux, Columbia University Medical Center, 111 East 59th Street, New York, NY, United States, United States, 67(19), pp. 2263–2288. doi: 10.1016/j.jacc.2016.02.057.

Georgievskaya, Z. *et al.* (2014) ‘Bartonella henselae endocarditis and glomerulonephritis with dominant C3 deposition in a 21-year-old male with a Melody transcatheter pulmonary valve: case report and review of the literature.’, *Pediatric and developmental pathology : the official journal of the Society for Pediatric Pathology and the Paediatric Pathology Society*. United States, 17(4), pp. 312–320. doi: 10.2350/14-04-1462-CR.1.

Ghanem, A. *et al.* (2013) ‘Novel approaches for prevention of stroke related to transcatheter aortic valve implantation’, *Expert Review of Cardiovascular Therapy*. A. Ghanem, Department of Medicine/Cardiology, University of Bonn, Bonn, Germany, England, 11(10), pp. 1311–1320. doi: 10.1586/14779072.2013.837696.

Ghanem, A. *et al.* (2016) ‘Mechanisms And Prevention Of TAVI-Related Cerebrovascular Events.’, *Current pharmaceutical design*. United Arab Emirates, 22(13), pp. 1879–1887.

Gillam, L. D.; Marcoff, L. & Shames, S. (2014) ‘Timing of surgery in valvular heart disease: Prophylactic surgery vs watchful waiting in the asymptomatic patient’, *Canadian Journal of Cardiology*. L.D. Gillam, Department of Cardiovascular Medicine, Morristown Medical Center, 100 Madison Ave, Morristown, NJ 07962, United States, 30(9), pp. 1035–1045. doi: 10.1016/j.cjca.2014.06.019.

Gillinov, A. M. and Garcia, M. J. (2005) ‘When is concomitant aortic valve replacement indicated in patients with mild to moderate stenosis undergoing coronary revascularization?’, *Current cardiology reports*. United States, 7(2), pp. 101–104.

Gilmore, R. C. *et al.* (2016) ‘Transcatheter Aortic Valve Replacement: Current Technology and Future Directions.’, *Innovations (Philadelphia, Pa.)*. United States, 11(4), pp. 234–242. doi: 10.1097/IMI.0000000000000296.

van Gils, L. *et al.* (2016) ‘TAVI with current CE-marked devices: strategies for optimal sizing and valve delivery.’, *EuroIntervention : journal of EuroPCR in collaboration with the Working Group on Interventional Cardiology of the European Society of Cardiology*. France, 12(Y), pp. Y22-7. doi: 10.4244/EIJV12SYA6.

GinghinÇŽ, C. *et al.* (2009) ‘Calcific aortic valve disease and aortic atherosclerosis--two faces of the same disease?’, *Romanian journal of internal medicine = Revue roumaine de médecine interne*. C. Ginghinǎ, Department of Cardiology, Carol Davila University of Medicine and Pharmacy, Bucharest, Romania., 47(4), pp. 319–329. Available at: <http://www.embase.com/search/results?subaction=viewrecord&from=export&id=L360279167>.

Girdauskas, E. *et al.* (2013) ‘Increased risk of late aortic events after isolated aortic valve replacement in patients with bicuspid aortic valve insufficiency versus stenosis’, *Journal of Cardiovascular Surgery*. E. Girdauskas, Department of Cardiac Surgery, Central Clinic Bad Berka, Robert-Koch-Allee 9, 99437 Bad Berka, Germany, Italy, 54(5), pp. 653–659. Available at: <http://www.embase.com/search/results?subaction=viewrecord&from=export&id=L370114123>.

Girdauskas, E. *et al.* (2012) ‘Risk of late aortic events after an isolated aortic valve replacement for bicuspid aortic valve stenosis with concomitant ascending aortic dilation’, *European Journal of Cardio-thoracic Surgery*. E. Girdauskas, Department of Cardiac Surgery, Central Clinic Bad Berka, Robert-Koch-Allee 9, 99437 Bad Berka, Germany, Germany, 42(5), pp. 832–838. doi: 10.1093/ejcts/ezs137.

Girdauskas, E. *et al.* (2014) ‘Adverse aortic events after isolated aortic valve replacement for bicuspid aortic valve insufficiency and concomitant aortic root dilation’, *Cardiology (Switzerland)*. E. Girdauskas, Central Hospital Bad Berka, Bad Berka, Germany, 128(2), p. 176. doi: 10.1159/000362180.

Girdauskas, E. *et al.* (2013) ‘Comparison of aortic media changes in patients with bicuspid aortic valve stenosis versus bicuspid valve insufficiency and proximal aortic aneurysm’, *Interactive Cardiovascular and Thoracic Surgery*. E. Girdauskas, Department of Cardiac Surgery, Central Clinic Bad Berka, Robert-Koch-Allee 9, 99437 Bad Berka, Germany, England, 17(6), pp. 931–936. doi: 10.1093/icvts/ivt406.

Girdauskas, E. *et al.* (2016) ‘The fate of mild-to-moderate proximal aortic dilatation after isolated aortic valve replacement for bicuspid aortic valve stenosis: A magnetic resonance imaging follow-up study’, *European Journal of Cardio-thoracic Surgery*. E. Girdauskas, Department of Cardiac Surgery, Central Hospital Bad Berka, Robert-Koch-Allee 9, Bad Berka, Germany, Germany, 49(4), pp. e80–e87. doi: 10.1093/ejcts/ezv472.

Glauber, M. *et al.* (2016) ‘International Expert Consensus on Sutureless and Rapid Deployment Valves in Aortic Valve Replacement Using Minimally Invasive Approaches.’, *Innovations (Philadelphia, Pa.)*. United States, 11(3), pp. 165–173. doi: 10.1097/IMI.0000000000000287.

Godino, C. *et al.* (2013) ‘Long-term results after transcatheter aortic valve implantation: Positive and side effects’, *Minerva Cardioangiologica*. C. Godino, Cardio-Thoracic-Vascular Department, San Raffaele Scientific Institute, via Olgettina 60, 20132 Milan, Italy, Italy, 61(4), pp. 377–391. Available at: <http://www.embase.com/search/results?subaction=viewrecord&from=export&id=L369942569>.

Goeddel, L. A. *et al.* (2018) ‘Transcatheter Aortic Valve Replacements: Current Trends and Future Directions.’, *Seminars in cardiothoracic and vascular anesthesia*. United States, p. 1089253218779389. doi: 10.1177/1089253218779389.

Goel, S. S. *et al.* (2013) ‘Severe aortic stenosis and coronary artery disease--implications for management in the transcatheter aortic valve replacement era: a comprehensive review.’, *Journal of the American College of Cardiology*. United States, 62(1), pp. 1–10. doi: 10.1016/j.jacc.2013.01.096.

Gomes, B., Katus, H. A. and Bekeredjian, R. (2017) ‘Repositionable self-expanding aortic bioprosthesis.’, *Expert review of medical devices*. England, 14(7), pp. 565–576. doi: 10.1080/17434440.2017.1338136.

[Gómez de Diego](https://www.researchgate.net/profile/Jose_Gomez_de_Diego), J. J. and [De Agustín](https://www.sciencedirect.com/science/article/pii/S2211412212000545?via%3Dihub#!), J. A. (2012) ‘Clinically guided use of cardiac CT in valvular heart diseases’, *Journal of Cardiovascular Echography*. J.J. Gómez De Diego, Cardiac Imaging Laboratory, Hospital Clínico San Carlos, Profesor Martín Lagos, Madrid, Spain, 22(4), pp. 129–139. doi: 10.1016/j.jcecho.2012.09.001.

Goncalves, A., Marcos-Alberca, P. and Zamorano, J. L. (2010) ‘Echocardiography: guidance during valve implantation.’, *EuroIntervention : journal of EuroPCR in collaboration with the Working Group on  Interventional Cardiology of the European Society of Cardiology*. France, 6 Suppl G, pp. G14-9. doi: 10.4244/.

Gooley, R., Cameron, J. D. and Meredith, I. T. (2015) ‘Transcatheter Aortic Valve Implantation - Yesterday, Today and Tomorrow.’, *Heart, lung & circulation*. Australia, 24(12), pp. 1149–1161. doi: 10.1016/j.hlc.2015.07.017.

[Gošev I](https://www.ncbi.nlm.nih.gov/pubmed/?term=Go%C5%A1ev%20I%5BAuthor%5D&cauthor=true&cauthor_uid=29026447). *et al.* (2017) ‘Epigenome alterations in aortic valve stenosis and its related left ventricular hypertrophy’, *Clinical Epigenetics*. F. Paić, University of Zagreb, Laboratory for Epigenetics and Molecular Medicine, Department of Biology, School of Medicine, Šalata 3, Zagreb, Croatia, 9(1). doi: 10.1186/s13148-017-0406-7.

Gottlieb, M., Long, B. and Koyfman, A. (2018) ‘Evaluation and Management of Aortic Stenosis for the Emergency Clinician: An Evidence-Based Review of the Literature.’, *The Journal of emergency medicine*. United States, 55(1), pp. 34–41. doi: 10.1016/j.jemermed.2018.01.026.

Grabert, S., Lange, R. and Bleiziffer, S. (2016) ‘Incidence and causes of silent and symptomatic stroke following surgical and transcatheter aortic valve replacement: a comprehensive review.’, *Interactive cardiovascular and thoracic surgery*. England, 23(3), pp. 469–476. doi: 10.1093/icvts/ivw142.

Greason, K. L. *et al.* (2013) ‘Transcatheter aortic valve insertion catastrophe in inoperable patients: should aortic valve replacement be denied?’, *Journal of cardiac surgery*. United States, 28(4), pp. 336–338. doi: 10.1111/jocs.12127.

Greason, K. L. *et al.* (2013) ‘Transcatheter aortic valve replacement in patients with cirrhosis’, *Journal of Cardiac Surgery*. K.L. Greason, Division of Cardiovascular Surgery, Department of Surgery, Mayo Clinic, 200 First Street, Southwest, Rochester, 55905 MN, United States, United States, 28(5), pp. 492–495. doi: 10.1111/jocs.12177.

Greco, A. *et al.* (2019) ‘Antithrombotic pharmacotherapy after transcatheter aortic valve implantation: an update.’, *Expert review of cardiovascular therapy*. England, 17(7), pp. 479–496. doi: 10.1080/14779072.2019.1632189.

Greenbaum, A. B. *et al.* (2014) ‘Caval-aortic access to allow transcatheter aortic valve replacement in otherwise  ineligible patients: initial human experience.’, *Journal of the American College of Cardiology*. United States, 63(25 Pt A), pp. 2795–2804. doi: 10.1016/j.jacc.2014.04.015.

Grigorios, T. *et al.* (2018) ‘Transcatheter versus surgical aortic valve replacement in severe, symptomatic aortic stenosis.’, *Journal of geriatric cardiology : JGC*. China, 15(1), pp. 76–85. doi: 10.11909/j.issn.1671-5411.2018.01.002.

Grimaldi, A. *et al.* (2011) ‘[Multimodality imaging in transcatheter aortic valve implantation procedures: the pivotal role of echocardiography].’, *Giornale italiano di cardiologia (2006)*. Italy, 12(10), pp. 652–663. doi: 10.1714/945.10350.

Grover, R. *et al.* (2018) ‘Role of MDCT Imaging in Planning Mitral Valve Intervention.’, *Current cardiology reports*. United States, 20(3), p. 16. doi: 10.1007/s11886-018-0960-4.

Guinot, P.-G. *et al.* (2010) ‘Anesthesia and perioperative management of patients undergoing transcatheter aortic valve implantation: analysis of 90 consecutive patients with focus on perioperative complications.’, *Journal of cardiothoracic and vascular anesthesia*. United States, 24(5), pp. 752–761. doi: 10.1053/j.jvca.2009.12.019.

Gunn, J. and Taggart, D. P. (2019) ‘Transcatheter versus surgical intervention: lessons from trials of coronary revascularisation.’, *Heart (British Cardiac Society)*. England, 105(Suppl 2), pp. s44–s49. doi: 10.1136/heartjnl-2018-313518.

Gunter, R. L. *et al.* (2013) ‘Impact of preoperative chronic lung disease on survival after surgical aortic valve replacement.’, *The Annals of thoracic surgery*. Netherlands, 96(4), pp. 1322–1328. doi: 10.1016/j.athoracsur.2013.05.061.

Gurvitch, R. *et al.* (2011) ‘Transcatheter valve-in-valve implantation for failed surgical bioprosthetic valves.’, *Journal of the American College of Cardiology*. United States, 58(21), pp. 2196–2209. doi: 10.1016/j.jacc.2011.09.009.

Guzzardi, D. G. *et al.* (2017) ‘Bicuspid aortic valve aortopathy: Mechanistic and clinical insights from recent studies’, *Current Opinion in Cardiology*. P.W.M. Fedak, Section of Cardiac Surgery, Libin Cardiovascular Institute of Alberta, University of Calgary, C880, 1403 - 29th Street NW, Calgary, AB, Canada, United States, 32(2), pp. 111–116. doi: 10.1097/HCO.0000000000000359.

Thyregod, H.G.H. *et al.* (2015) ‘Transcatheter versus optimal medical treatment and surgical aortic valve replacement for aortic valve stenosis’, *Cochrane Database of Systematic Reviews*. H.G.H. Thyregod, Rigshospitalet, Copenhagen University Hospital, Department of Cardiothoracic Surgery, RT 2152, Blegdamsvej 9, Copenhagen, Denmark, 2015(8). doi: 10.1002/14651858.CD010488.pub2.

Ha, F. J. *et al.* (2019) ‘Outcomes of incidental findings on multi-detector computed tomography for transcatheter aortic valve implantation assessment: A single-centre study and review of the literature.’, *Journal of medical imaging and radiation oncology*. Australia. doi: 10.1111/1754-9485.12872.

Haensig, M. *et al.* (2013) ‘Experience with anatomically orientated devices for transapical aortic valve implantation’, *Minerva Cardioangiologica*. M. Haensig, Department of Cardiac Surgery, Heart Center of the University of Leipzig, Strümpellstr. 39, 04289 Leipzig, Germany, Italy, 61(1), pp. 33–43. Available at: http://www.embase.com/search/results?subaction=viewrecord&from=export&id=L368738497.

Hahn, R. T. (2016) ‘Transcathether Valve Replacement and Valve Repair: Review of Procedures and Intraprocedural Echocardiographic Imaging.’, *Circulation research*. United States, 119(2), pp. 341–356. doi: 10.1161/CIRCRESAHA.116.307972.

Hahn, R. T. (2013) ‘Use of imaging for procedural guidance during transcatheter aortic valve replacement.’, *Current opinion in cardiology*. United States, 28(5), pp. 512–517. doi: 10.1097/HCO.0b013e3283632b5e.

Hahn, R. T. and Hahn, R. T. (2014) ‘Guidance of transcatheter aortic valve replacement by echocardiography’, *Current Cardiology Reports*. R.T. Hahn, Columbia University Medical Center, New York Presbyterian Hospital, 177 Fort Washington Avenue, New York, NY 10032, United States, United States, 16(1), p. 442. doi: 10.1007/s11886-013-0442-7.

Hahn, R. T. *et al.* (2014) ‘Paravalvular regurgitation following transcutaneous aortic valve replacement: predictors and clinical significance.’, *Current cardiology reports*. United States, 16(5), p. 475. doi: 10.1007/s11886-014-0475-6.

Hahn, R. T. *et al.* (2018) ‘Echocardiographic Imaging for Transcatheter Aortic Valve Replacement.’, *Journal of the American Society of Echocardiography : official publication of the American Society of Echocardiography*. United States, 31(4), pp. 405–433. doi: 10.1016/j.echo.2017.10.022.

Halim, S. A. *et al.* (2013) ‘Transcatheter aortic valve replacement: An update’, *Current Cardiology Reports*. S.A. Halim, Division of Cardiology, Duke University Medical Center, Durham, NC 27710, United States, United States, 15(6), p. 367. doi: 10.1007/s11886-013-0367-1.

Hamasaki, A. *et al.* (2006) ‘Advantage of supra-annular patch enlargement in aortic stenosis with a small aortic annulus’, *Kyobu geka. The Japanese journal of thoracic surgery*. A. Hamasaki, Department of Cardiovascular Surgery, Heart Center, Sendai Kosei Hospital, Japan., Japan, 59(4), pp. 289–293. Available at: <http://www.embase.com/search/results?subaction=viewrecord&from=export&id=L43768391>.

Hansen, K. L.; Nielsen, M. B. & Jensen, J. A. (2017) ‘Vector velocity estimation of blood flow – A new application in medical ultrasound’, *Ultrasound*. K.L. Hansen, Department of Radiology, Rigshospitalet, Blegdamsvej 9, Copenhagen, Denmark, 25(4), pp. 189–199. doi: 10.1177/1742271X17713353.

Hariri, E. H. *et al.* (2017) ‘Transcatheter aortic valve implantation: Acute and 6-month outcomes of the first Lebanese experience and aliterature review’, *Journal Medical Libanais*. G.Y. Ghanem, School of Medicine, Lebanese American University (LAU), Byblos, Lebanon, 65(1), pp. 7–14. doi: 10.12816/0035664.

Harjai, K. J., Grines, C. L. and Leon, M. B. (2016) ‘Transcatheter Aortic Valve Replacement: 2015 in Review.’, *Journal of interventional cardiology*. United States, 29(1), pp. 27–46. doi: 10.1111/joic.12274.

Harjai, K. J. *et al.* (2017) ‘Transcatheter aortic valve replacement: The year in review 2016’, *Journal of Interventional Cardiology*. K.J. Harjai, Geisinger Clinic, Pearsall Heart Hospital, Wilkes-Barre, PA, United States, 30(2), pp. 105–113. doi: 10.1111/joic.12372.

Harky, A. *et al.* (2019) ‘Bioprosthetic Aortic Valve Replacement in <50 Years Old Patients - Where is the Evidence?’, *Brazilian journal of cardiovascular surgery*. doi: 10.21470/1678-9741-2018-0374.

Harrison, J. D. *et al.* (2014) ‘Minimally invasive aortic valve replacement with orthotopic liver transplantation: Report of a case’, *Surgery Today*. R.D. Kim, Section of Transplantation and Hepatobiliary Surgery, Department of Surgery, University of Utah, 30 North 1900 East, 3B110 SOM, Salt Lake City, UT 84132, United States, Japan, 44(3), pp. 546–549. doi: 10.1007/s00595-013-0559-8.

Haussig, S. *et al.* (2014) ‘Worldwide TAVI registries: What have we learned?’, *Clinical Research in Cardiology*. A. Linke, Department of Internal Medicine/Cardiology, Heart Center, University of Leipzig, Strümpellstrasse 39, 04289 Leipzig, Germany, Germany, 103(8), pp. 603–612. doi: 10.1007/s00392-014-0698-y.

Hauville, C. *et al.* (2012) ‘Clinical and silent stroke following aortic valve surgery and transcatheter aortic valve implantation.’, *Cardiovascular revascularization medicine : including molecular interventions*. United States, 13(2), pp. 133–140. doi: 10.1016/j.carrev.2011.11.001.

Head, S. J. *et al.* (2016) ‘Considerations and recommendations for the introduction of objective performance criteria for transcatheter aortic heart valve device approval’, *Circulation*. S.J. Head, Cardiothoracic Surgery, Erasmus MC, PO Box 2040, Rotterdam, Netherlands, United States, 133(21), pp. 2086–2093. doi: 10.1161/CIRCULATIONAHA.115.020493.

Hecker, F. *et al.* (2018) ‘Transcatheter aortic valve implantation (TAVI ) in 2018: Recent advances and future development’, *Minerva Cardioangiologica*. T. Walther, Department of Cardiac Surgery, Kerckhoff Heartcenter, Kerckhoff Heartcenter, Benekestr. 2-8, Bad Nauheim, Germany, 66(3), pp. 314–328. doi: 10.23736/S0026-4725.17.04532-7.

Hecker, F., Arsalan, M. and Walther, T. (2017) ‘Managing Stroke During Transcatheter Aortic Valve Replacement.’, *Interventional cardiology (London, England)*. England, 12(1), pp. 25–30. doi: 10.15420/icr.2016:26:1.

Heimansohn, D. *et al.* (2016) ‘North American trial results at 1 year with the Sorin Freedom SOLO pericardial aortic valve.’, *European journal of cardio-thoracic surgery : official journal of the European Association for Cardio-thoracic Surgery*. Germany, 49(2), pp. 493–9; discussion 499. doi: 10.1093/ejcts/ezv169.

Helgason, D. *et al.* (2016) ‘Acute kidney injury and outcome following aortic valve replacement for aortic stenosis.’, *Interactive cardiovascular and thoracic surgery*. England, 23(2), pp. 266–272. doi: 10.1093/icvts/ivw117.

Helms, A. S. and Bach, D. S. (2013) ‘Heart valve disease.’, *Primary care*. United States, 40(1), pp. 91–108. doi: 10.1016/j.pop.2012.11.005.

Helton, T. J., Kapadia, S. R. and Tuzcu, E. M. (2011) ‘Clinical trial experience with transcatheter aortic valve insertion.’, *The international journal of cardiovascular imaging*. United States, 27(8), pp. 1143–1154. doi: 10.1007/s10554-011-9825-2.

Henne, S. *et al.* (2007) ‘Recurrent gastrointestinal bleeding and aortic valve stenosis (Heyde syndrome): Need for valve replacement? Case report and review of the literature’, *Zeitschrift fur Gastroenterologie*. S. Henne, Klinik und Poliklinik für Kardiologie/Angiologie, Universitäres Herzzentrum Hamburg, Martinistraße 52, 20249 Hamburg, Germany, 45(3), pp. 245–249. doi: 10.1055/s-2006-927122.

Hensey, M. *et al.* (2018) ‘Impact of Chronic Kidney Disease on Decision Making and Management in Transcatheter Aortic Valve Interventions.’, *The Canadian journal of cardiology*. England. doi: 10.1016/j.cjca.2018.11.010.

Hervault, M. & M.-A., Clavel (2018) ‘Sex-related Differences in Calcific Aortic Valve Stenosis: Pathophysiology, Epidemiology, Etiology, Diagnosis, Presentation, and Outcomes’, *Structural Heart*. M.-A. Clavel, Institut Universitaire de Cardiologie et de Pneumologie de Québec (Quebec Heart and Lung Institute), 2725, Chemin Sainte-Foy, #A-2047, Québec, QC, Canada, 2(2), pp. 102–113. doi: 10.1080/24748706.2017.1420273.

Hinterbuchner, L. *et al.* (2016) ‘Frailty scoring in transcatheter aortic valve replacement patients.’, *European journal of cardiovascular nursing : journal of the Working Group on Cardiovascular Nursing of the European Society of Cardiology*. England, 15(6), pp. 384–397. doi: 10.1177/1474515115596640.

Ho, C. and Argaez, C. (2018) 'Transcatheter Aortic Valve Implantation for Patients with Severe Aortic Stenosis at Various Levels of Surgical Risk: A Review of Clinical Effectiveness', Ottawa (ON).

Ho, E. *et al.* (2014) ‘Surgical aortic valve replacement in very elderly patients aged 80 years and over: evaluation of early clinical outcomes.’, *Heart, lung & circulation*. Australia, 23(3), pp. 242–248. doi: 10.1016/j.hlc.2013.08.001.

Ho, S. Y. (2009) ‘Structure and anatomy of the aortic root.’, *European journal of echocardiography : the journal of the Working Group on Echocardiography of the European Society of Cardiology*. England, 10(1), pp. i3-10. doi: 10.1093/ejechocard/jen243.

Hoffmann, C. T., Heiner, J. A. and Nguyen, T. C. (2017) ‘Review of minimal access versus transcatheter aortic valve replacement for patients with severe aortic stenosis.’, *Annals of cardiothoracic surgery*. China, 6(5), pp. 498–503. doi: 10.21037/acs.2017.09.02.

Holzer, R.; Q.-L., Cao & Hijazi, Z. M. (2004) ‘State of the art catheter interventions in adults with congenital heart disease’, *Expert Review of Cardiovascular Therapy*. Z.M. Hijazi, Department of Pediatric Cardiology, Univ. of Chicago Children’s Hospital, 5841 S. Maryland Avenue, Chicago, IL 60637, United States, 2(5), pp. 699–711. doi: 10.1586/14779072.2.5.699.

Holzhey, D. M. *et al.* (2012) ‘Transapical aortic valve implantation - The Leipzig experience.’, *Annals of cardiothoracic surgery*. China, 1(2), pp. 129–137. doi: 10.3978/j.issn.2225-319X.2012.06.09.

Honda, K. and Okamura, Y. (2014) ‘Prosthesis-patient mismatch in aortic stenosis.’, *General thoracic and cardiovascular surgery*. Japan, 62(2), pp. 78–86. doi: 10.1007/s11748-013-0331-4.

Horrocks, J. *et al.* (2014) ‘Quality of life in older adults with aortic stenosis: a narrative review’, *International journal of older people nursing*. England, 9(3), pp. 227–246. doi: 10.1111/opn.12026.

Howard, C. *et al.* (2019) ‘The bicuspid aortic valve: Is it an immunological disease process?’, *Journal of Cardiac Surgery*. M. Bashir, Department of Emergency Medicine and Surgery, Macclesfield General Hospital, Macclesfield, United Kingdom, 34(6), pp. 482–494. doi: 10.1111/jocs.14050.

Hoyt, M. J. *et al.* (2015) ‘Predictors of Permanent Pacemaker Implantation After Transcatheter Aortic Valve Replacement.’, *Journal of cardiothoracic and vascular anesthesia*. United States, 29(5), pp. 1162–1166. doi: 10.1053/j.jvca.2015.06.001.

Hu, K. *et al.* (2013) ‘Clinical implication of mitral annular plane systolic excursion for patients with cardiovascular disease’, *European Heart Journal Cardiovascular Imaging*. F. Weidemann, Department of Internal Medicine i, Comprehensive Heart Failure Center, University of Würzburg, Oberdürrbacher Str. 6, 97080 Würzburg, Germany, 14(3), pp. 205–212. doi: 10.1093/ehjci/jes240.

Hu, P. P. (2012) ‘TAVR and SAVR: Current Treatment of Aortic Stenosis.’, *Clinical Medicine Insights. Cardiology*. United States, 6, pp. 125–139. doi: 10.4137/CMC.S7540.

Hulman, M. *et al.* (2019) ‘Transapical transcatheter aortic valve replacement with the balloon expandable aortic bioprosthetic valve in high risk patients with severe aortic stenosis: Intermediate-term results from the register of the clinic of cardiac surgery.’, *Bratislavske lekarske listy*. Slovakia, 120(6), pp. 462–467. doi: 10.4149/BLL_2019_074.

Hung, J. *et al.* (2018) ‘Take home messages with cases from focused update on echocardiographic assessment of aortic stenosis’, *Heart*. J. Hung, Division of Cardiology, Massachusetts General Hospital, Boston, MA, United States, 104(16), pp. 1317–1322. doi: 10.1136/heartjnl-2017-312917.

Hussain, M. A. *et al.* (2017) ‘Complex Structural Interventions: The Role of Computed Tomography, Fluoroscopy, and Fusion Imaging’, *Methodist DeBakey cardiovascular journal*. United States, 13(3), pp. 98–105. doi: 10.14797/mdcj-13-3-98.

Hwang, D. M., Feindel, C. M. and Butany, J. W. (2003) ‘Quadricuspid semilunar valves: report of two cases.’, *The Canadian journal of cardiology*, 19(8), pp. 938–42. Available at: <http://www.ncbi.nlm.nih.gov/pubmed/12876615>.

Hwang, I.-C., Hayashida, K. and Kim, H.-S. (2019) ‘Current Key Issues in Transcatheter Aortic Valve Replacement Undergoing a Paradigm Shift’, *Circulation Journal*, 83(5), pp. 952–962. doi: 10.1253/circj.CJ-19-0096.

Hynes, B. G. and Rodes-Cabau, J. (2012) ‘Transcatheter aortic valve implantation and cerebrovascular events: the current state of the art.’, *Annals of the New York Academy of Sciences*. United States, 1254, pp. 151–163. doi: 10.1111/j.1749-6632.2012.06477.x

Iannaccone, A. and Marwick, T. H. (2015) ‘Cost effectiveness of transcatheter aortic valve replacement compared with medical management or surgery for patients with aortic stenosis.’, *Applied health economics and health policy*. New Zealand, 13(1), pp. 29–45. doi: 10.1007/s40258-014-0141-6.

Iantorno, M. *et al.* (2018) ‘Emergent valve-in-valve transcatheter aortic valve replacement in patient with acute aortic regurgitation and cardiogenic shock with preoperative extracorporeal membrane oxygenator: A case report and review of the literature.’, *Cardiovascular revascularization medicine : including molecular interventions*. United States, 19(8S), pp. 68–70. doi: 10.1016/j.carrev.2018.11.007.

Iaselli, F. *et al.* (2011) ‘Adult-onset pulmonary involvement in Niemann-Pick disease type B’, *Monaldi Archives for Chest Disease - Cardiac Series*. F. Iaselli, Corso Trieste 273, Caserta, Italy, 75(4), pp. 235–240. doi: 10.4081/monaldi.2011.211.

Ibrahim, H. *et al.* (2018) ‘Von Willebrand factor and the aortic valve: Concepts that are important in the transcatheter aortic valve replacement era’, *Thrombosis Research*. N.S. Kleiman, 6565 Fannin, MS F-1035, Houston, TX, United States, 170, pp. 20–27. doi: 10.1016/j.thromres.2018.07.028.

Ielasi, A. *et al.* (2013) ‘Current and new-generation transcatheter aortic valve devices: An update on emerging technologies’, *Expert Review of Cardiovascular Therapy*. M. Tespili, Cardiology Division, Azienda Ospedaliera Bolognini, Seriate (BG), Italy, England, 11(10), pp. 1393–1405. doi: 10.1586/14779072.2013.837702.

Indolfi, C. *et al.* (2018) ‘Updated clinical indications for transcatheter aortic valve implantation in patients with severe aortic stenosis: expert opinion of the Italian Society of Cardiology and GISE.’, *Journal of cardiovascular medicine (Hagerstown, Md.)*. United States, 19(5), pp. 197–210. doi: 10.2459/JCM.0000000000000636.

Itchhaporia, D. (2018) ‘Transcatheter aortic valve replacement in women.’, *Clinical cardiology*. United States, 41(2), pp. 228–231. doi: 10.1002/clc.22912.

Iturra, S. A. *et al.* (2014) ‘Repeat sternotomy for surgical aortic valve replacement in octogenarian patients with aortic valve stenosis and previous coronary artery bypass graft operation: What is the operative risk?’, *Journal of Thoracic and Cardiovascular Surgery*. K.L. Greason, Division of Cardiovascular Surgery, Mayo Clinic, Rochester, MN, United States, United States, 148(5), pp. 1899–1902. doi: 10.1016/j.jtcvs.2013.11.048.

Izquierdo-Gomez, M. M. *et al.* (2017) ‘Valve Calcification in Aortic Stenosis: Etiology and Diagnostic Imaging Techniques.’, *BioMed research international*. United States, 2017, p. 5178631. doi: 10.1155/2017/5178631.

Izumo, M. and Akashi, Y. J. (2016) ‘Exercise echocardiography for structural heart disease.’, *Journal of echocardiography*. Japan, 14(1), pp. 21–29. doi: 10.1007/s12574-016-0274-8.

J.-B., O. *et al.* (2011) ‘Hyperamylasemia after cardiac surgery: Which significance?’, *Annales de Biologie Clinique*. J.-B. Oudart, CHU de Reims, Laboratoire Central de Biochimie, Reims, France, 69(2), pp. 223–227. doi: 10.1684/abc.2011.0528.

J.J.M., T. *et al.* (2001) ‘Estimated event-free life expectancy after autograft aortic root replacement in adults’, *Annals of Thoracic Surgery*. J.J.M. Takkenberg, Department of Cardiothoracic Surgery, Erasmus Medical Center Rotterdam, Bd162, PO Box 2040, 3000 CA Rotterdam, Netherlands, 71(5 SUPPL.), pp. S344–S348. doi: 10.1016/S0003-4975(01)02559-0.

J.M.I., I. *et al.* (1975) ‘Aortic valve incompetence and replacement in rheumatoid arthritis’, *Annals of the Rheumatic Diseases*. Dept. Rheumatol., Cardiol. Cardiothorac. Surg., Gen. Infirm., Leeds, England, 34(4), pp. 312–320. doi: 10.1136/ard.34.4.312.

Jagannath, A. D. *et al.* (2011) ‘Quadricuspid aortic valve: A report of 12 cases and a review of the literature’, *Echocardiography*. A.M. Johri, Kingston General Hospital, FAPC 3, 76 Stuart Street, Kingston, ON K7L 2V7, Canada, 28(9), pp. 1035–1040. doi: 10.1111/j.1540-8175.2011.01477.x.

Jahangiri, M., Edmondson, S. J. and Rees, G. M. (1995) ‘Surgery for radiation-induced valvular disease.’, *The Journal of heart valve disease*. England, 4(3), pp. 288–290.

Jahangiri, M., Hussain, A. and Akowuah, E. (2019) ‘Minimally invasive surgical aortic valve replacement.’, *Heart (British Cardiac Society)*. England, 105(Suppl 2), pp. s10–s15. doi: 10.1136/heartjnl-2018-313512.

Jain, S. (2015) ‘Transcatheter aortic valve implantation (TAVI) versus surgical aortic valve replacement (SAVR) for the treatment of aortic valve stenosis’, *European Surgical Research*. S. Jain, Faculty of Medicine, University of Liverpool, Liverpool, United Kingdom, 55, p. 91. doi: 10.1159/381839.

Javangula, K. *et al.* (2007) ‘Hyperdominant left anterior descending artery continuing across left ventricular apex as posterior descending artery coexistent with aortic stenosis.’, *Journal of cardiothoracic surgery*. K. Javangula, Yorkshire Heart Centre, Leeds General Infirmary, Great George Street, Leeds, LS1 3EX UK., England, 2, p. 42. doi: 10.1186/1749-8090-2-42.

Jilaihawi, H. *et al.* (2012) ‘Meta-analysis of complications in aortic valve replacement: comparison of Medtronic-Corevalve, Edwards-Sapien and surgical aortic valve replacement in 8,536 patients.’, *Catheterization and cardiovascular interventions : official journal of the Society for Cardiac Angiography & Interventions*. United States, 80(1), pp. 128–138. doi: 10.1002/ccd.23368.

Jin, X. Y. and Pepper, J. R. (2002) ‘Do stentless valves make a difference?’, *European journal of cardio-thoracic surgery : official journal of the European Association for Cardio-thoracic Surgery*. Germany, 22(1), pp. 95–100. doi: 10.1016/s1010-7940(02)00195-1.

Johnson, D. W. *et al.* (2017) ‘Use of Impella 5.0 Prior to Transcatheter Aortic Valve Replacement in a Patient with Severe Aortic Stenosis and Cardiogenic Shock’, *The Journal of heart valve disease*. England, 26(4), pp. 485–487. Available at: <http://www.embase.com/search/results?subaction=viewrecord&from=export&id=L622866379>.

Johnson, L. W. *et al.* (1976) ‘Familial supravalvular aortic stenosis. Report of a large family and review of the literature.’, *Chest*. United States, 70(4), pp. 494–500. doi: 10.1378/chest.70.4.494.

Jonas, R. A. (2009) ‘Fontan or Septation: When I Abandon Septation in Complex Lesions With Two Ventricles’, *Seminars in Thoracic and Cardiovascular Surgery: Pediatric Cardiac Surgery Annual*. R.A. Jonas, Cardiac Surgery, Children’s National Medical Center, Washington, DC, United States, 12(1), pp. 94–98. doi: 10.1053/j.pcsu.2009.01.007.

Jones, B. M. *et al.* (2017) ‘Matching patients with the ever-expanding range of TAVI devices’, *Nature Reviews Cardiology*. S.R. Kapadia, Robert and Suzanne Tomsich Department of Cardiovascular Medicine, Cleveland Clinic, 9500 Euclid Avenue, Cleveland, OH, United States, 14(10), pp. 615–626. doi: 10.1038/nrcardio.2017.82.

Jose, J., Manik, G. and Abdel-Wahab, M. (2016) ‘Setting up a transcatheter aortic valve implantation program: Indian perspective.’, *Indian heart journal*. India, 68(5), pp. 732–736. doi: 10.1016/j.ihj.2015.12.006.

Joseph, J. *et al.* (2017) ‘Aortic Stenosis: Pathophysiology, Diagnosis, and Therapy.’, *The American journal of medicine*. United States, 130(3), pp. 253–263. doi: 10.1016/j.amjmed.2016.10.005.

Jover, E. *et al.* (2018) ‘Cell Sources for Tissue Engineering Strategies to Treat Calcific Valve Disease.’, *Frontiers in cardiovascular medicine*. Switzerland, 5, p. 155. doi: 10.3389/fcvm.2018.00155.

Jorgensen, T. H. *et al.* (2015) ‘New-onset atrial fibrillation after surgical aortic valve replacement and transcatheter aortic valve implantation: A concise review’, *Journal of Invasive Cardiology*. T.H. Jørgensen, Departments of Cardiology, Copenhagen University Hospital, Blegdamsvej 9, Copenhagen, Denmark, United States, 27(1), pp. 41–47. Available at: <http://www.embase.com/search/results?subaction=viewrecord&from=export&id=L602217753>.

Kachel, M. *et al.* (2017) ‘State-of-the-art of transcatheter treatment of aortic valve stenosis and the overview of the InFlow project aiming at developing the first Polish TAVI system’, *Cardiology Journal*. P. Buszman, Center for Cardiovascular Research and Development of American Heart of Poland, ul. 41 Czajek, Katowice, Poland, 24(6), pp. 685–694. doi: 10.5603/CJ.a2017.0134.

Kaden, J. J. (2006) ‘Pathogenesis of calcific aortic valve stenosis’, *Herz*. J.J. Kaden, I. Medizinische Klinik, Universitäsklinikum Mannheim, Theodor-Kutzer-Ufer 1-3, 68167 Mannheim, 31(7), pp. 620–628. doi: 10.1007/s00059-006-2888-5.

Kaden, J. J. *et al.* (2003) ‘Pathogenetic role of chlamydia pneumoniae in calcific aortic stenosis: Immunohistochemistry study and review of the literature’, *Journal of Heart Valve Disease*. J.J. Kaden, Universitätsklinikum Mannheim, I. Medizinische Klinik, Theodor-Kutzer-Ufer 1-3, D-68167 Mannheim, Germany, 12(4), pp. 447–453. Available at: <http://www.embase.com/search/results?subaction=viewrecord&from=export&id=L36975031>.

Kagemoto, Y. *et al.* (2019) ‘Late Clinical Presentation of Prosthesis-Patient Mismatch Following Transcatheter Aortic Valve Replacement.’, *Journal of cardiothoracic and vascular anesthesia*. United States, 33(1), pp. 245–248. doi: 10.1053/j.jvca.2018.03.005.

Kahlert, P. *et al.* (2013) ‘Incidence, predictors, origin and prevention of early and late neurological events after transcatheter aortic valve implantation (TAVI): A comprehensive review of current data’, *Journal of Thrombosis and Thrombolysis*. P. Kahlert, Department of Cardiology, University Duisburg-Essen, Essen University Hospital, Hufelandstr.55, 45122 Essen, Germany, Netherlands, 35(4), pp. 436–449. doi: 10.1007/s11239-012-0863-y.

Kalra, A. *et al.* (2018) ‘Transcatheter and Doppler waveform correlation in transcatheter aortic valve replacement.’, *Open heart*. England, 5(1), p. e000728. doi: 10.1136/openhrt-2017-000728.

Kang, G.; Kim, J. B.; Kang, Guson & Kim, Juyong Brian (2018) ‘Expanding transcatheter aortic valve replacement into uncharted indications’, *Korean Journal of Internal Medicine*. J.B. Kim, Division of Cardiovascular Medicine, Department of Medicine and Cardiovascular Institute, Stanford University, 300 Pasteur Dr Rm CV273, Falk Bldg CVRC MC 5406, Stanford, CA, United States, 33(3), pp. 474–482. doi: 10.3904/kjim.2018.015.

Kanjanauthai, S. *et al.* (2018) ‘Subclinical leaflet thrombosis following transcatheter aortic valve replacement.’, *Journal of interventional cardiology*. United States, 31(5), pp. 640–647. doi: 10.1111/joic.12521.

Kansagra, J. *et al.* (2015) ‘Proper balloon sizing for optimal bridging aortic valvuloplasty therapy in patients awaiting transcatheter aortic valve replacement’, *Journal of the American College of Cardiology*. J. Kansagra, Henry Ford Health System, Detroit, MI, United States, 66(15), pp. B130–B131. Available at: <http://www.embase.com/search/results?subaction=viewrecord&from=export&id=L72065062>.

Kanwar, A. *et al.* (2018) ‘Management of Patients With Aortic Valve Stenosis’, *Mayo Clinic Proceedings*. V.T. Nkomo, Mayo Clinic College of Medicine and Science, 200 First St SW, Rochester, MN, United States, 93(4), pp. 488–508. doi: 10.1016/j.mayocp.2018.01.020.

Kapadia, S.R. *et al.* (2014) ‘Single center TAVR experience with a focus on the prevention and management of catastrophic complications’, *Catheterization and Cardiovascular Interventions*. E.M. Tuzcu, Heart and Vascular Institute, Cleveland Clinic, Department of Cardiology, 9500 Euclid Ave, J2-3, Cleveland, OH, United States, United States, 84(5), pp. 834–842. doi: 10.1002/ccd.25356.

Kappetein, A. P. *et al.* (2008) ‘Optimum management of elderly patients with calcified aortic stenosis.’, *Expert review of cardiovascular therapy*. England, 6(4), pp. 491–501. doi: 10.1586/14779072.6.4.491.

Kar, S. *et al.* (2010) ‘Safety and efficacy of carotid stenting in individuals with concomitant severe carotid and aortic stenosis.’, *EuroIntervention : journal of EuroPCR in collaboration with the Working Group on  Interventional Cardiology of the European Society of Cardiology*. France, 6(4), pp. 492–497. doi: 10.4244/EIJ30V6I4A82.

Karangelis, D. *et al.* (2017) ‘What is the role of sutureless aortic valves in today’s armamentarium?’, *Expert Review of Cardiovascular Therapy*. D. Karangelis, Department of Cardiac Surgery, St. Michaels’s Hospital, 30 Bond St, Toronto, ON, Canada, 15(2), pp. 83–91. doi: 10.1080/14779072.2017.1273108.

Karimov, J. H. *et al.* (2013) ‘Overview of current sutureless and transcatheter mitral valve replacement technology’, *Expert Review of Medical Devices*. K. Fukamachi, Department of Biomedical Engineering, Cleveland Clinic, 9500 Euclid Avenue/ND20, Cleveland, OH 44195, United States, England, 10(1), pp. 73–83. doi: 10.1586/erd.12.66.

Kasdi, R. *et al.* (2014) ‘To spare or not to spare.? the aortic valve in scleroderma and aortic root aneurysm’, *Journal of Cardiac Surgery*. T. Modine, Faculty of Medicine, Cardiovascular Surgery Department, Cardiologic Hospital, Lille Nord de, France, 29(1), pp. 55–58. doi: 10.1111/jocs.12239.

Kasel, A. M. *et al.* (2013) ‘Standardized imaging for aortic annular sizing: Implications for transcatheter valve selection’, *JACC: Cardiovascular Imaging*. A.M. Kasel, Clinic for Cardiology and Cardiovascular Diseases, Deutsches Herzzentrum, Technische Universität, Lazarettstrasse 36, Munich, Germany, 6(2), pp. 249–262. doi: 10.1016/j.jcmg.2012.12.005.

Kataruka, A. and Otto, C. M. (2018) ‘Valve durability after transcatheter aortic valve implantation.’, *Journal of thoracic disease*. China, 10(Suppl 30), pp. S3629–S3636. doi: 10.21037/jtd.2018.07.38.

Katayama, M. & Chaliki, H. P. (2016) ‘Diagnosis and management of patients with asymptomatic severe aortic stenosis’, *World Journal of Cardiology*. H.P. Chaliki, Division of Cardiovascular Diseases, Mayo Clinic, 13400 East Shea Boulevard, Scottsdale, AZ, United States, 8(2), pp. 192–200. doi: 10.4330/wjc.v8.i2.192.

Kayatta, M. O. *et al.* (2015) ‘Outcomes for Transcatheter Aortic Valve Replacement in Nonagenarians.’, *The Annals of thoracic surgery*. Netherlands, 100(4), pp. 1261–7; discussion 1267. doi: 10.1016/j.athoracsur.2015.04.037.

Kaza, A. K. & Gruber, P. J. (2013) ‘Surgical approaches for CHD: And update on success and challenges’, *Current Opinion in Pediatrics*. P.J. Gruber, Jensen Department in Surgery, University of Utah School of Medicine, Pediatric Cardiothoracic Surgery, 100 Mario Capecchi Drive, Salt Lake City, UT 84113, United States, 25(5), pp. 591–596. doi: 10.1097/MOP.0b013e3283650607.

Keeble, T. R. *et al.* (2016) ‘Percutaneous balloon aortic valvuloplasty in the era of transcatheter aortic valve implantation: a narrative review.’, *Open heart*. England, 3(2), p. e000421. doi: 10.1136/openhrt-2016-000421.

Kenny, D. & Hijazi, Z. M. (2012) ‘State-of-the-art percutaneous pulmonary valve therapy’, *Expert Review of Cardiovascular Therapy*. Z.M. Hijazi, Rush Center for Congenital and Structural Heart Disease, Rush University Medical Center, Chicago, IL, United States, 10(5), pp. 589–597. doi: 10.1586/erc.11.141.

Khalique, O. K. and Hahn, R. T. (2017) ‘Role of Echocardiography in Transcatheter Valvular Heart Disease Interventions.’, *Current cardiology reports*. United States, 19(12), p. 128. doi: 10.1007/s11886-017-0942-y.

Khan, A. A. *et al.* (2014) ‘Challenges in the echocardiographic assessment of aortic stenosis’, *Future Cardiology*. A.A. Khan, Department of Medicine, Massachusetts General Hospital, Harvard Medical School, Boston, MA, United States, England, 10(4), pp. 541–552. doi: 10.2217/fca.14.33.

Khatri, P. J. *et al.* (2013) ‘Adverse effects associated with transcatheter aortic valve implantation: a meta-analysis of contemporary studies.’, *Annals of internal medicine*. United States, 158(1), pp. 35–46. doi: 10.7326/0003-4819-158-1-201301010-00007.

Khawaja, M. Z., Redwood, S. R. and Thomas, M. (2014) ‘Coronary artery disease in patients undergoing TAVI--why not to treat.’, *EuroIntervention : journal of EuroPCR in collaboration with the Working Group on  Interventional Cardiology of the European Society of Cardiology*. France, 10 Suppl U, pp. U76-83. doi: 10.4244/EIJV10SUA11.

Kheiwa, A., Divanji, P. and Mahadevan, V. S. (2018) ‘Transcatheter pulmonary valve implantation: will it replace surgical pulmonary valve replacement?’, *Expert review of cardiovascular therapy*. England, 16(3), pp. 197–207. doi: 10.1080/14779072.2018.1435273.

Khetarpal, V. *et al.* (2009) ‘Calcific aortic valve and spontaneous embolic stroke: A review of literature’, *Journal of the Neurological Sciences*. N. Mahajan, Department of Medicine, Wayne State University, United States, 287(1–2), pp. 32–35. doi: 10.1016/j.jns.2009.07.018.

Kilic, T. *et al.* (2017) ‘Transcatheter aortic valve implantation: A revolution in the therapy of elderly and high-risk patients with severe aortic stenosis’, *Journal of Geriatric Cardiology*. T. Kilic, Invasive Cardiology Research and Application Unit, Department of Cardiology, Kocaeli University, Kocaeli, Turkey, China, 14(3), pp. 204–217. doi: 10.11909/j.issn.1671-5411.2017.03.002.

Kim, C. and Hong, M. K. (2019) ‘Aortic Stenosis and Transcatheter Aortic Valve Implantation: Current Status and Future Directions in Korea.’, *Korean circulation journal*. Korea (South), 49(4), pp. 283–297. doi: 10.4070/kcj.2019.0044.

Kim, W.-K. *et al.* (2017) ‘Aortic Valve Stenosis: When to Treat Interventionally, when Surgically?’, *Deutsche Medizinische Wochenschrift*. W.-K. Kim, Leitender Oberarzt Strukturelle Herzerkrankungen/TAVI, Kerckhoff-Klinik, Abteilung für Kardiologie/Herzchirurgie, Benekestr. 2-8, , Germany, Germany, 142(8), pp. 571–578. doi: 10.1055/s-0042-109890.

Kim, W.-K. *et al.* (2016) ‘Myocardial injury associated with transcatheter aortic valve implantation (TAVI).’, *Clinical research in cardiology : official journal of the German Cardiac Society*. Germany, 105(5), pp. 379–387. doi: 10.1007/s00392-015-0949-6.

Kinno, M. *et al.* (2019) ‘The transition from transesophageal to transthoracic echocardiography during transcatheter aortic valve replacement: an evolving field’, *Journal of Echocardiography*. V.H. Rigolin, Department of Medicine/Division of Cardiology, Northwestern University Feinberg School of Medicine, Bluhm Cardiovascular Institute, 675 N. St Clair St-Suite 19-100, Chicago, IL, United States, Japan, 17(1), pp. 25–34. doi: 10.1007/s12574-018-0409-1.

Kiser, A. C. *et al.* (2015) ‘Suprasternal direct aortic approach transcatheter aortic valve replacement avoids sternotomy and thoracotomy: first-in-man experiencedagger.’, *European journal of cardio-thoracic surgery : official journal of the European Association for Cardio-thoracic Surgery*. Germany, 48(5), pp. 778–83; discussion 784. doi: 10.1093/ejcts/ezu524.

Kleiman, N. S. *et al.* (2016) ‘Advances in Transcatheter Aortic Valve Replacement’, *Methodist DeBakey cardiovascular journal*. United States, 12(1), pp. 33–36. doi: 10.14797/mdcj-12-1-33.

Klein, A. *et al.* (2006) ‘Long-term mortality, cause of death, and temporal trends in complications after percutaneous aortic balloon valvuloplasty for calcific aortic stenosis’, *Journal of Interventional Cardiology*. A.D. Michaels, Division of Cardiology, University of Utah Health Sciences Center, 30 North 1900 East, Salt Lake City, UT 84132-2401, United States, 19(3), pp. 269–275. doi: 10.1111/j.1540-8183.2006.00142.x.

Klein, P. *et al.* (2018) ‘Planning for minimally invasive aortic valve replacement: Key steps for patient assessment’, *European Journal of Cardio-thoracic Surgery*. P. Kleina, Department of Cardiothoracic Surgery, St. Antonius Hospital, Koekoekslaan 1, Nieuwegein, Netherlands, Germany, 53(suppl_2), pp. ii3–ii8. doi: 10.1093/ejcts/ezy086.

Klinkhammer, B. (2018) ‘Clinical Arterial Peripheral Vascular Pathology Does Not Impact Short- or Long-Term Survival after Transcatheter Aortic Valve Replacement.’, *International journal of vascular medicine*. United States, 2018, p. 2707421. doi: 10.1155/2018/2707421.

Klinkhammer, B. (2018) ‘Transcatheter Aortic Valve Replacement After Coronary Artery Bypass Graft Is Associated With Increased Pacemaker Implantation but Not Reduced Overall Survival.’, *Cardiology research*. Canada, 9(1), pp. 40–45. doi: 10.14740/cr684w.

Knez, I. *et al.* (1996) ‘Concomitant procedures in the small versus standard aortic root.’, *The Journal of heart valve disease*. England, 5 Suppl 3, pp. S294-301.

Ko, W. *et al.* (1996) ‘Cardiopulmonary bypass procedures in patients with cold-reactive hemagglutination. A case report and a literature review’, *Journal of Cardiovascular Surgery*. W. Ko, Department of Cardiothoracic Surgery, New York Hospital, Cornell University Medical College, 525 East 68th Street, New York, NY 10021, United States, Italy, 37(6), pp. 623–626. Available at: <http://www.embase.com/search/results?subaction=viewrecord&from=export&id=L27072474>.

Kochman, J. *et al.* (2015) ‘Transcatheter aortic valve replacement in bicuspid aortic valve disease’, *Current Opinion in Cardiology*. J. Kochman, 1st Department of Cardiology, Medical University of Warsaw, ul.Banacha 1a, Warsaw, Poland, United States, 30(6), pp. 594–602. doi: 10.1097/HCO.0000000000000219.

Kojodjojo, P. *et al.* (2008) ‘Outcomes of elderly patients aged 80 and over with symptomatic, severe aortic stenosis: Impact of patient’s choice of refusing aortic valve replacement on survival’, *QJM*. P. Kojodjojo, Department of Cardiology, Hemel Hempstead General Hospital, Hillfield Road, Hertfordshire HP2 4AD, United Kingdom, England, 101(7), pp. 567–573. doi: 10.1093/qjmed/hcn052.

Konda, M. K. *et al.* (2017) ‘Aorto-right Ventricular Fistula Following Percutaneous Transcatheter Aortic Valve Replacement: Case Report and Literature Review.’, *Heart views : the official journal of the Gulf Heart Association*. India, pp. 133–136. doi: 10.4103/HEARTVIEWS.HEARTVIEWS_115_16.

Konishi, Y. *et al.* (1985) ‘Ventricular arrhythmias late after aortic and/or mitral valve replacement.’, *Japanese circulation journal*. Japan, 49(6), pp. 576–583.

Kowalewski, M. *et al.* (2019) ‘Transcathether aortic valve implantation with the new repositionable self-expandable Medtronic Evolut R vs. CoreValve system: evidence on the benefit of a meta-analytical approach.’, *Journal of cardiovascular medicine (Hagerstown, Md.)*. United States, 20(4), pp. 226–236. doi: 10.2459/JCM.0000000000000757.

Kowalowka, A. R. *et al.* (2016) ‘Do we have to operate on moderate functional mitral regurgitation during aortic valve replacement for aortic stenosis?’, *Interactive cardiovascular and thoracic surgery*. England, 23(5), pp. 806–809. doi: 10.1093/icvts/ivw212.

Kpodonu, J. and Raney, A. A. (2010) ‘Access platform techniques for transcatheter aortic valve replacement.’, *Journal of cardiac surgery*. United States, 25(4), pp. 373–380. doi: 10.1111/j.1540-8191.2010.01041.x.

Krakow, E. F. *et al.* (2009) ‘Cardiac surgery in patients with haemophilia B: a case report and review of the literature.’, *Haemophilia : the official journal of the World Federation of Hemophilia*. England, 15(1), pp. 108–113. doi: 10.1111/j.1365-2516.2008.01918.x.

Krausz, Y. *et al.* (1977) ‘Thrombosis on Bjork-Shiley aortic valve prosthesis. A case report and review of the literature.’, *Israel journal of medical sciences*. Israel, 13(4), pp. 410–413.

Krishna, R. K. *et al.* (2014) ‘Minimally invasive aortic valve replacement in octogenarians performed via a right anterior thoracotomy approach’, *The Journal of heart valve disease*. England, 23(6), pp. 671–674. Available at: <http://www.embase.com/search/results?subaction=viewrecord&from=export&id=L603856893>.

Krishnamurthy, V. K. *et al.* (2014) ‘Review of Molecular and Mechanical Interactions in the Aortic Valve and Aorta: Implications for the Shared Pathogenesis of Aortic Valve Disease and Aortopathy’, *Journal of Cardiovascular Translational Research*. R.B. Hinton, Division of Cardiology, the Heart Institute, Cincinnati Children’s Hospital Medical Center, 240 Albert Sabin Way, MLC 7020, Cincinnati, OH, United States, 7(9), pp. 823–846. doi: 10.1007/s12265-014-9602-4.

Krishnan, S. *et al.* (2018) ‘Analysis of Neurologic Complications After Surgical Versus Transcatheter Aortic Valve Replacement.’, *Journal of cardiothoracic and vascular anesthesia*. United States. doi: 10.1053/j.jvca.2018.11.011.

Kumar, G. *et al.* (2014) ‘Economic assessment of the use of transcatheter aortic valve replacement in inoperable stenotic patients in Mexico’, *Value in Health*. G. Kumar, ICON Health Economics, Oxford, United Kingdom, 17(3), p. A115. doi: 10.1016/j.jval.2014.03.670.

Kumar, G. P. *et al.* (2013) ‘Has percutaneous aortic valve replacement taken center stage in the treatment of aortic valve disease?’, *Critical Reviews in Biomedical Engineering*. F. Cui, Institute of High Performance Computing, A STAR, Singapore, United States, 41(4–5), pp. 405–424. doi: 10.1615/CritRevBiomedEng.2014010464.

Kurecko, M. *et al.* (2015) ‘[Transcatheter aortic valve implantation - diagnostic, procedure and outcomes].’, *Vnitrni lekarstvi*. Czech Republic, 61(7–8), pp. 641–648.

Kurtz, C. E. & Otto, C. M. (2010) ‘Aortic stenosis: Clinical aspects of diagnosis and management, with 10 illustrative case reports from a 25-year experience’, *Medicine*. C. E. Kurtz, Division of Cardiology, University of Washington, Box 356422, 1959 NE Pacific Street, Seattle, WA 98195, United States, 89(6), pp. 349–379. doi: 10.1097/MD.0b013e3181fe5648.

Kwan, J. (2014) ‘Three-dimensional echocardiography: A new paradigm shift’, *Journal of Echocardiography*. J. Kwan, Department of Cardiology, Inha University Hospital, 7-206, 3-Ga Shinheung-Dong, Jung-Gu, Inchon, 400-711, South Korea, 12(1), pp. 1–11. doi: 10.1007/s12574-013-0189-6

Lad, V., David, T. E. and Vegas, A. (2009) ‘Mitral regurgitation due to myxomatous degeneration combined with bicuspid aortic valve disease is often due to prolapse of the anterior leaflet of the mitral valve.’, *The Annals of thoracic surgery*. Netherlands, 87(1), pp. 79–82. doi: 10.1016/j.athoracsur.2008.09.082.

Lake, M. *et al.* (2010) ‘Ventricular noncompaction and associated cardiac anomalies.’, *The American journal of the medical sciences*. United States, 340(5), pp. 399–401. doi: 10.1097/MAJ.0b013e3181ec9431.

Lamba, H. *et al.* (2016) ‘Non-Bacterial Thrombotic Endocarditis of Aortic Valve due to Hypereosinophilic Syndrome’, *The Journal of heart valve disease*. England, 25(6), pp. 760–763. Available at: <http://www.embase.com/search/results?subaction=viewrecord&from=export&id=L621990278>.

Lansky, A. *et al.* (2018) ‘TAVI and the brain: update on definitions, evidence of neuroprotection and adjunctive pharmacotherapy.’, *EuroIntervention : journal of EuroPCR in collaboration with the Working Group on Interventional Cardiology of the European Society of Cardiology*. France, 14(AB), pp. AB53–AB63. doi: 10.4244/EIJ-D-18-00454.

Latsios, G. *et al.* (2018) ‘Multi-slice CT (MSCT) imaging in pretrans-catheter aortic valve implantation (TAVI) screening. How to perform and how to interpret’, *Hellenic Journal of Cardiology*. G. Latsios, Alexandroupoleos 9, Athens, Greece, Netherlands, 59(1), pp. 3–7. doi: 10.1016/j.hjc.2017.09.013.

Lauck, S. B. *et al.* (2018) ‘Avoidance of urinary catheterization to minimize in-hospital complications after transcatheter aortic valve implantation: An observational study.’, *European journal of cardiovascular nursing : journal of the Working Group on Cardiovascular Nursing of the European Society of Cardiology*. England, 17(1), pp. 66–74. doi: 10.1177/1474515117716590.

Lauck, S. B. *et al.* (2016) ‘Transition to palliative care when transcatheter aortic valve implantation is not an option: opportunities and recommendations’, *Current opinion in supportive and palliative care*. United States, 10(1), pp. 18–23. doi: 10.1097/SPC.0000000000000180.

Le Tourneau, T. *et al.* (1999) ‘Mid-term comparative follow-up after aortic valve replacement with Carpentier-Edwards and Pericarbon pericardial prostheses.’, *Circulation*. United States, 100(19 Suppl), pp. II11-6. doi: 10.1161/01.cir.100.suppl_2.ii-11.

Le Tourneau, T. *et al.* (2002) ‘Ten-year echocardiographic and clinical follow-up of aortic Carpentier-Edwards pericardial and supraannular prosthesis: a case-match study.’, *The Annals of thoracic surgery*. Netherlands, 74(6), pp. 2010–2015. doi: 10.1016/s0003-4975(02)04032-8.

Lee, J. J., Goldschlager, N. and Mahadevan, V. S. (2018) ‘Atrioventricular and intraventricular block after transcatheter aortic valve implantation.’, *Journal of interventional cardiac electrophysiology : an international journal of arrhythmias and pacing*. Netherlands, 52(3), pp. 315–322. doi: 10.1007/s10840-018-0391-6.

Lehmkuhl, L. *et al.* (2013) ‘Role of preprocedural computed tomography in transcatheter aortic valve implantation.’, *RöFo : Fortschritte auf dem Gebiete der Röntgenstrahlen und der Nuklearmedizin*. L. Lehmkuhl, Dept. of Interventional and Diagnostic Radiology, University of Leipzig - Heart Center, Leipzig., Germany, 184(10), pp. 941–949. doi: 10.1055/s-0033-1350416.

Leipsic, J. *et al.* (2011) ‘Multidetector computed tomography in transcatheter aortic valve implantation.’, *JACC. Cardiovascular imaging*. United States, 4(4), pp. 416–429. doi: 10.1016/j.jcmg.2011.01.014.

Lewin, R. F. *et al.* (1989) ‘Aortic annular tear after valvuloplasty: The role of aortic annulus echocardiographic measurement’, *Catheterization and Cardiovascular Diagnosis*. St. Luke’s Medical Center, Milwaukee, WI 53215, 16(2), pp. 123–129. doi: 10.1002/ccd.1810160212.

Li, Z. *et al.* (2019) ‘Frailty in patients undergoing transcatheter aortic valve implantation: a protocol for a systematic review.’, *BMJ open*. England, 9(2), p. e024163. doi: 10.1136/bmjopen-2018-024163.

Liakos, C.I. *et al.* (2017) ‘Arterial hypertension and aortic valve stenosis: Shedding light on a common “liaison”’, *Hellenic Journal of Cardiology*. C.I. Liakos, ESH Centre of Excellence for Hypertension, 1st Cardiology Department, “Hippokration” Hospital, National and Kapodistrian University of Athens, Medical School, 108 V. Sofias Avenue, Athens, Greece, 58(4), pp. 261–266. doi: 10.1016/j.hjc.2017.03.005.

Liebe, V. *et al.* (2006) ‘Statin therapy of calcific aortic stenosis: Hype or hope?’, *European Heart Journal*. J.J. Kaden, First Department of Medicine (Cardiology), University Hospital Mannheim, Theodor-Kutzer-Ufer 1-3, D-68167 Mannheim, Germany, 27(7), pp. 773–778. doi: 10.1093/eurheartj/ehi697.

Lindman, B. R. *et al.* (2014) ‘Futility, benefit, and transcatheter aortic valve replacement’, *JACC: Cardiovascular Interventions*. B.R. Lindman, Washington University, School of Medicine, Campus Box 8086, 660 South Euclid Avenue, St. Louis, MO 63110, United States, United States, 7(7), pp. 707–716. doi: 10.1016/j.jcin.2014.01.167.

Lindman, B. R. and Patel, J. N. (2016) ‘Multimorbidity in Older Adults with Aortic Stenosis.’, *Clinics in geriatric medicine*. United States, 32(2), pp. 305–314. doi: 10.1016/j.cger.2016.01.006.

Litmanovich, D. E. *et al.* (2014) ‘Imaging in Transcatheter Aortic Valve Replacement (TAVR): role of the radiologist.’, *Insights into imaging*. Germany, 5(1), pp. 123–145. doi: 10.1007/s13244-013-0301-5.

Little, S. H. *et al.* (2012) ‘Multimodality noninvasive imaging for transcatheter aortic valve implantation: a primer.’, *Methodist DeBakey cardiovascular journal*. S.H. Little, Methodist DeBakey Heart & Vascular Center, The Methodist Hospital, Houston, Texas, USA., United States, 8(2), pp. 29–37. Available at: <http://www.embase.com/search/results?subaction=viewrecord&from=export&id=L366359441>.

Litwin, S. B. *et al.* (2007) ‘The double patch repair for complete atrioventricularis communis.’, *Seminars in thoracic and cardiovascular surgery. Pediatric cardiac surgery annual*. S.B. Litwin, Department of Cardiothoracic Surgery, Herma Heart Center, Children’s Hospital of Wisconsin, Medical College of Wisconsin, Milwaukee, WI, USA., pp. 21–27. doi: 10.1053/j.pcsu.2007.01.003.

Lo, J. and Hill, C. (2015) ‘Intensive care unit management of transcatheter aortic valve recipients.’, *Seminars in cardiothoracic and vascular anesthesia*. United States, 19(2), pp. 95–105. doi: 10.1177/1089253215575183.

Logeais, Y. *et al.* (2006) ‘[Cardiac surgery in the elderly].’, *Bulletin de l’Academie nationale de medecine*. Netherlands, 190(4–5), pp. 855–856.

Logeais, Y. *et al.* (2007) ‘Surgery for aortic valve stenosis after age 80 years’, *Bulletin de l’Académie nationale de médecine*. Y. Logeais, Centre Cardio-Pneumologique, Hôpital Pontchaillou, CHU 35033 Rennes cedex 9., 191(2), pp. 245–258. Available at: <http://www.embase.com/search/results?subaction=viewrecord&from=export&id=L350324424>.

Logeais, Y.; Leguerrier, A. & Rioux, C. (1984) ‘Surgery for calcific aortic stenosis in subjects aged 70 and over. Review of 229 cases’, *Annales de Cardiologie et d’Angeiologie*. Clinique Chirurgicale Cardiovasculaire et Thoracique, Centre Hospitalier Universitaire, Pontchaillou, 35000 Rennes, 33(6), pp. 385–388. Available at: http://www.embase.com/search/results?subaction=viewrecord&from=export&id=L14040538.

Logeais, Y. *et al.* (1991) ‘[A comparative evaluation of a prosthetic operation and balloon valvuloplasty in  aortic valve stenosis in elderly people].’, *Grudnaia i serdechno-sosudistaia khirurgiia*. Russia (Federation), (11), pp. 3–9.

Loh, P. H., Bundgaard, H. and S Ndergaard, L. (2013) ‘Infective endocarditis following transcatheter aortic valve replacement-: diagnostic and management challenges.’, *Catheterization and cardiovascular interventions : official journal of the Society for Cardiac Angiography & Interventions*. United States, 81(4), pp. 623–627. doi: 10.1002/ccd.24368.

Long, A. and Mahoney, P. (2018) ‘Fulminant presentation of a failed TAVR valve: Successful revision with a transcatheter approach - Case report and review of the literature.’, *Cardiovascular revascularization medicine : including molecular interventions*. United States. doi: 10.1016/j.carrev.2018.10.018.

Loomba, R. S. *et al.* (2015) ‘Is Aortic Valve Leaflet Morphology Predictive of Outcome in Pediatric Aortic Valve Stenosis?’, *Congenital Heart Disease*. R.S. Loomba, Division of Pediatric Cardiology, Children’s Hospital of Wisconsin, Milwaukee, WI, United States, 10(6), pp. 552–560. doi: 10.1111/chd.12278.

Lopez-Mattei, J. C. and Shah, D. J. (2013) ‘When to consider cardiovascular magnetic resonance in patients undergoing transcatheter aortic valve replacement?’, *Current opinion in cardiology*. United States, 28(5), pp. 505–511. doi: 10.1097/HCO.0b013e32836245fb.

Lorusso, R. *et al.* (2007) ‘Coronary-to-bronchial anastomosis: an unusual cause of hemoptysis.’, *Journal of cardiovascular medicine (Hagerstown, Md.)*. United States, 8(8), pp. 642–644. doi: 10.2459/01.JCM.0000281695.31112.90.

Lorusso, R.; Gelsomino, S. & Renzulli, A. (2013) ‘Sutureless aortic valve replacement: An alternative to transcatheter aortic valve implantation?’, *Current Opinion in Cardiology*. R. Lorusso, Cardiac Surgery Unit, Community Hospital, Piazzale Spedali Civili, 1, 215128 Brescia, Italy, 28(2), pp. 158–163. doi: 10.1097/HCO.0b013e32835da4b2.

Luciani, G. B. *et al.* (2014) ‘Two decades of experience with the Ross operation in neonates, infants and children from the Italian Paediatric Ross Registry’, *Heart*. G.B. Luciani, Division of Cardiac Surgery, Department of Surgery, University of Verona, O. C. M. Piazzale Stefani 1, Verona, Italy, England, 100(24), pp. 1954–1959. doi: 10.1136/heartjnl-2014-305873

Luk, A., David, T. E. and Butany, J. (2012) ‘Complications of Bioglue postsurgery for aortic dissections and aortic valve replacement.’, *Journal of clinical pathology*. England, 65(11), pp. 1008–1012. doi: 10.1136/jclinpath-2012-200809.

Lynch, D. R. J. *et al.* (2013) ‘Considerations in antithrombotic therapy among patients undergoing transcatheter aortic valve implantation’, *Journal of Thrombosis and Thrombolysis*. D.R. Lynch Jr., Division of Cardiology, Vanderbilt Heart and Vascular Institute, Vanderbilt University Medical Center, 2220 Pierce Avenue 383 PRB, Nashville TN 37232-6300, United States, Netherlands, 35(4), pp. 476–482. doi: 10.1007/s11239-013-0886-z.

M.-A., D. *et al.* (2013) ‘Beyond adding years to life: Health-related quality-of-life and functional outcomes in patients with severe aortic valve stenosis at high surgical risk undergoing transcatheter aortic valve replacement’, *Current Cardiology Reviews*. M.-A. Deutsch, Department of Cardiovascular Surgery, German Heart Center Munich, Technische Universität München, Lazarettstrasse 36, 80636 Munich, Germany, United Arab Emirates, 9(4), pp. 281–294. doi: 10.2174/1573403X09666131202121750.

M.-Y., H. and S., T. (2014) ‘What is the ultimate test that lowering lipoprotein(a) is beneficial for cardiovascular disease and aortic stenosis?’, *Current Opinion in Lipidology*. S. Tsimikas, School of Medicine, University of California San Diego, Vascular Medicine Program, 9500 Gilman Drive, San Diego, CA, United States, 25(6), pp. 423–430. doi: 10.1097/MOL.0000000000000131.

Ma, G. S. *et al.* (2019) ‘Lipoprotein(a) in Patients Undergoing Transcatheter Aortic Valve Replacement’, *Angiology*. S. Tsimikas, Division of Cardiovascular Diseases, Sulpizio Cardiovascular Center, Vascular Medicine Program, University of California San Diego, San Diego, United States, United States, 70(4), pp. 332–336. doi: 10.1177/0003319719826461.

Magkoutis, N. A. *et al.* (2016) ‘Antiplatelet therapy in TAVI: Current clinical practice and recommendations’, *Current Pharmaceutical Design*. N.A. Magkoutis, 133, Avenue de la Résistance, , Le Plessis-Robinson, France, United Arab Emirates, 22(13), pp. 1888–1895. Available at: <http://www.embase.com/search/results?subaction=viewrecord&from=export&id=L610088982>.

Mahajan, N.; Khetarpal, V. & Afonso, L. (2009) ‘Stroke secondary to calcific bicuspid aortic valve: Case report and literature review’, *Journal of Cardiology*. N. Mahajan, Department of Medicine, Detroit Medical Center, Wayne State University School of Medicine, Detroit, MI, United States, 54(1), pp. 158–161. doi: 10.1016/j.jjcc.2008.10.011.

Maher, K. O. & Tweddell, J. S. (2016) ‘Aortic and mitral valve disease and left ventricular dysfunction in children’, *Pediatric Critical Care Medicine*. J.S. Tweddell, Department of Surgery, Cincinnati College of Medicine, Cincinnati Children’s Hospital Medical Center, Cincinnati, OH, United States, 17(8), pp. S131–S139. doi: 10.1097/PCC.0000000000000828.

Mahtta, D., Elgendy, I. Y. and Bavry, A. A. (2017) ‘From CoreValve to Evolut PRO: Reviewing the Journey of Self-Expanding Transcatheter Aortic Valves.’, *Cardiology and therapy*. England, 6(2), pp. 183–192. doi: 10.1007/s40119-017-0100-z.

Maisano, F. *et al.* (2006) ‘Percutaneous valve interventions’, *Current Cardiology Reviews*. F. Maisano, Department of Cardiac Surgery, San Raffaele Hospital, Via Olgettina 60, 20132 Milano, Italy, 2(1), pp. 29–36. Available at: <http://www.embase.com/search/results?subaction=viewrecord&from=export&id=L44082919>.

Makdisi, G., Makdisi, P. B. and Wang, I.-W. (2016) ‘Use of extracorporeal membranous oxygenator in transcatheter aortic valve replacement.’, *Annals of translational medicine*. China, 4(16), p. 306. doi: 10.21037/atm.2016.08.14.

Makki, N. *et al.* (2017) ‘Conduction disturbances after TAVR: Electrophysiological studies and pacemaker dependency.’, *Cardiovascular revascularization medicine : including molecular interventions*. United States, 18(5S1), pp. S10–S13. doi: 10.1016/j.carrev.2017.03.009.

Malaisrie, S. C. *et al.* (2011) ‘Transcatheter aortic valve implantation decreases the rate of unoperated aortic stenosis.’, *European journal of cardio-thoracic surgery : official journal of the European Association for Cardio-thoracic Surgery*. Germany, 40(1), pp. 43–48. doi: 10.1016/j.ejcts.2010.11.031.

Maluenda, G. *et al.* (2014) ‘Expanding indications for transcatheter aortic valve replacement’, *Expert Review of Cardiovascular Therapy*. G. Maluenda, Cardiovascular Center, Hospital San Borja Arriaran, School of Medicine, University of Chile, Santiago, Chile, England, 12(6), pp. 693–702. doi: 10.1586/14779072.2014.916615.

Mangieri, A. *et al.* (2017) ‘Single-Antiplatelet Therapy in Patients with Contraindication to Dual-Antiplatelet Therapy After Transcatheter Aortic Valve Implantation’, *American Journal of Cardiology*. A. Mangieri, Cardiothoracic Department, San Raffaele Scientific Institute, Milan, Italy, 119(7), pp. 1088–1093. doi: 10.1016/j.amjcard.2016.11.065.

Mangieri, A. *et al.* (2018) ‘Predictors of Advanced Conduction Disturbances Requiring a Late (>/=48 H) Permanent Pacemaker Following Transcatheter Aortic Valve Replacement.’, *JACC. Cardiovascular interventions*. United States, 11(15), pp. 1519–1526. doi: 10.1016/j.jcin.2018.06.014.

Mangieri, A. *et al.* (2018) ‘TAVI and Post Procedural Cardiac Conduction Abnormalities.’, *Frontiers in cardiovascular medicine*. Switzerland, 5, p. 85. doi: 10.3389/fcvm.2018.00085.

Mangieri, A. *et al.* (2015) ‘Recent advances in transcatheter aortic valve replacement for high-risk patients.’, *Expert review of cardiovascular therapy*. England, 13(11), pp. 1237–1249. doi: 10.1586/14779072.2015.1093935.

Mangla, A. and Gupta, S. (2016) ‘Vascular complications post-transcatheter aortic valve procedures.’, *Indian heart journal*. India, 68(5), pp. 724–731. doi: 10.1016/j.ihj.2015.11.024.

Manning, W. J. (2013) ‘Asymptomatic aortic stenosis in the elderly: a clinical review.’, *JAMA*. United States, 310(14), pp. 1490–1497. doi: 10.1001/jama.2013.279194.

Manolis, A. S. and Manolis, A. A. (2017) ‘Transcatheter aortic valve implantation in nonagenarians: selectively feasible or extravagantly futile?’, *Annals of cardiothoracic surgery*. China, 6(5), pp. 524–531. doi: 10.21037/acs.2017.07.11.

Maragiannis, D. *et al.* (2014) ‘Interventional imaging: the role of echocardiography’, *Methodist DeBakey cardiovascular journal*. United States, 10(3), pp. 172–177. doi: 10.14797/mdcj-10-3-172.

Marchaux, S. *et al.* (2011) ‘Illustration of the echocardiographic diagnosis of subaortic membrane stenosis in adults: Surgical and live three-dimensional transoesophageal findings’, *European Journal of Echocardiography*. P. Ennezat, Cardiology Hospital, 59037 Lille Cedex, France, 12(1), p. E2. doi: 10.1093/ejechocard/jeq096.

Mariathas, M. *et al.* (2017) ‘Transcatheter aortic valve implantation: Where are we now?’, *Future Cardiology*. N. Curzen, Coronary Research Group, University Hospital Southampton NHS Foundation Trust, Southampton, United Kingdom, 13(6), pp. 551–566. doi: 10.2217/fca-2017-0056.

Markowicz, S. *et al.* (2014) ‘Gonococcal aneurysm of the ascending aorta: Case report and review of neisseria gonorrhoeae endovascular infections’, *Sexually Transmitted Diseases*. F. Jacobs, Departments of Infectious Diseases, Hôpital Erasme, Université Libre de Bruxelles, Brussels, Belgium, 41(2), pp. 111–113. doi: 10.1097/OLQ.0000000000000079.

Marquis-Gravel, G. *et al.* (2016) ‘Medical Treatment of Aortic Stenosis’, *Circulation*. P. Généreux, Cardiovascular Research Foundation, 1700 Broadway, 9th Floor, New York, NY, United States, 134(22), pp. 1766–1784. doi: 10.1161/CIRCULATIONAHA.116.023997.

Marthy, A. G. *et al.* (2015) ‘Impact of LVH by ECG voltage criteria on trans-catheter aortic valve replacement outcomes’, *Cardiology (Switzerland)*. A.G. Marthy, Albany Medical College, Albany, NY, United States, 131, p. 320. Available at: <http://www.embase.com/search/results?subaction=viewrecord&from=export&id=L71972338>.

Martin, G. P., Sperrin, M. and Mamas, M. A. (2018) ‘Pre-procedural risk models for patients undergoing transcatheter aortic valve implantation.’, *Journal of thoracic disease*. China, 10(Suppl 30), pp. S3560–S3567. doi: 10.21037/jtd.2018.05.67.

Martinez-Comendador, J. *et al.* (2017) ‘Sutureless aortic bioprosthesis.’, *Interactive cardiovascular and thoracic surgery*. England, 25(1), pp. 114–121. doi: 10.1093/icvts/ivx051.

Martinez-Diaz, J. L. (2008) ‘Valvular heart disease in pregnancy: a review of the literature.’, *Boletín de la Asociación Médica de Puerto Rico*. J.L. Martinez-Diaz, Cardiology Section, Department of Medicine, VA Caribbean Healthcare System, San Juan, Puerto Rico, USA., 100(4), pp. 55–59. Available at: http://www.embase.com/search/results?subaction=viewrecord&from=export&id=L354600708.

Martinez-Selles, M. *et al.* (2015) ‘Clinical significance of conduction disturbances after aortic valve intervention: current evidence.’, *Clinical research in cardiology : official journal of the German Cardiac Society*. Germany, 104(1), pp. 1–12. doi: 10.1007/s00392-014-0739-6.

Martin-Hernandez, P. *et al.* (2015) ‘[Experience of transcatheter aortic valve implantation in the Central Military Hospital].’, *Archivos de cardiologia de Mexico*. Mexico, 85(4), pp. 296–306. doi: 10.1016/j.acmx.2015.07.004.

Maskatia, S. *et al.* (2010) ‘Risk factors for long term-outomes in balloon valvuloplasty for aortic stenosis’, *Journal of the American College of Cardiology*. S. Maskatia, Section Pediatric Cardiology, Department of Pediatrics, Baylor College of Medicine, Houston, TX, United States, 55(10), p. A39.E374. doi: 10.1016/S0735-1097(10)60375-0.

Masson, J.-B. *et al.* (2009) ‘Transcatheter aortic valve implantation: review of the nature, management, and avoidance of procedural complications.’, *JACC. Cardiovascular interventions*. United States, 2(9), pp. 811–820. doi: 10.1016/j.jcin.2009.07.005.

Mathieu, P., Despres, J. P. and Pibarot, P. (2007) ‘The “valvulo-metabolic” risk in calcific aortic valve disease.’, *The Canadian journal of cardiology*. England, 23 Suppl B, pp. 32B-39B. doi: 10.1016/s0828-282x(07)71008-5.

Matsagas, M. I. *et al.* (2006) ‘Bilateral one-stage carotid endarterectomy combined with aortic valve replacement: A case report’, *Chirurgia*. C.E. Anagnostopoulos, Department of Cardiothoracic Surgery, University Hospital of Ioannina, University Avenue, 45500, Ioannina, Greece, 19(4), pp. 267–268. Available at: http://www.embase.com/search/results?subaction=viewrecord&from=export&id=L46177978.

Matthews, I. G. *et al.* (2011) ‘In patients undergoing aortic valve replacement, what factors predict the requirement for permanent pacemaker implantation?’, *Interactive cardiovascular and thoracic surgery*. England, 12(3), pp. 475–479. doi: 10.1510/icvts.2010.254607.

Maurovich-Horvat, P. *et al.* (2018) ‘Role of Multidetector Computed Tomography in Transcatheter Aortic Valve Implantation - From Pre-procedural Planning to Detection of Post-procedural Complications’, *Journal of Cardiovascular Emergencies*. P. Maurovich-Horvat, Cardiovascular Imaging Research Group, Heart and Vascular Center, Semmelweis University, Vaìrosmajor u. 68, Budapest, Hungary, 4(4), pp. 178–186. doi: 10.2478/jce-2018-0022.

Mavroudis, C., Backer, C. L. and Kaushal, S. (2009) ‘Aortic stenosis and aortic insufficiency in children: impact of valvuloplasty and modified Ross-Konno procedure.’, *Seminars in thoracic and cardiovascular surgery. Pediatric cardiac surgery annual*. United States, pp. 76–86. doi: 10.1053/j.pcsu.2009.01.008.

May Khan, A. & Kim, Y. (2014) ‘Aortic dilatation and aortopathies in congenital heart disease’, *Current Opinion in Cardiology*. Y. Kim, Philadelphia Adult Congenital Heart Center, Assistant Prof. of Medicine, Perelman Center for Advanced Medicine, 3400 Civic Center Boulevard, Philadelphia, PA 19104, United States, 29(1), pp. 91–96. doi: 10.1097/HCO.0000000000000029.

Mazine, A. *et al.* (2017) ‘Sutureless aortic valves: who is the right patient?’, *Current opinion in cardiology*. United States, 32(2), pp. 130–136. doi: 10.1097/HCO.0000000000000371.

McBrien, A. *et al.* (2012) ‘Single-centre experience of 101 paediatric and adult Ross procedures: Mid-term results’, *Interactive Cardiovascular and Thoracic Surgery*. A. McBrien, Department of Paediatric Cardiology, Freeman Hospital, Freeman Road, High Heaton, Newcastle-upon-Tyne NE7 7DN, United Kingdom, 14(5), pp. 570–574. doi: 10.1093/icvts/ivr149.

McCarthy, Cian P. *et al.* (2016) ‘When does asymptomatic aortic stenosis warrant surgery? Assessment techniques’, *Cleveland Clinic journal of medicine*. United States, 83(4), pp. 271–280. doi: 10.3949/ccjm.83a.15069.

McConkey, H. *et al.* (2017) ‘Adjuncts to transcatheter aortic valve implantation.’, *Expert review of cardiovascular therapy*. England, 15(5), pp. 357–365. doi: 10.1080/14779072.2017.1297230.

McHugh, F. *et al.* (2018) ‘Antithrombotic management after transcatheter aortic valve implantation.’, *Journal of thoracic disease*. China, 10(Suppl 30), pp. S3620–S3628. doi: 10.21037/jtd.2018.10.59.

Mehrpooya, M. *et al.* (2014) ‘Undiagnosed interrupted aortic arch in a 59-year-old male patient with severe aortic valve stenosis: A case report and literature review’, *ARYA Atherosclerosis*. M. Mehrpooya, Department of Cardiology, School of Medicine, Imam Khomeini Hospital, Tehran University of Medical Sciences, Tehran, Iran, 10(4), pp. 230–232. Available at: http://www.embase.com/search/results?subaction=viewrecord&from=export&id=L373598614.

Mehta, Y. and Singh, R. (2009) ‘Quantification of AS and AR.’, *Annals of cardiac anaesthesia*. India, 12(2), p. 166. doi: 10.4103/0971-9784.53437.

Meier, P., Franzen, O. and Lansky, A. J. (2013) ‘Almanac 2013: novel non-coronary cardiac interventions.’, *Heart (British Cardiac Society)*. England, 99(18), pp. 1309–1316. doi: 10.1136/heartjnl-2013-304430.

Meier, P. and Timmis, A. (2012) ‘Almanac 2012: interventional cardiology’, *Anadolu Kardiyoloji Dergisi/The Anatolian Journal of Cardiology*. doi: 10.5152/akd.2013.049.

Meier, P. & Timmise, A. (2013) ‘Almanac 2012: Interventional cardiology. The national society journals present selected research that has driven recent advances in clinical cardiology’, *Hellenic Journal of Cardiology*. P. Meier, The Heart Hospital, University College, London Hospitals, UCLH, 16-18 Westmoreland St, United Kingdom, 54(4), pp. 242–254. Available at: <http://www.embase.com/search/results?subaction=viewrecord&from=export&id=L369487417>.

Melby, S. J. *et al.* (2007) ‘Aortic Valve Replacement in Octogenarians: Risk Factors for Early and Late Mortality’, *Annals of Thoracic Surgery*. R.J. Damiano Jr., Division of Cardiothoracic Surgery, Department of Surgery, Washington University School of Medicine, St. Louis, MO, United States, 83(5), pp. 1651–1657. doi: 10.1016/j.athoracsur.2006.09.068.

Meller, S. M. *et al.* (2014) ‘Clinical implications for diffusion-weighted MRI brain lesions associated with transcatheter aortic valve replacement.’, *Catheterization and cardiovascular interventions : official journal of the Society for Cardiac Angiography & Interventions*. United States, 83(3), pp. 502–508. doi: 10.1002/ccd.24904.

Metaxa, S., Ioannou, A. and Missouris, C. G. (2017) ‘Transcatheter aortic valve implantation: new hope in the management of valvular heart disease.’, *Postgraduate medical journal*. England, 93(1099), pp. 280–288. doi: 10.1136/postgradmedj-2016-134554.

Meyer, S. R. *et al.* (2013) ‘Outcomes of consecutive patients referred for consideration for transcatheter aortic valve implantation from an encompassing health-care region.’, *The American journal of cardiology*. United States, 112(9), pp. 1450–1454. doi: 10.1016/j.amjcard.2013.06.038.

Meza, J. M. *et al.* (2018) ‘A novel, data-driven conceptualization for critical left heart obstruction’, *Computer Methods and Programs in Biomedicine*. B.W. McCrindle, 555 University Ave, Room 4432, Black Wing, Toronto, ON, Canada, 165, pp. 107–116. doi: 10.1016/j.cmpb.2018.08.014.

Miceli, A. (2017) ‘Sutureless valve associated a minimally invasive approach covers the gap between transcatheter aortic valve implantation and conventional aortic valve replacement’, *Cardiology (Switzerland)*. A. Miceli, Istituto Clinico sant’Ambrogio, Gruppo Ospedaliero San Donato, Milano, Italy, 137, p. 112. doi: 10.1159/000477751.

Minh, T. H. *et al.* (2014) ‘Expanding the indication for sutureless aortic valve replacement to patients with mitral disease.’, *The Journal of thoracic and cardiovascular surgery*. United States, 148(4), pp. 1354–1359. doi: 10.1016/j.jtcvs.2013.12.061.

Miyamoto, T. *et al.* (2006) ‘Twenty years experience of surgical aortic valvotomy for critical aortic stenosis in early infancy.’, *European journal of cardio-thoracic surgery : official journal of the European Association for Cardio-thoracic Surgery*. Germany, 30(1), pp. 35–40. doi: 10.1016/j.ejcts.2006.03.050.

Moghbeli, N.; Pare, E. & Webb, G. (2008) ‘Practical assessment of maternal cardiovascular risk in pregnancy’, *Congenital Heart Disease*. G. Webb, Cardiovascular Division, University of Pennsylvania, Philadelphia, PA, United States, 3(5), pp. 308–316. doi: 10.1111/j.1747-0803.2008.00207.x.

Mohammed Imran, G. and Alexandra, L. (2018) ‘Understanding Neurologic Complications Following TAVR.’, *Interventional cardiology (London, England)*. England, 13(1), pp. 27–32. doi: 10.15420/icr.2017:25:1.

Mohammed, S. *et al.* (2018) ‘Antithrombotic Therapy for Transcatheter Valvular Interventions: A Revisit.’, *Current vascular pharmacology*. United Arab Emirates, 16(2), pp. 107–113. doi: 10.2174/1570161115666170406105753.

Moini, C. *et al.* (2016) ‘[Cardiac permanent pacemaker after transcatheter aortic valve implantation: A predictive and scientific review].’, *Annales de cardiologie et d’angeiologie*. France, 65(5), pp. 346–351. doi: 10.1016/j.ancard.2016.09.003.

Mokin, M. *et al.* (2017) ‘Transcatheter aortic valve replacement: perioperative stroke and beyond’, *Expert Review of Neurotherapeutics*. A.H. Siddiqui, University at Buffalo, Neurosurgery, 100 High Street, Suite B4, Buffalo, NY, United States, 17(4), pp. 327–334. doi: 10.1080/14737175.2017.1253475.

Monteiro, J. P. *et al.* (2017) ‘Trifecta Bioprothesis for Aortic Valve Replacement: Our Experience.’, *Revista portuguesa de cirurgia cardio-toracica e vascular : orgao oficial da Sociedade Portuguesa de Cirurgia Cardio-Toracica e Vascular*. Portugal, 24(3–4), p. 157.

Moore, M. *et al.* (2017) ‘The economic value of rapid deployment aortic valve replacement via full sternotomy.’, *Journal of comparative effectiveness research*. England, 6(4), pp. 293–302. doi: 10.2217/cer-2016-0064.

Moraca, R. J. *et al.* (2018) ‘Combined carotid endarterectomy and transcatheter aortic valve replacement: Technique and outcomes.’, *Journal of cardiac surgery*. United States, 33(5), pp. 265–269. doi: 10.1111/jocs.13601.

Morita, K. & Kurosawa, H. (2001) ‘Indications for and clinical outcome of the Ross procedure: a review’, *Nippon Geka Gakkai zasshi*. K. Morita, Jikei University School of Medicine, Tokyo., 102(4), pp. 330–336. Available at: <http://www.embase.com/search/results?subaction=viewrecord&from=export&id=L33468118>.

Morris, R. J., Samuels, L. E. and Brockman, S. K. (1998) ‘Total simultaneous repair of coarctation and intracardiac pathology in adult patients.’, *The Annals of thoracic surgery*. Netherlands, 65(6), pp. 1698–1702. doi: 10.1016/s0003-4975(98)00291-4.

Moura, L. M. *et al.* (2007) ‘New understanding about calcific aortic stenosis and opportunities for pharmacologic intervention’, *Current Opinion in Cardiology*. N.M. Rajamannan, Northwestern University Feinberg School of Medicine, 303 E Chicago, Tarry 12-717, Chicago, IL 60611, United States, 22(6), pp. 572–577. doi: 10.1097/HCO.0b013e3282f0dae6.

Muhammad, K. I. and Tokarchik, G. C. (2018) ‘Transcaval transcatheter aortic valve replacement: a visual case review.’, *Journal of visualized surgery*. China, p. 102. doi: 10.21037/jovs.2018.04.02.

Mukdad, L. *et al.* (2019) ‘The Incidence of Dysphagia Among Patients Undergoing TAVR With Either General Anesthesia or Moderate Sedation.’, *Journal of cardiothoracic and vascular anesthesia*. United States, 33(1), pp. 45–50. doi: 10.1053/j.jvca.2018.05.040.

Muralidharan, R. *et al.* (2011) ‘The challenges with brain death determination in adult patients on extracorporeal membrane oxygenation’, *Neurocritical Care*. R. Muralidharan, Department of Neurology, Mayo Clinic, 200 First Street SW, Rochester, MN 55905, United States, 14(3), pp. 423–426. doi: 10.1007/s12028-011-9516-9.

Murashita, T. *et al.* (2014) ‘Aortic valve replacement for severe aortic valve stenosis in the nonagenarian patient’, *The Annals of thoracic surgery*. Netherlands, 98(5), pp. 1593–1597. doi: 10.1016/j.athoracsur.2014.06.015.

Murtaza, G. *et al.* (2014) ‘Predictors of mortality in inoperable patients undergoing transcatheter aortic valve implantation for severe aortic valve stenosis’, *Innovations: Technology and Techniques in Cardiothoracic and Vascular Surgery*. G. Murtaza, Medical College of Wisconsin, Milwaukee, WI, United States, 9(3), p. 225. doi: 10.1097/IMI.0000000000000069.

Murtuza, B. *et al.* (2008) ‘Does minimal-access aortic valve replacement reduce the incidence of postoperative atrial fibrillation?’, *Texas Heart Institute Journal*. B. Murtuza, Department of Cardiothoracic Surgery, St. Mary’s Hospital, London W2 1NY, United Kingdom, United States, 35(4), pp. 428–438. Available at: <http://www.embase.com/search/results?subaction=viewrecord&from=export&id=L354120786>.

Myasoedova, V. A. *et al.* (2018) ‘Novel pharmacological targets for calcific aortic valve disease: Prevention and treatments’, *Pharmacological Research*. V.A. Myasoedova, Unit for the Study of Aortic, Valvular, and Coronary Pathologies, Centro Cardiologico Monzino IRCCS, Via Carlo Parea 4, Milan, Italy, Netherlands, 136, pp. 74–82. doi: 10.1016/j.phrs.2018.08.020.

Mylotte, D. *et al.* (2014) ‘Adoption of Transcatheter Aortic Valve Implantation in Western Europe.’, *Interventional cardiology (London, England)*. England, 9(1), pp. 37–40. doi: 10.15420/icr.2011.9.1.37.

Nagula, P. *et al.* (2017) ‘“Dissecting ascending aortic aneurysm manifesting as superior vena cava syndrome and pulmonary thromboembolism in a patient with former aortic valve replacement — A rare case report”’, *IHJ Cardiovascular Case Reports (CVCR)*. P. Nagula, CARE Hospitals, Banjara Hills, Hyderabad, India, 1(3), pp. 125–128. doi: 10.1016/j.ihjccr.2017.09.002.

Naoum, C. *et al.* (2016) ‘Clinical Outcomes and Imaging Findings in Women Undergoing TAVR.’, *JACC. Cardiovascular imaging*. United States, 9(4), pp. 483–493. doi: 10.1016/j.jcmg.2016.02.009.

Naqvi, T.Z. (2018) ‘Echocardiography in transcatheter aortic valve implantation—Part 1—Transthoracic echocardiography’, *Echocardiography*. T.Z. Naqvi, Echocardiographic Laboratory, Department of Cardiology, Mayo Clinic, Scottsdale, AZ, United States, 35(7), pp. 1005–1019. doi: 10.1111/echo.13799.

Natorska, J. *et al.* (2016) ‘Increased bleeding risk in patients with aortic valvular stenosis: From new mechanisms to new therapies’, *Thrombosis Research*. J. Natorska, Institute of Cardiology, Jagiellonian University School of Medicine, 80 Pradnicka St., Krakow, Poland, United States, 139, pp. 85–89. doi: 10.1016/j.thromres.2016.01.016.

Navarra, E. *et al.* (2016) ‘Endovascular resection of the native aortic valve before transcatheter aortic valve implantation: State of the art and review’, *European Journal of Cardio-thoracic Surgery*. E. Navarra, Saint-Luc Hospital, Cardiovascular and Thoracic Surgery Department, Catholic University of Louvain, Avenue Hippocrate 10/6107, Brussels, Belgium, Germany, 50(3), pp. 406–410. doi: 10.1093/ejcts/ezw027.

Negi, S. I. *et al.* (2014) ‘Retrieval of ruptured valves and their accessories during transcatheter aortic valve replacement.’, *Journal of cardiac surgery*. United States, 29(2), pp. 209–212. doi: 10.1111/jocs.12285.

Nelson, J. A. *et al.* (2012) ‘Who is being referred for TAVR?: Patterns of Referral for TAVR at a single academic medical center’, *Circulation*. J.A. Nelson, Cardiology, Duke Univ, Durham, NC, United States, 126(21). Available at: <http://www.embase.com/search/results?subaction=viewrecord&from=export&id=L70958644>.

Nemes, A.; Forster, T. & Csanády, M. (2004) ‘Coronary flow reserve in patients with aortic valve stenosis - Review of the literature and own observations’, *Polski Przeglad Kardiologiczny*. A. Nemes, 2nd Dept. of Medicine/Cardiology Ctr, Albert Szent-Gyorgyi Med./Pharm. Ctr, University of Szeged, Koranyi fasor 6, H-6720 Szeged, Hungary, 6(2), pp. 203–207. Available at: <http://www.embase.com/search/results?subaction=viewrecord&from=export&id=L39419854>.

Neragi-Miandoab, S. and Michler, R. E. (2013) ‘A review of most relevant complications of transcatheter aortic valve implantation.’, *ISRN cardiology*. Egypt, 2013, p. 956252. doi: 10.1155/2013/956252.

Neragi-Miandoab, S. *et al.* (2012) ‘Recently patented and widely used valves for transcatheter aortic valve implantation’, *Recent Patents on Cardiovascular Drug Discovery*. S. Neragi-Miandoab, Montefiore Medical Center, Department of Cardiovascular and Thoracic Surgery, Albert Einstein College of Medicine, 3400 Bainbridge Ave, MAP 5, New York, NY 10467, United States, United Arab Emirates, 7(3), pp. 196–205. doi: 10.2174/157489012803832810.

Neragi-Miandoab, S. *et al.* (2013) ‘Recently patented transcatheter aortic valves in clinical trials.’, *Recent patents on cardiovascular drug discovery*. United Arab Emirates, 8(3), pp. 186–191.

Neragi-Miandoab, S. *et al.* (2015) ‘Prosthetic valve endocarditis five months following transcatheter aortic valve implantation and review of literature.’, *The heart surgery forum*. United States, 18(1), pp. E20-2. doi: 10.1532/hsf.1221.

Nerla, R. *et al.* (2017) ‘Repositionable and retrievable Lotus Valve System for the treatment of bicuspid aortic stenosis: is it time to treat all valves?’, *Expert review of medical devices*. England, 14(7), pp. 557–563. doi: 10.1080/17434440.2017.1344095.

Neuburger, P. J. *et al.* (2019) ‘Operational and Institutional Recommendations and Requirements for TAVR: A Review of Expert Consensus and the Impact on Health Care Policy.’, *Journal of cardiothoracic and vascular anesthesia*. United States, 33(6), pp. 1731–1741. doi: 10.1053/j.jvca.2019.01.062.

Neuburger, P. J. and Patel, P. A. (2017) ‘Anesthetic Techniques in Transcatheter Aortic Valve Replacement and the Evolving Role of the Anesthesiologist.’, *Journal of cardiothoracic and vascular anesthesia*. United States, 31(6), pp. 2175–2182. doi: 10.1053/j.jvca.2017.03.033.

Neuburger, P. J. *et al.* (2016) ‘A Practical Approach to Managing Transcatheter Aortic Valve Replacement With Sedation.’, *Seminars in cardiothoracic and vascular anesthesia*. United States, 20(2), pp. 147–157. doi: 10.1177/1089253215625111.

Ngo, D. T.; Sverdlov, A. L. & Horowitz, J. D. (2012) ‘Prevention of aortic valve stenosis: A realistic therapeutic target?’, *Pharmacology and Therapeutics*. J.D. Horowitz, Department of Cardiology, Queen Elizabeth Hospital, University of Adelaide, 28 Woodville Road, Woodville, SA 5011, Australia, 135(1), pp. 78–93. doi: 10.1016/j.pharmthera.2012.04.001.

Nguyen, G. and Leipsic, J. (2013) ‘Cardiac computed tomography and computed tomography angiography in the evaluation of patients prior to transcatheter aortic valve implantation.’, *Current opinion in cardiology*. United States, 28(5), pp. 497–504. doi: 10.1097/HCO.0b013e32836245c1.

Nguyen, T. C. *et al.* (2013) ‘Impact of varying degrees of renal dysfunction on transcatheter and surgical aortic valve replacement.’, *The Journal of thoracic and cardiovascular surgery*. United States, 146(6), pp. 1397–1399. doi: 10.1016/j.jtcvs.2013.07.065.

Nicolini, F. *et al.* (2014) ‘The evolution of cardiovascular surgery in elderly patient: A review of current options and outcomes’, *BioMed Research International*. F. Nicolini, Unità di Cardiochirurgia, Dipartimento di Medicina Clinica e Sperimentale, Università Degli Studi di Parma, Via A. Gramsci 14, 43126 Parma, Italy, 2014. doi: 10.1155/2014/736298.

Nicolini, F. *et al.* (2010) ‘Aortic valve replacement in octogenarians: analysis of risk factors for early and late mortality.’, *The Journal of heart valve disease*. England, 19(5), pp. 615–622.

Nijenhuis, V. J. *et al.* (2019) ‘Antithrombotic therapy in patients undergoing transcatheter aortic valve implantation’, *Heart*. J.M. Ten Berg, Department of Cardiology, St Antonius Hospital, Nieuwegein, Netherlands, 105(10), pp. 742–748. doi: 10.1136/heartjnl-2018-314313.

Nir, R.-R. and Bolotin, G. (2017) ‘[TECHNOLOGICAL SOLUTIONS FOR THE IMPROVEMENT OF OUTCOMES IN CARDIAC SURGERY OF OCTOGENARIAN PATIENTS].’, *Harefuah*. Israel, 156(9), pp. 600–603.

Nistri, S. *et al.* (2008) ‘Practical echocardiography in aortic valve stenosis’, *Journal of Cardiovascular Medicine*. S. Nistri, Servizio di Cardiologia, CMSR Veneto Medica, Via Vicenza, 204, 360777 Altavilla Vicentina (VI), Italy, 9(7), pp. 653–665. doi: 10.2459/JCM.0b013e3282f27d49.

Noble, S. & Ibrahim, R. (2009) ‘Percutaneous interventions in adults with congenital heart disease: Expanding indications and opportunities’, *Current Cardiology Reports*. R. Ibrahim, Adult Congenital Heart Center, Montreal Heart Institute, 5000 Belanger Street East, Montreal, QC H1T 1C8, Canada, 11(4), pp. 306–313. doi: 10.1007/s11886-009-0044-6.

Noguchi, K. *et al.* (1991) ‘Supravalvular aortic stenosis syndrome with advanced mitral regurgitation requiring extended aortoplasty and mitral valve replacement: a case report’, *Kyobu geka. The Japanese journal of thoracic surgery*. K. Noguchi, Department of Thoracic and Cardiovascular Surgery, Kyorin University School of Medicine., Japan, 44(4), pp. 351–354. Available at: <http://www.embase.com/search/results?subaction=viewrecord&from=export&id=L21827629>.

Nombela-Franco, L. *et al.* (2013) ‘Role of balloon postdilation following trancatheter aortic valve implantation.’, *Minerva cardioangiologica*. Italy, 61(5), pp. 499–512.

Nombela-Franco, L. *et al.* (2014) ‘Significant mitral regurgitation left untreated at the time of aortic valve replacement: a comprehensive review of a frequent entity in the transcatheter aortic valve replacement era.’, *Journal of the American College of Cardiology*. United States, 63(24), pp. 2643–2658. doi: 10.1016/j.jacc.2014.02.573.

Noorani, A., Radia, R. and Bapat, V. (2015) ‘Challenges in valve-in-valve therapy.’, *Journal of thoracic disease*. China, 7(9), pp. 1501–1508. doi: 10.3978/j.issn.2072-1439.2015.06.16.

Nqayana, T.; Moodley, J. & Naidoo, D. P. (2008) ‘Cardiac disease in pregnancy’, *Cardiovascular Journal of Africa*. T. Nqayana, Department of Obstetrics and Gynaecology, Nelson R. Mandela School of Medicine, University of KwaZulu-Natal, Durban, 19(3), pp. 145–151. Available at: http://www.embase.com/search/results?subaction=viewrecord&from=export&id=L352079340.

Nugteren, L. B. *et al.* (2010) ‘Critical review of health-related quality of life studies of patients with aortic stenosis.’, *The Journal of cardiovascular nursing*. L.B. Nugteren, Intensive Care Unit, Mercy Hospital, Coon Rapids, Minnesota 55433, USA., United States, 25(1), pp. 25–39. doi: 10.1097/JCN.0b013e3181b99828.

O. F. M., Sellevold *et al.* (2010) ‘Transcutaneous aortic valve implantation: Recent advances and future’, *Current Opinion in Anaesthesiology*. O. F. M. Sellevold, St Olavs University Hospital, Hans Nissens gt 3, Trondheim, Norway, United States, 23(1), pp. 67–73. doi: 10.1097/ACO.0b013e3283346c07.

Oakley, L. *et al.* (2017) ‘Development and Early Experience of the First Joint Military Health System-Veterans Affairs Transcatheter Aortic Valve Replacement Program’, *Military medicine*, 182(11), pp. e2036–e2040. doi: 10.7205/MILMED-D-16-00398.

Obadia, J. F. *et al.* (1991) ‘[Colonic angiodysplasia with chronic digestive hemorrhage cured after valvular replacement for aortic valve stenosis].’, *Archives des maladies du coeur et des vaisseaux*. France, 84(4), pp. 569–572.

O’Byrne, M. L. and Gillespie, M. J. (2014) ‘Will catheter interventions replace surgery for valve abnormalities?’, *Current opinion in cardiology*. United States, 29(1), pp. 83–90. doi: 10.1097/HCO.0000000000000026.

Oh, J. K. and Zorn, G. L. 3rd (2016) ‘Prosthesis-Patient Mismatch: Another Reason for TAVR?’, *JACC. Cardiovascular imaging*. United States, pp. 934–936. doi: 10.1016/j.jcmg.2015.12.016.

Ohno, Y., Tamburino, C. and Barbanti, M. (2015) ‘Transcatheter Aortic Valve Implantation Experience with SAPIEN 3.’, *Minerva cardioangiologica*. Italy, 63(3), pp. 205–216.

Olasinska-Wisniewska, A. *et al.* (2011) ‘Transcatheter aortic valve implantation: The new option for high-risk patients with aortic stenosis’, *Cardiology Journal*. A. Olasińska-Wiśniewska, Department of Cardiology, Poznan University of Medical Sciences, ul. Długa 1/2, 61-848 Poznań, Poland, Poland, 18(4), pp. 461–468. Available at: <http://www.embase.com/search/results?subaction=viewrecord&from=export&id=L362183882>.

O’Malley, R. G., Mahaffey, K. W. and Fearon, W. F. (2017) ‘Adjuvant Antithrombotic Therapy in TAVR.’, *Current cardiology reports*. United States, 19(5), p. 41. doi: 10.1007/s11886-017-0850-1.

Omer, S. *et al.* (2009) ‘Outcomes of aortic valve replacement performed by residents in octogenarians.’, *The Journal of surgical research*. United States, 156(1), pp. 139–144. doi: 10.1016/j.jss.2009.03.045.

Ong, G. *et al.* (2018) ‘Paravalvular Regurgitation After Transcatheter Aortic Valve Replacement: Is the Problem Solved?’, *Interventional cardiology clinics*. Netherlands, 7(4), pp. 445–458. doi: 10.1016/j.iccl.2018.06.005.

Onishi, T. *et al.* (2018) ‘The role of echocardiography in transcatheter aortic valve implantation.’, *Cardiovascular diagnosis and therapy*. China (Republic : 1949- ), 8(1), pp. 3–17. doi: 10.21037/cdt.2018.01.06.

Opp, D. *et al.* (2019) ‘Congenital aortic valve stenosis in a newborn female with 45, X/47, XYY mixed gonadal dysgenesis’, *World Journal for Pediatric and Congenital Heart Surgery*. D. Opp, Mayo Clinic, United States, 10(2), p. NP23. doi: 10.1177/2150135119830271.

O’Sullivan, C. J. and Wenaweser, P. (2015) ‘Optimizing clinical outcomes of transcatheter aortic valve implantation patients  with comorbidities.’, *Expert review of cardiovascular therapy*. England, 13(12), pp. 1419–1432. doi: 10.1586/14779072.2015.1102056.

O’Sullivan, C. J. *et al.* (2014) ‘Coronary revascularization and TAVI: Before, during, after or never?’, *Minerva Medica*. P. Wenaweser, Department of Cardiology, Swiss Cardiovascular Center Bern, Bern University Hospital, Bern, Switzerland, Italy, 105(6), pp. 475–485. Available at: <http://www.embase.com/search/results?subaction=viewrecord&from=export&id=L603548568>.

O'Sullivan, K. E. *et al.* (2016) ‘Advances in the management of severe aortic stenosis’, *Irish Journal of Medical Science*. K.E. O’Sullivan, Department of Cardiothoracic Surgery, Mater Private Hospital, Eccles St., Dublin 7, Ireland, Ireland, 185(2), pp. 309–317. doi: 10.1007/s11845-016-1417-7.

Ota, T. *et al.* (2016) ‘Value of mitral and tricuspid annular displacement to assess the interventricular systolic relationship in severe aortic valve stenosis : A Pilot study’, *European Heart Journal Cardiovascular Imaging*. T. Ota, Shonan Kamakura General Hospital, Anesthesiology, Kamakura, Japan, 17, p. ii122. doi: 10.1093/ehjci/jew250.

Oteo, J. A. *et al.* (2006) ‘[Endocarditis due to Bartonella spp. Three new clinical cases and Spanish literature review].’, *Enfermedades infecciosas y microbiologia clinica*. Spain, 24(5), pp. 297–301.

Otto, C. M. and C.M., O. (2010) ‘Calcific Aortic Valve Disease: New Concepts’, *Seminars in Thoracic and Cardiovascular Surgery*. C.M. Otto, Cardiology Fellowship Programs, Division of Cardiology, University of Washington, Box 356422, Seattle, WA 98195, United States, United States, 22(4), pp. 276–284. doi: 10.1053/j.semtcvs.2011.01.009.

Oudart, J.-B. *et al.* (2011) ‘[Hyperamylasemia after cardiac surgery: which significance?].’, *Annales de biologie clinique*. France, 69(2), pp. 223–227. doi: 10.1684/abc.2011.0528.

Overtchouk, P., Prendergast, B. and Modine, T. (2019) ‘Why should we extend transcatheter aortic valve implantation to low-risk patients? A comprehensive review.’, *Archives of cardiovascular diseases*. Netherlands, 112(5), pp. 354–362. doi: 10.1016/j.acvd.2019.03.004.

Ozier, D. *et al.* (2017) ‘Timing of Conduction Abnormalities Leading to Permanent Pacemaker Insertion After Transcatheter Aortic Valve Implantation-A Single-Centre Review.’, *The Canadian journal of cardiology*. England, 33(12), pp. 1660–1667. doi: 10.1016/j.cjca.2017.08.012.

Ozkan, A. *et al.* (2011) ‘Assessment of left ventricular function in aortic stenosis.’, *Nature reviews. Cardiology*. England, 8(9), pp. 494–501. doi: 10.1038/nrcardio.2011.80.

P.-H., Huang *et al.* (2013) ‘transcatheter aortic valve replacement using the edwards SAPIEN transcatheter heart valves’, *Cardiology Clinics*. A.C. Eisenhauer, Division of Cardiovascular Medicine, Brigham and Women’s Hospital, Harvard Medical School, 75 Francis Street, Boston, MA 02115, United States, Netherlands, 31(3), pp. 337–350. doi: 10.1016/j.ccl.2013.05.009.

P.T.L., Chiam *et al.* (2013) ‘An update on complications associated with transcatheter aortic valve implantation: Stroke, paravalvular leak, atrioventricular block and perforation’, *Future Cardiology*. P.T.L. Chiam, Department of Cardiology, National Heart Centre Singapore, 17 Third Hospital Avenue, Mistri Wing, Singapore 168752, Singapore, England, 9(5), pp. 733–747. doi: 10.2217/FCA.13.43.

Pacheco, C. *et al.* (2018) ‘Transcatheter Aortic Valve Replacement for Severe Aortic Stenosis in Women: Clinical Characteristics and Outcomes.’, *The Canadian journal of cardiology*. England, 34(4), pp. 422–428. doi: 10.1016/j.cjca.2017.10.026.

Pagel, P. S. *et al.* (2016) ‘Moderate Aortic Valvular Insufficiency Invalidates Vortex Formation Time as an Index of Left Ventricular Filling Efficiency in Patients With Severe Degenerative Calcific Aortic Stenosis Undergoing Aortic Valve Replacement’, *Journal of Cardiothoracic and Vascular Anesthesia*. P.S. Pagel, Zablocki Veterans Affairs Medical Center, Anesthesia Service, 5000 W. National Avenue, Milwaukee, United States, United States, 30(5), pp. 1260–1265. doi: 10.1053/j.jvca.2016.03.144.

Pagel, P. S. *et al.* (2013) ‘Chronic pressure-overload hypertrophy attenuates vortex formation time in patients with severe aortic stenosis and preserved left ventricular systolic function undergoing aortic valve replacement’, *Journal of Cardiothoracic and Vascular Anesthesia*. P.S. Pagel, Anesthesia Service, Clement J. Zablocki Veterans Affairs Medical Center, 5000W, National Avenue, Milwaukee, WI 53295, United States, United States, 27(4), pp. 660–664. doi: 10.1053/j.jvca.2013.01.007.

Pagel, P. S. *et al.* (2014) ‘Aortic valve replacement reduces valvuloarterial impedance but does not affect systemic arterial compliance in elderly men with degenerative calcific trileaflet aortic valve stenosis’, *Journal of Cardiothoracic and Vascular Anesthesia*. P.S. Pagel, Anesthesia Service, Clement J. Zablocki Veterans Affairs Medical Center, 5000 W. National Avenue, Milwaukee, WI, United States, United States, 28(6), pp. 1540–1544. doi: 10.1053/j.jvca.2014.05.017.

Pagnesi, M. *et al.* (2017) ‘Is Transcatheter Aortic Valve Replacement Superior to Surgical Aortic Valve Replacement?: A Meta-Analysis of Randomized Controlled Trials.’, *JACC. Cardiovascular interventions*. United States, pp. 1899–1901. doi: 10.1016/j.jcin.2017.06.025.

Panaich, S. S. *et al.* (2018) ‘Case-Based Discussion Regarding Challenges in Patient Selection and Procedural Planning in Left Atrial Appendage Occlusion’, *Mayo Clinic Proceedings*. D.R. Holmes, Department of Cardiovascular Diseases, Mayo Clinic, 200 First St SW, Rochester, MN, United States, 93(5), pp. 630–638. doi: 10.1016/j.mayocp.2018.03.015.

Panayiotides, I. M. and Nikolaides, E. (2014) ‘Transcatheter Aortic Valve Implantation (TAVI): Is it Time for This Intervention  to be Applied in a Lower Risk Population?’, *Clinical Medicine Insights. Cardiology*. United States, 8, pp. 93–102. doi: 10.4137/CMC.S19217.

Panchal, H. B. *et al.* (2016) ‘Mortality and major adverse cardiovascular events after transcatheter aortic valve replacement using Edwards valve versus CoreValve: A meta-analysis.’, *Cardiovascular revascularization medicine : including molecular interventions*. United States, 17(1), pp. 24–33. doi: 10.1016/j.carrev.2015.11.005.

Panchal, H. B. (2013) ‘Improvement of valvular parameters following transcatheter aoritc valve implantation versus surgical aortic valve replacement in patients with severe aortic stenosis: A meta-analysis’, *Circulation*. H.B. Panchal, Dept of Internal Medicine, East Tennessee State Univ, Johnson City, TN, United States, 128(22). Available at: http://www.embase.com/search/results?subaction=viewrecord&from=export&id=L71339835.

Paradis, J. M. *et al.* (2013) ‘Evolution of definitions and understanding of vascular complications related to transcatheter aortic valve replacement.’, *Minerva cardioangiologica*. Italy, 61(5), pp. 513–528.

Paradis, J.-M., Altisent, O. A.-J. and RodEs-Cabau, J. (2015) ‘Reducing periprocedural complications in transcatheter aortic valve replacement:  review of paravalvular leaks, stroke and vascular complications.’, *Expert review of cardiovascular therapy*. England, 13(11), pp. 1251–1262. doi: 10.1586/14779072.2015.1096778.

Paradis, J.-M. *et al.* (2015) ‘Transcatheter Valve-in-Valve and Valve-in-Ring for Treating Aortic and Mitral Surgical Prosthetic Dysfunction.’, *Journal of the American College of Cardiology*. United States, 66(18), pp. 2019–2037. doi: 10.1016/j.jacc.2015.09.015.

Paradis, J.-M. *et al.* (2014) ‘Aortic stenosis and coronary artery disease: What do we know?What don’t we know? A comprehensive review of the literature with proposed treatment algorithms’, *European Heart Journal*. J.-M. Paradis, Quebec Heart and Lung Institute, 2725 Chemin Sainte-Foy, Quebec, QC, G1V 4G5, Canada, England, 35(31), pp. 2069-2082b. doi: 10.1093/eurheartj/ehu247.

Paradis, J.-M., Labbe, B. and Rodes-Cabau, J. (2015) ‘Coronary artery disease and transcatheter aortic valve replacement: current treatment paradigms.’, *Coronary artery disease*. England, 26(3), pp. 272–278. doi: 10.1097/MCA.0000000000000210.

Parker, D. J. (1984) ‘The patient after cardiac valve surgery--risks and complications.’, *European heart journal*. England, 5 Suppl A, pp. 141–145. doi: 10.1093/eurheartj/5.suppl_a.141.

Parolari, A. *et al.* (2009) ‘Nonrheumatic calcific aortic stenosis: an overview from basic science to pharmacological prevention.’, *European journal of cardio-thoracic surgery : official journal of the European Association for Cardio-thoracic Surgery*. Germany, 35(3), pp. 493–504. doi: 10.1016/j.ejcts.2008.11.033.

Patel, A. D. *et al.* (2018) ‘Neo Left Main Channel Creation Using Double Stenting Alongside a Sapien 3 Aortic Valve Bioprosthesis for Left Main Coronary Obstruction Following Valve-in-Valve Transcatheter Aortic Valve Replacement: A Case Report With Review of Literature.’, *Journal of investigative medicine high impact case reports*. United States, 6, p. 2324709618767696. doi: 10.1177/2324709618767696.

Patel, H. J. *et al.* (2013) ‘Aortic valve replacement: using a statewide cardiac surgical database identifies  a procedural volume hinge point.’, *The Annals of thoracic surgery*. Netherlands, 96(5), pp. 1560–1566. doi: 10.1016/j.athoracsur.2013.05.103.

Patel, J. H., Mathew, S. T. and Hennebry, T. A. (2009) ‘Transcatheter aortic valve replacement: a potential option for the nonsurgical patient.’, *Clinical cardiology*. United States, 32(6), pp. 296–301. doi: 10.1002/clc.20535.

Patel, J. S. *et al.* (2016) ‘Access Options for Transcatheter Aortic Valve Replacement in Patients with Unfavorable Aortoiliofemoral Anatomy.’, *Current cardiology reports*. United States, 18(11), p. 110. doi: 10.1007/s11886-016-0788-8.

Patel, J. K. *et al.* (2012) ‘Managing arrhythmias before and after aortic valve surgery in children’, *American Journal of Cardiovascular Drugs*. V.R. Iyer, Childrens Hospital of Philadelphia, 34th Street and Civic Center Boulevard, Philadelphia, PA 19104, United States, New Zealand, 12(1), pp. 23–34. doi: 10.2165/11596350-000000000-00000.

Pawade, T. A., Newby, D. E. and Dweck, M. R. (2015) ‘Calcification in Aortic Stenosis: The Skeleton Key.’, *Journal of the American College of Cardiology*. United States, 66(5), pp. 561–577. doi: 10.1016/j.jacc.2015.05.066.

Pedersen, M.W. *et al.* (2018) ‘Clinical and pathophysiological aspects of bicuspid aortic valve disease’, *Cardiology in the Young*. N.H. Andersen, Department of Cardiology, Aalborg University Hospital, Aalborg, Denmark, England, 29(1), pp. 1–10. doi: 10.1017/S1047951118001658.

Perez, S., Thielhelm, T. P. and Cohen, M. G. (2018) ‘To revascularize or not before transcatheter aortic valve implantation?’, *Journal of thoracic disease*. China, 10(Suppl 30), pp. S3578–S3587. doi: 10.21037/jtd.2018.09.85.

Pericas, J. M. *et al.* (2015) ‘Infective endocarditis in patients with an implanted transcatheter aortic valve: Clinical characteristics and outcome of a new entity’, *Journal of Infection*. J.M. Miro, Infectious Diseases Service, Hospital Clínic, Villarroel 170, Barcelona, Spain, 70(6), pp. 565–576. doi: 10.1016/j.jinf.2014.12.013.

Perlman, G. Y. *et al.* (2017) ‘Transcatheter aortic valve replacement with the Portico valve: One-year results of the early Canadian experience’, *EuroIntervention*. J.G. Webb, St. Paul’s Hospital, 1081 Burrard St, Vancouver, BC, Canada, France, 12(13), pp. 1653–1659. doi: 10.4244/EIJ-D-16-00299.

Perrin, N., Frei, A. and Noble, S. (2018) ‘Transcatheter aortic valve implantation: Update in 2018.’, *European journal of internal medicine*. Netherlands, 55, pp. 12–19. doi: 10.1016/j.ejim.2018.07.002.

Pettet, J. K. *et al.* (2014) ‘Comparison of pulmonary complications in patients undergoing transcatheter aortic valve implantation versus open aortic valve replacement.’, *Journal of cardiothoracic and vascular anesthesia*. United States, 28(3), pp. 497–501. doi: 10.1053/j.jvca.2013.04.006.

Pfeiffer, S. *et al.* (2017) ‘The “entangled” stent: a preventable cause of paravalvular leak of the Perceval bioprosthesis.’, *Interactive cardiovascular and thoracic surgery*. England, 25(6), pp. 987–989. doi: 10.1093/icvts/ivx239.

Pflederer, T. *et al.* (2010) ‘Aortic valve stenosis: CT contributions to diagnosis and therapy’, *Journal of Cardiovascular Computed Tomography*. T. Pflederer, Department of Internal Medicine 2 (Cardiology), University of Erlangen, Ulmenweg 18, D-91054 Erlangen, Germany, United States, 4(6), pp. 355–364. doi: 10.1016/j.jcct.2010.09.008.

Philip, F. *et al.* (2015) ‘Aortic annulus and root characteristics in severe aortic stenosis due to bicuspid aortic valve and tricuspid aortic valves: implications for transcatheter aortic valve therapies.’, *Catheterization and cardiovascular interventions : official journal of the Society for Cardiac Angiography & Interventions*. United States, 86(2), pp. E88-98. doi: 10.1002/ccd.25948.

Phillips, D. (2006) ‘Aortic stenosis: A review.’, *AANA journal*. United States, 74(4), pp. 309–315.Piazza, N. *et al.* (2011) ‘Transcatheter aortic valve implantation for failing surgical aortic bioprosthetic valve: from concept to clinical application and evaluation (part 2).’, *JACC. Cardiovascular interventions*. United States, 4(7), pp. 733–742. doi: 10.1016/j.jcin.2011.05.007.

Piazza, N. *et al.* (2011) ‘Transcatheter aortic valve implantation for failing surgical aortic bioprosthetic valve: from concept to clinical application and evaluation (part 1).’, *JACC. Cardiovascular interventions*. United States, 4(7), pp. 721–732. doi: 10.1016/j.jcin.2011.03.016.

Pibarot, P. and Dumesnil, J. G. (2000) ‘Hemodynamic and clinical impact of prosthesis-patient mismatch in the aortic valve position and its prevention.’, *Journal of the American College of Cardiology*. United States, 36(4), pp. 1131–1141. doi: 10.1016/s0735-1097(00)00859-7.

Pibarot, P. and Dumesnil, J. G. (2011) ‘Prosthesis-patient mismatch’, *Aswan Heart Centre Science and Practice Series*. P. Pibarot, Research Group in Valvular Heart Disease, Quebec Heart and Lung Institute, 2725 Chemin Sainte-Foy, Québec, QC, G1V-4G5, Canada, 2011(1). doi: 10.5339/ahcsps.2011.7.

Pibarot, P. *et al.* (2015) ‘Assessment of paravalvular regurgitation following TAVR: a proposal of unifying grading scheme.’, *JACC. Cardiovascular imaging*. United States, 8(3), pp. 340–360. doi: 10.1016/j.jcmg.2015.01.008.

Pibarot, P. *et al.* (2019) ‘Moderate Aortic Stenosis and Heart Failure With Reduced Ejection Fraction: Can Imaging Guide Us to Therapy?’, *JACC. Cardiovascular imaging*. United States, 12(1), pp. 172–184. doi: 10.1016/j.jcmg.2018.10.021.

Pieper, P. G., Balci, A. and Van Dijk, A. P. (2008) ‘Pregnancy in women with prosthetic heart valves.’, *Netherlands heart journal : monthly journal of the Netherlands Society of Cardiology and the Netherlands Heart Foundation*. Netherlands, 16(12), pp. 406–411. doi: 10.1007/bf03086187.

Pighi, M. *et al.* (2014) ‘TAVI: New trials and registries offer further welcome evidence - U.S. CoreValve,  CHOICE, and GARY.’, *Global cardiology science & practice*. Qatar, 2014(1), pp. 78–87. doi: 10.5339/gcsp.2014.12.

Pineda, A. M. & Kiefer, T. L. (2018) ‘Asymptomatic Severe Aortic Valve Stenosis—When to Intervene: a Review of the Literature, Current Trials, and Guidelines’, *Current Cardiology Reports*. A.M. Pineda, Division of Cardiology - ACC Building 5th floor, University of Florida College of Medicine – Jacksonville, 655 West 8th Street, Jacksonville, FL, United States, 20(12). doi: 10.1007/s11886-018-1072-x.

Pineda, A. M. *et al.* (2017) ‘Outcomes of transcatheter aortic valve replacement in high or extreme risk patients with severe bicuspid aortic valve stenosis’, *Circulation*. A.M. Pineda, Dept of Cardiology, Duke Univ Med Cntr, Durham, NC, United States, 136. Available at: <http://www.embase.com/search/results?subaction=viewrecord&from=export&id=L619983299>.

Pires de Morais, G. *et al.* (2011) ‘[Multislice computed tomography in the selection of candidates for transcatheter  aortic valve implantation].’, *Revista portuguesa de cardiologia : orgao oficial da Sociedade Portuguesa de Cardiologia = Portuguese journal of cardiology : an official journal of the Portuguese Society of Cardiology*. Portugal, 30(9), pp. 717–726. doi: 10.1016/S0870-2551(11)70015-4.

Pislaru, S. V *et al.* (2015) ‘Aortic Stenosis and Noncardiac Surgery: Managing the Risk.’, *Current problems in cardiology*. United States, 40(11), pp. 483–503. doi: 10.1016/j.cpcardiol.2015.06.003.

Pislaru, S. V, Nkomo, V. T. and Sandhu, G. S. (2016) ‘Assessment of Prosthetic Valve Function After TAVR.’, *JACC. Cardiovascular imaging*. United States, 9(2), pp. 193–206. doi: 10.1016/j.jcmg.2015.11.010.

Poels, T. T. *et al.* (2014) ‘Transcatheter aortic valve implantation-induced left bundle branch block: Causes and consequences’, *Journal of Cardiovascular Translational Research*. T.T. Poels, Department of Cardiothoracic Surgery, Maastricht University Medical Center, Postbus 5800, 6202 AZ Maastricht, Netherlands, United States, 7(4), pp. 395–405. doi: 10.1007/s12265-014-9560-x.

Poels, T. T. *et al.* (2015) ‘Severely thrombosed transcatheter aortic valve 9 months after implantation’, *Annals of Thoracic Surgery*. T.T. Poels, Maastricht University Medical Center, Department of Cardiothoracic Surgery, Postbus 5800, Maastricht, Netherlands, 100(4), pp. 1441–1444. doi: 10.1016/j.athoracsur.2014.12.052.

Poirier, P. & Eckel, R. H. (2008) ‘Cardiovascular consequences of obesity’, *Drug Discovery Today: Therapeutic Strategies*. P. Poirier, Institut universitaire de cardiologie et de pneumologie, Laval Hospital, 2725 Chemin Sainte-Foy, Sainte-Foy, Que. G1V 4G5, Canada, 5(1), pp. 45–51. doi: 10.1016/j.ddstr.2008.05.005.

Pollak, P. M. *et al.* (2014) ‘Quality, economics, and national guidelines for transcatheter aortic valve replacement’, *Progress in Cardiovascular Diseases*. P.M. Pollak, Mayo Clinic, 200 First Street SW, Rochester, MN 55905, United States, United States, 56(6), pp. 610–618. doi: 10.1016/j.pcad.2014.03.001.

Polsani, V. and Little, S. H. (2013) ‘Assessment of prosthetic valve function and para-valvular regurgitation after trans-catheter aortic valve replacement.’, *Current opinion in cardiology*. United States, 28(5), pp. 518–523. doi: 10.1097/HCO.0b013e3283632a3d.

Poltak, J. M. *et al.* (2015) ‘Paravertebral analgesia in transapical transcatheter aortic valve replacement.’, *Heart, lung and vessels*. Italy, 7(3), pp. 217–223.

Poulin, A. *et al.* (2015) ‘Management of Coronary Disease in the Era of Transcatheter Aortic Valve Replacement: Comprehensive Review of the Literature’, *Interventional Cardiology Clinics*. J.-M. Paradis, 2725, Chemin Sainte-Foy, Québec, QC, Canada, Netherlands, 4(1), pp. 13–21. doi: 10.1016/j.iccl.2014.09.003.

Pozzoli, A. *et al.* (2019) ‘Clinical Outcomes in Patients with Severe Aortic Valve Stenosis Treated with a Portico Transcatheter Aortic Valve System’, *Surgical technology international*, 34, pp. 331–338. Available at: <http://www.embase.com/search/results?subaction=viewrecord&from=export&id=L628022329>.

Prabhu, W. *et al.* (2014) ‘Transcatheter aortic valve replacement: a review of current indications and outcomes’, *Rhode Island medical journal (2013)*. United States, 97(2), pp. 38–41. Available at: <http://www.embase.com/search/results?subaction=viewrecord&from=export&id=L605896528>.

Pratt, A. K., Shah, N. S. and Boyce, S. W. (2014) ‘Left ventricular assist device management in the ICU.’, *Critical care medicine*. United States, 42(1), pp. 158–168. doi: 10.1097/01.ccm.0000435675.91305.76.

Praz, F. and Nietlispach, F. (2013) ‘Cerebral protection devices for transcatheter aortic valve implantation: is better the enemy of good?’, *EuroIntervention : journal of EuroPCR in collaboration with the Working Group on  Interventional Cardiology of the European Society of Cardiology*. France, 9 Suppl, pp. S124-8. doi: 10.4244/EIJV9SSA26.

Praz, F. *et al.* (2017) ‘Latest evidence on transcatheter aortic valve implantation vs. surgical aortic valve replacement for the treatment of aortic stenosis in high and intermediate-risk patients.’, *Current opinion in cardiology*. United States, 32(2), pp. 117–122. doi: 10.1097/HCO.0000000000000379.

Praz, F. *et al.* (2015) ‘Expanding Indications of Transcatheter Heart Valve Interventions’, *JACC: Cardiovascular Interventions*. P. Wenaweser, Department of Cardiology, Swiss Cardiovascular Center Bern, Bern University Hospital, Bern, Switzerland, United States, 8(14), pp. 1777–1796. doi: 10.1016/j.jcin.2015.08.015.

Probst, V. *et al.* (2011) ‘Systematic familial screening identified a high frequency of heritability in calcific aortic valve disease’, *European Heart Journal*. V. Probst, University Hospital of Nantes, Hospital Guillaume and Rene Laennec, Nantes, France, 32, p. 776. doi: 10.1093/eurheartj/ehr324.

Puri, R. *et al.* (2016) ‘TAVI or No TAVI: identifying patients unlikely to benefit from transcatheter aortic valve implantation.’, *European heart journal*. England, 37(28), pp. 2217–2225. doi: 10.1093/eurheartj/ehv756.

Quader, M. A. *et al.* (2006) ‘Aortic valve repair for congenital abnormalities of the aortic valve.’, *Heart, lung & circulation*. Australia, 15(4), pp. 248–255. doi: 10.1016/j.hlc.2006.05.005.

Rader, F. *et al.* (2015) ‘Left ventricular hypertrophy in valvular aortic stenosis: Mechanisms and clinical implications’, *American Journal of Medicine*. F. Rader, Cedars-Sinai Medical Center, 127 S San Vincente Blvd, Los Angeles, CA, United States, United States, 128(4), pp. 344–352. doi: 10.1016/j.amjmed.2014.10.054.

Raffa, G. M. *et al.* (2012) ‘Aortic valve replacement for paraprosthetic leak after transcatheter implantation.’, *Journal of cardiac surgery*. United States, 27(1), pp. 47–51. doi: 10.1111/j.1540-8191.2011.01351.x.

Ragosta, M. (2013) ‘Multi-modality imaging of the aortic valve in the era of transcatheter aortic valve replacement: a guide for patient selection, valve selection, and valve delivery.’, *Journal of cardiovascular translational research*. United States, 6(5), pp. 665–674. doi: 10.1007/s12265-013-9490-z.

Rahimtoola, S. H. *et al.* (2010) ‘Statins for calcific aortic valve stenosis: Into oblivion after SALTIRE and SEAS? An extensive review from bench to bedside’, *Current problems in cardiology*. H. Hermans, United States, 35(6), pp. 284–306. doi: 10.1016/j.cpcardiol.2010.02.002.

Raiten, J. M. *et al.* (2013) ‘Critical care management of patients following transcatheter aortic valve replacement.’, *F1000Research*. England, 2, p. 62. doi: 10.12688/f1000research.2-62.v1.

Raja, S. G. & Pozzi, M. (2004) ‘Ross operation in children and young adults: The Alder Hey case series’, *BMC Cardiovascular Disorders*. S.G. Raja, Dept. Paediatr. Cardiothoracic Surg., Alder Hey Hospital, Eaton Road, Liverpool L12 2AP, United Kingdom, 4. doi: 10.1186/1471-2261-4-3.

Rajamannan, N. M. (2009) ‘Calcific aortic stenosis: lessons learned from experimental and clinical studies.’, *Arteriosclerosis, thrombosis, and vascular biology*. United States, 29(2), pp. 162–168. doi: 10.1161/ATVBAHA.107.156752.

Rajamannan, N.M. *et al.* (2007) ‘Calcific aortic stenosis: An update’, *Nature Clinical Practice Cardiovascular Medicine*. S.H. Rahimtoola, University of Southern California, Los Angeles, CA, United States, England, 4(5), pp. 254–262. doi: 10.1038/ncpcardio0827.

Rajamannan, N. M. (2011) ‘Bicuspid aortic valve disease: The role of oxidative stress in Lrp5 bone formation’, *Cardiovascular Pathology*. N. M. Rajamannan, Division of Cardiology, Northwestern University, Feinberg School of Medicine, Chicago, IL 60611, United States, 20(3), pp. 168–176. doi: 10.1016/j.carpath.2010.11.007.

Rajamannan, N. M. (2007) ‘Reassessment of statins to retard the progression of aortic stenosis’, *Current Cardiology Reports*. N.M. Rajamannan, Division of Cardiology, Department of Medicine, Northwestern University Medical School, 300 E. Superior, Tarry 12-717, Chicago, IL 60611, United States, 9(2), pp. 99–104. doi: 10.1007/BF02938335.

Rajamannan, N. M. (2004) ‘Is it time for medical therapy for aortic valve disease?’, *Expert Review of Cardiovascular Therapy*. N.M. Rajamannan, NW Univ. Feinberg School of Medicine, Feinberg Cardiovascular Res. Inst., 303 E Chicago Avenue, Chicago, IL 60611, United States, 2(6), pp. 845–854. doi: 10.1586/14779072.2.6.845.

Rajani, R. *et al.* (2013) ‘An insight into transcatheter aortic valve implantation - A perspective from multidetector-computed tomography’, *Catheterization and Cardiovascular Interventions*. R. Rajani, Department of Cardiac Computed Tomography, St Thomas’ Hospital, Westminster Bridge Road, London SE1 7EH, United Kingdom, United States, 82(7), pp. E952–E958. doi: 10.1002/ccd.24624.

Rajani, R., Hancock, J. and Chambers, J. B. (2012) ‘The art of assessing aortic stenosis.’, *Heart (British Cardiac Society)*. England, 98 Suppl 4, pp. iv14-22. doi: 10.1136/heartjnl-2012-302392.

Raju, S. *et al.* (2016) ‘Vascular complications and procedures following trans catheter aortic valve implantation (TAVI)’, *Journal of Vascular Surgery*. S. Raju, University of Toronto, Toronto, ON, Canada, 63(6), p. 177S. doi: 10.1016/j.jvs.2016.03.299.

Ralph-Edwards, A. C. *et al.* (1999) ‘Valve surgery in octogenarians’, *Canadian Journal of Cardiology*. A.C. Ralph-Edwards, Division of Cardiovascular Surgery, Toronto General Hospital, 200 Elizabeth Street, Toronto, Ont. M5G 2C4, Canada, England, 15(10), pp. 1113–1119. Available at: <http://www.embase.com/search/results?subaction=viewrecord&from=export&id=L30071730>.

Ram, P. *et al.* (2017) ‘Acute kidney injury post-transcatheter aortic valve replacement.’, *Clinical cardiology*. United States, 40(12), pp. 1357–1362. doi: 10.1002/clc.22820.

Ramakrishna, H. *et al.* (2017) ‘The Year in Cardiothoracic and Vascular Anesthesia: Selected Highlights From 2016.’, *Journal of cardiothoracic and vascular anesthesia*. United States, 31(1), pp. 1–13. doi: 10.1053/j.jvca.2016.10.009.

Ramakrishna, H. *et al.* (2014) ‘Incidental moderate mitral regurgitation in patients undergoing aortic valve replacement for aortic stenosis: Review of guidelines and current evidence’, *Journal of Cardiothoracic and Vascular Anesthesia*. J.G.T. Augoustides, Department of Anesthesiology and Critical Care, Perelman School of Medicine, University of Pennsylvania, 3400 Spruce Street, Philadelphia, PA, United States, United States, 28(2), pp. 417–422. doi: 10.1053/j.jvca.2013.11.003.

Ramakrishna, H. *et al.* (2016) ‘Transcatheter Aortic Valve Replacement: Clinical Update on Access Approaches in the Contemporary Era.’, *Journal of cardiothoracic and vascular anesthesia*. United States, 30(5), pp. 1425–1429. doi: 10.1053/j.jvca.2016.03.006.

Ramlawi, B. *et al.* (2012) ‘Transcatheter aortic valve replacement (TAVR): access planning and strategies.’, *Methodist DeBakey cardiovascular journal*. B. Ramlawi, Methodist DeBakey Heart & Vascular Center, The Methodist Hospital, Houston, Texas, USA., United States, 8(2), pp. 22–25. Available at: <http://www.embase.com/search/results?subaction=viewrecord&from=export&id=L366359439>.

Ranasinghe, M. P. *et al.* (2019) ‘Thromboembolic and bleeding complications in transcatheter aortic valve implantation: Insights on mechanisms, prophylaxis and therapy’, *Journal of Clinical Medicine*. K. Peter, Baker Heart and Diabetes Institute, 75 Commercial Road, PO Box, 6492, Melbourne, VIC, Australia, Switzerland, 8(2). doi: 10.3390/jcm8020280.

Rashid, H. N. *et al.* (2018) ‘Subclinical Leaflet Thrombosis in Transcatheter Aortic Valve Replacement Detected by Multidetector Computed Tomography- A Review of Current Evidence.’, *Circulation journal : official journal of the Japanese Circulation Society*. Japan, 82(7), pp. 1735–1742. doi: 10.1253/circj.CJ-17-1363.

Ravindra, V. M. *et al.* (2016) ‘Carotid Artery Stenosis in the Setting of Transcatheter Aortic Valve Replacement: Clinical and Technical Considerations of Carotid Stenting.’, *World neurosurgery*. United States, 86, pp. 194–198. doi: 10.1016/j.wneu.2015.09.063.

Rayner, J. *et al.* (2014) ‘Aortic valve disease.’, *International journal of clinical practice*. England, 68(10), pp. 1209–1215. doi: 10.1111/ijcp.12471.

Rea, C. W. *et al.* (2019) ‘Characteristics and Outcomes of Patients With Severe Aortic Stenosis Discussed by the Multidisciplinary “Heart Team” According to Treatment Allocation’, *Heart Lung and Circulation*. P.N. Ruygrok, Green Lane Cardiovascular Service, Auckland City Hospital, Private Bag 92024, Auckland, New Zealand. doi: 10.1016/j.hlc.2019.02.192.

Rehman, H.; Kleiman, N. S. & Kalra, A. (2018) ‘Transcatheter valvular interventions in 2017: Some things old, some things new!’, *Current Opinion in Cardiology*. A. Kalra, Division of Cardiovascular Medicine, Department of Medicine, University Hospitals Cleveland Medical Center, 11100 Euclid Ave, Cleveland, OH, United States, 33(4), pp. 363–368. doi: 10.1097/HCO.0000000000000531.

Reineke, D. *et al.* (2016) ‘Mechanical versus biological aortic valve replacement strategies.’, *Expert review of cardiovascular therapy*. England, 14(4), pp. 423–430. doi: 10.1586/14779072.2016.1133293.

Rengier, F. *et al.* (2014) ‘State-of-the-art aortic imaging: Part II - applications in transcatheter aortic valve replacement and endovascular aortic aneurysm repair’, *Vasa - Journal of Vascular Diseases*. S. Partovi, Radiology, University Hospitals Case Medical Center, Case Western Reserve University, 11100 Euclid Ave, 44124 Cleveland OH, United States, Switzerland, 43(1), pp. 6–26. doi: 10.1024/0301-1526/a000324.

Reustle, A. & Torzewski, M. (2018) ‘Role of p38 MAPK in atherosclerosis and aortic valve sclerosis’, *International Journal of Molecular Sciences*. M. Torzewski, Department of Laboratory Medicine and Hospital Hygiene, Robert Bosch-Hospital, Stuttgart, Germany, 19(12). doi: 10.3390/ijms19123761.

Rex, S. and S., R. (2013) ‘Anesthesia for transcatheter aortic valve implantation: An update’, *Current Opinion in Anaesthesiology*. S. Rex, Department of Anesthesiology, University Hospitals Leuven, Herestraat 49, B-3000 Leuven, Belgium, United States, 26(4), pp. 456–466. doi: 10.1097/ACO.0b013e3283628d1e.

Rezq, A. *et al.* (2014) ‘Comprehensive multidisciplinary patient assessment and selection before TAVI procedure’, *Minerva Cardioangiologica*. C. Godino, Cardio-Thoracic-Vascular Department, San Raffaele Institute, Milan, Italy, Italy, 62(2), pp. 177–191. Available at: <http://www.embase.com/search/results?subaction=viewrecord&from=export&id=L373005275>.

Rheude, T. *et al.* (2018) ‘Spotlight on the SAPIEN 3 transcatheter heart valve’, *Medical Devices: Evidence and Research*. O. Husser, Department of Cardiology, St-Johannes-Hospital, Johannesstraße 9-13, Dortmund, Germany, 11, pp. 353–360. doi: 10.2147/MDER.S143897.

Riem Vis, P. W. *et al.* (2012) ‘The pathophysiological basis of pharmacological interventions in CAVD’, *Netherlands Heart Journal*. P. W. Riem Vis, Department of Cardiothoracic Surgery, University Medical Center Utrecht, PO BOX 85500, E03.511, 3508GA Utrecht, Netherlands, 20(6), pp. 270–278. doi: 10.1007/s12471-011-0230-1.

Riha, H. *et al.* (2013) ‘Major themes for 2012 in cardiovascular anesthesia and intensive care’, *HSR Proceedings in Intensive Care and Cardiovascular Anesthesia*. J.G.T. Augoustides, Cardiovascular and Thoracic Section, Department of Anesthesiology and Critical Care, University of Pennsylvania, Dulles 680, HUP 3400 Spruce Street, Philadelphia, PA, 19104-4283, United States, 5(1), pp. 9–16. Available at: <http://www.embase.com/search/results?subaction=viewrecord&from=export&id=L368868214>.

Roberts, W. C. (1983) ‘Morphologic features of the normal and abnormal mitral valve.’, *The American journal of cardiology*. United States, 51(6), pp. 1005–1028. doi: 10.1016/s0002-9149(83)80181-7.

Roberts, W. C. *et al.* (2012) ‘Cardiac transplantation in adults with aortic valve disease with focus on the bicuspid aortic valve’, *American Journal of Cardiology*. W.C. Roberts, Department of Internal Medicine (Division of Cardiology), Baylor University Medical Center, Dallas, TX, United States, United States, 109(8), pp. 1212–1214. doi: 10.1016/j.amjcard.2011.12.002.

Roger, V. L. (1995) ‘Left ventricular function in aortic stenosis: A clinical review’, *Journal of Heart Valve Disease*. V.L. Roger, Division of Cardiovascular Diseases, Mayo Clinic, 200 First Street SW, Rochester, MN 55905, United States, 4(SUPPL. 2), pp. S230–S235. Available at: http://www.embase.com/search/results?subaction=viewrecord&from=export&id=L25331919.

Rogers, T. and Waksman, R. (2016) ‘Role of CMR in TAVR.’, *JACC. Cardiovascular imaging*. United States, 9(5), pp. 593–602. doi: 10.1016/j.jcmg.2016.01.011.

Roifman, I.; Glikstein, R. S. & Rudski, L. (2009) ‘Spontaneous Cerebral Embolism in a Forty Year Old Man: Case Report and Review of the Literature’, *Journal of the American Society of Echocardiography*. I. Roifman, Division of Cardiology, Department of Medicine, McGill University Health Center, Montreal, Que., Canada, 22(10), pp. 1197.e1-1197.e3. doi: 10.1016/j.echo.2009.04.010.

Rose, A. G. (1986) ‘Etiology of acquired valvular heart disease in adults. A survey of 18,132 autopsies and 100 consecutive valve-replacement operations.’, *Archives of pathology & laboratory medicine*. United States, 110(5), pp. 385–388.

Ross, D. and Yacoub, M. H. (1969) ‘Homograft replacement of the aortic valve. A critical review.’, *Progress in cardiovascular diseases*. United States, 11(4), pp. 275–293.

Rosseel, L., De Backer, O. and Sondergaard, L. (2019) ‘Clinical Valve Thrombosis and Subclinical Leaflet Thrombosis Following Transcatheter Aortic Valve Replacement: Is There a Need for a Patient- Tailored Antithrombotic Therapy?’, *Frontiers in cardiovascular medicine*. Switzerland, 6, p. 44. doi: 10.3389/fcvm.2019.00044.

Rossow, C. F. and McCabe, J. M. (2016) ‘Protection from Cerebral Embolic Events During Transcatheter Aortic Valve Replacement.’, *Current cardiology reports*. United States, 18(2), p. 16. doi: 10.1007/s11886-015-0692-7.

Rotman, O. M. *et al.* (2018) ‘Principles of TAVR valve design, modelling, and testing.’, *Expert review of medical devices*. England, 15(11), pp. 771–791. doi: 10.1080/17434440.2018.1536427.

Routledge, H. C. *et al.* (2007) ‘Percutaneous aortic valve replacement: new hope for inoperable and high-risk patients.’, *The Journal of invasive cardiology*. United States, 19(11), pp. 478–483.

Roychoudhury, D. *et al.* (2003) ‘Culture-negative suppurative endocarditis causing severe mitral valve obstruction: Complementary use of transesophageal and transthoracic echocardiography’, *Echocardiography*. D. Roychoudhury, Division of Cardiology, Department of Medicine, NY Hospital Medical Center of Queens, 56-45 Main Street, Flushing, NY 11355, United States, 20(5), pp. 429–434. doi: 10.1046/j.1540-8175.2003.03080.x.

Rozeik, M., Wheatley, D. and Gourlay, T. (2014) ‘The aortic valve: structure, complications and implications for transcatheter aortic valve replacement.’, *Perfusion*. England, pp. 285–300. doi: 10.1177/0267659114521650.

Ruel, M. and Labinaz, M. (2010) ‘Transcatheter aortic-valve replacement: a cardiac surgeon and cardiologist team perspective.’, *Current opinion in cardiology*. United States, 25(2), pp. 107–113. doi: 10.1097/HCO.0b013e328335fff4.

Ruggeri, L. *et al.* (2012) ‘Anesthetic management for percutaneous aortic valve implantation: an overview of  worldwide experiences.’, *HSR proceedings in intensive care & cardiovascular anesthesia*. Italy, 4(1), pp. 40–46.

Ruparelia, N. (2018) ‘Oral Anticoagulant Therapy for Early Post-TAVI Thrombosis.’, *Interventional cardiology (London, England)*. England, 13(1), pp. 33–36. doi: 10.15420/icr.2017:14:1.

Ruparelia, N. and Prendergast, B. D. (2015) ‘Transcatheter aortic valve implantation - what the general physician needs to know.’, *Clinical medicine (London, England)*. England, 15(5), pp. 420–425. doi: 10.7861/clinmedicine.15-5-420.

Ruparelia, N. and Prendergast, B. D. (2015) ‘Indications for transcatheter aortic valve implantation--now and next?’, *Minimally invasive therapy & allied technologies : MITAT : official journal of the Society for Minimally Invasive Therapy*. England, 24(5), pp. 264–273. doi: 10.3109/13645706.2015.1031680.

Russo, E. *et al.* (2019) ‘Rate and Predictors of Permanent Pacemaker Implantation After Transcatheter Aortic Valve Implantation: Current Status.’, *Current cardiology reviews*. United Arab Emirates, 15(3), pp. 205–218. doi: 10.2174/1573403X15666181205105821.

Ruygrok, P. N. *et al.* (1993) ‘Aortic valve replacement in the elderly.’, *The Journal of heart valve disease*. P.N. Ruygrok, Department of Cardiology, Green Lane Hospital, Auckland, New Zealand., England, 2(5), pp. 550–557. Available at: <http://www.embase.com/search/results?subaction=viewrecord&from=export&id=L24856192>.

S.-C., Y. *et al.* (2008) ‘Risk of complications during pregnancy in women with congenital aortic stenosis’, *International Journal of Cardiology*. S.-C. Yap, Department of Cardiology, Erasmus MC, Rotterdam, Netherlands, 126(2), pp. 240–246. doi: 10.1016/j.ijcard.2007.03.134.

S.-H., K. (2017) ‘Recent advances in pediatric interventional cardiology’, *Korean Journal of Pediatrics*. S.-H. Kim, Department of Pediatrics, Sejong General Hospital, 28, 489-gil, Hohyeon-ro, Sosa-gu, Bucheon, South Korea, 60(8), pp. 237–244. doi: 10.3345/kjp.2017.60.8.237.

S.-H., M. and M., S. (2004) ‘Mid-term results of the Ross operation’, *Archives of Iranian Medicine*. S.-H. Mirkhani, Department of Cardiovascular Surgery, Iman Khomeini Hospital, Keshavarz Bolvd., Tehran, Iran, 7(2), pp. 104–107. Available at: <http://www.embase.com/search/results?subaction=viewrecord&from=export&id=L39162280>.

Saam, T. *et al.* (2008) ‘[Assessment of aortic stenosis after aortic valve replacement: comparative evaluation of dual-source CT and echocardiography].’, *RoFo : Fortschritte auf dem Gebiete der Rontgenstrahlen und der Nuklearmedizin*. Germany, 180(6), pp. 553–560. doi: 10.1055/s-2008-1027288.

Sabet, H. Y. *et al.* (1999) ‘Congenitally bicuspid aortic valves: A surgical pathology study of 542 cases (1991 through 1996) and a literature review of 2,715 additional cases’, *Mayo Clinic Proceedings*. W.D. Edwards, Division of Anatomic Pathology, Mayo Clinic Rochester, 200 First Street SW, Rochester, MN 55905, United States, 74(1), pp. 14–26. doi: 10.4065/74.1.14.

Saeed, S. *et al.* (2018) ‘Managing complications of hypertension in aortic valve stenosis patients’, *Expert Review of Cardiovascular Therapy*. E. Gerdts, Department of Clinical Science, University of Bergen, P.O. Box 7804, Bergen, Norway, England, 16(12), pp. 897–907. doi: 10.1080/14779072.2018.1535899.

Safarova, M. S. *et al.* (2015) ‘Transcatheter treatment of degenerative critical aortic valve stenosis in a patient with severe heart failure and chronic lymphocytic leukemia’, *Kardiologiia*. Russia (Federation), 55(1), pp. 82–87. Available at: <http://www.embase.com/search/results?subaction=viewrecord&from=export&id=L609386060>.

Saia, F. *et al.* (2011) ‘Incidence, prognostic value and management of vascular complications with transfemoral transcatheter aortic valve implantation’, *Future Cardiology*. F. Saia, Institute of Cardiology, University of Bologna, Policlinico S Orsola-Malpighi, Via Massarenti, 40138 Bologna, Italy, England, 7(3), pp. 321–331. doi: 10.2217/fca.11.16.

Saji, M. & Lim, D. S. (2016) ‘Transcatheter Aortic Valve Replacement in Lower Surgical Risk Patients: Review of Major Trials and Future Perspectives’, *Current Cardiology Reports*. D.S. Lim, Advanced Cardiac Valve Center, Department of Medicine, Division of Cardiovascular Medicine, University of Virginia, 1215 Lee Street, Hospital Expansion, Room 4033, Charlottesville, VA, United States, 18(10). doi: 10.1007/s11886-016-0772-3.

Sakaguchi, H., Kawata, T. and Taniguchi, S. (2004) ‘Concomitant mitral valve repair and left ventricular reconstruction in a patient  with chronic ischemic mitral regurgitation and inferior left ventricular aneurysm.’, *The Japanese journal of thoracic and cardiovascular surgery : official publication of the Japanese Association for Thoracic Surgery = Nihon Kyobu Geka Gakkai zasshi*. Japan, 52(2), pp. 71–74. doi: 10.1007/s11748-004-0087-y.

Sakamoto, Y. and Hashimoto, K. (2006) ‘[Avoidance of patient-prosthesis mismatch].’, *Kyobu geka. The Japanese journal of thoracic surgery*. Japan, 59(4), pp. 262–268.

Salaun, E. *et al.* (2018) ‘Bioprosthetic aortic valve durability in the era of transcatheter aortic valve implantation.’, *Heart (British Cardiac Society)*. England, 104(16), pp. 1323–1332. doi: 10.1136/heartjnl-2017-311582.

Salgado, R. A. *et al.* (2014) ‘Preprocedural CT evaluation of transcatheter aortic valve replacement: what the radiologist needs to know’, *Radiographics : a review publication of the Radiological Society of North America, Inc*. United States, 34(6), pp. 1491–1514. doi: 10.1148/rg.346125076.

Salinas, P., Moreno, R. and Lopez-Sendon, J. L. (2011) ‘Transcatheter aortic valve implantation: Current status and future perspectives.’, *World journal of cardiology*. United States, 3(6), pp. 177–185. doi: 10.4330/wjc.v3.i6.177.

Sannino, A. and Grayburn, P. A. (2018) ‘Mitral regurgitation in patients with severe aortic stenosis: diagnosis and management.’, *Heart (British Cardiac Society)*. England, 104(1), pp. 16–22. doi: 10.1136/heartjnl-2017-311552.

Sannino, A. *et al.* (2017) ‘Meta-Analysis of Effect of Body Mass Index on Outcomes After Transcatheter Aortic Valve Implantation.’, *The American journal of cardiology*. United States, 119(2), pp. 308–316. doi: 10.1016/j.amjcard.2016.09.031.

Santarpino, G. *et al.* (2018) ‘Sutureless aortic valve replacement vs. Transcatheter aortic valve implantation: A review of a single center experience’, *Minerva Cardioangiologica*. G. Santarpino, GVM Care and Research, Città di Lecce Hospital, Lecce, Italy, Italy, 66(2), pp. 160–162. doi: 10.23736/S0026-4725.17.04549-2.

Sardar, M. R. *et al.* (2017) ‘Management strategies and possible risk factors for ventricular septal defects after transcatheter aortic valve replacement: Case series from a single center and review of literature.’, *Cardiovascular revascularization medicine : including molecular interventions*. United States, 18(6), pp. 462–470. doi: 10.1016/j.carrev.2017.04.003.

Sardar, M. R. *et al.* (2017) ‘Vascular complications associated with transcatheter aortic valve replacement’, *Vascular Medicine (United Kingdom)*. H.D. Aronow, Warren Alpert Medical School, Brown University, Lifespan Cardiovascular Institute, 593 Eddy Street, RIH APC 730, Providence, RI, United States, 22(3), pp. 234–244. doi: 10.1177/1358863X17697832.

Sareyyupoglu, B. *et al.* (2009) ‘Management of mild aortic stenosis at the time of coronary artery bypass surgery: should the valve be replaced?’, *The Annals of thoracic surgery*. Netherlands, 88(4), pp. 1224–1231. doi: 10.1016/j.athoracsur.2009.05.085.

Sarkar, K. *et al.* (2015) ‘Core valve implant failure in the presence of mechanical mitral prosthesis: Importance of assessing left ventricular outflow tract’, *Catheterization and Cardiovascular Interventions*. G.P. Ussia, Department of Cardiology, Policlinico Tor Vergata, University of Rome, Tor Vergata, Viale Oxford, 81, Rome, Italy, United States, 85(5), pp. 920–924. doi: 10.1002/ccd.25376.

Sathyamurthy, I. & Jayanthi, K. (2016) ‘Asymptomatic severe aortic stenosis with normal left ventricular function – A review’, *Indian Heart Journal*. I. Sathyamurthy, Apollo Main Hospitals, Chennai, No: 21, Greams Lane, Off Greams Road, Chennai, India, 68(4), pp. 576–580. doi: 10.1016/j.ihj.2016.05.006.

Savoj, J. *et al.* (2019) ‘Transcatheter Double Valve-in-Valve Replacement of Aortic and Mitral Bioprosthetic Valves.’, *Cardiology research*. Canada, pp. 193–198. doi: 10.14740/cr863.

Sawa, Y. (2015) ‘Transcatheter aortic valve implantation’, *Surgery Today*. Y. Sawa, Department of Cardiovascular Surgery, Osaka University Graduate School of Medicine, 2-2 Yamada-Oka, Suita, Osaka, Japan, Japan, 45(5), pp. 527–536. doi: 10.1007/s00595-014-0902-8.

Sawaya, F. *et al.* (2012) ‘Aortic stenosis: a contemporary review.’, *The American journal of the medical sciences*. United States, 343(6), pp. 490–496. doi: 10.1097/MAJ.0b013e3182539d70.

Saxon, J. T. *et al.* (2018) ‘Bioprosthetic Valve Fracture During Valve-in-valve TAVR: Bench to Bedside.’, *Interventional cardiology (London, England)*. England, 13(1), pp. 20–26. doi: 10.15420/icr.2017:29:1.

Saxon, J. T. *et al.* (2019) ‘Complications of Bioprosthetic Valve Fracture as an Adjunct to Valve-in-Valve TAVR’, *Structural Heart*. J.T. Saxon, Saint Luke’s Mid America Heart Institute, 4330 Wornall Road, Suite 2000, Kansas City, MO, United States, 3(2), pp. 92–99. doi: 10.1080/24748706.2019.1578446.

Schäfer , U. *et al.* (2015) ‘Symetis ACURATE TAVI: Review of the technology, developments and current data with this self-expanding transcatheter heart valve’, *Minerva Cardioangiologica*. U. Schäfer, Division of Cardiology, University Heart Center Eppendorf, Martinistrasse 52, Hamburg, Germany, 63(5), pp. 359–369. Available at: <http://www.embase.com/search/results?subaction=viewrecord&from=export&id=L612922650>.

Schimke, A. *et al.* (2013) ‘Intervention and management of congenital left heart obstructive lesions’, *Current Treatment Options in Cardiovascular Medicine*. J. Kuvin, CardioVascular Center, Division of Cardiology, Tufts Medical Center, 800 Washington Street, Box 315, Boston, MA 02111, United States, 15(5), pp. 632–645. doi: 10.1007/s11936-013-0260-7.

Schmidt, T. *et al.* (2019) ‘Treatment Challenges in Patients with Acute Heart Failure and Severe Aortic Valve Stenosis’, *Current Cardiology Reports*. T. Schmidt, Department of Cardiology, Asklepios Klinik St. Georg, Lohmühlenstr. 5, Hamburg, Germany, United States, 21(6), p. 47. doi: 10.1007/s11886-019-1135-7.

Schmitto, J. D., Mohr, F. W. and Cohn, L. H. (2011) ‘Minimally invasive aortic valve replacement: how does this perform in high-risk patients?’, *Current opinion in cardiology*. United States, 26(2), pp. 118–122. doi: 10.1097/HCO.0b013e328343983a.

Schneider, A. E.. *et al.* (2014) ‘Percutaneous coronary intervention in pediatric and adolescent patients’, *Congenital Heart Disease*. J.N. Johnson, Pediatric Heart Transplant Program, Mayo Clinic, Gonda 138-SW, 200 First Street SW, Rochester, MN 55905, United States, 9(3), pp. 228–234. doi: 10.1111/chd.12130.

Schoen, F. J. (2012) ‘Mechanisms of function and disease of natural and replacement heart valves’, *Annual Review of Pathology: Mechanisms of Disease*. F.J. Schoen, Department of Pathology, Brigham and Women’s Hospital, Harvard Medical School, Boston, MA 02115, United States, pp. 161–183. doi: 10.1146/annurev-pathol-011110-130257.

Schoenenberger, A. W. *et al.* (2016) ‘Evolution of Cognitive Function after Transcatheter Aortic Valve Implantation’, *Circulation: Cardiovascular Interventions*. A.W. Schoenenberger, Department of Geriatrics, Inselspital, Bern University Hospital, University of Bern, Freiburgstrasse, Bern, Switzerland, United States, 9(10). doi: 10.1161/CIRCINTERVENTIONS.116.003590.

Schoenhagen, P. *et al.* (2013) ‘Transcatheter aortic valve repair, imaging, and electronic imaging health record topical collection on cardiac PET, CT, and MRI’, *Current Cardiology Reports*. P. Schoenhagen, Cleveland Clinic, Imaging Institute, Heart and Vascular Institute, Desk J1-4, 9500 Euclid Avenue, Cleveland, OH 44195, United States, United States, 15(1), p. 319. doi: 10.1007/s11886-012-0319-1.

Schwede, I. *et al.* (2007) ‘[Endocarditis due to Granulicatella adiacens].’, *Medizinische Klinik (Munich, Germany : 1983)*. Germany, 102(1), pp. 56–58. doi: 10.1007/s00063-007-1009-4.

Seamans, D. P. *et al.* (2016) ‘The utility of live video capture to enhance debriefing following transcatheter aortic valve replacement.’, *Annals of cardiac anaesthesia*. India, 19(Supplement), pp. S6–S11. doi: 10.4103/0971-9784.192576.

Segalote, R. C. *et al.* (2008) ‘Aortic valve preservation surgery in elderly patients with aortic stenosis’, *Brazilian Journal of Cardiovascular Surgery*. R. C. Segalote, Rua dois de dezembro, 66, apto 1004, Flamengo, Rio de Janeiro - RJ. CEP 22220-040, 23(4), pp. 519–523. Available at: http://www.embase.com/search/results?subaction=viewrecord&from=export&id=L354253234

Sehatzadeh, S. *et al.* (2013) ‘Transcatheter aortic valve implantation (TAVI) for treatment of aortic valve stenosis: An evidence update’, *Ontario Health Technology Assessment Series*. Canada, 13(1), pp. 1–40. Available at: <http://www.embase.com/search/results?subaction=viewrecord&from=export&id=L369081575>.

Sehatzadeh, S. *et al.* (2012) ‘Transcatheter aortic valve implantation (TAVI) for treatment of aortic valve stenosis: An evidence-based analysis (Part B)’, *Ontario Health Technology Assessment Series*. Canada, 12(14), pp. 1–62. Available at: <http://www.embase.com/search/results?subaction=viewrecord&from=export&id=L369081368>.

Sen, S. *et al.* (2014) ‘Wave intensity analysis in the human coronary circulation in health and disease’, *Current Cardiology Reviews*. S. Sen, International Centre of Circulatory Health, National Heart and Lung Institute, 59-61 North Wharf Road, London, W2 1LA, United Kingdom, 10(1), pp. 17–23. doi: 10.2174/1573403X10999140226121300.

Sergie, Z. *et al.* (2013) ‘Current periprocedural anticoagulation in transcatheter aortic valve replacement: Could bivalirudin be an option? Rationale and design of the BRAVO 2/3 studies’, *Journal of Thrombosis and Thrombolysis*. G.D. Dangas, Zena and Michael A. Wiener Cardiovascular Institute, Mount Sinai Medical Center, One Gustave L. Levy Place, New York NY 10029, United States, Netherlands, 35(4), pp. 483–493. doi: 10.1007/s11239-013-0890-3.

Setina, M. *et al.* (2002) ‘[Is a bioprosthesis with a rigid stent a good choice for aortic valve replacement in elderly patients?].’, *Rozhledy v chirurgii : mesicnik Ceskoslovenske chirurgicke spolecnosti*. Czech Republic, 81(8), pp. 401–404.

Sfeir, P. M.; Jebara, V. A. & Ayoub, C. M. (2006) ‘Mitral valve repair or replacement in elderly people’, *Current Opinion in Anaesthesiology*. C.M. Ayoub, Department of Anesthesiology, American University of Beirut Medical Center, PO Box 11, 0236 Beirut, Lebanon, 19(1), pp. 82–87. doi: 10.1097/01.aco.0000192771.79032.29.

Shah, B. *et al.* (2018) ‘Outcomes after transcatheter aortic valve replacement in patients with low versus high gradient severe aortic stenosis in the setting of preserved left ventricular ejection fraction’, *Journal of Interventional Cardiology*. B. Shah, Department of Medicine, Division of Cardiology, Veterans Affairs New York Harbor Health System and New York University (NYU) School of Medicine, New York, NY, United States, 31(6), pp. 849–860. doi: 10.1111/joic.12561.

Shah, P. M. and Roberts, D. L. (1977) ‘Diagnosis and treatment of aortic valve stenosis.’, *Current problems in cardiology*. United States, 2(6), pp. 1–49.

Sharma, A. *et al.* (2015) ‘Clinical applications of natriuretic peptides in assessment of valvular heart disease’, *Disease Markers*. A. Sharma, Division of Cardiovascular Diseases, State University of New York, Downstate Medical Center, New York, NY, United States, 2015. doi: 10.1155/2015/807861.

Sharma, A. *et al.* (2013) ‘Access site bleeding after transcatheter aortic valve implantation’, *Journal of Thrombosis and Thrombolysis*. A. Sharma, Department of Internal Medicine, Maimonides Medical Center, 1016 50th Street Apt 2C, Brooklyn NY 11219, United States, Netherlands, 35(4), pp. 463–468. doi: 10.1007/s11239-013-0928-6.

Shbeeb, I. *et al.* (1984) ‘The aortic valve. Colonic axis’, *Diseases of the Colon and Rectum*. Sansum Medical Clinic, Santa Barbara, CA 90026, United States, 27(1), pp. 38–41. Available at: http://www.embase.com/search/results?subaction=viewrecord&from=export&id=L14217580.

Shehatha, J. S.; Taha, A. Y. & Mirza, A. J. (2018) ‘Late Shone complex: A case report and literature review’, *Journal of the Egyptian Society of Cardio-Thoracic Surgery*. A.Y. Taha, Mamostayan Street 112, Road 33, House 5, P O Box 1155/64, Sulaymaniyah, Iraq, 26(2), pp. 133–135. doi: 10.1016/j.jescts.2018.03.007.

Shen, M. *et al.* (2018) ‘Blood, tissue and imaging biomarkers in calcific aortic valve stenosis: Past, present and future’, *Current Opinion in Cardiology*. M.-A. Clavel, Institut Universitaire de Cardiologie et de Pneumologie de Québec, Université Laval, #A-2047-2725, Québec, , QC, Canada, 33(2), pp. 125–133. doi: 10.1097/HCO.0000000000000487.

Sherwood, M. W. & Kiefer, T. L. (2017) ‘Challenges in Aortic Valve Stenosis: Low-Flow States Diagnosis, Management, and a Review of the Current Literature’, *Current Cardiology Reports*. M.W. Sherwood, Duke Clinical Research Institute, 2400 Pratt Street, P.O. Box 17969, Durham, NC, United States, 19(12). doi: 10.1007/s11886-017-0941-z.

Sherwood, M. W. and Vora, A. N. (2018) ‘Challenges in Aortic Stenosis: Review of Antiplatelet/Anticoagulant Therapy Management with Transcatheter Aortic Valve Replacement (TAVR): TAVR with Recent PCI, TAVR in the Patient with Atrial Fibrillation, and TAVR Thrombosis Management.’, *Current cardiology reports*. United States, 20(12), p. 130. doi: 10.1007/s11886-018-1073-9.

Shimura, T. *et al.* (2017) ‘Impact of the Clinical Frailty Scale on Outcomes After Transcatheter Aortic Valve Replacement.’, *Circulation*. United States, 135(21), pp. 2013–2024. doi: 10.1161/CIRCULATIONAHA.116.025630.

Shiyovich, A. and Kornowski, R. (2019) ‘Neuroprotective measures throughout the TAVI pathway.’, *Minerva cardioangiologica*. Italy, 67(1), pp. 39–56. doi: 10.23736/S0026-4725.18.04763-1.

Shreenivas, S., Kaneko, T. and Tang, G. H. L. (2019) ‘Predicting the future of TAVR: an obituary to open aortic valve replacement?’, *Current opinion in cardiology*. United States, 34(2), pp. 112–123. doi: 10.1097/HCO.0000000000000609.

Shrestha, M. *et al.* (2008) ‘A staged approach towards interventional aortic valve implantation with a sutureless valve: Initial human implants’, *Thoracic and Cardiovascular Surgeon*. M. Shrestha, Department of Cardiac, Thoracic, Transplantation and Vascular Surgery, Hannover Medical School, Carl-Neuberg-Str. 1, 30625 Hannover, Germany, 56(7), pp. 398–400. doi: 10.1055/s-2008-1038722.

Shunk, K. A. *et al.* (2015) ‘Development of a Veterans Affairs hybrid operating room for transcatheter aortic  valve replacement in the cardiac catheterization laboratory.’, *JAMA surgery*. United States, 150(3), pp. 216–222. doi: 10.1001/jamasurg.2014.1404.

Siddiqui, J. *et al.* (2013) ‘Surgical valvotomy and repair for neonatal and infant congenital aortic stenosis achieves better results than interventional catheterization’, *Journal of the American College of Cardiology*. Y. D’Udekem, Department of Cardiac Surgery, Royal Children’s Hospital, Flemington Road, Parkville, Melbourne, VIC 3052, Australia, 62(22), pp. 2134–2140. doi: 10.1016/j.jacc.2013.07.052.

Siegel, R. J. *et al.* (2012) ‘Echocardiography in transcatheter aortic valve implantation and mitral valve clip’, *Korean Journal of Internal Medicine*. R. J. Siegel, Cardiac Noninvasive Laboratory, Heart Institute, Cedar-Sinai Medical Center, 8700 Beverly Blvd, Los Angeles, CA 90048, United States, Korea (South), 27(3), pp. 245–261. doi: 10.3904/kjim.2012.27.3.245.

Singh, H. S. *et al.* (2013) ‘Interventional cardiology in adults with congenital heart disease’, *Nature Reviews Cardiology*. L.N. Benson, Department of Pediatrics, Labatt Family Heart Centre, Hospital for Sick Children, 555 University Avenue, Toronto, ON M5G 1X8, Canada, 10(11), pp. 662–678. doi: 10.1038/nrcardio.2013.127.

Singh, K. *et al.* (2017) ‘Efficacy and Complications of Transcatheter Aortic Valve Implantation With and Without Balloon Aortic Valvuloplasty’, *The Journal of heart valve disease*, 26(2), pp. 139–145. Available at: <http://www.embase.com/search/results?subaction=viewrecord&from=export&id=L621620045>.

Singh, V. *et al.* (2013) ‘Transcatheter aortic valve replacement: techniques, complications, and bailout strategies.’, *Postgraduate medicine*. V. Singh, Cardiovascular Division, University of Miami Miller School of Medicine, Miami, FL., 125(5), pp. 31–42. doi: 10.3810/pgm.2013.09.2697.

Singhal, P. *et al.* (2010) ‘Congenital left main coronary artery to main pulmonary artery fistula with bicuspid aortic valve: a case report and review of literature.’, *Journal of cardiac surgery*. United States, 25(3), pp. 295–299. doi: 10.1111/j.1540-8191.2010.01016.x.

Sintek, M. and Zajarias, A. (2014) ‘Patient evaluation and selection for transcatheter aortic valve replacement: the  heart team approach.’, *Progress in cardiovascular diseases*. United States, 56(6), pp. 572–582. doi: 10.1016/j.pcad.2014.02.003.

Siqueira, D. *et al.* (2012) ‘Transcatheter aortic valve replacement in elderly patients.’, *Journal of geriatric cardiology : JGC*. China, 9(2), pp. 78–82. doi: 10.3724/SP.J.1263.2011.12291.

Siripornpitak, S. *et al.* (2013) ‘Cardiac CT angiography in children with congenital heart disease’, *European Journal of Radiology*. S. Siripornpitak, Department of Diagnostic and Therapeutic Radiology, Ramathibodi Hospital, Mahidol University, 270 Rama VI Road, Bangkok 10400, Thailand, 82(7), pp. 1067–1082. doi: 10.1016/j.ejrad.2011.11.042.

Siu, S. C. *et al.* (2010) ‘Bicuspid Aortic Valve Disease’, *Journal of the American College of Cardiology*. S.C. Siu, Division of Cardiology, University of Western Ontario, London, Ont., Canada, United States, 55(25), pp. 2789–2800. doi: 10.1016/j.jacc.2009.12.068.

Sloan, K. P. *et al.* (2009) ‘Characterization of aortic valve disease in patients with Paget’s disease’, *Journal of the American Society of Echocardiography*. K.P. Sloan, Mayo Clinic, Rochester, MN, United States, 22(5), p. 599. doi: 10.1016/j.echo.2009.03.037.

Smith, J. A. *et al.* (1993) ‘Excellent long-term results of cardiac valve replacement with the St Jude Medical valve prosthesis.’, *Circulation*. United States, 88(5 Pt 2), pp. II49-54.

Smith, L. A. and Monaghan, M. J. (2013) ‘Monitoring of procedures: peri-interventional echo assessment for transcatheter aortic valve implantation.’, *European heart journal cardiovascular Imaging*. England, 14(9), pp. 840–850. doi: 10.1093/ehjci/jet042.

Smith-Parrish, M.; Yu, S. & Rocchini, A. (2014) ‘Obesity and elevated blood pressure following repair of coarctation of the aorta’, *Journal of Pediatrics*. M. Smith-Parrish, Division of Pediatric Cardiology, Department of Pediatrics, C. S. Mott Children’s Hospital, 1540 East Hospital Drive, Ann Arbor, MI 48109-4204, United States, 164(5), pp. 1074-1078.e1. doi: 10.1016/j.jpeds.2014.01.043.

Sokalskis, V. *et al.* (2017) ‘Echocardiographic follow-up after transcatheter aortic valve replacement.’, *Echocardiography (Mount Kisco, N.Y.)*. United States, 34(2), pp. 267–278. doi: 10.1111/echo.13443.

Solomonica, A., Choudhury, T. and Bagur, R. (2019) ‘Newer-generation of Edwards transcatheter aortic valve systems: SAPIEN 3, Centera, and SAPIEN 3 Ultra.’, *Expert review of medical devices*. England, 16(2), pp. 81–87. doi: 10.1080/17434440.2019.1555465.

Somers, P.; Knaapen, M. & Mistiaen, W. (2006) ‘Histopathology of calcific aortic valve stenosis’, *Acta Cardiologica*. M. Knaapen, Department of Pathology, Middelheim Hospital, Lindendreef 1, BE-2020 Antwerpen, Belgium, 61(5), pp. 557–562. doi: 10.2143/AC.61.5.2017772.

Sommers, K. E. & David, T. E. (1997) ‘Aortic valve replacement with patch enlargement of the aortic annulus’, *Annals of Thoracic Surgery*. T.E. David, 200 Elizabeth St, Toronto, Ont. M5G 2C4, Canada, 63(6), pp. 1608–1612. doi: 10.1016/S0003-4975(97)00127-6.

Soon, J. *et al.* (2017) ‘Multimodality Imaging for Planning and Follow-up of Transcatheter Aortic Valve Replacement.’, *The Canadian journal of cardiology*. England, 33(9), pp. 1110–1123. doi: 10.1016/j.cjca.2017.03.024.

Soon, J. L. *et al.* (2011) ‘Transapical transcatheter aortic valve implantation in the presence of a mitral prosthesis.’, *Journal of the American College of Cardiology*. United States, 58(7), pp. 715–721. doi: 10.1016/j.jacc.2011.04.023.

Sordelli, C. *et al.* (2014) ‘Echocardiographic assessment of heart valve prostheses’, *Journal of Cardiovascular Echography*. C. Sordelli, Via della Gioventù 12, Torre del greco (NA), Italy, 24(4), pp. 103–113. doi: 10.4103/2211-4122.147201.

Sorensen, B. *et al.* (1986) ‘[Compression of the right pulmonary artery by a dissecting aneurysm of the ascending aorta. Apropos of a case occurring long after aortic valve replacement].’, *Archives des maladies du coeur et des vaisseaux*. France, 79(7), pp. 1111–1115.

Sorrentino, S. *et al.* (2018) ‘Antithrombotic Treatment after Transcatheter Heart Valves Implant.’, *Seminars in thrombosis and hemostasis*. United States, 44(1), pp. 38–45. doi: 10.1055/s-0037-1607457.

Soschynski, M. *et al.* (2018) ‘Post-TAVI Follow-Up with MDCT of the Valve Prosthesis: Technical Application, Regular Findings and Typical Local Post-Interventional Complications.’, *RoFo : Fortschritte auf dem Gebiete der Rontgenstrahlen und der Nuklearmedizin*. Germany, 190(6), pp. 521–530. doi: 10.1055/s-0043-124190.

Spaccarotella, C. *et al.* (2017) ‘Transcatheter aortic valve implantation in patients at intermediate surgical risk’, *International Journal of Cardiology*, 243, pp. 161–168. doi: 10.1016/j.ijcard.2017.04.107.

Spaccarotella, C. *et al.* (2011) ‘Pathophysiology of aortic stenosis and approach to treatment with percutaneous valve implantation’, *Circulation Journal*. C. Indolfi, Department of Cardiology, University Magna Graecia, Catanzaro, Italy, Japan, 75(1), pp. 11–19. doi: 10.1253/circj.CJ-10-1105.

Spaziano, M. *et al.* (2014) ‘Imaging and functional testing to assess clinical and subclinical neurological events after transcatheter or surgical aortic valve replacement: A comprehensive review’, *Journal of the American College of Cardiology*. P. Généreux, Hôpital du Sacré-Coeur de Montréal, Université de Montréal, 5400 Boulevard Gouin Ouest, Montréal, QC, Canada, United States, 64(18), pp. 1950–1963. doi: 10.1016/j.jacc.2014.07.986.

Speiser, B. *et al.* (2014) ‘Transcatheter aortic valve replacement: establishing a comprehensive program model for hybrid cardiac catheterization laboratories in the Department of Veterans Affairs’, *Dimensions of critical care nursing : DCCN*. United States, 33(5), pp. 262–274. doi: 10.1097/DCC.0000000000000069.

Spina, R. *et al.* (2015) ‘Transcatheter Aortic Valve Replacement for Native Aortic Valve Regurgitation’, *Interventional Cardiology Review*. D. Roy, Interventional Cardiologist, Department of Cardiology, St Vincent’s Hospital, 390 Victoria Street, Darlinghurst, Sydney, NSW, Australia, England, 10(1), pp. 49–54. doi: 10.15420/icr.2015.10.1.49.

Spitzer, E. *et al.* (2018) ‘Moderate Aortic Stenosis and Reduced Left Ventricular Ejection Fraction: Current Evidence and Challenges Ahead.’, *Frontiers in cardiovascular medicine*. Switzerland, 5, p. 111. doi: 10.3389/fcvm.2018.00111.

Stahli, B. E. *et al.* (2013) ‘Aortic regurgitation after transcatheter aortic valve implantation: mechanisms and implications.’, *Cardiovascular diagnosis and therapy*. China (Republic : 1949- ), 3(1), pp. 15–22. doi: 10.3978/j.issn.2223-3652.2013.02.01.

Stahli, B. E. *et al.* (2018) ‘Transcatheter Aortic Valve Replacement and Concomitant Mitral Regurgitation.’, *Frontiers in cardiovascular medicine*. Switzerland, 5, p. 74. doi: 10.3389/fcvm.2018.00074.

Stefanini, G. G. *et al.* (2013) ‘Severe aortic stenosis and coronary artery disease’, *EuroIntervention*. P. Wenaweser, Department of Cardiology, Bern University Hospital, Bern, Switzerland, France, 9, pp. S63–S68. doi: 10.4244/EIJV9SSA12.

Steiner, J. *et al.* (2017) ‘Mechanical Intervention for Aortic Valve Stenosis in Patients With Heart Failure and Reduced Ejection Fraction.’, *Journal of the American College of Cardiology*. United States, 70(24), pp. 3026–3041. doi: 10.1016/j.jacc.2017.10.040.

Still, S., Szerlip, M. and Mack, M. (2018) ‘TAVR Vs. SAVR in Intermediate-Risk Patients: What Influences Our Choice of Therapy.’, *Current cardiology reports*. United States, 20(10), p. 82. doi: 10.1007/s11886-018-1026-3.

Stortecky, S. *et al.* (2012) ‘Transcatheter aortic valve implantation: prevention and management of complications.’, *Heart (British Cardiac Society)*. England, 98 Suppl 4, pp. iv52-64. doi: 10.1136/heartjnl-2012-302403.

Stortecky, S. *et al.* (2013) ‘Transcatheter aortic valve implantation: Patient selection’, *Minerva Cardioangiologica*. S. Windecker, Department of Cardiology, Swiss Cardiovascular Center Bern, Bern University Hospital, 3010 Bern, Switzerland, Italy, 61(5), pp. 487–497. Available at: <http://www.embase.com/search/results?subaction=viewrecord&from=export&id=L370496346>.

Storz, C. *et al.* (2016) ‘Role of Imaging in Transcatheter Aortic Valve Replacement.’, *Current treatment options in cardiovascular medicine*. United States, 18(10), p. 59. doi: 10.1007/s11936-016-0482-6.

Stouffer, G. A. *et al.* (2004) ‘Timing of aortic valve surgery in chronic aortic stenosis and regurgitation.’, *The American journal of the medical sciences*. United States, 327(6), pp. 348–351.

Streiff, M. B. & Ness, P. M. (2002) ‘Acquired FV inhibitors: a needless iatrogenic complication of bovine thrombin exposure.’, *Transfusion*. M.B. Streiff, Department of Medicine, Johns Hopkins University School of Medicine, Baltimore, Maryland 21205, USA., 42(1), pp. 18–26. doi: 10.1046/j.1537-2995.2002.00011.x.

Subramaniam, K. and Nazarnia, S. (2017) ‘Prosthesis-patient mismatch - what cardiac anesthesiologists need to know?’, *Annals of cardiac anaesthesia*. India, 20(2), pp. 234–242. doi: 10.4103/aca.ACA_9_17.

Sud, M., Tam, D. Y. and Wijeysundera, H. C. (2017) ‘The Economics of Transcatheter Valve Interventions.’, *The Canadian journal of cardiology*. England, 33(9), pp. 1091–1098. doi: 10.1016/j.cjca.2017.03.015.

Suda, H. *et al.* (1993) ‘[Quadricuspid aortic valve: a case report and the review of the literature].’, *Kyobu geka. The Japanese journal of thoracic surgery*. Japan, 46(3), pp. 283–286.

Sultan, I. *et al.* (2017) ‘Management of coronary obstruction following transcatheter aortic valve replacement.’, *Journal of cardiac surgery*. United States, 32(12), pp. 777–781. doi: 10.1111/jocs.13252.

Sulzenko, J., Tousek, P. and Linkova, H. (2014) ‘Infective endocarditis as a mid-term complication after transcatheter aortic valve implantation: case report and literature review.’, *Catheterization and cardiovascular interventions : official journal of the Society for Cardiac Angiography & Interventions*. United States, 84(2), pp. 311–315. doi: 10.1002/ccd.25454.

Sundh, F. and Ugander, M. (2014) ‘Impact of left bundle branch block after transcatheter aortic valve replacement.’, *Journal of electrocardiology*. United States, 47(5), pp. 608–611. doi: 10.1016/j.jelectrocard.2014.07.009.

Supariwala, A. *et al.* (2018) ‘Latent myopathy is more pronounced in patients with low flow versus normal flow aortic stenosis with normal left ventricular ejection fraction who are undergoing surgical aortic valve replacement: Multicenter study with a brief review of the literature.’, *Echocardiography (Mount Kisco, N.Y.)*. United States, 35(5), pp. 611–620. doi: 10.1111/echo.13839.

Suradi, H. S. and Hijazi, Z. M. (2015) ‘TAVR update: Contemporary data from the UK TAVI and US TVT registries.’, *Global cardiology science & practice*. Qatar, 2015(2), p. 21. doi: 10.5339/gcsp.2015.21.

Svensson, L. G. *et al.* (2013) ‘A comprehensive review of the PARTNER trial’, *Journal of Thoracic and Cardiovascular Surgery*. L.G. Svensson, Department of Thoracic and Cardiovascular Surgery, Heart and Vascular Institute, Cleveland Clinic, 9500 Euclid Ave, Desk J4-1, Cleveland, OH 44915, United States, United States, 145(3 SUPPL.), pp. S11–S16. doi: 10.1016/j.jtcvs.2012.11.051.

Szabo, T. A. *et al.* (2012) ‘Management of aortic valve bypass surgery’, *Seminars in Cardiothoracic and Vascular Anesthesia*. T.A. Szabo, Department of Anesthesiology, Ralph H. Johnson Veterans Affairs Medical Center, 109 Bee Street, Charleston, SC 29401, United States, United States, 16(1), pp. 52–58. doi: 10.1177/1089253211434565.

T.K.M., W. *et al.* (2014) ‘Aortic valve replacement in over 70-and over 80-year olds: 5-year cohort study’, *Asian Cardiovascular and Thoracic Annals*. T.K.M. Wang, Green Lane Cardiovascular Service, Auckland City Hospital, 2 Grafton Road, Grafton-Auckland-1023, New Zealand, 22(5), pp. 526–533. doi: 10.1177/0218492313497950.

Taha, S. *et al.* (2015) ‘Impact of residual coronary artery disease on patients undergoing TAVI: A meta-analysis of adjusted observational studies’, *International journal of cardiology*, 181, pp. 77–80. doi: 10.1016/j.ijcard.2014.11.150.

Takagi, H. *et al.* (2013) ‘A meta-analysis comparing observed 30-day all-cause mortality with the Society of Thoracic Surgeons Predicted Risk of Mortality in contemporary studies using Valve Academic Research Consortium definitions.’, *International journal of cardiology*. Netherlands, pp. 1598–1602. doi: 10.1016/j.ijcard.2013.01.038.

Takagi, H. and Umemoto, T. (2014) ‘A meta-analysis of adjusted observational studies for mortality in transapical versus transfemoral aortic valve implantation.’, *International journal of cardiology*. Netherlands, pp. 165–170. doi: 10.1016/j.ijcard.2014.03.191.

Takagi, H. *et al.* (2011) ‘Less invasiveness may not always result in less mortality: a meta-analysis of transcatheter versus surgical aortic valve replacement for aortic stenosis.’, *International journal of cardiology*. Netherlands, pp. 207–208. doi: 10.1016/j.ijcard.2011.07.081.

Takemura, T. *et al.* (2006) ‘Apicoaortic conduit insertion for elderly patients with acquired aortic stenosis and small aortic annulus’, *Kyobu geka. The Japanese journal of thoracic surgery*. T. Takemura, Department of Cardiovascular Surgery, National Health Organization Nagano Hospital, Ueda, Japan., Japan, 59(4), pp. 294–300. Available at: <http://www.embase.com/search/results?subaction=viewrecord&from=export&id=L43768392>.

Takeshita, T. *et al.* (2017) ‘Concomitant surgery for symptomatic severe carotid artery stenosis and aortic valve stenosis with contralateral internal carotid artery occlusion: A case report’, *Neurological Surgery*. T. Takeshita, Department of Neurosurgery, Prefectural Okinawa Nanbu Medical Center and Children’s Medical Center, 118-1 Arakawa, Haebaru-cho, Shimajiri-gun, Okinawa, Japan, 45(5), pp. 409–415. Available at: <http://www.embase.com/search/results?subaction=viewrecord&from=export&id=L617362503>.

Tami, L. F. *et al.* (1993) ‘Sinus of valsalva aneurysms involving both coronary ostia’, *Catheterization and Cardiovascular Diagnosis*. Z.G. Turi, Cardiac Catheterization Laboratory, Cardiology Division, Harper Hospital, 3990 John R, Detroit, MI 48201, United States, United States, 29(4), pp. 304–308. Available at: <http://www.embase.com/search/results?subaction=viewrecord&from=export&id=L23219016>.

Tamura, A.; Yoshizaki, T.; Kusadokoro, S. (2017) ‘Early Structural Valve Deterioration of Trifecta Biological Prosthesis;Report of a Case’, *Kyobu geka. The Japanese journal of thoracic surgery*, 70(7), pp. 533–535. Available at: <http://www.embase.com/search/results?subaction=viewrecord&from=export&id=L620220405>.

Taneja, R. *et al.* (2009) ‘The role of epicardial echocardiography in the measurement of transvalvular flow  velocities during aortic valve replacement.’, *Journal of cardiothoracic and vascular anesthesia*. United States, 23(3), pp. 292–297. doi: 10.1053/j.jvca.2009.01.007.

Taramasso, M. *et al.* (2019) ‘The Portico transcatheter aortic valve for the treatment of severe aortic stenosis’, *Future Cardiology*. M. Taramasso, Cardiovascular Surgical Department, Heart Valve Clinic, University of Zurich, Zurich, Switzerland, England, 15(1), pp. 31–37. doi: 10.2217/fca-2018-0070.

Taramasso, M. *et al.* (2014) ‘New devices for TAVI: technologies and initial clinical experiences.’, *Nature reviews. Cardiology*. England, 11(3), pp. 157–167. doi: 10.1038/nrcardio.2013.221.

Tarantini, G. *et al.* (2017) ‘Atrial fibrillation in patients undergoing transcatheter aortic valve implantation: Epidemiology, timing, predictors, and outcome’, *European Heart Journal*. G. Tarantini, Department of Cardiac, Thoracic and Vascular Sciences, University Hospital of Padova, Via Giustiniani 2, Padova, Italy, 38(17), pp. 1285–1293. doi: 10.1093/eurheartj/ehw456.

Tarantini, G., Nai Fovino, L. and Gersh, B. J. (2018) ‘Transcatheter aortic valve implantation in lower-risk patients: what is the perspective?’, *European heart journal*. England, 39(8), pp. 658–666. doi: 10.1093/eurheartj/ehx489.

Tavakoli, R. *et al.* (2018) ‘Biological aortic valve replacement: advantages and optimal indications of stentless compared to stented valve substitutes. A review.’, *General thoracic and cardiovascular surgery*. Japan, 66(5), pp. 247–256. doi: 10.1007/s11748-018-0884-3.

Teply, J. F., Grunkemeier, G. L. and Starr, A. (1981) ‘Cardiac valve replacement in patients over 75 years of age.’, *The Thoracic and cardiovascular surgeon*. Germany, 29(1), pp. 47–50. doi: 10.1055/s-2007-1023440.

Terre, J. A., George, I. and Smith, C. R. (2017) ‘Pros and cons of transcatheter aortic valve implantation (TAVI).’, *Annals of cardiothoracic surgery*. China, 6(5), pp. 444–452. doi: 10.21037/acs.2017.09.15.

Terwelp, M. D. *et al.* (2017) ‘Minimally Invasive Versus Transcatheter and Surgical Aortic Valve Replacement: A Propensity Matched Study’, *The Journal of heart valve disease*. England, 26(2), pp. 146–154. Available at: <http://www.embase.com/search/results?subaction=viewrecord&from=export&id=L621620129>.

Testa, L. *et al.* (2016) ‘Transcatheter mitral valve regurgitation treatment: State of the art and a glimpse to the future.’, *The Journal of thoracic and cardiovascular surgery*. United States, 152(2), pp. 319–327. doi: 10.1016/j.jtcvs.2016.04.055.

Thakur, K. *et al.* (2018) ‘Transcatheter aortic valve replacement: The year in review 2017’, *Journal of Interventional Cardiology*. K.J. Harjai, Department of Cardiology, Geisinger Northeast, Pearsall Heart Hospital, Wilkes Barre, PA, United States, United States, 31(5), pp. 543–552. doi: 10.1111/joic.12562.

Theriault-Lauzier, P. *et al.* (2015) ‘Computed Tomography for Structural Heart Disease and Interventions’, *Interventional Cardiology Review*. N. Piazza, Cardiology Division, McGill University Health Centre, 1001, Decarie Boulevard, Montreal, QC, Canada, England, 10(3), pp. 149–154. doi: 10.15420/ICR.2015.10.03.149.

Thiago, L. *et al.* (2016) ‘Statins for aortic valve stenosis’, *Sao Paulo Medical Journal*, 134(6), p. 555. doi: 10.1590/1516-3180.20161346T1.

Thielmann, M. *et al.* (2011) ‘Current developments in transcatheter aortic valve implantation techniques’, *Herz*. M. Thielmann, Department of Thoracic and Cardiovascular Surgery, West German Heart Center Essen, University Hospital Essen, Hufelandstr. 55, Essen 45122, Germany, Germany, 36(8), pp. 696–705. doi: 10.1007/s00059-011-3534-4.

Thomas, M. (2010) ‘The global experience with percutaneous aortic valve replacement’, *JACC: Cardiovascular Interventions*. M. Thomas, Cardiovascular Services, Guys and St. Thomas’ Hospital, London SE1 7EH, United Kingdom, United States, 3(11), pp. 1103–1109. doi: 10.1016/j.jcin.2010.10.001.

Thompson I I I, J. L. *et al.* (2012) ‘Risk of recurrent gastrointestinal bleeding after aortic valve replacement in patients with Heyde syndrome’, *Journal of Thoracic and Cardiovascular Surgery*. H.V. Schaff, Division of Cardiovascular Surgery, Mayo Clinic, 200 First St SW, Rochester, MN 55905, United States, United States, 144(1), pp. 112–116. doi: 10.1016/j.jtcvs.2011.05.034.

Thongprayoon, C., Cheungpasitporn, W. and Kashani, K. (2017) ‘The impact of frailty on mortality after transcatheter aortic valve replacement.’, *Annals of translational medicine*. China, p. 144. doi: 10.21037/atm.2017.01.35.

Thourani, V. H. *et al.* (2011) ‘Outcomes of off-pump aortic valve bypass surgery for the relief of aortic stenosis in adults.’, *The Annals of thoracic surgery*. Netherlands, 91(1), pp. 131–136. doi: 10.1016/j.athoracsur.2010.10.074.

Thourani, V. H. *et al.* (2015) ‘High-risk patients with inoperative aortic stenosis: use of transapical, transaortic, and transcarotid techniques.’, *The Annals of thoracic surgery*. Netherlands, 99(3), pp. 815–817. doi: 10.1016/j.athoracsur.2014.10.012.

Tiwari, N. and Madan, N. (2018) ‘Hypertension and transcatheter aortic valve replacement: parallel or series?’, *Integrated blood pressure control*. New Zealand, 11, pp. 81–91. doi: 10.2147/IBPC.S177258.

Tiwari, N. and Patel, K. (2018) ‘Newer echocardiographic techniques for aortic-valve imaging: Clinical aids today, clinical practice tomorrow.’, *World journal of cardiology*. United States, 10(8), pp. 62–73. doi: 10.4330/wjc.v10.i8.62.

Toggweiler, S. and Webb, J. G. (2012) ‘Challenges in transcatheter aortic valve implantation.’, *Swiss medical weekly*. Switzerland, 142, p. w13735. doi: 10.4414/smw.2012.13735.

Tomey, M. I., Gidwani, U. K. and Sharma, S. K. (2013) ‘Cardiac critical care after transcatheter aortic valve replacement.’, *Cardiology clinics*. Netherlands, 31(4), pp. 607–18, ix. doi: 10.1016/j.ccl.2013.07.006.

Totsugawa, T. *et al.* (2019) ‘Minimally invasive aortic valve replacement through a right anterolateral mini-thoracotomy for the treatment of octogenarians with aortic valve stenosis’, *Heart and Vessels*. T. Totsugawa, Department of Cardiovascular Surgery, The Sakakibara Heart Institute of Okayama, 2-5-1 Nakai-cho, Kita-ku, Okayama, Japan, 34(3), pp. 462–469. doi: 10.1007/s00380-018-1262-x.

Toutouzas, K. *et al.* (2019) ‘Biomarkers in aortic valve stenosis and their clinical significance in transcatheter aortic valve implantation’, *Current Medicinal Chemistry*. K. Toutouzas, Karaoli kai Dimitriou Street 24, Holargos, Athens, Greece, 26(5), pp. 864–872. doi: 10.2174/0929867324666170727110241.

Toutouzas, K. *et al.* (2018) ‘The requirement of extracorporeal circulation system for transluminal aortic valve replacement: Do we really need it in the catheterization laboratory?’, *Catheterization and cardiovascular interventions : official journal of the Society for Cardiac Angiography & Interventions*. United States, 91(5), pp. E43–E48. doi: 10.1002/ccd.25988.

Toutouzas , K.P. *et al.* (2012) ‘Recent valves used for transluminal implantation in patients with aortic valve stenosis’, *Recent Patents on Cardiovascular Drug Discovery*. K. P. Toutouzas, 26 Karaoli and Dimitriou str., Holargos, 15562, Athens, Greece, United Arab Emirates, 7(3), pp. 206–215. doi: 10.2174/157489012803832766.

Towler, D. A. (2013) ‘Molecular and cellular aspects of calcific aortic valve disease’, *Circulation Research*.

Tresch, D. D. & Knickelbine, T. (1994) ‘Aortic valvular stenosis in the elderly: A disorder with a favorable outcome if correctly diagnosed and treated’, *Cardiovascular Reviews and Reports*. D.D. Tresch, Department of Cardiology, Medical College of Wisconsin, Box 123, 8700 W. Wisconsin Avenue, Milwaukee, WI 53226, United States, 15(7), pp. 35–38. Available at: http://www.embase.com/search/results?subaction=viewrecord&from=export&id=L24217708.

Tsai, M.-T., Tang, G. H. L. and Cohen, G. N. (2016) ‘Year in review: transcatheter aortic valve replacement.’, *Current opinion in cardiology*. United States, 31(2), pp. 139–147. doi: 10.1097/HCO.0000000000000260.

Tsimikas, S. (2019) ‘Potential Causality and Emerging Medical Therapies for Lipoprotein(a) and Its Associated Oxidized Phospholipids in Calcific Aortic Valve Stenosis’, *Circulation research*, 124(3), pp. 405–415. doi: 10.1161/CIRCRESAHA.118.313864.

Tsu, L.V. *et al.* (2017) ‘A clinical review of surgical vs transcatheter aortic valve replacement in geriatric patients’, *Consultant Pharmacist*. L.V. Tsu, Chapman University School of Pharmacy, 9401 Jeronimo Road, Irvine, CA, United States, 32(4), pp. 202–214. doi: 10.4140/TCP.n.2017.202.

Unger, P. *et al.* (2016) ‘Pathophysiology and management of multivalvular disease’, *Nature Reviews Cardiology*. P. Unger, Cardiology Department, CHU Saint-Pierre, Université Libre de Bruxelles, 322 rue Haute, Brussels, Belgium, 13(7), pp. 429–440. doi: 10.1038/nrcardio.2016.57.

Urena, M. and Rodes-Cabau, J. (2015) ‘Managing heart block after transcatheter aortic valve implantation: from monitoring to device selection and pacemaker indications.’, *EuroIntervention : journal of EuroPCR in collaboration with the Working Group on  Interventional Cardiology of the European Society of Cardiology*. France, 11 Suppl W, pp. W101-5. doi: 10.4244/EIJV11SWA30.

Urso, S., Sadaba, R. and Aldamiz-Echevarria, G. (2009) ‘Is patient-prosthesis mismatch an independent risk factor for early and mid-term  overall mortality in adult patients undergoing aortic valve replacement?’, *Interactive cardiovascular and thoracic surgery*. England, 9(3), pp. 510–518. doi: 10.1510/icvts.2009.207597.

Urso, S., Sadaba, R. and de la Cruz, E. (2014) ‘[Asymptomatic severe aortic stenosis: a reopened debate].’, *Medicina clinica*. Spain, 142(9), pp. 406–411. doi: 10.1016/j.medcli.2013.05.004.

Vahanian, A. and Acar, C. (2005) ‘Percutaneous valve procedures: what is the future?’, *Current opinion in cardiology*. United States, 20(2), pp. 100–106.

Vahanian, A. *et al.* (2008) ‘Transcatheter valve implantation for patients with aortic stenosis: a position statement from the European Association of Cardio-Thoracic Surgery (EACTS) and the European Society of Cardiology (ESC), in collaboration with the European Association of Percutaneous Cardiovascular Interventions (EAPCI)’, *European journal of cardio-thoracic surgery : official journal of the European Association for Cardio-thoracic Surgery*. Germany, 34(1), pp. 1–8. doi: 10.1016/j.ejcts.2008.04.039.

Vahl, T. P., Kodali, S. K. and Leon, M. B. (2016) ‘Transcatheter Aortic Valve Replacement 2016: A Modern-Day “Through the Looking-Glass” Adventure.’, *Journal of the American College of Cardiology*. United States, 67(12), pp. 1472–1487. doi: 10.1016/j.jacc.2015.12.059.

Valvo, R. *et al.* (2019) ‘Antithrombotic Therapy in Transcatheter Aortic Valve Replacement.’, *Frontiers in cardiovascular medicine*. Switzerland, 6, p. 73. doi: 10.3389/fcvm.2019.00073.

Van Belle, E. *et al.* (2019) ‘von Willebrand Factor and Management of Heart Valve Disease: JACC Review Topic of the Week.’, *Journal of the American College of Cardiology*. United States, 73(9), pp. 1078–1088. doi: 10.1016/j.jacc.2018.12.045.

Van de Veire, N. R. L. (2010) ‘Imaging to guide transcatheter aortic valve implantation’, *Journal of Echocardiography*. N. R. L. Veire, Department of Cardiology, Leiden University Medical Center, Albinusdreef 2, 2333 ZA Leiden, Netherlands, Japan, 8(1), pp. 1–6. doi: 10.1007/s12574-010-0033-1.

Van Hemelrijck, M. *et al.* (2018) ‘Recent advances in understanding and managing aortic stenosis.’, *F1000Research*. England, 7, p. 58. doi: 10.12688/f1000research.11906.1.

Varadarajan, P. *et al.* (2006) ‘Clinical profile and natural history of 453 nonsurgically managed patients with severe aortic stenosis.’, *The Annals of thoracic surgery*. Netherlands, 82(6), pp. 2111–2115. doi: 10.1016/j.athoracsur.2006.07.048.

Vaszily, M. *et al.* (1988) ‘Successful surgical repair of a congenital subvalvular left ventricular aneurysm.’, *The Thoracic and cardiovascular surgeon*. Germany, 36(5), pp. 269–271. doi: 10.1055/s-2007-1020094.

Vavuranakis, M. *et al.* (2014) ‘TAVI in the case of preexisting mitral prosthesis: tips & tricks and literature review’, *The Journal of invasive cardiology*. United States, 26(11), pp. 609–613. Available at: <http://www.embase.com/search/results?subaction=viewrecord&from=export&id=L615102343>.

Vaz, A. *et al.* (2010) ‘Heyde syndrome - The link between aortic stenosis and gastrointestinal bleeding’, *Revista Portuguesa de Cardiologia*. A. Vaz, Serviço de Medicina I, Hospital S. Teotónio, Viseu, Portugal, 29(2), pp. 309–314. Available at: <http://www.embase.com/search/results?subaction=viewrecord&from=export&id=L361165012>.

Verdoia, M. *et al.* (2018) ‘Dual Versus Single Antiplatelet Regimen With or Without Anticoagulation in Transcatheter Aortic Valve Replacement: Indirect Comparison and Meta- analysis.’, *Revista espanola de cardiologia (English ed.)*. Spain, 71(4), pp. 257–266. doi: 10.1016/j.rec.2017.06.012.

Vida, V. L. *et al.* (2005) ‘Critical aortic stenosis in early infancy: surgical treatment for residual lesions after balloon dilation.’, *The Annals of thoracic surgery*. Netherlands, 79(1), pp. 42–47. doi: 10.1016/j.athoracsur.2004.02.120.

Virk, S. A. *et al.* (2015) ‘Systematic review of percutaneous coronary intervention and transcatheter aortic valve implantation for concomitant aortic stenosis and coronary artery disease’, *International Journal of Cardiology*. C. Cao, Systematic Review Unit, Collaborative Research (CORE) Group, Macquarie University, Sydney, Australia, Netherlands, 187(1), pp. 453–455. doi: 10.1016/j.ijcard.2015.03.391.

Vlastra, W. *et al.* (2018) ‘Cerebral protection devices during transcatheter aortic valve implantation’, *Trends in Cardiovascular Medicine*. R. Delewi, Heart Center, Academic Medical Center, University of Amsterdam, Amsterdam, Netherlands, United States, 28(6), pp. 412–418. doi: 10.1016/j.tcm.2018.01.007.

Vogelgesang, A., Hasenfuss, G. and Jacobshagen, C. (2017) ‘Low-flow/low-gradient aortic stenosis-Still a diagnostic and therapeutic challenge.’, *Clinical cardiology*. United States, 40(9), pp. 654–659. doi: 10.1002/clc.22728.

Vollema, E. M., Delgado, V. and Bax, J. J. (2019) ‘Echocardiography in Transcatheter Aortic Valve Replacement.’, *Heart, lung & circulation*. Australia, 28(9), pp. 1384–1399. doi: 10.1016/j.hlc.2018.12.020.

von Segesser, L. K. *et al.* (2013) ‘Prevention and management of potential adverse events during transapical aortic valve replacement’, *The Journal of heart valve disease*. England, 22(3), pp. 276–286. Available at: <http://www.embase.com/search/results?subaction=viewrecord&from=export&id=L603392751>.

Vora, A. N. and Rao, S. V (2018) ‘Percutaneous or surgical access for transfemoral transcatheter aortic valve implantation.’, *Journal of thoracic disease*. China, 10(Suppl 30), pp. S3595–S3598. doi: 10.21037/jtd.2018.09.48.

Voudris, K. V *et al.* (2018) ‘Timing and Outcomes of PCI in the TAVR Era.’, *Current treatment options in cardiovascular medicine*. United States, 20(3), p. 22. doi: 10.1007/s11936-018-0619-x.

Vranckx, P. *et al.* (2017) ‘Thrombo-embolic prevention after transcatheter aortic valve implantation.’, *European heart journal*. England, 38(45), pp. 3341–3350. doi: 10.1093/eurheartj/ehx390.

Vymazal, T. and T., V. (2015) ‘Minimally invasive approach to calcified aortic valve replacement: Anaesthetic considerations’, *Indian Journal of Anaesthesia*. T. Vymazal, Department of Anesthesiology and Intensive Care Medicine, 2nd School of Medicine Charles University, V Úvalu 84, Prague 5, Czech Republic, India, 59(6), pp. 338–341. doi: 10.4103/0019-5049.158731.

Waller, B. F. *et al.* (1991) ‘Catheter balloon valvuloplasty of stenotic aortic valves - Part II: Balloon valvuloplasty during life subsequent tissue examination’, *Clinical Cardiology*. B.F. Waller, Harcourt Road, Indianapolis, IN 46260, United States, 14(11), pp. 924–930. Available at: <http://www.embase.com/search/results?subaction=viewrecord&from=export&id=L21344245>.

Walther, T., Chu, M. W. A. and Mohr, F. W. (2008) ‘Transcatheter aortic valve implantation: time to expand?’, *Current opinion in cardiology*. United States, 23(2), pp. 111–116. doi: 10.1097/HCO.0b013e3282f47c1b.

Wanamaker, K. M., Magovern, G. J. J. and Moraca, R. J. (2012) ‘Aortic valve replacement in patients with systemic mastocytosis.’, *Journal of cardiac surgery*. United States, 27(2), pp. 189–191. doi: 10.1111/j.1540-8191.2011.01404.x.

Wang, T. K. M. *et al.* (2014) ‘Aortic valve replacement in over 70- and over 80-year olds: 5-year cohort study.’, *Asian cardiovascular & thoracic annals*. England, 22(5), pp. 526–533. doi: 10.1177/0218492313497950.

Warembourg, H.; Pauchant, M. & Soots, G. (1975) ‘The moment for surgery in aortic stenosis’, *Annales de Cardiologie et d’Angeiologie*. Clin. Med. Ouest, Cent. Hosp. Reg., Lille, 24(5), pp. 393–399. Available at: http://www.embase.com/search/results?subaction=viewrecord&from=export&id=L6146391.

Warkentin, T. E. *et al.* (2003) ‘Gastrointestinal bleeding, angiodysplasia, cardiovascular disease, and acquired von Willebrand syndrome.’, *Transfusion medicine reviews*. United States, 17(4), pp. 272–286.

Watt, M. *et al.* (2012) ‘Cost-effectiveness of transcatheter aortic valve replacement in patients ineligible for conventional aortic valve replacement.’, *Heart (British Cardiac Society)*. England, 98(5), pp. 370–376. doi: 10.1136/heartjnl-2011-300444.

Webb, J. and Cribier, A. (2011) ‘Percutaneous transarterial aortic valve implantation: what do we know?’, *European heart journal*. England, 32(2), pp. 140–147. doi: 10.1093/eurheartj/ehq453.

Weber, M. & Werner, N. (2019) ‘Transcatheter aortic valve implantation (TAVI)’, *Kardiologe*. N. Werner, Medizinische Klinik und Poliklinik II, Universitätsklinikum Bonn, Sigmund-Freud-Str. 25, Bonn, Germany, 13(1), pp. 51–60. doi: 10.1007/s12181-018-0298-6.

Weininger, M. *et al.* (2011) ‘Hemodynamic assessment of severe aortic stenosis: MRI evaluation of dynamic changes of vena contracta’, *Investigative Radiology*. M. Weininger, Department of Radiology and Radiological Science, Medical University of South Carolina, Ashley River Tower, 25 Courtenay Drive, Charleston, SC 29401, United States, United States, 46(1), pp. 1–10. doi: 10.1097/RLI.0b013e3181f79ca2.

Weir, I. R., Tian, L. and Trinquart, L. (2019) ‘Multivariate meta-analysis model for the difference in restricted mean survival times.’, *Biostatistics (Oxford, England)*. England. doi: 10.1093/biostatistics/kxz018.

Weissman, G. (2015) ‘Cardiac CT in 2015: Clinical Role According to Current Multi-Societal Guidelines’, *Current Cardiovascular Imaging Reports*. G. Weissman, Medstar Heart and Vascular Institute/Georgetown University, 110 Irving St. NW, Rm 1F-1221, Washington, DC, United States, 8(9). doi: 10.1007/s12410-015-9350-0.

Wenaweser, P. *et al.* (2016) ‘Transcatheter aortic valve implantation today and tomorrow’, *Swiss medical weekly*. Switzerland, 146, p. w14299. doi: 10.4414/smw.2016.14299.

West, P. N. and Weldon, C. S. (1978) ‘Reconstructive valve surgery.’, *The Annals of thoracic surgery*. Netherlands, 25(2), pp. 167–177. doi: 10.1016/s0003-4975(10)63514-x.

Wiedemann, D. *et al.* (2010) ‘The elderly patient and cardiac surgery - A mini-review’, *Gerontology*. D. Wiedemann, Innsbruck Medical University, University Clinic of Cardiac Surgery, Anichstrasse 35, AT-6020 Innsbruck, Austria, 56(3), pp. 241–249. doi: 10.1159/000248761. D.A. Towler, Diabetes and Obesity Research Center, Sanford-Burnham Medical Research Institute at Lake Nona, Florida Hospital Translational Research, 6400 Sanger Rd, Orlando, FL 32827, United States, 113(2), pp. 198–208. doi: 10.1161/CIRCRESAHA.113.300155.

Williams, J. W. J. W. *et al.* (2010) *No Title*. Rockville (MD).Williams, J. W. J. W. *et al.* (2010) *No Title*. Rockville (MD).

Willson, A. and Webb, J. (2011) ‘Transcatheter treatment approaches for aortic valve disease.’, *The international journal of cardiovascular imaging*. United States, 27(8), pp. 1123–1132. doi: 10.1007/s10554-011-9803-8.

Windram, J. D. *et al.* (2014) ‘Valvular heart disease in pregnancy.’, *Best practice & research. Clinical obstetrics & gynaecology*. Netherlands, 28(4), pp. 507–518. doi: 10.1016/j.bpobgyn.2014.03.009.

Witberg, G. *et al.* (2019) ‘Future Directions. Transcatheter Aortic Valve Implantation for Low-risk Patients: Inevitable Evolution or a Step Too Far?’, *Revista espanola de cardiologia (English ed.)*. Spain, 72(8), pp. 664–671. doi: 10.1016/j.rec.2019.02.009.

Wollersheim, L.W. *et al.* (2014) ‘Current status of surgical treatment for aortic valve stenosis’, *Journal of Cardiac Surgery*. L.W. Wollersheim, Department of Cardiothoracic Surgery, Academic Medical Center, University of Amsterdam, Meibergdreef 9, AZ Amsterdam, Netherlands, United States, 29(5), pp. 630–637. doi: 10.1111/jocs.12384.

Wong, C. Y., Green, P. and Williams, M. (2013) ‘Decision-making in transcatheter aortic valve replacement: the impact of frailty  in older adults with aortic stenosis.’, *Expert review of cardiovascular therapy*. England, 11(6), pp. 761–772. doi: 10.1586/erc.13.45.

Wu, A. *et al.* (2017) ‘Use of Historical Surgical Times to Predict Duration of Primary Aortic Valve Replacement.’, *Journal of cardiothoracic and vascular anesthesia*. United States, 31(3), pp. 810–815. doi: 10.1053/j.jvca.2016.11.023.

Wu, Z. *et al.* (2014) ‘Replacement of the st jude medical regent valve in the aortic position with a continuous suture technique in the small aortic root.’, *Journal of cardiac surgery*. United States, 29(2), pp. 170–174. doi: 10.1111/jocs.12227. Y., L. *et al.* (2006) ‘Cardiac surgery in the elderly’, *Bulletin de l’Academie Nationale de Medecine*. Y. Logeais, 31 bld de Sévigné, 35700 Rennes, 190(4–5), pp. 855–871. Available at: <http://www.embase.com/search/results?subaction=viewrecord&from=export&id=L44758018>.

Xi, W. *et al.* (2015) ‘Calcific aortic valve disease: A review of the pathogenesis and therapeutical trends’, *Academic Journal of Second Military Medical University*. Z.-N. Wang, Department of Cardiothoracic Surgery, Changzheng Hospital, Second Military Medical University, Shanghai, China, 36(3), pp. 309–314. doi: 10.3724/SP.J.1008.2015.00309.

Xiao, E. *et al.* (2017) ‘Prevalence and clinical correlates of left ventricular hypertrophy by sokolow-lyon and cornell electrocardiogram voltage criteria in transcatheter aortic valve replacement patients’, *Circulation: Cardiovascular Quality and Outcomes*. E. Xiao, Albany Med Cntr, Albany, NY, United States, 10. Available at: <http://www.embase.com/search/results?subaction=viewrecord&from=export&id=L621102150>.

Yalta, K. *et al.* (2019) ‘Serum copeptin might improve risk stratification and management of aortic valve stenosis: a review of pathophysiological insights and practical implications’, *Therapeutic advances in cardiovascular disease*, 13, p. 1753944719826420. doi: 10.1177/1753944719826420.

Yalta, K. *et al.* (2018) ‘Late coronary ischemıc syndromes assocıated wıth transcatheter aortıc valve ımplantatıon: A revıew of mechanıstıc and clınıcal aspects’, *Indian Heart Journal*. K. Yalta, Trakya University, Cardiology Department, Edirne, Turkey, 70(6), pp. 901–906. doi: 10.1016/j.ihj.2018.06.015.

Yeang, C.; Wilkinson, M. J. & Tsimikas, S. (2016) ‘Lipoprotein(a) and oxidized phospholipids in calcific aortic valve stenosis’, *Current Opinion in Cardiology*. S. Tsimikas, Division of Cardiovascular Diseases, Sulpizio Cardiovascular Center, Department of Medicine, University of California San Diego, 9500 Gilman Drive, BSB 1080, San Diego, CA, United States, 31(4), pp. 440–450. doi: 10.1097/HCO.0000000000000300.

Yeghiazaryan, K. *et al.* (2008) ‘Prediction of degeneration of native and bioprosthetic aortic valves: Issue-related particularities of Diabetes mellitus’, *Infectious Disorders - Drug Targets*. O. Golubnitschaja, Division of Molecular/Experimental Radiology, Department of Radiology, Rheinische Friedrich-Wilhelms-University of Bonn, Sigmund-Freud-Str. 25, D-53105 Bonn, Germany, United Arab Emirates, 8(2), pp. 88–99. doi: 10.2174/187152608784746547.

Yetkin, E. & Waltenberger, J. (2009) ‘Molecular and cellular mechanisms of aortic stenosis’, *International Journal of Cardiology*. E. Yetkin, Department of Cardiology, University Hospital Maastricht, Netherlands, 135(1), pp. 4–13. doi: 10.1016/j.ijcard.2009.03.108.

Yildirir, A. (2009) ‘[Valvular heart disease associated with coronary artery disease].’, *Anadolu kardiyoloji dergisi : AKD = the Anatolian journal of cardiology*. Turkey, 9 Suppl 1, pp. 10–16.

Yoon, S.-H. *et al.* (2018) ‘Diagnosis and outcomes of transcatheter aortic valve implantation in bicuspid aortic valve stenosis’, *Interventional Cardiology Review*. R. Makkar, Cedars-Sinai Heart Institute, 8700 Beverly Blvd, Los Angeles, CA, United States, England, 13(2), pp. 62–65. doi: 10.15420/icr.2018:8:2.

Yoon, S.-H. and Makkar, R. (2019) ‘Transcatheter Mitral Valve Replacement in Patients with Severe Mitral Annular Calcification.’, *Interventional cardiology clinics*. Netherlands, 8(3), pp. 301–312. doi: 10.1016/j.iccl.2019.02.008.

Yoshida, K. *et al.* (2006) ‘Acquired von Willebrand disease type IIA in patients with aortic valve stenosis’, *Annals of Thoracic Surgery*. K. Yoshida, Department of Cardiovascular and Thoracic Surgery, Akashi Medical Center, Akashi, 743-33 Okubo-cho, Yagi, Akashi, 674-0063, Japan, Netherlands, 81(3), pp. 1114–1116. doi: 10.1016/j.athoracsur.2005.01.023.

Young Lee, M. *et al.* (2015) ‘Mechanisms of Heart Block after Transcatheter Aortic Valve Replacement - Cardiac  Anatomy, Clinical Predictors and Mechanical Factors that Contribute to Permanent Pacemaker Implantation.’, *Arrhythmia & electrophysiology review*. England, 4(2), pp. 81–85. doi: 10.15420/aer.2015.04.02.81.

Young, M. N. and Inglessis, I. (2017) ‘Transcatheter Aortic Valve Replacement: Outcomes, Indications, Complications, and Innovations.’, *Current treatment options in cardiovascular medicine*. United States, 19(10), p. 81. doi: 10.1007/s11936-017-0580-0.

Young, M. N., Singh, V. and Sakhuja, R. (2018) ‘A Review of Alternative Access for Transcatheter Aortic Valve Replacement.’, *Current treatment options in cardiovascular medicine*. United States, 20(7), p. 62. doi: 10.1007/s11936-018-0648-5.

Yousef, A. *et al.* (2016) ‘Transcatheter Aortic Valve Implantation: Current and Evolving Indications.’, *The Canadian journal of cardiology*. England, 32(2), pp. 266–269. doi: 10.1016/j.cjca.2015.04.033.

Yudi, M. B. *et al.* (2018) ‘Coronary Angiography and Percutaneous Coronary Intervention After Transcatheter Aortic Valve Replacement.’, *Journal of the American College of Cardiology*. United States, 71(12), pp. 1360–1378. doi: 10.1016/j.jacc.2018.01.057.

Lok, Z. S. Y. *et al.* (2013) ‘Alkaptonuria-associated aortic stenosis’, *Journal of Cardiac Surgery*. J.A. Smith, Department of Surgery, Monash Medical Centre, Monash University, 246 Clayton Road, Clayton, VIC 3168, Australia, United States, 28(4), pp. 417–420. doi: 10.1111/jocs.12129.

Zalaquett, R. *et al.* (2011) ‘Aortic valve replacement with bioprosthesis in patients 70 years old or older with aortic stenosis’, *Revista Medica de Chile*. Z. S. Ricardo, Departamento de Enfermedades Cardiovasculares, Pontificia Universidad Católica de Chile, Marcoleta 367, 8 piso, Santiago, Chile, Chile, 139(2), pp. 150–157. doi: 10.4067/S0034-98872011000200002.

Zaleska-Kociecka, M., Dabrowski, M. and Stepinska, J. (2019) ‘Acute kidney injury after transcatheter aortic valve replacement in the elderly: outcomes and risk management.’, *Clinical interventions in aging*. New Zealand, 14, pp. 195–201. doi: 10.2147/CIA.S149916.

Zamorano, J. L., Goncalves, A. and Lang, R. (2014) ‘Imaging to select and guide transcatheter aortic valve implantation.’, *European heart journal*. England, 35(24), pp. 1578–1587. doi: 10.1093/eurheartj/eht569.

Zannis, K. *et al.* (2012) ‘New sutureless aortic valve prosthesis: Another tool in less invasive aortic valve replacement’, *Current Opinion in Cardiology*. K. Zannis, Departement de Pathologie Cardiaque, Institut Mutualiste Montsouris, 42 Bd Jourdan, 75014 Paris, France, United States, 27(2), pp. 125–129. doi: 10.1097/HCO.0b013e3283501a11.

Želízko, M. (2017) ‘TAVI – From patient selection to follow-up’, *Cor et Vasa*. M. Želízko, Klinika kardiologie IKEM, Vídeňská 1958/9, Praha, Czech Republic, 59(1), pp. e2–e9. doi: 10.1016/j.crvasa.2017.01.011.

Zeng, Y. *et al.* (2016) ‘Pathophysiology of valvular heart disease (Review)’, *Experimental and Therapeutic Medicine*. P. Zhang, Department of Cardiology, Xuzhou Central Hospital, The Affiliated Xuzhou Hospital of Medical College of Southeast University, 199 South Jiefang Road, Xuzhou, Jiangsu, China, 11(4), pp. 1184–1188. doi: 10.3892/etm.2016.3048.

ZENKER, R., BORST, H. G. and KLINNER, W. (1964) ‘[ADVANCES IN THE FIELD OF HEART SURGERY. 3. OPEN HEART OPERATIONS AND HEART SURGERY IN CHILDHOOD].’, *Deutsche medizinische Wochenschrift (1946)*. Germany, 89, pp. 2078–2081. doi: 10.1055/s-0028-1113239.

Zhang, P. and Melander, S. (2014) ‘Transcatheter aortic valve replacement for severe aortic stenosis.’, *Critical care nursing quarterly*. United States, 37(4), pp. 346–356. doi: 10.1097/CNQ.0000000000000035.

Zhang, R. *et al.* (2018) ‘Left ventricular hypertrophy by electrocardiogram criteria is associated with post-transcatheter aortic valve replacement high-degree atrioventricular block and left bundle branch block’, *Circulation: Cardiovascular Quality and Outcomes*. R. Zhang, Albany Med Cntr, Albany, NY, United States, 11. Available at: <http://www.embase.com/search/results?subaction=viewrecord&from=export&id=L625455088>.

Zhang, R. *et al.* (2018) ‘Prevalence and regression of left ventricular hypertrophy by sokolow-lyon and cornell electrocardiogram voltage criteria after transcatheter aortic valve replacement’, *Circulation: Cardiovascular Quality and Outcomes*. R. Zhang, Albany Med Cntr, Albany, NY, United States, 11. Available at: <http://www.embase.com/search/results?subaction=viewrecord&from=export&id=L625455091>.

Zhang, R. *et al.* (2017) ‘Prevalence and clinical correlates of LVH by sokolowlyon and cornell ECG voltage criteria in TAVR patients’, *Circulation: Cardiovascular Quality and Outcomes*. R. Zhang, Albany Med Cntr, Albany, NY, United States, 10. Available at: http://www.embase.com/search/results?subaction=viewrecord&from=export&id=L621102087.

Zhao, F., Xie, X. and Roach, M. (2015) ‘Computer Vision Techniques for Transcatheter Intervention.’, *IEEE journal of translational engineering in health and medicine*. United States, 3, p. 1900331. doi: 10.1109/JTEHM.2015.2446988.

Zhao, Y.; Nicoll, R.; He, Y. H. & Henein, M. Y. (2016) ‘The effect of statins on valve function and calcification in aortic stenosis: A meta-analysis’, *Atherosclerosis*. M.Y. Henein, Department of Public Health and Clinical Medicine, Umeå University, Heart Centre, Umeå, Sweden, 246, pp. 318–324. doi: 10.1016/j.atherosclerosis.2016.01.023.

Zhao, Y., Owen, A. and Henein, M. (2013) ‘Early valve replacement for aortic stenosis irrespective of symptoms results in better clinical survival: a meta-analysis of the current evidence.’, *International journal of cardiology*. Netherlands, 168(4), pp. 3560–3563. doi: 10.1016/j.ijcard.2013.05.089.

Zheng, Y. and Li, T. (2018) ‘Association between transcatheter aortic valve implantation or replacement and mortality, and major adverse events after coronary artery bypass grafting.’, *International journal of cardiology. Heart & vasculature*. Ireland, 21, pp. 57–63. doi: 10.1016/j.ijcha.2018.08.004.

Zhu, M., Li, M. and Lu, B. (2018) ‘Comment on “Cardiovascular morbidity and mortality in patients with aortic valve sclerosis: A systematic review and meta-analysis”.’, *International journal of cardiology*. Netherlands, p. 324. doi: 10.1016/j.ijcard.2018.05.004.

Zhu, Y. *et al.* (2016) ‘Outcomes After Operations for Unicuspid Aortic Valve With or Without Ascending Repair in Adults’, *Annals of Thoracic Surgery*. E.E. Roselli, Department of Thoracic and Cardiovascular Surgery, Cleveland Clinic, 9500 Euclid Ave, Desk J4-1, Cleveland, OH, United States, Netherlands, 101(2), pp. 613–619. doi: 10.1016/j.athoracsur.2015.07.058.

Zoltowska, D. M. *et al.* (2019) ‘Association Between Pulmonary Hypertension and Transcatheter Aortic Valve Replacement: Analysis of a Nationwide Inpatient Sample Database.’, *Reviews on recent clinical trials*. United Arab Emirates, 14(1), pp. 56–60. doi: 10.2174/1574887113666181120113034.

Zusterzeel, R. *et al.* (2018) ‘Sex-Specific Outcomes After Transcatheter Aortic Valve Replacement: FDA Patient-Level Meta-Analysis of Premarket Clinical Trials.’, *Journal of women’s health (2002)*. United States, 27(6), pp. 808–814. doi: 10.1089/jwh.2017.6760.

**Other than TAVI vs SAVR assessed in the study (n=208):**

Agarwal, S. *et al.* (2015) ‘Comparative meta-analysis of balloon-expandable and self-expandable valves for transcatheter aortic valve replacement.’, *International journal of cardiology*. Netherlands, 197, pp. 87–97. doi: 10.1016/j.ijcard.2015.06.002.

Akinseye, O.A. *et al.* (2018) ‘Clinical outcomes of coronary occlusion following transcatheter aortic valve replacement: A systematic review’, *Cardiovascular Revascularization Medicine*. U.N. Ibebuogu, 956 Court Avenue, Suite D 334, Memphis, TN, United States, United States, 19(2), pp. 229–236. doi: 10.1016/j.carrev.2017.09.006.

Alghamdi, A. A. *et al.* (2010) ‘Is concomitant surgery for moderate functional mitral regurgitation indicated during aortic valve replacement for aortic stenosis? A systematic review and evidence-based recommendations.’, *Journal of cardiac surgery*. United States, 25(2), pp. 182–187. doi: 10.1111/j.1540-8191.2009.00965.x.

Amrane, H. *et al.* (2017) ‘A meta-analysis on clinical outcomes after transaortic transcatheter aortic valve implantation by the Heart Team.’, *EuroIntervention : journal of EuroPCR in collaboration with the Working Group on  Interventional Cardiology of the European Society of Cardiology*. France, 13(2), pp. e168–e176. doi: 10.4244/EIJ-D-16-00103.

Anand, A. *et al.* (2017) ‘The relationship between preoperative frailty and outcomes following transcatheter aortic valve implantation: a systematic review and meta-analysis.’, *European heart journal. Quality of care & clinical outcomes*. England, 3(2), pp. 123–132. doi: 10.1093/ehjqcco/qcw030.

Andersson, C. *et al.* (2017) ‘Is the use of renin-angiotensin systeminhibitors in patients with aortic valve stenosis safe and of prognostic benefit? A systematic review and meta-analysis’, *European Heart Journal - Cardiovascular Pharmacotherapy*. C. Andersson, Department of Internal Medicine, Glostrup Hospital, University of Copenhagen, Nordre Ringvej 57, Glostrup, Denmark, England, 3(1), pp. 21–27. doi: 10.1093/ehjcvp/pvw027.

Ando, T. *et al.* (2016) ‘Iatrogenic Ventricular Septal Defect Following Transcatheter Aortic Valve Replacement: A Systematic Review.’, *Heart, lung & circulation*. Australia, 25(10), pp. 968–974. doi: 10.1016/j.hlc.2016.03.012.

Ando, T. *et al.* (2018) ‘A systematic review of reported cases of combined transcatheter aortic and mitral valve interventions.’, *Catheterization and cardiovascular interventions : official journal of the Society for Cardiac Angiography & Interventions*. United States, 91(1), pp. 124–134. doi: 10.1002/ccd.27256.

Ando, T. *et al.* (2018) ‘Comparison of outcomes in new-generation versus early-generation heart valve in transcatheter aortic valve implantation: A systematic review and meta-analysis.’, *Cardiovascular revascularization medicine : including molecular interventions*. United States, 19(2), pp. 186–191. doi: 10.1016/j.carrev.2017.07.006.

Athappan, G. *et al.* (2016) ‘A systematic review on the safety of second-generation transcatheter aortic valves’, *EuroIntervention*. S.R. Kapadia, Department of Cardiovascular Medicine, Heart and Vascular Institute, Cleveland Clinic, Cardiac Catheterization Laboratory, 9500 Euclid Avenue, J2-3, Cleveland, OH, United States, 11(9), pp. 1034–1043. doi: 10.4244/EIJV11I9A211.

Athappan, G. *et al.* (2013) ‘Incidence, predictors, and outcomes of aortic regurgitation after transcatheter aortic valve replacement: meta-analysis and systematic review of literature.’, *Journal of the American College of Cardiology*. United States: Elsevier Inc., 61(15), pp. 1585–1595. doi: 10.1016/j.jacc.2013.01.047.

Auffret, V. *et al.* (2017) ‘Feasibility, safety, and efficacy of transcatheter aortic valve replacement without balloon predilation: A systematic review and meta-analysis.’, *Catheterization and cardiovascular interventions : official journal of the Society for Cardiac Angiography & Interventions*. United States, 90(5), pp. 839–850. doi: 10.1002/ccd.27040.

Auffret, V. *et al.* (2016) ‘Predictors of Early Cerebrovascular Events in Patients With Aortic Stenosis Undergoing Transcatheter Aortic Valve Replacement.’, *Journal of the American College of Cardiology*. United States, 68(7), pp. 673–684. doi: 10.1016/j.jacc.2016.05.065.

Bagur, R. *et al.* (2016) ‘Transcatheter Aortic Valve Implantation With or Without Preimplantation Balloon Aortic Valvuloplasty: A Systematic Review and Meta-Analysis.’, *Journal of the American Heart Association*. England, 5(6). doi: 10.1161/JAHA.115.003191.

Bagur, R. *et al.* (2017) ‘Cerebral Embolic Protection Devices During Transcatheter Aortic Valve Implantation: Systematic Review and Meta-Analysis.’, *Stroke*. United States, 48(5), pp. 1306–1315. doi: 10.1161/STROKEAHA.116.015915.

Bajaj, A. *et al.* (2017) ‘Safety and feasibility of PCI in patients undergoing TAVR: A systematic review and meta-analysis.’, *Heart & lung : the journal of critical care*. United States, 46(2), pp. 92–99. doi: 10.1016/j.hrtlng.2016.12.003.

Bajaj, A. *et al.* (2016) ‘Impact of previous cardiac surgery on patients undergoing transcatheter aortic valve implantation: A meta-analysis’, *Heart and Lung: Journal of Acute and Critical Care*. A. Bajaj, 707 Tall Trees Drive, Scranton, United States, United States, 45(4), pp. 350–358. doi: 10.1016/j.hrtlng.2016.04.002.

Banerjee, K. *et al.* (2017) ‘Meta-Analysis of Usefulness of Anticoagulation After Transcatheter Aortic Valve Implantation.’, *The American journal of cardiology*. United States, 120(9), pp. 1612–1617. doi: 10.1016/j.amjcard.2017.07.059.

Bao, L. *et al.* (2018) ‘Feasibility and safety of combined percutaneous coronary intervention among high-risk patients with severe aortic stenosis undergoing transcatheter aortic valve implantation: a systematic review and meta-analysis.’, *European journal of cardio-thoracic surgery : official journal of the European Association for Cardio-thoracic Surgery*. Germany, 54(6), pp. 1052–1059. doi: 10.1093/ejcts/ezy240.

Barbanti, M. *et al.* (2017) ‘Transcatheter aortic valve replacement with new-generation devices: A systematic  review and meta-analysis.’, *International journal of cardiology*. Netherlands, 245, pp. 83–89. doi: 10.1016/j.ijcard.2017.07.083.

Bhatheja, S. *et al.* (2016) ‘Valvular performance and aortic regurgitation following transcatheter aortic valve replacement using Edwards valve versus CoreValve for severe aortic stenosis: A Meta-analysis.’, *Cardiovascular revascularization medicine : including molecular interventions*. United States, 17(4), pp. 248–255. doi: 10.1016/j.carrev.2016.02.007.

Biancari, F. *et al.* (2017) ‘Transcatheter aortic valve replacement in nonagenarians: early and intermediate outcome from the OBSERVANT study and meta-analysis of the literature’, *Heart and Vessels*. P. D’Errigo, National Center for Epidemiology, Surveillance and Health Promotion, Istituto Superiore di Sanità, Via Giano della Bella 34, Rome, Italy, Japan, 32(2), pp. 157–165. doi: 10.1007/s00380-016-0857-3.

Biancari, F. *et al.* (2014) ‘Basic data from 176 studies on the immediate outcome after aortic valve replacement with or without coronary artery bypass surgery.’, *Journal of cardiothoracic and vascular anesthesia*. United States, 28(5), pp. 1251–1256. doi: 10.1053/j.jvca.2013.07.020.

van den Boogert, T. P. W. *et al.* (2018) ‘CTCA for detection of significant coronary artery disease in routine TAVI work-up : A systematic review and meta-analysis.’, *Netherlands heart journal : monthly journal of the Netherlands Society of Cardiology and the Netherlands Heart Foundation*. Netherlands, 26(12), pp. 591–599. doi: 10.1007/s12471-018-1149-6.

Caceres, M., Braud, R. and Roselli, E. E. (2012) ‘The axillary/subclavian artery access route for transcatheter aortic valve replacement: a systematic review of the literature.’, *The Annals of thoracic surgery*. Netherlands, 93(3), pp. 1013–1018. doi: 10.1016/j.athoracsur.2011.10.056.

Chaikriangkrai, K. *et al.* (2017) ‘Diagnostic accuracy of coronary computed tomography angiography in aortic stenosis patients referred for aortic valve replacement: Systematic review and meta-analysis’, *Journal of the American College of Cardiology*. K. Chaikriangkrai, University of Iowa Hospitals and Clinics, Iowa City, IA, United States, 69(11), p. 1507. doi: 10.1016/S0735-1097(17)34896-9.

Chaikriangkrai, K. *et al.* (2018) ‘Diagnostic Accuracy of Coronary Computed Tomography Before Aortic Valve Replacement: Systematic Review and Meta-Analysis.’, *Journal of thoracic imaging*. United States, 33(4), pp. 207–216. doi: 10.1097/RTI.0000000000000322.

Chakravarty, T. *et al.* (2015) ‘Meta-Analysis of the Impact of Mitral Regurgitation on Outcomes After Transcatheter Aortic Valve Implantation’, *American Journal of Cardiology*. R.R. Makkar, Cedars-Sinai Heart Institute, Cedars-Sinai Medical Center, Los Angeles, California, United States, United States, 115(7), pp. 942–949. doi: 10.1016/j.amjcard.2015.01.022.

Chandrasekhar, J. *et al.* (2015) ‘Transfemoral vs Non-transfemoral Access for Transcatheter Aortic Valve Implantation: A Systematic Review and Meta-analysis.’, *The Canadian journal of cardiology*. England, 31(12), pp. 1427–1438. doi: 10.1016/j.cjca.2015.04.023.

Chen, C. *et al.* (2015) ‘Impact of renal dysfunction on mid-term outcome after transcatheter aortic valve  implantation: a systematic review and meta-analysis.’, *PloS one*. United States, 10(3), p. e0119817. doi: 10.1371/journal.pone.0119817.

Choudhury, T., Solomonica, A. and Bagur, R. (2019) ‘The Evolut R and Evolut PRO transcatheter aortic valve systems.’, *Expert review of medical devices*. England, 16(1), pp. 3–9. doi: 10.1080/17434440.2019.1557045.

Conrotto, F. *et al.* (2017) ‘Outcomes of patients with low-pressure aortic gradient undergoing transcatheter aortic valve implantation: A Meta-analysis’, *Catheterization and Cardiovascular Interventions*. F. Conrotto, Division of Cardiology, Città della Salute e della Scienza Hospital, Turin, Italy, 89(6), pp. 1100–1106. doi: 10.1002/ccd.26839.

Conrotto, F. *et al.* (2014) ‘Impact of access on TAVI procedural and midterm follow-up: a meta-analysis of 13  studies and 10,468 patients.’, *Journal of interventional cardiology*. United States, 27(5), pp. 500–508. doi: 10.1111/joic.12141.

Conrotto, F. *et al.* (2015) ‘Effect of gender after transcatheter aortic valve implantation: a meta-analysis.’, *The Annals of thoracic surgery*. Netherlands, 99(3), pp. 809–816. doi: 10.1016/j.athoracsur.2014.09.089.

D'Ascenzo, F. *et al.* (2013) ‘Mid-term prognostic value of coronary artery disease in patients undergoing transcatheter aortic valve implantation: a meta-analysis of adjusted observational results.’, *International journal of cardiology*. Netherlands: Elsevier Ireland Ltd, 168(3), pp. 2528–2532. doi: 10.1016/j.ijcard.2013.03.062.

De Sciscio, P. *et al.* (2017) ‘Quantifying the Shift Toward Transcatheter Aortic Valve Replacement in Low-Risk Patients: A Meta-Analysis.’, *Circulation. Cardiovascular quality and outcomes*. United States, 10(6), pp. 1–13. doi: 10.1161/CIRCOUTCOMES.116.003287.

De Vecchis, R. *et al.* (2013) ‘The issue whether it is feasible that progression of non-rheumatic aortic valve stenosis is retarded using statins: A meta-analysis’, *European Journal of Preventive Cardiology*. R. De Vecchis, Cardiology Unit, Presidio Sanitario Intermedio Elena d’Aosta, Naples, Italy, 20(1), p. S83. doi: 10.1177/2047487314530052.

De Vecchis, R. *et al.* (2013) ‘Statin use for nonrheumatic calcific aortic valve stenosis: A review with meta-analysis’, *Journal of Cardiovascular Medicine*. R. De Vecchis, Cardiology Unit, Presidio Sanitario Intermedio Elena d’Aosta, Napoli, Italy, 14(8), pp. 559–567. doi: 10.2459/JCM.0b013e3283587267.

Doebler, K. *et al.* (2012) ‘Indication and structures and management of transcatheter aortic valve implantation: A review of the literature’, *Thoracic and Cardiovascular Surgeon*. K. Doebler, MDK Baden-Württemberg-KCQ, Silberburgstraße 122, Stuttgart, 70176, Germany, Germany, 60(5), pp. 309–318. doi: 10.1055/s-0032-1322621.

Dunne, B. *et al.* (2015) ‘Transapical Versus Transaortic Transcatheter Aortic Valve Implantation: A Systematic Review.’, *The Annals of thoracic surgery*. Netherlands, 100(1), pp. 354–361. doi: 10.1016/j.athoracsur.2015.03.039.

Eaton, J. *et al.* (2014) ‘Is transcatheter aortic valve implantation (TAVI) a cost-effective treatment in patients who are ineligible for surgical aortic valve replacement? A systematic review of economic evaluations.’, *Journal of medical economics*. England, 17(5), pp. 365–375. doi: 10.3111/13696998.2014.903256.

Eggebrecht, H. (2012) ‘Risk of stroke after TAVI: A metaanalysis of 10,037 published patients’, *EuroIntervention*. H. Eggebrecht, Cardioangiological Center Bethanien, Frankfurt, Germany, 8, p. N198. Available at: <http://www.embase.com/search/results?subaction=viewrecord&from=export&id=L70887293>.

Eggebrecht, H. *et al.* (2013) ‘Emergent cardiac surgery During Transcatheter aortic valve implantation (TAVI): A weighted meta-analysis of 9,251 patients from 46 studies’, *EuroIntervention*. H. Eggebrecht, Cardioangiological Center Bethanien (CCB), Im Prüfling 23, 60389 Frankfurt, Germany, France, 8(9), pp. 1072–1080. doi: 10.4244/EIJV8I9A164.

Ehret, C. *et al.* (2017) ‘Is local anaesthesia a favourable approach for transcatheter aortic valve implantation? A systematic review and meta-analysis comparing local and general anaesthesia.’, *BMJ open*. England, 7(9), p. e016321. doi: 10.1136/bmjopen-2017-016321.

Eleid, M. F. *et al.* (2015) ‘Meta-Analysis of the Prognostic Impact of Stroke Volume, Gradient, and Ejection Fraction After Transcatheter Aortic Valve Implantation.’, *The American journal of cardiology*. United States, 116(6), pp. 989–994. doi: 10.1016/j.amjcard.2015.06.027.

Elkaryoni, A. *et al.* (2018) ‘Three-dimensional transesophageal echocardiography is an attractive alternative to cardiac multi-detector computed tomography for aortic annular sizing: Systematic review and meta-analysis.’, *Echocardiography (Mount Kisco, N.Y.)*. United States, 35(10), pp. 1626–1634. doi: 10.1111/echo.14147.

Elmistekawy, E. *et al.* (2010) ‘Apico-Aortic Conduit for severe aortic stenosis: Technique, applications, and systematic review.’, *Journal of the Saudi Heart Association*. Netherlands, 22(4), pp. 187–194. doi: 10.1016/j.jsha.2010.06.003.

Erlebach, M. *et al.* (2016) ‘VARC endpoint definition compliance rates in contemporary transcatheter aortic valve implantation studies.’, *EuroIntervention : journal of EuroPCR in collaboration with the Working Group on  Interventional Cardiology of the European Society of Cardiology*. France, 12(3), pp. 375–380. doi: 10.4244/EIJV12I3A60.

Fan, J. *et al.* (2019) ‘Impact of tricuspid regurgitation and right ventricular dysfunction on outcomes after transcatheter aortic valve replacement: A systematic review and meta-analysis’, *Clinical Cardiology*. J. Wang, Zhejiang University School of Medicine, Hangzhou, China, 42(1), pp. 206–212. doi: 10.1002/clc.23126.

Figulla, L. *et al.* (2011) ‘Transcatheter aortic valve implantation: Evidence on safety and efficacy compared with medical therapy. A systematic review of current literature’, *Clinical Research in Cardiology*. L. Figulla, Department of Cardiology, University Hospital of Essen, Hufelandstrasse 55, Essen 45122, Germany, Germany, 100(4), pp. 265–276. doi: 10.1007/s00392-010-0268-x.

Foroutan, F. *et al.* (2017) ‘Structural valve deterioration after transcatheter aortic valve implantation.’, *Heart (British Cardiac Society)*. England, 103(23), pp. 1899–1905. doi: 10.1136/heartjnl-2017-311329.

Foroutan, F. *et al.* (2016) ‘Prognosis after surgical replacement with a bioprosthetic aortic valve in patients with severe symptomatic aortic stenosis: Systematic review of observational studies’, *BMJ (Online)*. F. Foroutan, Department of Clinical Epidemiology and Biostatistics, McMaster University, 1280 Main St West, Hamilton, ON, Canada, 354. doi: 10.1136/bmj.i5065.

Fröhlich, G.M. *et al.* (2014) ‘Local versus general anesthesia for transcatheter aortic valve implantation (TAVR)--systematic review and meta-analysis’, *BMC medicine*, 12, p. 41. doi: 10.1186/1741-7015-12-41.G.M., F. *et al.* (2014) ‘Local versus general anesthesia for transcatheter aortic valve implantation (TAVR) - systematic review and meta-analysis’, *BMC Medicine*. P. Meier, The Heart Hospital, University College London Hospitals, London, United Kingdom, 12(1). doi: 10.1186/1741-7015-12-41.

Furukawa, H. *et al.* (2015) ‘Frailty in cardiothoracic surgery: systematic review of the literature’, *General Thoracic and Cardiovascular Surgery*. H. Furukawa, Department of Cardiovascular Surgery, Kawasaki Medical School, 577 Matsushima, Kurashiki, Japan, Japan, 63(8), pp. 425–433. doi: 10.1007/s11748-015-0553-8.

Gandhi, S. *et al.* (2015) ‘Comparison of Dual-antiplatelet Therapy to Mono-antiplatelet Therapy After Transcatheter Aortic Valve Implantation: Systematic Review and Meta-analysis.’, *The Canadian journal of cardiology*. England, 31(6), pp. 775–784. doi: 10.1016/j.cjca.2015.01.014.

Garcia, D.and Ansari, M. (2017) ‘Clinical and procedural outcomes in TAVR for bicuspid versus tricuspid aortic valve stenosis: A meta-analysis’, *Journal of the American College of Cardiology*. D. Garcia, Ochsner Heart and Vascular Institute, New Orleans, LA, United States, 70(18), pp. B184–B185. doi: 10.1016/j.jacc.2017.09.557.

Garcia, D. C. *et al.* (2014) ‘Device stratified comparison among transfemoral, transapical and transubclavian access for Transcatheter Aortic Valve Replacement (TAVR): a meta-analysis.’, *International journal of cardiology*. Netherlands, pp. e318-21. doi: 10.1016/j.ijcard.2013.12.162.

Gargiulo, G. *et al.* (2015) ‘Moderate and severe preoperative chronic kidney disease worsen clinical outcomes  after transcatheter aortic valve implantation: meta-analysis of 4992 patients.’, *Circulation. Cardiovascular interventions*. United States, 8(2), p. e002220. doi: 10.1161/CIRCINTERVENTIONS.114.002220.

Gargiulo, G. *et al.* (2015) ‘Impact of postoperative acute kidney injury on clinical outcomes after transcatheter aortic valve implantation: A meta-analysis of 5,971 patients.’, *Catheterization and cardiovascular interventions : official journal of the Society for Cardiac Angiography & Interventions*. United States, 86(3), pp. 518–527. doi: 10.1002/ccd.25867.

Genereux, P. *et al.* (2012) ‘Clinical outcomes after transcatheter aortic valve replacement using valve academic research consortium definitions: a weighted meta-analysis of 3,519 patients from 16 studies.’, *Journal of the American College of Cardiology*. United States, 59(25), pp. 2317–2326. doi: 10.1016/j.jacc.2012.02.022.

Gialama, F. *et al.* (2018) ‘Systematic review of the cost-effectiveness of transcatheter interventions for valvular heart disease.’, *European heart journal. Quality of care & clinical outcomes*. England, 4(2), pp. 81–90. doi: 10.1093/ehjqcco/qcx049.

Giordana, F. *et al.* (2014) ‘Meta-analysis of predictors of all-cause mortality after transcatheter aortic valve implantation’, *American Journal of Cardiology*. F. Giordana, Division of Cardiology, Department of Internal Medicine, University of Turin, Turin, Italy, United States, 114(9), pp. 1447–1455. doi: 10.1016/j.amjcard.2014.07.081.

Giustino, *G. et al.* (2016) ‘Neurological Outcomes With Embolic Protection Devices in Patients Undergoing Transcatheter Aortic Valve Replacement: A Systematic Review and Meta-Analysis of Randomized Controlled Trials.’, *JACC. Cardiovascular interventions*. United States, 9(20), pp. 2124–2133. doi: 10.1016/j.jcin.2016.07.024.

Hardikar, A. A. and Marwick, T. H. (2013) ‘Surgical thresholds for bicuspid aortic valve associated aortopathy.’, *JACC. Cardiovascular imaging*. United States, 6(12), pp. 1311–1320. doi: 10.1016/j.jcmg.2013.10.005.

Harky, A. *et al.* (2018) ‘Stented Versus Stentless Aortic Valve Replacement in Patients With Small Aortic Root: A Systematic Review and Meta-Analysis’, *Innovations (Philadelphia, Pa.)*, 13(6), pp. 404–416. doi: 10.1097/IMI.0000000000000569.

Harling, L. *et al.* (2011) ‘Aortic valve replacement for aortic stenosis in patients with concomitant mitral  regurgitation: should the mitral valve be dealt with?’, *European journal of cardio-thoracic surgery : official journal of the European Association for Cardio-thoracic Surgery*. Germany, 40(5), pp. 1087–1096. doi: 10.1016/j.ejcts.2011.03.036.harling

Hassell, M. E. C. J. *et al.* (2015) ‘Antiplatelet therapy following transcatheter aortic valve implantation’, *Heart (British Cardiac Society)*. England, 101(14), pp. 1118–1125. doi: 10.1136/heartjnl-2014-307053.

He, Z.M. *et al.* (2017) ‘Sleep-disordered breathing and severe aortic stenosis’, *Somnologie*. Z.M. He, Department of Respiratory and Critical Care Medicine, Karamay Central Hospital, Karamay, China, 21(4), pp. 265–272. doi: 10.1007/s11818-017-0108-6.

Head, S. J. *et al.* (2012) ‘The impact of prosthesis-patient mismatch on long-term survival after aortic valve replacement: a systematic review and meta-analysis of 34 observational studies comprising 27 186 patients with 133 141 patient-years.’, *European heart journal*. England, 33(12), pp. 1518–1529. doi: 10.1093/eurheartj/ehs003.

Hill, G.D. *et al.* (2016) ‘Surgical Valvotomy Versus Balloon Valvuloplasty for Congenital Aortic Valve Stenosis: A Systematic Review and Meta-Analysis’, *Journal of the American Heart Association*. G.D. Hill, 9000 W Wisconsin Avenue, Milwaukee, WI, United States, 5(8). doi: 10.1161/JAHA.116.003931.

Ho, C., Cimon, K. and Rabb, D. (2013) *Transcatheter Aortic Valve Replacement in Severe Aortic Stenosis: A Review of Comparative Durability and Clinical Effectiveness Beyond 12 Months*. Ottawa (ON).

Hurley, E. T. *et al.* (2015) ‘A Meta-Analysis Examining Differences in Short-Term Outcomes Between Sutureless and Conventional Aortic Valve Prostheses.’, *Innovations (Philadelphia, Pa.)*. United States, 10(6), pp. 375–382. doi: 10.1097/IMI.0000000000000221.

Huygens, S. A., Takkenberg, J. J. M. and Rutten-van Molken, M. P. M. H. (2018) ‘Systematic review of model-based economic evaluations of heart valve implantations.’, *The European journal of health economics : HEPAC : health economics in prevention and care*. Germany, 19(2), pp. 241–255. doi: 10.1007/s10198-017-0880-z.

Ibebuogu, U. N. *et al.* (2015) ‘Review of reported causes of device embolization following trans-catheter aortic  valve implantation.’, *The American journal of cardiology*. United States, 115(12), pp. 1767–1772. doi: 10.1016/j.amjcard.2015.03.024.ifedil

Ifedili, I. A. *et al.* (2017) ‘Impact of Pre-existing Kidney Dysfunction on Outcomes Following Transcatheter Aortic Valve Replacement.’, *Current cardiology reviews*. United Arab Emirates, 13(4), pp. 283–292. doi: 10.2174/1573403X13666170804151608.

Indraratna, P. *et al.* (2014) ‘Systematic review of the cost-effectiveness of transcatheter aortic valve implantation.’, *The Journal of thoracic and cardiovascular surgery*. United States, 148(2), pp. 509–514. doi: 10.1016/j.jtcvs.2013.10.023.

Jiang, J. *et al.* (2018) ‘Transcatheter Aortic Valve Replacement for Pure Native Aortic Valve Regurgitation: A Systematic Review.’, *Cardiology*. Switzerland, pp. 132–140. doi: 10.1159/000491919.

Johansson S. and Lind, M.N. (2017) ‘Central regional anaesthesia in patients with aortic stenosis – a systematic review’, *Danish Medical Journal*. S. Johansson, Department of Anaesthesiology, Copenhagen University Hospital, Herlev, Denmark, 64(9). Available at: <http://www.embase.com/search/results?subaction=viewrecord&from=export&id=L618291070>.

Kanjanahattakij, N. *et al.* (2018) ‘Comparing outcomes after transcatheter aortic valve replacement in patients with  stenotic bicuspid and tricuspid aortic valve: A systematic review and meta-analysis.’, *Clinical cardiology*. United States, 41(7), pp. 896–902. doi: 10.1002/clc.22992.

Kanjanahattakij, N. *et al.* (2018) ‘Anaemia and mortality in patients with transcatheter aortic valve replacement: a  systematic review and meta-analysis.’, *Acta cardiologica*. England, pp. 1–7. doi: 10.1080/00015385.2018.1510802.

Khan, M. M. *et al.* (2018) ‘Cognitive Outcomes After Transcatheter Aortic Valve Implantation: A Metaanalysis.’, *Journal of the American Geriatrics Society*. United States, 66(2), pp. 254–262. doi: 10.1111/jgs.15123.

Kim, S.-J. *et al.* (2014) ‘A critical review of hemodynamic changes and left ventricular remodeling after surgical aortic valve replacement and percutaneous aortic valve replacement.’, *American heart journal*. United States, 168(2), pp. 150–157. doi: 10.1016/j.ahj.2014.04.015.

Kokkinidis, D.G. *et al.* (2018) ‘The predictive value of baseline pulmonary hypertension in early and long term cardiac and all-cause mortality after transcatheter aortic valve implantation for patients with severe aortic valve stenosis: A systematic review and meta-analysis’, *Cardiovascular Revascularization Medicine*. D.G. Kokkinidis, Damianos Kokkinidis, Department of Medicine, Jacobi Medical Center, Albert Einstein College of Medicine, Pelham Parkway, NY, United States, 19(7), pp. 859–867. doi: 10.1016/j.carrev.2018.03.015.

Korteland, N. M. *et al.* (2017) ‘Mechanical aortic valve replacement in non-elderly adults: meta-analysis and microsimulation.’, *European heart journal*. England, 38(45), pp. 3370–3377. doi: 10.1093/eurheartj/ehx199.

Kotronias, R. A. *et al.* (2018) ‘Early Versus Standard Discharge After Transcatheter Aortic Valve Replacement: A Systematic Review and Meta-Analysis.’, *JACC. Cardiovascular interventions*. United States, 11(17), pp. 1759–1771. doi: 10.1016/j.jcin.2018.04.042.

Kotronias, R.A. *et al.* (2017) ‘Transcatheter Aortic Valve Implantation With or Without Percutaneous Coronary Artery Revascularization Strategy: A Systematic Review and Meta-Analysis’, *Journal of the American Heart Association*. England, 6(6). doi: 10.1161/JAHA.117.005960.

Krasopoulos, G. *et al.* (2016) ‘European real world trans-catheter aortic valve implantation: systematic review and meta-analysis of European national registries’, *Journal of cardiothoracic surgery*, 11(1), p. 159. Available at: <http://www.embase.com/search/results?subaction=viewrecord&from=export&id=L616637803>.

Kularatna, S. *et al.* (2016) ‘HEALTH TECHNOLOGY ASSESSMENTS REPORTING COST-EFFECTIVENESS OF TRANSCATHETER AORTIC VALVE IMPLANTATION.’, *International journal of technology assessment in health care*. England, 32(3), pp. 89–96. doi: 10.1017/S0266462316000180.

Kumar, A. *et al.* (2016) ‘Balloon Aortic Valvuloplasty in the Transcatheter Aortic Valve Replacement Era.’, *The Journal of invasive cardiology*. United States, 28(8), pp. 341–348.

Lai, K. S. P. *et al.* (2015) ‘Cognitive outcomes following transcatheter aortic valve implantation: A systematic review’, *Cardiovascular Psychiatry and Neurology*. K.L. Lanctôt, Neuropsychopharmacology Research Group, Sunnybrook Health Sciences Centre, Toronto, ON, Canada, Egypt, 2015, p. 209569. doi: 10.1155/2015/209569.

Lee, J. C. *et al.* (2018) ‘Evaluation of aortic regurgitation with cardiac magnetic resonance imaging: a systematic review.’, *Heart (British Cardiac Society)*. England, 104(2), pp. 103–110. doi: 10.1136/heartjnl-2016-310819.

Li, X. *et al.* (2013) ‘Comparison 30-day clinical complications between transfemoral versus transapical aortic valve replacement for aortic stenosis: a meta-analysis review.’, *Journal of cardiothoracic surgery*. X. Li, Cardiaovascular surgery, Department of second affiliated hospital, school of Medicine, Zhejiang university, No, 88, Jie fang road, Hangzhou, Zhejiang province 310009, China., England, 8, p. 168. doi: 10.1186/1749-8090-8-168.

Li, liaoY. *et al.* (2014) ‘Improvement in quality of life in old people with aortic stenosis after transcatheter aortic valve implantation’, *Current Signal Transduction Therapy*. W. Guo, Department of Vascular Surgery, Clinical Division of Surgery, Chinese PLA General Hospital, 28 Fuxing Road, Beijing, China, 9(3), pp. 164–171. Available at: <http://www.embase.com/search/results?subaction=viewrecord&from=export&id=L604399108>.

Liao, Y.-B. *et al.* (2016) ‘The relationship between chronic obstructive pulmonary disease and transcatheter  aortic valve implantation--A systematic review and meta-analysis.’, *Catheterization and cardiovascular interventions : official journal of the Society for Cardiac Angiography & Interventions*. United States, 87 Suppl 1, pp. 570–578. doi: 10.1002/ccd.26443.

Liao, Y.-B. *et al.* (2016) ‘Meta-Analysis of the Effectiveness and Safety of Transcatheter Aortic Valve Implantation Without Balloon Predilation.’, *The American journal of cardiology*. United States, 117(10), pp. 1629–1635. doi: 10.1016/j.amjcard.2016.02.036.

Lim, W. Y. *et al.* (2017) ‘Meta-analysis of the impact of intervention versus symptom-driven management in asymptomatic severe aortic stenosis.’, *Heart (British Cardiac Society)*. England, 103(4), pp. 268–272. doi: 10.1136/heartjnl-2016-309830.liu

Liu, Z. *et al.* (2016) ‘Transfemoral versus Transapical Aortic Implantation for Aortic Stenosis Based on  No Significant Difference in Logistic EuroSCORE: A Meta-Analysis.’, *The Thoracic and cardiovascular surgeon*. Germany, 64(5), pp. 374–381. doi: 10.1055/s-0035-1555606.

Lloyd, D. *et al.* (2019) ‘Transcatheter, sutureless and conventional aortic-valve replacement: a network meta-analysis of 16,432 patients.’, *Journal of thoracic disease*. China, 11(1), pp. 188–199. doi: 10.21037/jtd.2018.12.27.

Luo, X. *et al.* (2015) ‘Efficacy of transcatheter aortic valve implantation in patients with aortic stenosis and reduced LVEF. A systematic review.’, *Herz*. Germany, 40 Suppl 2, pp. 168–180. doi: 10.1007/s00059-014-4193-z.

Lv, W. *et al.* (2017) ‘The “obesity paradox” does exist in patients undergoing transcatheter aortic valve implantation for aortic stenosis: a systematic review and meta-analysis.’, *Interactive cardiovascular and thoracic surgery*. England, 25(4), pp. 633–642. doi: 10.1093/icvts/ivx191.

Lv, W. *et al.* (2018) ‘Diabetes mellitus is an independent prognostic factor for mid-term and long-term  survival following transcatheter aortic valve implantation: a systematic review and meta-analysis.’, *Interactive cardiovascular and thoracic surgery*. England, 27(2), pp. 159–168. doi: 10.1093/icvts/ivy040.

Maas, E. H. A. *et al.* (2016) ‘General or Local Anesthesia for TAVI? A Systematic Review of the Literature and Meta-Analysis.’, *Current pharmaceutical design*. United Arab Emirates, 22(13), pp. 1868–1878.

Makki, N. and Lilly, S. M. (2018) ‘Advanced chronic kidney disease: Relationship to outcomes post-TAVR, a meta-analysis.’, *Clinical cardiology*. United States, 41(8), pp. 1091–1096. doi: 10.1002/clc.22993.

Maniotis, C. *et al.* (2017) ‘A systematic review on the safety of Prostar XL versus ProGlide after TAVR and EVAR.’, *Cardiovascular revascularization medicine : including molecular interventions*. United States, 18(2), pp. 145–150. doi: 10.1016/j.carrev.2016.11.004.

Meco, M. *et al.* (2018) ‘Sutureless aortic valve replacement versus transcatheter aortic valve implantation: a meta-analysis of comparative matched studies using propensity score matching.’, *Interactive cardiovascular and thoracic surgery*. England, 26(2), pp. 202–209. doi: 10.1093/icvts/ivx294.

Mina, G. S. *et al.* (2017) ‘Diabetes mellitus is associated with increased acute kidney injury and 1-year mortality after transcatheter aortic valve replacement: A meta-analysis.’, *Clinical cardiology*. United States, 40(9), pp. 726–731. doi: 10.1002/clc.22723.

Mohananey, D. *et al.* (2017) ‘Clinical and Echocardiographic Outcomes Following Permanent Pacemaker Implantation After Transcatheter Aortic Valve Replacement: Meta-Analysis and Meta-Regression.’, *Circulation. Cardiovascular interventions*. United States, 10(7). doi: 10.1161/CIRCINTERVENTIONS.117.005046.

Mojoli, M. *et al.* (2017) ‘Impact of atrial fibrillation on outcomes of patients treated by transcatheter aortic valve implantation: A systematic review and meta-analysis.’, *American heart journal*. United States, 192, pp. 64–75. doi: 10.1016/j.ahj.2017.07.005.

Mookadam, F. *et al.* (2010) ‘Unicuspid aortic valve in children: a systematic review spanning four decades.’, *The Journal of heart valve disease*. England, 19(6), pp. 678–683.

Muralidharan, A. *et al.* (2016) ‘Meta-Analysis of Perioperative Stroke and Mortality in Transcatheter Aortic Valve Implantation.’, *The American journal of cardiology*. United States, 118(7), pp. 1031–1045. doi: 10.1016/j.amjcard.2016.07.011.

Mylotte, D. *et al.* (2015) ‘Transcatheter heart valve failure: a systematic review.’, *European heart journal*. England, 36(21), pp. 1306–1327. doi: 10.1093/eurheartj/ehu388.

Nalluri, N. *et al.* (2018) ‘Valve in valve transcatheter aortic valve implantation (ViV-TAVI) versus redo—Surgical aortic valve replacement (redo-SAVR): A systematic review and meta-analysis’, *Journal of Interventional Cardiology*. N. Nalluri, Department of Cardiology, Staten Island University Hospital, Northwell Health, Staten Island, New York City, NY, United States, 31(5), pp. 661–671. doi: 10.1111/joic.12520.

Neupane, S. *et al.* (2018) ‘Meta-Analysis of Transcatheter Valve-in-Valve Implantation Versus Redo Aortic Valve Surgery for Bioprosthetic Aortic Valve Dysfunction.’, *The American journal of cardiology*. United States, 121(12), pp. 1593–1600. doi: 10.1016/j.amjcard.2018.02.054.

Nombela-Franco, L. *et al.* (2015) ‘Clinical impact and evolution of mitral regurgitation following transcatheter aortic valve replacement: a meta-analysis.’, *Heart (British Cardiac Society)*. England, 101(17), pp. 1395–1405. doi: 10.1136/heartjnl-2014-307120.]

O’ Sullivan, K. E. *et al.* (2014) ‘Is local anesthesia the optimum strategy in retrograde transcatheter aortic valve implantation? A systematic review and meta-analysis.’, *The Thoracic and cardiovascular surgeon*. Germany, 62(6), pp. 489–497. doi: 10.1055/s-0034-1383721.

O’ Sullivan, K. E. *et al.* (2015) ‘Transaortic TAVI Is a Valid Alternative to Transapical Approach.’, *Journal of cardiac surgery*. United States, 30(5), pp. 381–390. doi: 10.1111/jocs.12527.

O’Connor, S. A. *et al.* (2015) ‘Revisiting Sex Equality With Transcatheter Aortic Valve Replacement Outcomes: A Collaborative, Patient-Level Meta-Analysis of 11,310 Patients.’, *Journal of the American College of Cardiology*. United States, 66(3), pp. 221–228. doi: 10.1016/j.jacc.2015.05.024.

Orlando, R. *et al.* (2013) ‘Cost-effectiveness of transcatheter aortic valve implantation (TAVI) for aortic stenosis in patients who are high risk or contraindicated for surgery: a model-based economic evaluation.’, *Health technology assessment (Winchester, England)*. England, 17(33), pp. 1–86. doi: 10.3310/hta17330.

Osnabrugge, R. L. J. *et al.* (2013) ‘Aortic stenosis in the elderly: disease prevalence and number of candidates for transcatheter aortic valve replacement: a meta-analysis and modeling study.’, *Journal of the American College of Cardiology*. United States, 62(11), pp. 1002–1012. doi: 10.1016/j.jacc.2013.05.015.

Overtchouk, P. and Modine, T. (2018) ‘A comparison of alternative access routes for transcatheter aortic valve implantation.’, *Expert review of cardiovascular therapy*. England, 16(10), pp. 749–756. doi: 10.1080/14779072.2018.1524295.

Pagnesi, M. *et al.* (2016) ‘Silent cerebral injury after transcatheter aortic valve implantation and the preventive role of embolic protection devices: A systematic review and meta-analysis.’, *International journal of cardiology*. Netherlands, 221, pp. 97–106. doi: 10.1016/j.ijcard.2016.06.143.

Panchal, H. B. *et al.* (2014) ‘A meta-analysis of mortality and major adverse cardiovascular and cerebrovascular events in patients undergoing transfemoral versus transapical transcatheter aortic valve implantation using edwards valve for severe aortic stenosis’, *American Journal of Cardiology*. T. Paul, Department of Internal Medicine, East Tennessee State University, Johnson City, TN, United States, United States, 114(12), pp. 1882–1890. doi: 10.1016/j.amjcard.2014.09.029.

Panchal, H. B. *et al.* (2013) ‘Thirty-day all-cause mortality in patients undergoing transcatheter aoritc valve implantation using edwards SAPIEN valve for severe aortic stenosis: A meta-analysis of transfemoral versus transapical approach’, *Circulation*. H.B. Panchal, Dept of Internal Medicine, East Tennessee State Univ, Johnson City, TN, United States, 128(22). Available at: <http://www.embase.com/search/results?subaction=viewrecord&from=export&id=L71339836>.

Patel, S.V. *et al.* (2017) ‘Outcomes of transcatheter aortic valve replacement for bicuspid aortic stenosis–a systematic review of existing literature’, *Expert Review of Pharmacoeconomics and Outcomes Research*. S.V. Patel, Sparks Health Systems, Internal Medicine Physician, 1001 Towson Avenue, Fort Smith, AR, United States, 17(6), pp. 579–585. doi: 10.1080/14737167.2017.1391692.

Phan, K. *et al.* (2015) ‘Early hemodynamic performance of the third generation St Jude Trifecta aortic prosthesis: A systematic review and meta-analysis’, *The Journal of thoracic and cardiovascular surgery*. United States, 149(6), pp. 1567–1575. doi: 10.1016/j.jtcvs.2015.01.043.

Phan, K. *et al.* (2015) ‘Transcatheter Aortic Valve Implantation (TAVI) in Patients With Bicuspid Aortic Valve Stenosis - Systematic Review and Meta-Analysis’, *Heart Lung and Circulation*. T.D. Yan, Macquarie University Hospital, Sydney, Australia, Australia, 24(7), pp. 649–659. doi: 10.1016/j.hlc.2014.12.163.

Pieters, F. A. *et al.* (1993) ‘Risk of aortic dissection after aortic valve replacement.’, *The American journal of cardiology*. United States, 72(14), pp. 1043–1047.

Powell, R. *et al.* (2017) ‘The Perceval Sutureless Aortic Valve: Review of Outcomes, Complications, and Future Direction.’, *Innovations (Philadelphia, Pa.)*. United States, 12(3), pp. 155–173. doi: 10.1097/IMI.0000000000000372.

Prasitlumkum, N. *et al.* (2019) ‘Previous coronary artery bypass graft is not associated with higher mortality in  transcatheter aortic valve replacement: systemic review and meta-analysis.’, *Acta cardiologica*. England, pp. 1–9. doi: 10.1080/00015385.2018.1541845.

Rafique, A. M. *et al.* (2009) ‘Meta-Analysis of Prognostic Value of Stress Testing in Patients With Asymptomatic Severe Aortic Stenosis’, *American Journal of Cardiology*. R.J. Siegel, Division of Cardiology, Cedars-Sinai Heart Institute, Los Angeles, CA, United States, United States, 104(7), pp. 972–977. doi: 10.1016/j.amjcard.2009.05.044.

Rahnavardi, M. *et al.* (2012) ‘A systematic review of transapical aortic valve implantation.’, *Annals of cardiothoracic surgery*. China, 1(2), pp. 116–128. doi: 10.3978/j.issn.2225-319X.2012.07.04.

Rashid, H. N. *et al.* (2018) ‘Bioprosthetic aortic valve leaflet thrombosis detected by multidetector computed  tomography is associated with adverse cerebrovascular events: a meta-analysis of observational studies.’, *EuroIntervention : journal of EuroPCR in collaboration with the Working Group on  Interventional Cardiology of the European Society of Cardiology*. France, 13(15), pp. e1748–e1755. doi: 10.4244/EIJ-D-17-01062.

Rashid, H. N. *et al.* (2018) ‘The prevalence of computed tomography-defined leaflet thrombosis in intra- versus supra-annular transcatheter aortic valve prostheses.’, *Catheterization and cardiovascular interventions : official journal of the Society for Cardiac Angiography & Interventions*. United States, 92(7), pp. 1414–1416. doi: 10.1002/ccd.27702.

Reddy, G.; Wang, Z. & Holmes, D. R. (2017) ‘Transcatheter aortic valve replacement for stenotic bicuspid aortic valves: Meta analysis of observational studies’, *Catheterization and Cardiovascular Interventions*. G. Reddy, Mayo Clinic, United States, 89, p. S200. doi: 10.1002/ccd.27053.

Reddy, G. *et al.* (2018) ‘Transcatheter aortic valve replacement for stenotic bicuspid aortic valves: Systematic review and meta analyses of observational studies’, *Catheterization and Cardiovascular Interventions*. D.R. Holmes, Department of Cardiovascular Diseases, Mayo Clinic, Rochester, MN, United States, 91(5), pp. 975–983. doi: 10.1002/ccd.27340.

Regueiro, A. *et al.* (2016) ‘Impact of New-Onset Left Bundle Branch Block and Periprocedural Permanent Pacemaker Implantation on Clinical Outcomes in Patients Undergoing Transcatheter Aortic Valve Replacement: A Systematic Review and Meta-Analysis.’, *Circulation. Cardiovascular interventions*. United States, 9(5), p. e003635. doi: 10.1161/CIRCINTERVENTIONS.115.003635.

Ren, B. *et al.* (2018) ‘Right ventricular systolic function in patients undergoing transcatheter aortic valve implantation: A systematic review and meta-analysis.’, *International journal of cardiology*. Netherlands, 257, pp. 40–45. doi: 10.1016/j.ijcard.2018.01.117.

Ribeiro, H. B. *et al.* (2013) ‘Coronary obstruction following transcatheter aortic valve implantation: a systematic review.’, *JACC. Cardiovascular interventions*. United States, 6(5), pp. 452–461. doi: 10.1016/j.jcin.2012.11.014.

Rojas, P. *et al.* (2016) ‘Acquired Aseptic Intracardiac Shunts Following Transcatheter Aortic Valve Replacement: A Systematic Review.’, *JACC. Cardiovascular interventions*. United States, 9(24), pp. 2527–2538. doi: 10.1016/j.jcin.2016.09.034.

Shehada, S.-E. *et al.* (2017) ‘Impact of previous cardiac surgery in patients undergoing transcatheter aortic valve implantation: A systematic review’, *Journal of Cardiovascular Surgery*. S.-E. Shehada, Department of Thoracic and Cardiovascular Surgery, West-German Heart and Vascular Center Essen, University of Duisburg-Essen, Hufelandstraße 55, Essen, Germany, 58(5), pp. 787–793. doi: 10.23736/S0021-9509.17.09636-7.

Sa, M. P. B. de O. *et al.* (2019) ‘Surgical aortic valve replacement and patient-prosthesis mismatch: a meta-analysis of 108 182 patients.’, *European journal of cardio-thoracic surgery : official journal of the European Association for Cardio-thoracic Surgery*. Germany, 56(1), pp. 44–54. doi: 10.1093/ejcts/ezy466.

Sa, M. P. B. O. *et al.* (2019) ‘Impact of surgical aortic root enlargement on the outcomes of aortic valve replacement: a meta-analysis of 13 174 patients.’, *Interactive cardiovascular and thoracic surgery*. England, 29(1), pp. 74–82. doi: 10.1093/icvts/ivy364.

Saad, M. *et al.* (2018) ‘Long-Term Outcomes With Transcatheter Aortic Valve Replacement in Women Compared  With Men: Evidence From a Meta-Analysis.’, *JACC. Cardiovascular interventions*. United States, 11(1), pp. 24–35. doi: 10.1016/j.jcin.2017.08.015.

Sankaramangalam, K. *et al.* (2017) ‘Impact of Coronary Artery Disease on 30-Day and 1-Year Mortality in Patients Undergoing Transcatheter Aortic Valve Replacement: A Meta-Analysis.’, *Journal of the American Heart Association*. England, 6(10). doi: 10.1161/JAHA.117.006092.

Sannino, A. *et al.* (2014) ‘Increased mortality after transcatheter aortic valve implantation (TAVI) in patients with severe aortic stenosis and low ejection fraction: a meta-analysis of 6898 patients.’, *International journal of cardiology*. Netherlands, 176(1), pp. 32–39. doi: 10.1016/j.ijcard.2014.06.017.

Sannino, A. *et al.* (2016) ‘A meta-analysis of the impact of pre-existing and new-onset atrial fibrillation on clinical outcomes in patients undergoing transcatheter aortic valve implantation.’, *EuroIntervention : journal of EuroPCR in collaboration with the Working Group on  Interventional Cardiology of the European Society of Cardiology*. France, 12(8), pp. e1047–e1056. doi: 10.4244/EIJY15M11_12.

Sannino, A. *et al.* (2015) ‘Meta-analysis of mortality outcomes and mitral regurgitation evolution in 4,839 patients having transcatheter aortic valve implantation for severe aortic stenosis’, *American Journal of Cardiology*. A. Sannino, Division of Cardiology, Department of Advanced Biomedical Sciences, Federico II University, Naples, Italy, United States, 114(6), pp. 875–882. doi: 10.1016/j.amjcard.2014.06.022.

Saung, M.T. *et al.* (2019) ‘Outcomes Following Balloon Aortic Valvuloplasty Versus Surgical Valvotomy in Congenital Aortic Valve Stenosis: A Meta-Analysis’, *The Journal of invasive cardiology*, 31(6), pp. E133–E142. Available at: <http://www.embase.com/search/results?subaction=viewrecord&from=export&id=L628110836>.

Saxena, A. *et al.* (2017) ‘Systematic review and meta-analysis on the impact of preoperative atrial fibrillation on short- and long-term outcomes after aortic valve replacement.’, *The Journal of cardiovascular surgery*. Italy, 58(6), pp. 943–950. doi: 10.23736/S0021-9509.17.09814-7.

Shan, L. *et al.* (2013) ‘A systematic review on the quality of life benefits after aortic valve replacement in the elderly.’, *The Journal of thoracic and cardiovascular surgery*. United States, pp. 1173–1189. doi: 10.1016/j.jtcvs.2013.01.004.

Sharma, M.; Eskandari, M. & Marwick, T. (2015) ‘Hypertension in aortic stenosis: A systematic review’, *Heart Lung and Circulation*. M. Sharma, Alfred Hospital, Melbourne, Australia, 24, p. S127. doi: 10.1016/j.hlc.2015.06.031.

Sharma, U. C. *et al.* (2004) ‘Systematic review of the outcome of aortic valve replacement in patients with aortic stenosis.’, *The Annals of thoracic surgery*. Netherlands, 78(1), pp. 90–95. doi: 10.1016/j.athoracsur.2004.02.020.

Shehada, S.-E. *et al.* (2017) ‘Minimal access versus conventional aortic valve replacement: a meta-analysis of propensity-matched studies.’, *Interactive cardiovascular and thoracic surgery*. England, 25(4), pp. 624–632. doi: 10.1093/icvts/ivx212.

Shinn, S.H. *et al.* (2018) ‘A Systemic Review and Meta-Analysis of Sutureless Aortic Valve Replacement Versus Transcatheter Aortic Valve Implantation’, *Annals of Thoracic Surgery*. S.H. Shinn, Department of Thoracic and Cardiovascular Surgery, Cheju Halla General Hospital, Doreongno 65, Jeju, Jeju Special Self-Governing Province, South Korea, 106(3), pp. 924–929. doi: 10.1016/j.athoracsur.2018.03.059.

Siontis, G. C. M. *et al.* (2014) ‘Predictors of permanent pacemaker implantation in patients with severe aortic stenosis undergoing TAVR: a meta-analysis.’, *Journal of the American College of Cardiology*. United States, 64(2), pp. 129–140. doi: 10.1016/j.jacc.2014.04.033.

Siontis, G. C. M. *et al.* (2018) ‘New-onset arrhythmias following transcatheter aortic valve implantation: a systematic review and meta-analysis.’, *Heart (British Cardiac Society)*. England, 104(14), pp. 1208–1215. doi: 10.1136/heartjnl-2017-312310.

Soud, M. *et al.* (2019) ‘Usefulness of skeletal muscle area detected by computed tomography to predict mortality in patients undergoing transcatheter aortic valve replacement: a meta-analysis study.’, *The international journal of cardiovascular imaging*. United States, 35(6), pp. 1141–1147. doi: 10.1007/s10554-019-01582-0.

Stangl, V. *et al.* (2014) ‘Influence of sex on outcome following transcatheter aortic valve implantation (TAVI): systematic review and meta-analysis.’, *Journal of interventional cardiology*. United States, 27(6), pp. 531–539. doi: 10.1111/joic.12150.

Sterling, L. H. *et al.* (2015) ‘Pharmacological management strategies for stroke prevention following transcatheter aortic valve replacement: A systematic review.’, *International journal of cardiology*. Netherlands, 191, pp. 303–311. doi: 10.1016/j.ijcard.2015.04.226.

Stewart, R. A. H. (2009) ‘Clinical trials in heart valve disease.’, *Current opinion in cardiology*. United States, 24(4), pp. 279–287.

Stonier, T., Harrison, M. and Choong, A. M. T. L. (2016) ‘A systematic review of transcatheter aortic valve implantation via carotid artery access.’, *International journal of cardiology*. Netherlands, 219, pp. 41–55. doi: 10.1016/j.ijcard.2016.05.049.

Straiton, N. *et al.* (2018) ‘Functional capacity and health-related quality of life outcomes post transcatheter aortic valve replacement: a systematic review and meta-analysis.’, *Age and ageing*. England, 47(3), pp. 478–482. doi: 10.1093/ageing/afx203.

Suchá, D. *et al.* (2015) ‘Does the aortic annulus undergo conformational change throughout the cardiac cycle? A systematic review’, *European Heart Journal Cardiovascular Imaging*. D. Suchá, Department of Radiology, University Medical Center Utrecht, Heidelberglaan 100, Utrecht, Netherlands, England, 16(12), pp. 1307–1317. doi: 10.1093/ehjci/jev210.

Sun, Y. *et al.* (2017) ‘Meta-analysis of Predictors of Early Severe Bleeding in Patients Who Underwent Transcatheter Aortic Valve Implantation.’, *The American journal of cardiology*. United States, 120(4), pp. 655–661. doi: 10.1016/j.amjcard.2017.05.035.

Sun, Y. *et al.* (2017) ‘Meta-Analysis of Impact of Diabetes Mellitus on Outcomes After Transcatheter Aortic Valve Implantation.’, *The American journal of cardiology*. United States, 119(4), pp. 623–629. doi: 10.1016/j.amjcard.2016.10.048.

Takagi, H., Ando, T. and Umemoto, T. (2017) ‘Direct and adjusted indirect comparisons of perioperative mortality after sutureless or rapid-deployment aortic valve replacement versus transcatheter aortic valve implantation.’, *International journal of cardiology*. Netherlands, 228, pp. 327–334. doi: 10.1016/j.ijcard.2016.11.253.

Takagi, H. *et al.* (2019) ‘Impact of concurrent tricuspid regurgitation on mortality after transcatheter aortic-valve implantation.’, *Catheterization and cardiovascular interventions : official journal of the Society for Cardiac Angiography & Interventions*. United States, 93(5), pp. 946–953. doi: 10.1002/ccd.27948.

Takagi, H. *et al.* (2019) ‘Meta-Analysis of Impact of Baseline N-TerminalPro-Brain Natriuretic Peptide Levels on SurvivalAfter Transcatheter Aortic Valve Implantation for Aortic Stenosis.’, *The American journal of cardiology*. United States, 123(5), pp. 820–826. doi: 10.1016/j.amjcard.2018.11.030.

Takagi, H. and Umemoto, T. (2017) ‘Better midterm survival in women after transcatheter aortic valve implantation.’, *The Journal of cardiovascular surgery*. Italy, 58(4), pp. 624–632. doi: 10.23736/S0021-9509.16.09382-4.

Takagi, H. and Umemoto, T. (2016) ‘Impact of paravalvular aortic regurgitation after transcatheter aortic valve implantation on survival.’, *International journal of cardiology*. Netherlands, 221, pp. 46–51. doi: 10.1016/j.ijcard.2016.07.006.

Takagi, H. and Umemoto, T. (2015) ‘Coexisting Mitral Regurgitation Impairs Survival After Transcatheter Aortic Valve Implantation.’, *The Annals of thoracic surgery*. Netherlands, 100(6), pp. 2270–2276. doi: 10.1016/j.athoracsur.2015.05.094.

Takagi, H. *et al.* (2016) ‘Sutureless aortic valve replacement may improve early mortality compared with transcatheter aortic valve implantation: A meta-analysis of comparative studies’, *Journal of Cardiology*. H. Takagi, Department of Cardiovascular Surgery, Shizuoka Medical Center, 762-1 Nagasawa, Shimizu-cho, Sunto-gun, Shizuoka, Japan, Netherlands, 67(6), pp. 504–512. doi: 10.1016/j.jjcc.2015.09.009.

Tam, D. Y. *et al.* (2018) ‘Transcatheter valve-in-valve versus redo surgical aortic valve replacement for the treatment of degenerated bioprosthetic aortic valve: A systematic review and meta-analysis.’, *Catheterization and cardiovascular interventions : official journal of the Society for Cardiac Angiography & Interventions*. United States, 92(7), pp. 1404–1411. doi: 10.1002/ccd.27686.

Tan, J.L. *et al.* (2019) ‘Clinical outcomes of incidental findings in initial body computed tomography prior to transcatheter aortic valve replacement: A systematic review and meta-analysis’, *Catheterization and Cardiovascular Interventions*. J.L. Tan, Crozer-Chester Medical Center, United States, 93, p. S92. doi: 10.1002/ccd.28216.

Tang, M. *et al.* (2017) ‘Meta-Analysis of Outcomes and Evolution of Pulmonary Hypertension Before and After Transcatheter Aortic Valve Implantation.’, *The American journal of cardiology*. United States, 119(1), pp. 91–99. doi: 10.1016/j.amjcard.2016.09.015.

Testa, L. *et al.* (2018) ‘Cerebral protection during transcatheter aortic valve implantation: An updated systematic review and meta-analysis’, *Journal of the American Heart Association*. L. Testa, Department of Cardiology, IRCCS Pol. S. Donato, S. Donato Milanese, Milan, Italy, England, 7(10). doi: 10.1161/JAHA.117.008463.

Thongprayoon, C. *et al.* (2015) ‘Acute kidney injury after transcatheter aortic valve replacement: a systematic review and meta-analysis.’, *American journal of nephrology*. Switzerland, 41(4–5), pp. 372–382. doi: 10.1159/000431337.

Thongprayoon, C. *et al.* (2017) ‘Association of frailty status with acute kidney injury and mortality after transcatheter aortic valve replacement: A systematic review and meta-analysis.’, *PloS one*. United States, 12(5), p. e0177157. doi: 10.1371/journal.pone.0177157.

Tilley, E. *et al.* (2018) ‘Meta-analysis of Prevalence and Risk Factors for Delirium After Transcatheter Aortic Valve Implantation.’, *The American journal of cardiology*. United States, 122(11), pp. 1917–1923. doi: 10.1016/j.amjcard.2018.08.037.

Turgeon, R. D. and Barry, A. R. (2015) ‘Single vs Dual Antiplatelet Therapy Following Transcatheter Aortic Valve Implantation: A Systematic Review.’, *Clinical cardiology*. United States, 38(10), pp. 629–634. doi: 10.1002/clc.22426.

Useini, D. *et al.* (2019) ‘Mid-Term Outcomes after Transapical and Transfemoral Transcatheter Aortic Valve Implantation for Aortic Stenosis and Porcelain Aorta with a Systematic Review of Transfemoral versus Transapical Approach.’, *The Thoracic and cardiovascular surgeon*. Germany. doi: 10.1055/s-0039-1692719.

Van Brabandt, H. *et al.* (2009) ‘Safety of percutaneous aortic valve insertion. A systematic review’, *BMC Cardiovascular Disorders*. H. Van Brabandt, Belgian Health Care Knowledge Centre, Administratief Centrum Kruidtuin, Kruidtuinlaan 55, 1000 Brussels, Belgium, England, 9, p. 45. doi: 10.1186/1471-2261-9-45.

Varela-Lema, L., De La Fuente Cid, R. and Lopez Garcia, M. L. (2013) ‘[Sutureless aortic valve replacement for high surgical risk patients with aortic  stenosis: systematic review].’, *Medicina clinica*. Spain, 140(3), pp. 119–127. doi: 10.1016/j.medcli.2012.05.022.

Vasques, F. *et al.* (2012) ‘Outcome of patients aged >/=80 years undergoing combined aortic valve replacement and coronary artery bypass grafting: a systematic review and meta-analysis of 40 studies.’, *American heart journal*. United States, 164(3), pp. 410-418.e1. doi: 10.1016/j.ahj.2012.06.019.

Vasques, F. *et al.* (2012) ‘Immediate and late outcome of patients aged 80 years and older undergoing isolated aortic valve replacement: a systematic review and meta-analysis of 48 studies.’, *American heart journal*. United States, 163(3), pp. 477–485. doi: 10.1016/j.ahj.2011.12.005.

Vavuranakis, M. *et al.* (2016) ‘Dual or Single Antiplatelet Therapy After Transcatheter Aortic Valve Implantation? A Systematic Review and Meta-Analysis’, *Current pharmaceutical design*. United Arab Emirates, 22(29), pp. 4596–4603. Available at: <http://www.embase.com/search/results?subaction=viewrecord&from=export&id=L619728528>.

Villablanca, P. A. *et al.* (2017) ‘Bivalirudin versus heparin in patients undergoing percutaneous transcatheter aortic valve interventions: A systematic review and meta-analysis.’, *Journal of interventional cardiology*. United States, 30(6), pp. 586–594. doi: 10.1111/joic.12428.

Villablanca, P. A. *et al.* (2018) ‘Comparison of local versus general anesthesia in patients undergoing transcatheter aortic valve replacement: A meta-analysis.’, *Catheterization and cardiovascular interventions : official journal of the Society for Cardiac Angiography & Interventions*. United States, 91(2), pp. 330–342. doi: 10.1002/ccd.27207.

Vinco, G. *et al.* (2013) ‘Meta-analysis of the outcome of patients with low gradient severe aortic stenosis and preserved left ventricular ejection fraction’, *International Journal of Cardiology*. C. Bergamini, Division of Cardiology, Department of Medicine, University of Verona, P.le Stefani 1, 37129 Verona, Italy, 168(5), pp. 5076–5078. doi: 10.1016/j.ijcard.2013.07.193.

Wang, J. *et al.* (2017) ‘Risk Factors for Post-TAVI Bleeding According to the VARC-2 Bleeding Definition and Effect of the Bleeding on Short-Term Mortality: A Meta-analysis.’, *The Canadian journal of cardiology*. England, 33(4), pp. 525–534. doi: 10.1016/j.cjca.2016.12.001.

Wang, N. and Lal, S. (2017) ‘Post-dilation in transcatheter aortic valve replacement: A systematic review and  meta-analysis.’, *Journal of interventional cardiology*. United States, 30(3), pp. 204–211. doi: 10.1111/joic.12378.

Wang, T. K. M. *et al.* (2017) ‘Performance of contemporary surgical risk scores for transcatheter aortic valve implantation: A meta-analysis.’, *International journal of cardiology*. Netherlands, 236, pp. 350–355. doi: 10.1016/j.ijcard.2016.12.188.

Winter, M.-P. *et al.* (2018) ‘Normal values for Doppler echocardiographic assessment of prosthetic valve function after transcatheter aortic valve replacement: a systematic review and meta-analysis.’, *European heart journal cardiovascular Imaging*. England, 19(4), pp. 361–368. doi: 10.1093/ehjci/jex212.

Witburg G.*et al.* (2018) ‘Impact of coronary artery revascularization completeness on outcomes of patients with coronary artery disease undergoing transcatheter aortic valve replacement a meta-analysis of studies using the residual syntax score (synergy between PCI with taxus and cardiac surgery) ’, *Circulation: Cardiovascular Interventions*. G. Witberg, Sackler Faculty of Medicine, Tel-Aviv University, Israel, 11(3). doi: 10.1161/CIRCINTERVENTIONS.117.006000.

Witkowski, A. *et al.* (2014) ‘Second transcatheter aortic valve implantation for treatment of suboptimal function of previously implanted prosthesis: review of the literature.’, *Journal of interventional cardiology*. United States, 27(3), pp. 300–307. doi: 10.1111/joic.12120.

Wollersheim, L. W. *et al.* (2015) ‘Aortic Valve Replacement With the Stentless Freedom SOLO Bioprosthesis: A Systematic Review.’, *The Annals of thoracic surgery*. Netherlands, 100(4), pp. 1496–1504. doi: 10.1016/j.athoracsur.2015.06.048.

Xie, X. *et al.* (2016) ‘Efficacy and Safety of Transcatheter Aortic Valve Implantation for Bicuspid Aortic Valves: A Systematic Review and Meta-Analysis’, *Annals of thoracic and cardiovascular surgery : official journal of the Association of Thoracic and Cardiovascular Surgeons of Asia*. Japan, 22(4), pp. 203–215. doi: 10.5761/atcs.ra.16-00032.

Xiong, T. *et al.* (2015) ‘Causes of Death Following Transcatheter Aortic Valve Replacement: A Systematic Review and Meta-Analysis.’, *Journal of the American Heart Association*. England, 4(9), p. e002096. doi: 10.1161/JAHA.115.002096.

Yan, T. D. *et al.* (2010) ‘Transcatheter aortic valve implantation for high-risk patients with severe aortic stenosis: A systematic review.’, *The Journal of thoracic and cardiovascular surgery*. United States, 139(6), pp. 1519–1528. doi: 10.1016/j.jtcvs.2009.08.037.

Yanagawa, B. *et al.* (2019) ‘Management of Less-Than-Severe Aortic Stenosis during Coronary Bypass: A Systematic Review and Meta-Analysis.’, *Innovations (Philadelphia, Pa.)*. United States, p. 1556984519849639. doi: 10.1177/1556984519849639.

Yang, Y. *et al.* (2017) ‘The safety of concomitant transcatheter aortic valve replacement and percutaneous coronary intervention’, *Medicine (United States)*. Y. Feng, Department of Cardiology, West China Hospital, Sichuan University, 37 Guoxue Street, Chengdu, Sichuan, China, United States, 96(48), p. e8919. doi: 10.1097/MD.0000000000008919.

Yousef, A. *et al.* (2018) ‘Transcatheter Aortic Valve Implantation (TAVI) for Native Aortic Valve Regurgitation- A Systematic Review.’, *Circulation journal : official journal of the Japanese Circulation Society*. Japan, 82(3), pp. 895–902. doi: 10.1253/circj.CJ-17-0672.

Yousef, A. *et al.* (2014) ‘Performance of transcatheter aortic valve implantation in patients with bicuspid  aortic valve: systematic review.’, *International journal of cardiology*. Netherlands, pp. 562–564. doi: 10.1016/j.ijcard.2014.07.013.

Zhao, Z.-G. *et al.* (2013) ‘Sex-Related differences in outcomes after transcatheter aortic valve implantation a systematic review and meta-analysis’, *Circulation: Cardiovascular Interventions*. Z.-G. Zhao, Department of Cardiology, West China Hospital, Sichuan University, 37 Guoxue St, Chengdu 610041, China, United States, 6(5), pp. 543–551. doi: 10.1161/CIRCINTERVENTIONS.111.000519.

Zhang, R. *et al.* (2013) ‘Comparison of Aortic Annulus Diameter Measurement between Multi-Detector Computed Tomography and Echocardiography: A Meta-Analysis’, *PLoS ONE*. R. Zhang, Department of Ultrasound, The First Affiliated Hospital of Zhengzhou University, Zhengzhou, Henan Province, China, United States, 8(3), p. e58729. doi: 10.1371/journal.pone.0058729.

Zhang, S. and Kolominsky-Rabas, P. L. (2017) ‘How TAVI registries report clinical outcomes-A systematic review of endpoints based on VARC-2 definitions.’, *PloS one*. United States, 12(9), p. e0180815. doi: 10.1371/journal.pone.0180815.

Zhao, A. *et al.* (2015) ‘A meta-analysis of transfemoral versus transapical transcatheter aortic valve implantation on 30-day and 1-year outcomes.’, *The heart surgery forum*. United States, 18(4), pp. E161-6. doi: 10.1532/hsf.1388.

Zheng, Q. *et al.* (2017) ‘Effects of Aortic Valve Replacement on Severe Aortic Stenosis and Preserved Systolic Function: Systematic Review and Network Meta-analysis’, *Scientific reports*, 7(1), p. 5092. doi: 10.1038/s41598-017-05021-9.

**Not aortic stenosis (n=0):**

/

**Risk for surgery (High risk excluded >20% EUROSCORE & >8% STS) (n=6):**

Arora, S. *et al.* (2017) ‘Review of Major Registries and Clinical Trials of Late Outcomes After Transcatheter Aortic Valve Replacement.’, *The American journal of cardiology*. United States, 120(2), pp. 331–336. doi: 10.1016/j.amjcard.2017.04.029.

Boothroyd, L. J. *et al.* (2013) ‘Transcatheter Aortic Valve Implantation: Recommendations for Practice Based on a Multidisciplinary Review Including Cost-Effectiveness and Ethical and Organizational Issues’, *Canadian Journal of Cardiology*. L.J. Boothroyd, INESSS, 2021 Union Avenue, Suite 10.083, Montréal, QC H3A 2S9, Canada, England, 29(6), pp. 718–726. doi: 10.1016/j.cjca.2012.09.002.

Chakos, A. *et al.* (2017) ‘Long term outcomes of transcatheter aortic valve implantation (TAVI): a systematic review of 5-year survival and beyond.’, *Annals of cardiothoracic surgery*. China, 6(5), pp. 432–443. doi: 10.21037/acs.2017.09.10.

Kim, C.A. *et al.* (2013) ‘Transcatheter aortic valve replacement improves functional status and quality of life in elderly patients with severe aortic stenosis’, *Circulation*. C.A. Kim, Medicine, Beth Israel Deaconess Med Cntr, Harvard Med Sch, Boston, MA, United States, 128(22). Available at: <http://www.embase.com/search/results?subaction=viewrecord&from=export&id=L71339826>.

Liu, Z. *et al.* (2018) ‘Transcatheter aortic valve implantation for aortic stenosis in high surgical risk patients: A systematic review and meta-analysis.’, *PloS one*. United States, 13(5), p. e0196877. doi: 10.1371/journal.pone.0196877.

S.-N., G. *et al.* (2012) ‘Transcatheter aortic valve implantation reduces neither 30-day nor midterm mortality over aortic valve replacement in high-risk patients: A meta-analysis of randomized and adjusted observational studies’, *Circulation*. S.-N. Goto, Cardiovascular Surgery, Shizuoka Med. Cntr., Sunto-gun, Japan, 126(21). Available at: http://www.embase.com/search/results?subaction=viewrecord&from=export&id=L70957450.

**No risk stratification (n=16):**

Ak, A. *et al.* (2018) ‘Transcatheter vs. surgical aortic valve replacement and medical treatment: Systematic review and meta-analysis of randomized and non-randomized trials’, *Herz*. P. Schlattmann, Institute of Medical Statistics, Informatics and Documentation (IMSID), Friedrich-Schiller University and University Hospital Jena, Bachstraße 18, Jena, Germany, Germany, 43(4), pp. 325–337. doi: 10.1007/s00059-017-4562-5.

Amato, L. *et al.* (2016) ‘[Transcatheter aortic valve implantation: a systematic review of the literature on efficacy and safety data].’, *Recenti progressi in medicina*. doi: 10.1701/2132.23102.

Arora, S. *et al.* (2018) ‘Meta-analysis of transfemoral TAVR versus surgical aortic valve replacement.’, *Catheterization and cardiovascular interventions : official journal of the Society for Cardiac Angiography & Interventions*. United States, 91(4), pp. 806–812. doi: 10.1002/ccd.27357.

Athappan, G. *et al.* (2014) ‘Influence of transcatheter aortic valve replacement strategy and valve design on stroke after transcatheter aortic valve replacement: A meta-analysis and systematic review of literature’, *Journal of the American College of Cardiology*. doi: 10.1016/j.jacc.2014.02.540.

Biondi-Zoccai, G. *et al.* (2014) ‘Network meta-analysis on the comparative effectiveness and safety of transcatheter aortic valve implantation with CoreValve or Sapien devices versus surgical replacement.’, *Heart, lung and vessels*.

Cao, C. *et al.* (2016) ‘Transcatheter Aortic Valve Implantation versus Surgical Aortic Valve Replacement: Meta-Analysis of Clinical Outcomes and Cost-Effectiveness.’, *Current pharmaceutical design*. United Arab Emirates, 22(13), pp. 1965–1977.

Carnero-Alcazar, M. *et al.* (2017) ‘Transcatheter versus surgical aortic valve replacement in moderate and high-risk  patients: a meta-analysis.’, *European journal of cardio-thoracic surgery : official journal of the European Association for Cardio-thoracic Surgery*. Germany, 51(4), pp. 644–652. doi: 10.1093/ejcts/ezw388.

Danielsen, S. O. *et al.* (2018) ‘Thirty-day readmissions in surgical and transcatheter aortic valve replacement: A systematic review and meta-analysis.’, *International journal of cardiology*. Netherlands, 268, pp. 85–91. doi: 10.1016/j.ijcard.2018.05.026.

Gargiulo, G. *et al.* (2016) ‘Transcatheter aortic valve implantation versus surgical aortic valve replacement: A Systematic review and meta-analysis’, *Annals of Internal Medicine*. doi: 10.7326/M16-0060.

Lytvyn, L. *et al.* (2016) ‘Patient values and preferences on transcatheter or surgical aortic valve replacement therapy for aortic stenosis: a systematic review’, *BMJ Open*. doi: 10.1136/bmjopen-2016-014327.
[truncated: 20,937 more chars]
